# Mechanistic elucidation of Wuling Powder targeting macrophage polarization to ameliorate renal ischemia-reperfusion injury via multidimensional computational systems pharmacology coupled with experimental validation

**DOI:** 10.3389/fphar.2026.1811709

**Published:** 2026-06-04

**Authors:** Qian-Qian Wang, Yu-Fan Gong, Zi-Feng Wang, Xing Pu, Xiang Li, Lin Bai, Jie Zhang, Juan Xie, Jing Li, Wei Jiang, Li-Min Liu, Jia-Wei Zuo, Ying-Yong Zhao, Dong-Hui Zheng, Hai-Lun Li

**Affiliations:** 1 Department of Nephrology, The Affiliated Huai’an Hospital of Xuzhou Medical University and The Second People’s Hospital of Huai’an, Huai’an, Jiangsu, China; 2 Department of Nephrology, Jiangdu People’s Hospital Affiliated to Yangzhou University, Yangzhou, Jiangsu, China; 3 Faculty of Life Science and Medicine, Northwest University, Xi’an, Shaanxi, China; 4 Department of Radiotherapy, The Affiliated Huai’an Hospital of Xuzhou Medical University and The Second People’s Hospital of Huai’an, Huai’an, Jiangsu, China; 5 School of Pharmaceutical Sciences, Zhejiang Chinese Medical University, Hangzhou, Zhejiang, China

**Keywords:** renal ischemia-reperfusion injury, macrophage polarization, Wuling Powder, mechanism of action, Urokinase Plasminogen Activator Receptor

## Abstract

**Introduction:**

The blurry mechanism of Wuling Powder (WLP) in treating renal ischemia-reperfusion injury (R–IRI) has greatly hindered its clinical promotion and application as well as the development of modern preparations. This study applied computational systems pharmacology combined with experimental validation to reveal the mechanism of Wuling Powder (WLP) in ameliorating renal ischemia reperfusion injury (R–IRI) by regulating macrophage polarization (MP).

**Methods:**

Firstly, the core target mechanism of WLP in treating R–IRI by regulating MP was clearly identified through using multidimensional AI computational systems pharmacology, including network pharmacology, the Gene Expression Omnibus database analysis, machine learning, molecular docking etc.

**Results:**

As a result, the core target of WLP in improving MP for treating R–IRI was confirmed to be PLAUR, HSPA1A, and FOS. Then, molecular dynamics simulation revealed that the 3,4–Dihydroxybenzaldehyde–PLAUR complex, which exhibited the least negative docking score, could still bind stably. On this basis, in vivo experiments combined with multivariate statistics and correlation analysis further demonstrated that WLP and its main active compound 3,4–Dihydroxybenzaldehyde could significantly modulate MP by regulating PLAUR to improve R–IRI.

**Discussion:**

These findings collectively support PLAUR, HSPA1A, and FOS as core therapeutic targets through which WLP modulates MP to alleviate R–IRI. This systematic research provides a solid theoretical basis for the clinical application and modern drug development of WLP in the prevention and treatment of R–IRI.

## 1 Introduction

Renal ischemia-reperfusion injury (R-IRI) is a prevalent and pathophysiological severe condition characterized by the restoration of blood flow to renal parenchymal tissue following acute ischemia, thereby initiating a coordinated cascade of cellular and molecular responses—including oxidative stress, robust inflammatory activation, and programmed cell death (Nieuwenhuijs-Moeke et al., 2020; Song et al., 2023; Huang et al., 2024). As a leading cause of acute kidney injury (AKI) in clinical contexts, including cardiovascular surgery, circulatory shock, and renal transplantation, R-IRI not only precipitates acute renal dysfunction but also confers an elevated risk of progression to chronic kidney disease and end-stage renal disease (Chen and Yang, 2025; Liang et al., 2025). Despite substantial advances in supportive care and symptomatic treatments, effective strategies for the prevention and management of R-IRI remain limited (Kell et al., 2023; Pan and Zhu, 2026), underscoring the urgent need to identify novel therapeutic targets and develop candidate pharmacotherapies.

As key innate immune effectors infiltrating the injured renal parenchyma, macrophages play a pivotal regulatory role across the initiation, progression, and resolution phases of R-IRI (Lu et al., 2026; Rossi et al., 2021). During the progression of R-IRI, macrophage polarization (MP) can transform macrophages into distinct M1 and M2 subtypes, which determines the exacerbation or alleviation of the inherent inflammatory response (Murray, 2017; Zhao et al., 2021). Amid the harsh, oxygen-starved terrain of ischemic injury, infiltrating macrophages undergo a decisive shift toward the pro-inflammatory M1 phenotype, unleashing a potent storm of cytokines, including tumor necrosis factor-α (TNF-α), interleukin-1β (IL-1β), and interleukin-6. This fiery cascade not only inflicts direct assault on renal tubular epithelial cells but also fans the flames of local inflammation, transforming the microenvironment into a crucible of tissue destruction (Wei et al., 2025; Hu et al., 2021). On the contrary, M2-polarized macrophages promote the secretion of anti-inflammatory cytokines, such as interleukin-10 and interleukin-4, thereby facilitating tissue repair, angiogenesis, and the resolution of inflammation (Wu Y. et al., 2025; Sun et al., 2022). A growing body of evidence supports the therapeutic potential of modulating MP from a pro-inflammatory M1 to a reparative M2 phenotype as a promising strategy to attenuate R-IRI (Gouda et al., 2023).

Traditional Chinese medicine (TCM) is widely used for the treatment of renal disease (Cai et al., 2025; Acquah-Mills et al., 2025; Yuan et al., 2025; Wu X. et al., 2025; Miao et al., 2025; Feng et al., 2025; Wang et al., 2023a), and its unique theoretical framework—grounded in holistic principles and dynamic balance—combined with abundant clinical practice experience, underpins its distinctive capacity to sustain bodily homeostasis (Wu et al., 2023). Wuling Powder (WLP) is a classic formula originally recorded in *Treatise on Febrile and Miscellaneous Diseases* compiled by Zhang Zhongjing in the Eastern Han Dynasty. It is composed of five medicinal herbs in a ratio of 9:6:9:9:15, including the Fuling (*Poria cocos* (Schw.) Wolf), Guizhi (*Cinnamomum cassia* Presl), Baizhu (*Atractylodes macrocephala* Koidz), Zhuling (*Polyporus umbellatus* (Pers.) Fries), and Zexie (*Alisma orientalis* (Sam.) Juzep) (Guo et al., 2025; Zhang et al., 2019; Tian et al., 2014; Ren et al., 2025; Luo et al., 2025). According to TCM theory, WLP exerts three principal pharmacological actions, such as promoting diuresis to eliminate dampness, invigorating the Spleen to tonify Qi, and warming Kidney-Yang to restore Qi transformation (Zeng et al., 2025; Yang et al., 2022; Lee et al., 2024). These effects that closely correspond to the core pathophysiological features of R-IRI, which is characterized by splenic deficiency complicated by dampness stagnation and kidney-yang deficiency with water retention (Dou and Zhang, 2025). A substantial body of experimental evidence have demonstrated that WLP can significantly attenuate renal fibrosis, a critical pathological process linking R-IRI to end-stage renal disease, via anti-inflammatory mechanisms (Yang et al., 2022; Ru, 2023; Li et al., 2022). Based on this, we speculated that WLP might treat R-IRI by regulating MP. However, at present, this speculation still lacks experimental data to support it, as well as its underlying molecular mechanisms also remains to be fully elucidated. Accordingly, we hypothesize that Wuling Powder (WLP) may ameliorate renal ischemia-reperfusion injury (R-IRI) by regulating macrophage polarization through targeting PLAUR, HSPA1A, and FOS. The blurry mechanism of WLP in treating R-IRI has greatly hindered its clinical promotion and application as well as the development of modern preparations.

In recent years, multidimensional artificial intelligence (AI) computational systems pharmacology, an integrated strategy incorporating molecular docking, network pharmacology and machine learning has emerged as a powerful tool for deciphering the sophisticated crosstalk between TCM formulae and biological systems (Wang X. et al., 2024). This technical platform facilitates the systematic identification of promising pharmacotherapeutic targets and the prediction of key regulatory pathways underlying the action of TCM formulae, thereby addressing the inherent limitations of conventional reductionist approaches in characterizing their multi-target, multi-component pharmacological profiles (Li et al., 2023).

**FIGURE 1:**
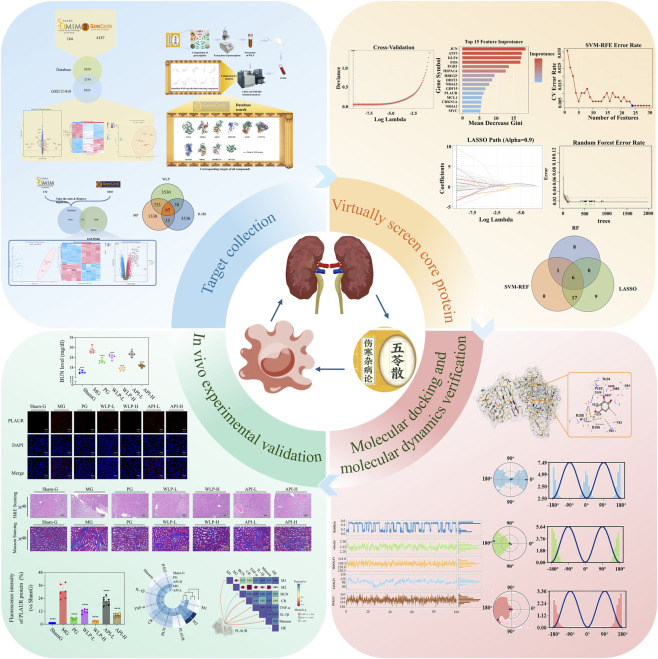
A schematic diagram illustrating the detailed workflow of the entire research approach.

In the current study, multidimensional AI computational systems pharmacology coupled with experimental validation strategies was employed to systematically examine the therapeutic effects and intrinsic mechanisms of WLP against R-IRI by regulating MP. Firstly, the core target mechanism of WLP in treating R-IRI by regulating MP was clearly identified through using multidimensional AI computational systems pharmacology including network pharmacology, the Gene Expression Omnibus (GEO) database analysis, machine learning, molecular docking, etc. Then, these core targets were systematically verified through molecular dynamics simulations and comprehensive *in vivo* experiments. Based on the above evidence and our preliminary network pharmacology analysis, we hypothesize that WLP exerts renoprotective effects against R-IRI by acting on its potential core targets, thereby driving MP toward an anti-inflammatory M2 phenotype and alleviating tubular damage, fibrosis, and systemic inflammation. The research workflow was illustrated in (Figure 1). Figure 1 schematically illustrates the overall research design, including component identification, target screening, machine learning, molecular docking, molecular dynamics simulation, and *in vivo* experimental validation. These pioneering and systematic findings provide a robust theoretical basis for the clinical translation and drug development of the classic TCM formula WLP in R-IRI therapy.

## 2 Experimental materials and methods

### 2.1 Experimental materials and reagents

All medicinal material crude slices of WLP conformed to the specifications of the Pharmacopoeia of the People’s Republic of China and were procured from the Bozhou Chinese Medicinal Materials Market in Anhui Province (Bozhou, China). The authenticity of medicinal material crude slices of WLP was identified by Professor Qingping Xiong from Huaiyin Institute of Technology. Dexamethasone was sourced from Pfizer Pharmaceuticals Ltd. (New York, USA). Assay kits for serum creatinine (CR) and blood urea nitrogen (BUN) were purchased from Nanjing Jiancheng Biotechnology Co., Ltd. (Nanjing, China). TNF-α and IL-1β were obtained from Meimian Biotechnology Co, Ltd. (Yancheng, China). Masson and Hematoxylin & Eosin (H&E) staining assay kits were purchased from Beijing Pulilai Gene Technology Co., Ltd. (Beijing, China) or Nanjing Jiancheng Biotechnology Co., Ltd. (Nanjing, China). All primary and secondary antibodies were sourced from Abcam Co., Ltd. (Cambridge, UK).

### 2.2 Potential targets prediction of main blood-entering components from WLP

#### 2.2.1 Preparation of WLP extract and identification of its blood-entering components

All medicinal materials are evenly mixed, pulverized, and passed through a 14-mesh sieve before being transferred to the extraction apparatus. An appropriate amount of purified water is added and allowed to soak for 30 min, followed by heating and decocting for 60 min before filtration. The filter residue is decocted twice again, and all filtrates are combined. After centrifugation, the entire supernatant is collected, concentrated to 300–500 mL, and finally lyophilized to obtain the product.

The lyophilized product is taken, mixed with 10 mL of 70% methanol, and subjected to ultrasonic extraction twice to obtain the supernatant. The supernatant was collected and evaporated to dryness under a stream of nitrogen. The residue was reconstituted in purified water to a final volume of 2 mL. This reconstituted solution was loaded onto a pre-activated C_18_ solid-phase extraction (SPE) cartridge. The cartridge was then washed sequentially with purified water, followed by elution with methanol. The eluate was collected and subjected to high-speed centrifugation. Subsequently, 2 µL of the supernatant was transferred and dried under a stream of nitrogen.

Blood was collected into EP tubes containing 10 μL of EDTA (0.09 g/mL) from the orbital venous plexus of mice at 10 time points. After centrifugation obtained plasma samples. Blank plasma was prepared following the same procedure. To each plasma sample, ice-cold methanol was added to precipitate proteins. After thorough centrifugation, the supernatant was collected and dried under a stream of nitrogen. The dried samples were reconstituted in 50% acetonitrile prior to injection, centrifuged again, and the supernatant was injected into the LC-MS for analysis.

WLP sample, rat plasma sample, and blank plasma sample were analyzed using UPLC, resulting in UPLC-QTOF-MS/MS for identification. The chromatographic conditions employed a mobile phase of acetonitrile and 0.1% formic acid aqueous solution, with a flow rate of 0.3 mL/min and an injection volume of 2 μL. Mass spectrometry conditions included an electrospray ionization (ESI) source, scanning in both positive and negative ion modes over a mass range of m/z 100–1,250. Based on retention time, accurate molecular weight, and MS/MS data, the blood-absorbed components in WLP were identified.

#### 2.2.2 Target prediction of blood-entering components of WLP

Based on the identified blood-entering components of WLP, their corresponding targets were obtained through database retrieval methods. First, the names of these blood-absorbed components were searched in the PubChem database (https://pubchem.ncbi.nlm.nih.gov/) to obtain their corresponding SMILES structures. Then, these SMILES notations were entered into the SwissTargetPrediction platform (https://www.swisstargetprediction.ch/) for target prediction, with the species parameter set to human (*Homo sapiens*). Only targets with a prediction probability greater than 0 were selected as potential targets for the bioactive components of WLP.

### 2.3 Identification of characteristic targets from R-IRI

#### 2.3.1 Identification of R-IRI related signature genes based on GEO dataset analysis

Gene expression profile data related to R-IRI were sifted out from the GEO (https://www.ncbi.nlm.nih.gov/geo/), a database hosted by the National Center for Biotechnology Information (NCBI). Using “Renal ischemia-reperfusion injury” as the key search term, the dataset GSE39548 was screened out, which consisted of 4 samples from the normal group and 4 samples from the R-IRI.

Raw gene expression data were subjected to differential analysis using the limma package implemented in R language (v4.5.2), where the filtering thresholds were set at |log_2_ fold change| > 1 and false discovery rate (FDR) < 0.05 after multiple testing correction. The differentially expressed genes (DEGs) obtained through this screening were identified as candidate disease targets associated with R-IRI.

#### 2.3.2 Screening key genes related to R-IRI through database retrieval

Using “Renal ischemia-reperfusion injury” as the keyword, disease-related target information was collected from the GeneCards database (https://www.genecards.org/) and the Online Mendelian Inheritance in Man (OMIM, https://www.omim.org/) database, with the species restricted to *H. sapiens*. The disease-related targets retrieved from the databases were then integrated with the aforementioned DEGs, followed by the removal of duplicate entries. All targets were uniformly converted into normalized gene names with unique identifiers via the UniProt database (https://www.uniprot.org/), which were considered as a characteristic gene of R-IRI for subsequent research.

### 2.4 Identification of MP targets

#### 2.4.1 Identification of MP-related characteristic genes based on GEO dataset analysis

Gene expression profile data linked to MP were retrieved from the GEO (https://www.ncbi.nlm.nih.gov/geo/), a database maintained by the National Center for Biotechnology Information (NCBI). Using “Macrophage polarization” as the search keyword, the dataset GSE121410 was screened out, consisting of 3 control group samples and 6 MP group samples.

Differential expression analysis of raw gene expression data was conducted via the limma package in R software (version 4.5.2), with filtering thresholds defined as false discovery rate (FDR) < 0.05 and |log_2_ fold change| > 1 following multiple testing correction. The DEGs obtained through this screening were designated as candidate disease targets associated with MP.

#### 2.4.2 Screening key genes related to MP through database retrieval

Using “Macrophage polarization” as the search term, disease-associated target information was retrieved from the GeneCards database (https://www.genecards.org/) and the OMIM database (https://www.omim.org/), with the species limited to *H. sapiens.* The disease-related targets retrieved from the two databases were integrated with the aforementioned DEGs, followed by the elimination of duplicate entries. Subsequently, all targets were uniformly converted into standardized gene names with unique identifiers via the UniProt database, which were regarded as a characteristic gene of MP for subsequent research.

### 2.5 Potential characteristic genes acquisition of WLP in regulating MP to improve R-IRI

Intersection analysis using Venn diagram in R software was conducted to obtain the common target genes of characteristic target genes of main blood-entering components from WLP, R-IRI-related genes and MP-related genes. The common target genes identified by intersection analysis were defined as potential characteristic genes of WLP to ameliorate R-IRI via regulating MP.

### 2.6 Functional enrichment analyses

Based on the above overlapping targets from 2.5 section obtained, GO functional enrichment and KEGG pathway enrichment analyses were conducted with the species set to “*H. sapiens*” using the clusterProfiler package in R. The GO analysis included three categories: biological process, cellular component, and molecular function. Results of the enrichment analyses were sorted by *P*-value. The circlize package was employed to generate chord diagrams for the GO enrichment results, displaying the top 8 significantly enriched functional terms. Meanwhile, the ggsankeyfier package was utilized to draw Sankey diagrams for the top 15 significantly enriched KEGG signaling pathways, so as to intuitively reflect the distribution characteristics of the targets at the functional and signaling pathway levels.

### 2.7 Screening of potential core hub genes via machine learning algorithms

To further discern potential core hub genes from the pool of potential characteristic genes of WLP to ameliorate R-IRI via regulating MP, multiple machine learning algorithms were integrated for feature selection, including Least Absolute Shrinkage and Selection Operator (LASSO) regression, Random Forest, and support vector machine-recursive feature elimination (SVM-RFE). All computational analyses were implemented in the R programming environment. The gene expression matrix corresponding to intersection targets was utilized as the input feature set, with sample grouping automatically assigned according to the “_con” and “_tre” suffixes embedded in sample identifiers.

A binary logistic regression model for LASSO regression was constructed applying the glmnet package, with the regularization framework set to Elastic Net (α = 0.9). The optimal penalty parameter λ was selected through 10-fold cross-validation, and genes corresponding to λ min in the model exhibiting non-zero regression coefficients were designated as candidate features. For Random Forest analysis, a classification model was established with the Random Forest package, where the number of decision trees (ntree) was fixed at 2,000 and the mtry parameter was dynamically adjusted according to the total amount of input features. The importance of individual genes was quantified applying the Mean Decrease Gini index. In the SVM-RFE pipeline, the algorithm was built on a support vector machine with a linear kernel, and recursive elimination of features with relatively low contribution was performed through 5-fold stratified cross-validation. The optimal feature subset was determined by comparing cross-validation accuracy across a gradient of feature numbers. In the final step, intersection genes from three aforementioned machine learning approaches were defined as potential core hub genes of WLP in regulating MP to improve R-IRI.

### 2.8 Molecular docking

AutoDock Vina (version 1.5.7) was applied for molecular docking between the blood-entering active components of WLP and potential core hub genes to evaluate their binding activity. The Structure-Data File structure files of small-molecule compounds were downloaded from the PubChem database, and their structures were optimized using ChemDraw 3D, followed by minimization of energy with the MMFF94 force field. The 3D structures of the target proteins were obtained exclusively from the Protein Data Bank (PDB). If no reported crystal structure was available, the protein was directly excluded from the subsequent docking analysis. Preprocessing of proteins and ligands was performed in AutoDock, including adding hydrogen atoms, removing water molecules, setting rotatable bonds of ligands, and defining the coordinate parameters of the docking box. The combining ability between target proteins and ligands was evaluated by docking scores. The structure with the lowest binding energy (The highest score among those with a docking score less than −5) was selected to showcase the optimal binding mode. CB-Dock2 was utilized for visualizing the docking results, while LigPlot + v2.2 and PyMOL 3.1 software were applied to analyze and display the two-dimensional interactions between compounds and key amino acid residues as well as the three-dimensional complex structures, respectively.

### 2.9 Molecular dynamics simulation

In the present study, molecular dynamics simulations were performed using the Desmond module integrated within the Schrodinger software package, aiming to verify the binding stability between core target proteins and blood-entering bioactive components of WLP. Initially, the simulation system was constructed with the OPLS4 molecular force field, followed by solvation using the OPC water model: the protein-small molecule compounds were placed in a rectangular water box, with Cl^−^ ions added to neutralize the system charge. Meanwhile, 0.15 M NaCl was introduced to mimic the osmotic pressure under physiological conditions. The system temperature was set at 310 K and the pressure was maintained at 1 atmosphere (atm). During the simulation process, energy minimization was first performed to eliminate steric hindrance conflicts and unfavorable interactions, followed by a 12-ps NPT equilibration molecular dynamics simulation. The time step was set at 100 ps for all stages of the dynamic simulation, with the total simulation duration reaching 100.102 ns. From the simulation results, the root-mean-square deviation (RMSD) and radius of gyration (Rg) data corresponding to WLP’s blood-entering bioactive components and core target proteins were extracted separately. Based on these data, the binding free energy was calculated using Python software, and the free energy landscape was further constructed accordingly.

### 2.10 *In vivo* experimental verification

#### 2.10.1 Animals

BALB/c mice (6 weeks old, weighing 18–22 g) were supplied by the Animal Center of Xuzhou Medical University. Animals were kept in clear plastic cages under a daily 12 h light cycle, with ambient humidity maintained at 50%–55% and room temperature. All mice were fed sterile water and standard chow by free access way during the acclimation period. This experiment followed the rules of animal experiment procedure formulated by the Experimental Animal Ethics Committee (Ethical approval No. HEYLL202588). The sample size for each group was determined by power analysis using G*Power 3.1 software. With the effect size set to 0.8, significance level (α) = 0.05, and statistical power (1-β) = 0.85, the minimum required sample size was calculated to be 5 per group. To ensure the reliability of the experimental results, we increased the sample size to 6 mice per group, which is consistent with the sample size used in previous studies on R-IRI (Zhou et al., 2024).

#### 2.10.2 Animals model construction and interventions

After 1 week of adaptive feeding, mice were randomly divided into seven groups (n = 6) using a random number table, including sham-operated group (Sham-G), model group (MG), positive control group (PG), low-dose group of WLP (WLP-L), high-dose group of WLP (WLP-H), low-dose group of 3,4−Dihydroxybenzaldehyde (API-L), and high-dose group of 3,4−Dihydroxybenzaldehyde (API-H). All animal interventions, sample collection, histological processing, and quantitative analysis were performed by independent researchers in a blinded manner to avoid selection and measurement bias. Mice in MG, PG, WLP-L, WLP-H, API-L and API-H groups were anesthetized with 2% isoflurane + oxygen (0.5 L/h). Core body temperature was maintained at approximately 37 °C by means of an electrically heated plate. Clip the hair on both bilateral dorsal regions, then make two renal incisions to expose the renal hilum. Then, use non-invasive vascular clamps to clamp bilateral renal pedicles for 30 min. After reperfusion, the peritoneum was sutured 4/0 and 0.5 mL sterile normal saline was injected intraperitoneally. Mice in Sham-G underwent identical surgical incisions with bilateral renal mobilization, but without renal artery clamping, followed by wound closure at the same time point. Following the aforementioned interventions, mice in different groups were subjected to intragastric administration for 14 consecutive days. The dosing regimen for intragastric administration was shown in Table 1. Among them, the dosages of WLP-L and WLP-H were determined based on the dose conversion between humans and mice by the body surface area method. Meanwhile, the dosages of API-L and API-H were calculated by the content of 3,4-Dihydroxybenzaldehyde in WLP were converted according to the dosages of WLP-L and WLP-H. The dose of the PG group was determined based on the dose reported in the literature (Li et al., 2020). Following a 24-h fasting period at the last intervention, the mice were euthanized under ether anesthesia, and plasma and renal tissue samples were harvested for subsequent experiment analysis.

**TABLE 1:** Experimental grouping and administration dosage *in vivo* experiment.

Group	Treatment details
Sham-G	An equal amount of normal saline
MG	An equal amount of normal saline
WLP-L	WLP extract at 2.14 g/kg/d
WLP-H	WLP extract at 8.56 g/kg/d
PG	Dexamethasone at 5 mg/kg
API-L	3,4−Dihydroxybenzaldehyde at 10 mg/kg
API-H	3,4−Dihydroxybenzaldehyde at 20 mg/kg

#### 2.10.3 Determination of mice serum biochemistry, renal histopathology, and calculation of renal fibrosis-related indicators

The levels of BUN, CR, TNF-α and IL-1β were determined by applying appropriate detection kits, following the manufacturer’s instructions. Renal tissue sections were stained with H&E and Masson according to the manufacturer’s standard procedures and previous publications (Wang YN. et al., 2024; Wang et al., 2025; Cao et al., 2022). Renal histological data were obtained by three independent observers in a blinded fashion. The degree of renal tissue injury was evaluated and scored (Song et al., 2024; Yang et al., 2023), and the level of renal tissue fibrosis was observed according to the area percentage of fibrosis positive staining.

#### 2.10.4 Immunofluorescence assay

Immunofluorescence analysis was performed as per a previous method (Wang et al., 2023b; Miao et al., 2023; Li XJ. et al., 2024). The sections were placed in 1.5 mL centrifuge tubes, and 1 mL of 3% H_2_O_2_ solution was added to cover the tissues first. The tubes were incubated at room temperature for 1 h in the dark to block endogenous peroxidase activity, followed by washing three times with wash buffer on a multi-functional shaker for 10 min per wash. Subsequently, 1 mL of serum blocking buffer was added, and the samples were incubated with shaking on the same shaker for 1 h to complete serum blocking. After discarding the blocking buffer, 1 mL of primary antibody diluted in serum blocking buffer (prepared at the predetermined ratio) was added, and the mixture was incubated with shaking at 4 °C for 24 h. The primary antibody was discarded, and the sections were washed three times with wash buffer under the aforementioned conditions. Thereafter, 1 mL of horseradish peroxidase-conjugated secondary antibody matching the species of the primary antibody was added, and incubation was performed with shaking at 4 °C for 24 h. The secondary antibody was discarded, and the sections were washed three times with wash buffer using the same method. The centrifuge tubes were then transferred to phosphate-buffered saline (pH 7.4) and washed three times on a decolorizing shaker for 10 min per wash. Tyramide signal amplification reagent was added, and the samples were incubated at room temperature for 10 min in the dark. After incubation, the sections were washed three times with Tris-buffered saline with Tween-20 on a decolorizing shaker for 10 min per wash. Next, 1 mL of ready-to-use 4′,6-diamidino-2-phenylindole was added to the centrifuge tubes, and incubation was carried out at room temperature for approximately 2 h in the dark, followed by three washes with wash buffer on a multi-functional shaker for 10 min per wash. Finally, blank glass slides were labeled with sample numbers, and anti-fluorescence quenching mounting medium was added dropwise. The sections were gently picked up with a fine writing brush, spread flat on the glass slides, and coverslipped. It was ensured that the sections were flat without curling or air bubbles. Samples were finally observed under a light fluorescence microscope, and the acquired images were analyzed with CaseViewer and ImageJ software.

#### 2.10.5 Multivariate statistical analysis

The core protein expression data, MP indicators and R-IRI’s pathological parameters of each group were first standardized by comparing with those of MG. Cluster analysis was performed on the core protein expression data, MP indicators and R-IRI’s pathologicalclinical parameters of each group. At the same time, the correlation between the parameters was systematically analyzed using Pearson correlation analysis.

### 2.11 Statistical analysis

GraphPad Prism version 9.5.1 (GraphPad Software, San Diego, CA, USA) and SPSS version 19.0 (IBM Corporation, Chicago, IL, USA) were applied to analyze the experimental data. Data are presented as mean ± standard deviation, accompanied by 95% confidence intervals (95% CI). Intergroup differences were analyzed using one-way ANOVA, followed by Tukey’s *post hoc* test for multiple comparisons. Exact P-values were reported for all statistical tests. The correlation was analyzed by multivariate statistical analysis, and *P* < 0.05 was deemed statistically significant.

## 3 Results

### 3.1 Acquisition of blood-entering components from WLP and its related targets

**FIGURE 2:**
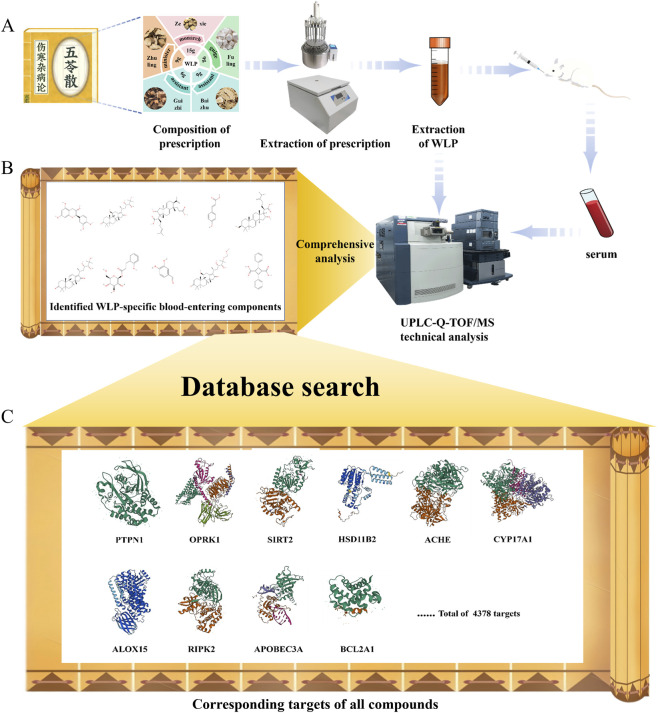
Acquisition of WLP blood-entering components and their targets. **(A)** Flowchart for the acquisition of WLP blood-entering components. **(B)** Structures of WLP blood-entering components in the blood. **(C)** Structures of the targets corresponding to WLP blood-entering components.

The extraction process of WLP was illustrated in (Figure 2A). The components of the WLP extract and active substances in the serum of mice with and without WLP extract intervention were determined separately using UPLC-QTOF-MS/MS. Comparative analysis was conducted to identify the blood-entering components of WLP. As a result, a total of 10 blood-entering components of WLP were identified (Supplementary Table S1), and their chemical structures were shown in (Figure 2B).

The 10 identified blood-entering chemical active components of WLP were chosen as the investigation subjects. The chemical structure files (in SDF format) and corresponding SMILES codes for each component were acquired from the PubChem database (https://pubchem.ncbi.nlm.nih.gov/). Subsequently, potential targets of the active components were predicted using TargetNet (http://targetnet.scbdd.com/), SEA (https://sea.bkslab.org/), SwissTargetPrediction (https://www.swisstargetprediction.ch/), and the Comparative Toxicogenomics Database to improve the comprehensiveness and reliability of target coverage (CTD, https://ctdbase.org/).

The target information acquired from all the aforementioned databases was integrated, with duplicate entries and unannotated targets removed. Subsequently, the refined set of targets underwent standardized annotation via the UniProt database (https://www.uniprot.org), and all target names were unified into official gene symbols, ultimately resulting in 4,378 unique targets (Figure 2C; Supplementary Table S2). These targets were considered potential therapeutic targets for WLP and lay the foundation for further research into the mechanism of WLP in treating R-IRI.

### 3.2 Identification of characteristic targets of R-IRI

**FIGURE 3:**
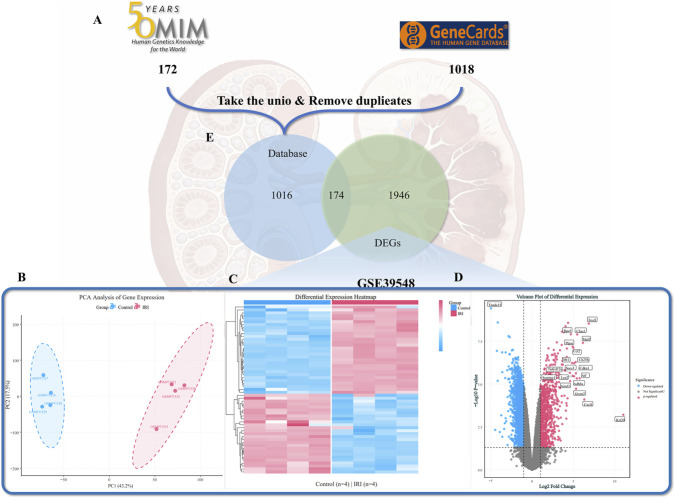
**(A)** Gene targets of R-IRI from OMIM and GeneCards. **(B)** PCA of gene expression. **(C)** Heatmap of DEGs. **(D)** Volcano plot of DEGs. **(E)** Intersection of R-IRI signature genes.

R-IRI related targets were systematically screened from multiple authoritative biological databases. A total of 1,018 disease-associated targets were searched from the GeneCards (https://www.genecards.org/) database (Supplementary Table S3), and 172 targets were obtained from the OMIM (https://www.omim.org/) database (Figure 3A) (Supplementary Table S4). After data integration and deduplication, 1,190 unique targets were identified (Supplementary Table S5). Subsequently, gene expression profile data were extracted from the GEO (https://www.ncbi.nlm.nih.gov/geo/) database using the search term “Renal ischemia-reperfusion injury”. The dataset GSE39548, which contained 2,120 DEGs, was selected for further analysis (Supplementary Table S6). PCA analysis revealed a clear separation of the two groups in their gene expression profiles, confirming the presence of global gene expression differences between them (Figure 3B). The differential expression heatmap further characterized the expression patterns of discrepant genes, with a large number of genes exhibiting opposite expression trends between the two groups (Figure 3C). Volcano plot analysis precisely identified the significantly differentially expressed genes, which consisted of 119 upregulated and 55 downregulated genes in total (Figure 3D). The intersection of database-derived targets and DEGs yielded 174 relevant targets for subsequent research (Supplementary Table S7; Figure 3E).

### 3.3 Identification of characteristic targets of MP

**FIGURE 4:**
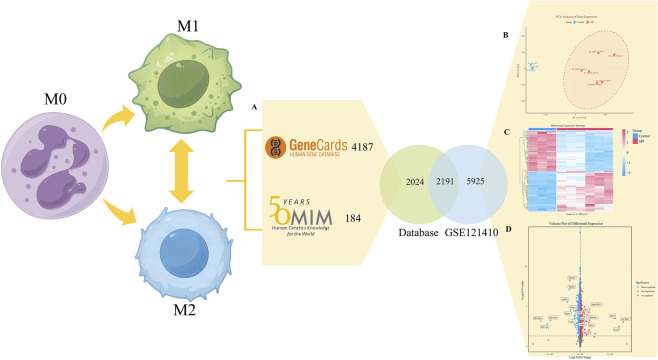
**(A)** Gene targets of MP from OMIM and GeneCards **(B)** PCA of gene expression, **(C)** Heatmap of DEGs, **(D)** Volcano plot of DEGs. **(E)** Intersection of MP-related genes.

We collect MP-related targets from multiple biological databases. The GeneCards (https://www.genecards.org/) and OMIM (https://www.omim.org/) databases provided 4,187 and 184 disease-related targets (Figure 4A; Supplementary Table S8; Supplementary Table S9), respectively, whose union set was subsequently acquired (Supplementary Table S10). Gene expression profile data related to MP was retrieved from the GEO (https://www.ncbi.nlm.nih.gov/geo/) database of the National Center for Biotechnology Information (NCBI). Screening with the keyword “Macrophage polarization” yielded the GSE121410 dataset, generating 8,116 differentially expressed genes (Supplementary Table S11). PCA showed a clear separation between the Control group and MP group samples, reflecting a prominent discrepancy in gene expression patterns between the two groups (Figure 4B). The analytical results were visualized via the heatmaps, and the volcano plot of these differentially expressed genes are showed in (Figures 4C,D). The volcano plot intuitively illustrated the distribution of up- and downregulated genes, with 1,332 genes found to be significantly upregulated and 859 genes significantly downregulated. The intersection of genes from the aforementioned databases and these DEGs resulted in 2,191 targets (Figure 4E; Supplementary Table S12).

### 3.4 Identification of potential genes for WLP in alleviating R-IRI by regulating MP

**FIGURE 5:**
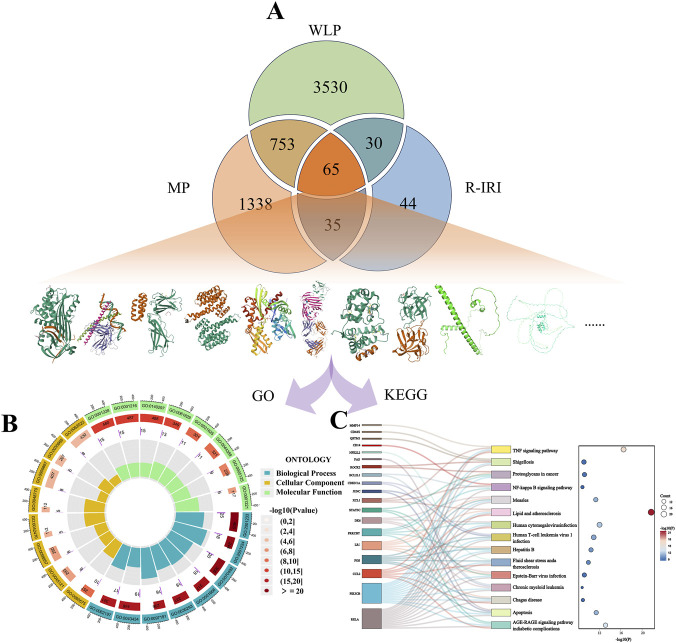
GO and KEGG enrichment analysis. **(A)** Venn diagram of overlapping targets among WLP, MP and R-IRI, and their visualization of corresponding target protein structures. **(B)** Plot of GO functional enrichment analysis. **(C)** KEGG pathway enrichment analysis, integrating chord diagram and bubble plot.

Distinct characteristic targets associated with MP, WLP and R-IRI were identified from divergent perspectives via the aforementioned strategies. There was no doubt that the intersection of these gene datasets represents the potential target genes for WLP in regulating MP to alleviate R-IRI. As shown in (Figure 5A) and (Supplementary Table S13), their intersection yielded a total of 65 common genes, which were considered the potential genes for WLP in regulating MP to alleviate R-IRI.

### 3.5 GO and KEGG enrichment analysis

Functional enrichment analysis of these 65 candidate genes in Section 3.4 was performed using the Metascape platform, with results ranked by *P*-value. For GO enrichment, we used the circlize package to generate a circos plot, which displays the top 8 significantly enriched functional terms (Figure 5B). In the biological process category of GO enrichment analysis, 3,167 significantly enriched terms were identified, with “positive regulation of cell migration” (GO:00140297) being the most prominent. Core modules included “response to wounding” (GO:0001228), “leukocyte migration” and “chemotaxis” (GO:00061629) (Supplementary Table S14). These findings collectively demonstrate that WLP may exert its protective effects against R-IRI by modulating inflammatory responses and macrophage motility programs. Within the MF category of GO enrichment analysis, 298 significantly enriched terms were identified, with GO:0005125 (“cytokine activity”) emerging as the most prominent. These MF terms are centered on kinase-associated enzymatic activities, encompassing functions such as phosphotransferase activity (alcohol group as acceptor; GO:0016773) and protein kinase activity (GO:0004672). Collectively, these findings imply that the candidate genes modulate signal transduction cascades via enzymatic reactions (Supplementary Table S15). In the cellular component category of GO enrichment analysis, a total of 180 significantly enriched terms were identified, with the strongest enrichment observed for phosphatidylinositol 3-kinase complex subtypes (GO:0097191). This finding indicates that these genes are localized to signaling scaffolds that underpin phosphatidylinositol 3-kinase -mediated signaling cascades (Supplementary Table S16).

In KEGG pathway enrichment analysis, a total of 223 pathways were identified (Supplementary Table S17), and a Sankey diagram (Figure 5C) was constructed using the ggsankeyfier package to visualize the 15 most significantly enriched top-ranked pathways. Key pathways included the AGE-RAGE signaling pathway in diabetic complications, *proteoglycans in cancer*, and the NF-κB signaling pathway, all functionally linked to pathological stress responses and inflammatory signaling networks, aligning with the inflammatory pathogenesis of R-IRI. Thus, these findings collectively highlight the enrichment of WLP’s core regulatory targets in inflammation and pathological stress response-related signaling pathways, which further validates the critical role of modulating inflammatory networks in the therapeutic effects of WLP against R-IRI.

### 3.6 Identification of potential core hub genes via machine learning for WLP in regulating MP to alleviate R-IRI

**FIGURE 6:**
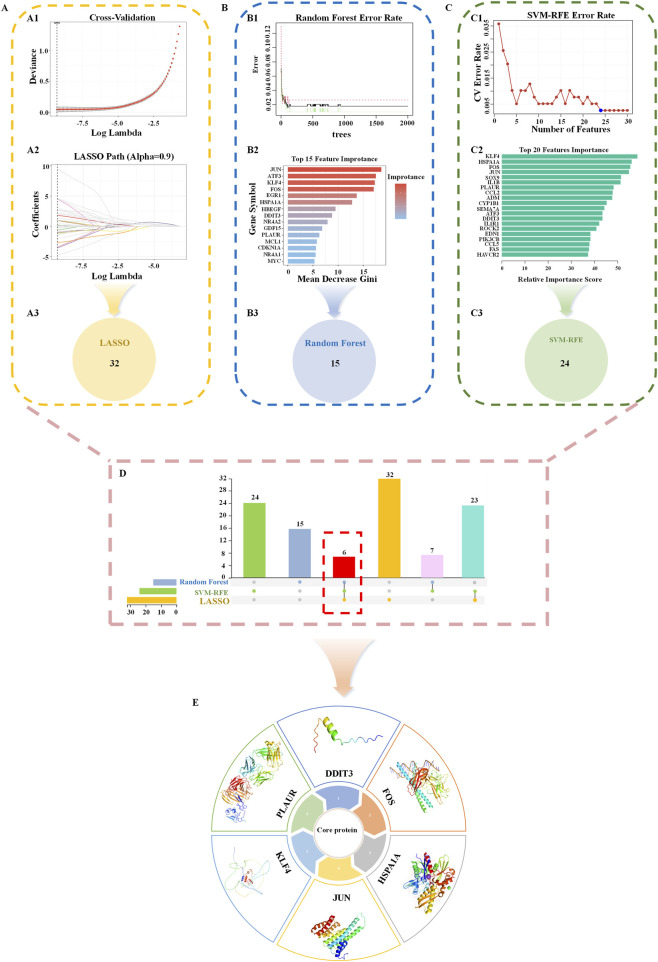
Machine learning screens core targets. **(A)** LASSO regression-based feature screening: **(A1)** Cross-validation curve for determining the optimal regularization parameter; **(A2)** Coefficient profile plot showing feature coefficient changes with λ values; **(A3)** Visualization of 32 features selected by LASSO regression. **(B)** Random Forest-based feature screening: **(B1)** Error rate curve for optimizing the number of decision trees; **(B2)** Feature importance ranking to identify high-contribution targets; **(B3)** Visualization of 15 features selected by Random Forest. **(C)** SVM-RFE-based feature screening: **(C1)** Error rate curve for determining the optimal feature subset size; **(C2)** Feature importance ranking based on elimination order; **(C3)** Visualization of 24 features selected by SVM-RFE. **(D)** Comparative bar plot of feature numbers selected by the three algorithms, with the red box highlighting 6 core targets identified by the intersection of all three methods. **(E)** Circular visualization of core target proteins (e.g., PLAUR, DDIT3, JUN, FOS, HSPA1A, KLF4). and their interaction networks.

Based on the identification of 65 candidate genes, this study further applied three independent machine learning algorithms for core gene screening, including LASSO regression, Random Forest and SVM-RFE. A cross-validation curve (Figure 6A1) was generated to determine the optimal penalty coefficient λ. According to the 1-standard error (1-SE) criterion, the model achieved an optimal trade-off between fitting performance and feature parsimony at a log Lambda value of −9.0, corresponding to an actual λ value of approximately 1.23 × 10^−4^. This optimal λ value was then applied to the LASSO coefficient path plot (Figure 6A2), where all genes with non-zero regression coefficients were screened out, leading to the identification of a total of 32 core signature genes (Figure 6A3). A random forest model was constructed to further validate and refine the core gene set initially screened by LASSO regression, with its predictive stability assessed via an error rate convergence curve (Figure 6B1). As illustrated in the curve, the model’s error rate gradually stabilized and persisted at a low level of 0.02 when the number of decision trees reached around 200, thereby confirming the reliability and robustness of the established model. Utilizing this stable random forest model, we quantified and ranked the predictive contribution of each candidate feature gene by employing the mean decrease Gini coefficient. The feature importance bar chart (Figure 6B2) further exhibited a clear gradient distribution in the importance of these genes. Based on the resulting importance ranking, the top 15 genes with the highest contribution scores were ultimately selected as the core signature genes for subsequent analyses (Figure 6B3). The SVM-RFE error rate curve (Figure 6C1) showed that when the number of features increased to 24, the model’s cross-validation error rate reached its global minimum and stabilized, which was identified as the optimal feature subset size. Subsequently, all feature genes were ranked by their relative importance scores, and the top 24 were selected as the final core target genes (Figure 6C3; Supplementary Table S18). The top 20 of these genes were presented in the feature importance bar chart (Figure 6C2). On this basis, cross-analysis was performed on the key genes identified by the three algorithms (Figure 6D). Ultimately, as a result, six shared core genes, including DDIT3, KLF4, JUN, PLAUR, HSPA1A, and FOS, were identified (Figure 6E). These six genes were regarded as the potential core hub genes of WLP regulating MP to alleviate R-IRI. To further verify the robustness and clinical relevance of these core genes, only one independent external validation cohort (GSE126805) was used for verification, and no additional datasets were included beyond this cohort. Receiver operating characteristic curve analysis was applied to evaluate the ability of DDIT3, KLF4, JUN, PLAUR, HSPA1A, and FOS to distinguish renal ischemia–reperfusion injury (R-IRI) samples from control samples. The results indicated that these genes showed favorable discriminatory performance, supporting the reliability of the core hub genes identified in this study (Supplementary Figure S1).

### 3.7 Core hub genes identification by molecular docking analysis between potential core hub genes and WLP’s blood-entering components

**FIGURE 7:**
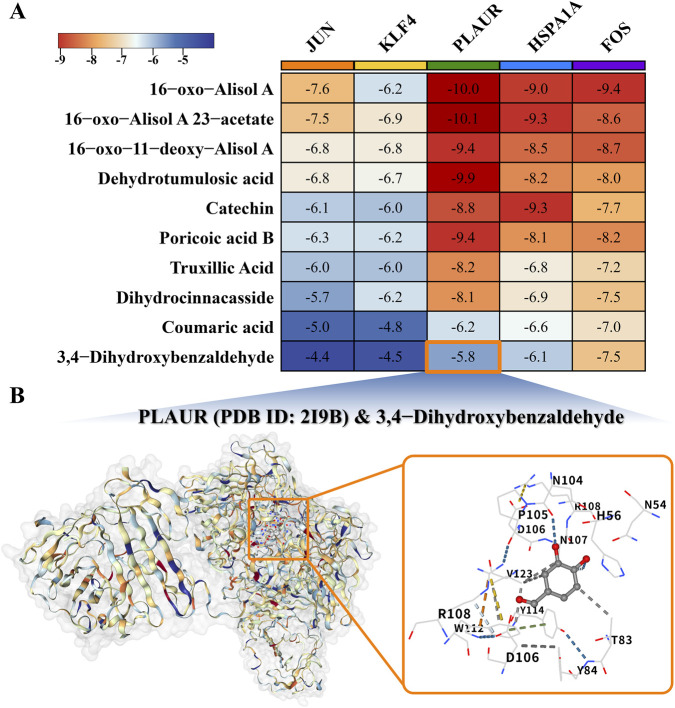
Molecular docking analysis of WLP bioactive components and core targets. **(A)** Heatmap of molecular docking scores between WLP blood-entering components and core targets (JUN, KLF4, PLAUR, HSPA1A, FOS), with 3,4-Dihydroxybenzaldehyde showing favorable binding to PLAUR (score = −5.8 kcal/mol). **(B)** Visualization of PLAUR (PDB ID: 2I9B) binding to 3,4-Dihydroxybenzaldehyde.

To further screen the core hub genes, a systematic study on the molecular docking between the 10 potential core hub genes and WLP’s blood-entering components was conducted. However, in the molecular docking analysis, it was found that DDIT3 has no crystal structure in the PDB. Therefore, DDIT3 was excluded from the core hub gene database due to its inability to undergo molecular docking. The remaining 5 potential core hub genes were subjected to systematic molecular docking analysis. As illustrated in (Figure 7A), among the 5 potential core genes, only PLAUR, HSPA1A, and FOS demonstrated docking scores lower than −5 kcal/mol with all ten WLP’s blood-entering components. These data indicated that only PLAUR, HSPA1A, and FOS can form firm and stable bonds with ten WLP’s blood-entering components, suggesting that these three targets should be regarded as the core hub genes for WLP in regulating MP to alleviate R-IRI. Analysis of the docking scores between PLAUR, HSPA1A, and FOS with the ten WLP’s blood-entering components revealed that their scores range from −5.8 to −10.1 kcal/mol (Figure 7A). The least negative docking score among all the target-compound pairs that meet the requirements in the docking scoring, −5.8 kcal/mol, was observed in the interaction between 3,4-Dihydroxybenzaldehyde and PLAUR (Figure 7A). This indicates that 3,4-Dihydroxybenzaldehyde–PLAUR shows the weakest binding affinity among all identified complexes. Since this complex still binds stably as verified by molecular dynamics simulation, it is reasonable to infer that all other component–target combinations with more negative (stronger) docking scores can bind more stably. Thus, to verify that PLAUR, HSPA1A, and FOS were core hubs, 3,4-Dihydroxybenzaldehyde-PLAUR was selected for detailed analysis. Detailed visualization of the 3,4-Dihydroxybenzaldehyde-PLAUR complex (Figure 7B) revealed stable binding within the active pocket, where the ligand formed hydrogen bonds with residues N107 (Asn107) and Y114 (Tyr114) as well as hydrophobic interactions with V123 (Val123), W112 (Trp112) and R108 (Arg108) (Liébecq, 1992; Dayhoff, 1972). This stable binding mode of the pair with the weakest yet still significant affinity in the high-confidence docking subset fully verifies the credibility of our docking pipeline, thereby enabling reliable inference of robust binding for other ligand-protein pairs with more favorable (more negative) docking scores.

### 3.8 Molecular dynamics simulation from PLAUR and 3,4−Dihydroxybenzaldehyde

**FIGURE 8:**
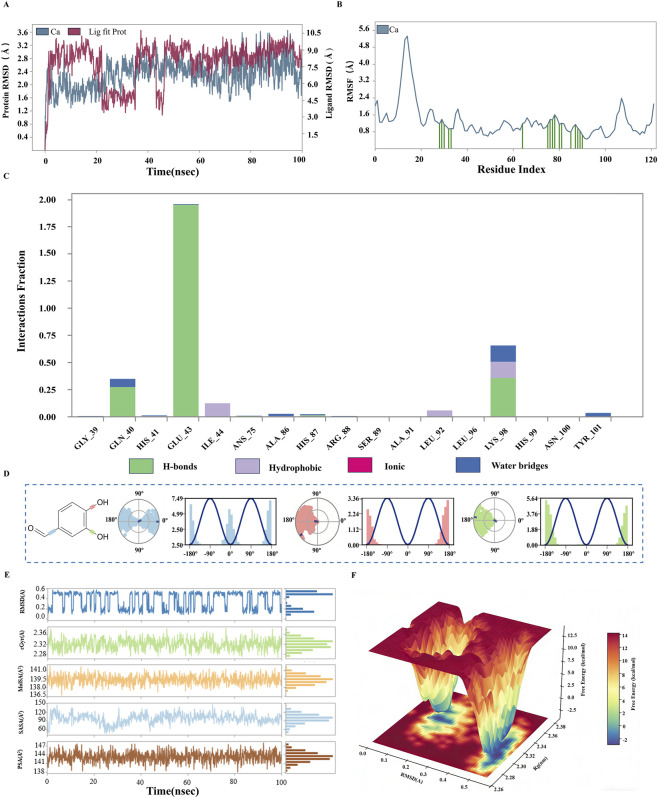
Structural dynamics and interaction profile of the PLAUR-3,4-Dihydroxybenzaldehyde complex from 100 ns molecular dynamics simulations. **(A)** RMSD of PLAUR-3,4-Dihydroxybenzaldehyde over simulation time. **(B)** RMSF of PLAUR-3,4-Dihydroxybenzaldehyde. **(C)** Protein-Ligand contacts between 3,4-Dihydroxybenzaldehyde and PLAUR residues. **(D)** Ligand Torsion Profile. **(E)** Ligand Properties. **(F)** FEL of the complex.

To elucidate the binding mechanism and long-term conformational stability of the PLAUR-3,4-dihydroxybenzaldehyde complex, we further performed 100-ns all-atom molecular dynamics simulations, employing multi-dimensional structural and energetic analyses to characterize the dynamic behavior of the complex. The two-axis plot of Figure 8A illustrated the root-mean-square deviation (RMSD) values for Cα atoms of PAUR (blue curve, left ordinate, unit: Å) and the ligand relative to its cognate protein (red curve, right ordinate, unit: Å) across a 100 ns molecular dynamics simulation. In the initial 20 ns structural relaxation phase, the Cα RMSD increases rapidly from ∼1.0 Å to ∼2.0 Å, after which the complex reaches dynamic equilibrium. For the remainder of the simulation, the Cα RMSD fluctuates stably within the range of 1.5–2.5 Å, indicating that the overall conformation of PLAUR is well stabilized. The ligand RMSD exhibits greater variability, with a notable dip occurring between 20 and 40 ns, which reflects dynamic reorientation of the ligand while remaining within the binding pocket, highlighting its conformational flexibility in the bound state. The root-mean-square fluctuation (RMSF) profile in Figure 8B further delineated the local conformational dynamics along the residue chain: most residues exhibited low flexibility, with RMSF values below 2.4 Å, consistent with the rigidity of secondary structural elements. A prominent RMSF peak reaching approximately 4.8 Å was observed in Figure 8B, which overlapped with the ligand-interacting residues marked by green bars, indicating a flexible region that coincides with the ligand-binding interface. This pattern reflects a typical protein dynamic profile, characterized by stable structured domains paired with flexible ligand-binding loop regions. The bar chart in Figure 8C quantified the interaction scores of individual protein residues with the ligand, revealing residue-specific preferences in the stabilization of the PLAUR-3,4-dihydroxybenzaldehyde complex. Among these, GLU_43 exhibited the highest overall interaction score, approaching 2.0, which was predominantly driven by hydrophobic contacts and water-bridging interactions. Residues such as LYS_98 showed notable contributions from hydrogen bonding, while most other interacting residues displayed a mixed interaction profile involving both water bridges and hydrophobic contacts. These findings collectively demonstrated that the protein-ligand binding is stabilized by a synergistic combination of hydrophobic interactions, hydrogen bonds, and water-mediated bridges, with distinct interaction type preferences across different residues. The dihedral angle dynamics of the hydroxyphenyl and carboxyl functional moieties of the ligand, as shown in Figure 8D, were directly correlated with the protein-ligand interaction profile. The hydroxyphenyl moiety exhibited constrained dihedral angles centered at ±180° and 0°, with a peak probability density of approximately 7.49, reflecting its relatively rigid conformation. The third functional segment displayed a trimodal distribution centered at ±180° and 0°, with a peak density of ∼5.64, indicating a balance between flexibility and restricted motion. These distinct dynamic behaviors underpin the ligand’s binding mode: rigid moieties enhance hydrophobic stabilization, while flexible segments drive specific non-covalent interactions, consistent with the synergistic binding forces identified in the preceding residue interaction analysis. Figure 8E depicted the time evolution of key structural and energetic properties of the PLAUR-3,4-dihydroxybenzaldehyde complex over the 100-ns simulation, shedding light on its conformational stability and dynamic behavior: the RMSD remained stably around 0.4 Å, indicating consistent maintenance of the protein’s conformational integrity throughout the simulation. Meanwhile, the radius of gyration (Rg) stayed at approximately 2.40 Å with fluctuations of less than 0.1 Å, confirming the absence of significant global folding or unfolding events. Furthermore, molecular accessible surface area exhibited consistent fluctuations between 138 and 142.5 Å^2^, suggesting that the solvent exposure level of the target molecular region remained largely unchanged. And, the solvent accessible surface area showed moderate variability ranging from 0 to 60 Å^2^ and displayed a slight downward trend in the later stage of the simulation, implying a minor conformational compaction. Based on this, the polar surface area maintained stable fluctuations between 144 and 153 Å^2^ with persistently high values, indicating that the polar domains of the molecule retained sufficient and continuous solvent exposure, a critical property that mediates the solubility and intermolecular interaction behavior of the system. To further characterize the conformational landscape and thermodynamic stability of the system, we constructed the three-dimensional free energy landscape shown in Figure 8F. A dominant low free-energy basin was concentrated at a RMSD of ≈0.2 Å and a radius of gyration of ≈2.40 nm, representing a thermodynamically favorable conformational state. The surrounding regions exhibited higher free energy (up to 12.5 kcal/mol), indicating restricted flexibility. This narrow distribution of low energy confirms that the system maintained a stable, compact conformation with minimal structural fluctuations.

### 3.9 Effects of WLP on R-IRI, MP, and expression of core target proteins

**FIGURE 9:**
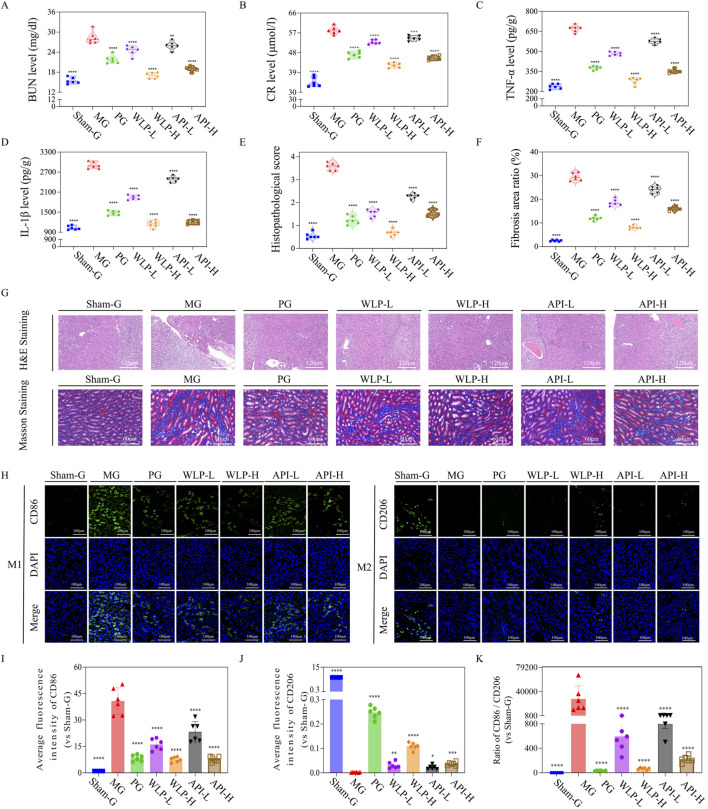
**(A)** Concentration of BUN. **(B)** Concentration of R **(C)** Concentration of TNF-α. **(D)** Concentration of IL-1β. **(E)** Renal Histopathological Score. **(F)** Percentage of the Masson positive area (%). **(G)** Representative images of H&E and Masson staining in renal tissues. **(H)** Immunofluorescence staining of M1 macrophage marker CD86 and M2 macrophage marker CD206 in renal tissues. **(I)** Average fluorescence intensity of CD86 (vs. Sham-G). **(J)** Average fluorescence intensity of CD206 (vs. Sham-G). **(K)** Ratio of CD86/CD206 fluorescence intensity (vs. Sham-G). (Measurement data were expressed as mean ± SD, *n* = 6 per group. *P < 0.05, **P < 0.01, and ***P < 0.001 ****P < 0.0001 vs MG group).

Serum biochemical assays of (Figures 9A,B) revealed that BUN and CR concentrations were maintained at normal baseline levels in the Sham-G, Compared with the Sham-G group, the MG group exhibited significantly increased levels of both BUN (mean difference = −12.90, 95% CI = −15.15 to −10.65, *P* < 0.0001) and Cr (mean difference = −23.59, 95% CI = −26.13 to −21.05, *P* < 0.0001). Compared with the MG group, intervention with PG, WLP-L, WLP-H, API-L, and API-H significantly reduced BUN and Cr levels. For BUN, the mean differences and 95% CIs were as follows: PG (mean difference = 6.543, 95% CI = 4.293 to 8.792, *P* < 0.0001), WLP-L (mean difference = 3.859, 95% CI = 1.610 to 6.109, *P* = 0.0001), WLP-H (mean difference = 11.35, 95% CI = 9.105 to 13.60, *P* < 0.0001), API-L (mean difference = 2.468, 95% CI = 0.2183 to 4.718, *P* = 0.05), and API-H (mean difference = 9.388, 95% CI = 7.139 to 11.64, *P* < 0.0001). For Cr, the mean differences and 95% CIs were: PG (mean difference = 11.08, 95% CI = 8.543 to 13.62, *P* < 0.0001), WLP-L (mean difference = 5.393, 95% CI = 2.852 to 7.934, *P* < 0.0001), WLP-H (mean difference = 15.85, 95% CI = 13.31 to 18.39, *P* < 0.0001), API-L (mean difference = 3.591, 95% CI = 1.050 to 6.132, *P* = 0.01), and API-H (mean difference = 12.60, 95% CI = 10.06 to 15.14, *P* < 0.0001). Notably, WLP exerted a dose-dependent effect: WLP-H normalized BUN (BUN: mean difference = 7.495, 95% CI = 5.245 to 9.745, *P* < 0.0001; Cr: mean difference = 10.46, 95% CI = 7.915 to 13.00, *P* < 0.0001). WLP-H also showed superior regulatory efficacy compared with PG (BUN: mean difference = 4.812, 95% CI = 2.562 to 7.062, *P* < 0.0001; Cr: mean difference = 4.765, 95% CI = 2.224 to 7.305, *P* < 0.0001) and API-H (Cr: mean difference = −3.246, 95% CI = −5.787 to −0.7054, *P* = 0.01), confirming WLP effectively repairs impaired glomerular filtration function. Serum inflammatory factor detection (Figures 9C,D) revealed that TNF-α and IL-1β were maintained at low baseline levels in Sham-G. Compared with the Sham-G group, the MG group exhibited markedly upregulated levels of both TNF-α (mean difference = −1874, 95% CI = −2020 to −1728, *P* < 0.0001) and IL-1β (mean difference = −435.2, 95% CI = −471.0 to −399.5, *P* < 0.0001), reflecting activated local and systemic inflammatory responses post-injury. All intervention groups significantly downregulated TNF-α and IL-1β compared with MG (*P* < 0.0001). WLP exhibited a dose-dependent anti-inflammatory effect: (TNF-α: mean difference = 796.9, 95% CI = 650.8 to 942.9, *P* < 0.0001; IL-1β: mean difference = 201.8, 95% CI = 166.0 to 237.5, *P* < 0.0001), and showed superior efficacy to PG (TNF-α: mean difference = 329.0, 95% CI = 183.0 to 475.0, *P* < 0.0001; IL-1β: mean difference = 96.11, 95% CI = 60.34 to 131.9, *P* < 0.0001) and API-H (IL-1β: mean difference = −71.54, 95% CI = −107.3 to −35.76, *P* < 0.0001), restoring factor levels to near Sham-G baseline. These results indicated that WLP and 3,4-dihydroxybenzaldehyde were associated with suppressed inflammatory cascades and mitigated inflammation-mediated tubular epithelial injury in the setting of R-IRI.

Histopathological scoring (Figure 9E) confirmed a significantly higher score in MG than Sham-G (mean difference = −27.04, 95% CI = −29.05 to −25.02, *P* < 0.0001), while WLP and 3,4-dihydroxybenzaldehyde, indicating enhanced tubular repair and anti-inflammation. Fibrosis quantification (Figure 9F) showed a marked increase in fibrotic area ratio in MG (mean difference = −3.033, 95% CI = −3.300 to −2.766, *P* < 0.0001), which was dose-dependently reduced by WLP and 3,4-dihydroxybenzaldehyde. H&E and Masson’s trichrome staining (Figure 9G) showed intact tubules and no inflammation in Sham-G, whereas MG presented tubular epithelial necrosis, brush border loss, luminal dilation and inflammatory infiltration. PG and API-H only partially alleviated swelling and inflammation with persistent structural disorder. Meanwhile, WLP-L preserved tubular morphology and reduced inflammation. And, WLP-H restored tubular structure to near Sham-G levels. Masson staining also showed extensive collagen deposition in MG, with WLP-H showing no significant difference from Sham-G (*P* > 0.05 vs. Sham-G) and significantly lower fibrosis scores than MG, PG, and API-H (MG vs. WLP-H: mean difference = 2.900, 95% CI = 2.633 to 3.167, *P* < 0.001; PG vs. WLP-H: mean difference = 0.5500, 95% CI = 0.2829 to 0.8171, *P* < 0.001; API-H vs. WLP-H: mean difference = −0.8500, 95% CI = −1.117 to −0.5829, *P* < 0.001).

Immunofluorescence staining (Figure 9H) showed minimal CD86^+^ M1 macrophages and abundant CD206^+^ M2 macrophages in Sham-G, while MG exhibited enhanced CD86^+^ signal and weakened CD206^+^ signal, indicating M1-predominant polarization imbalance. Quantitative analysis (Figures 9I–K) further confirmed MG had increased CD86 ^+^ average fluorescence intensity (mean difference = 39.72, 95% CI = 33.60 to 45.80, *P* < 0.0001), decreased CD206 ^+^ average fluorescence intensity (mean difference = −0.9978, 95% CI = −1.017 to −0.9787, *P* < 0.0001) and elevated CD86/CD206 ratio (mean difference = 27,844, 95% CI = 15,284 to 40,400, *P* < 0.0001) compared with the Sham-G group. All interventions reversed these changes (all *P* < 0.01), with WLP showing dose-dependent efficacy: WLP-H outperformed WLP-L and was comparable to PG. API also exhibited dose-dependent regulation, suggesting it is a key active component of WLP. Thus, WLP corrected inflammatory imbalance by inhibiting M1 and promoting M2 polarization to alleviate renal injury. These results demonstrated that WLP and its main active compound, 3,4-dihydroxybenzaldehyde, were associated with modulated MP and improved R-IRI.

### 3.10 Immunofluorescence evaluation of PLAUR protein expression

**FIGURE 10:**
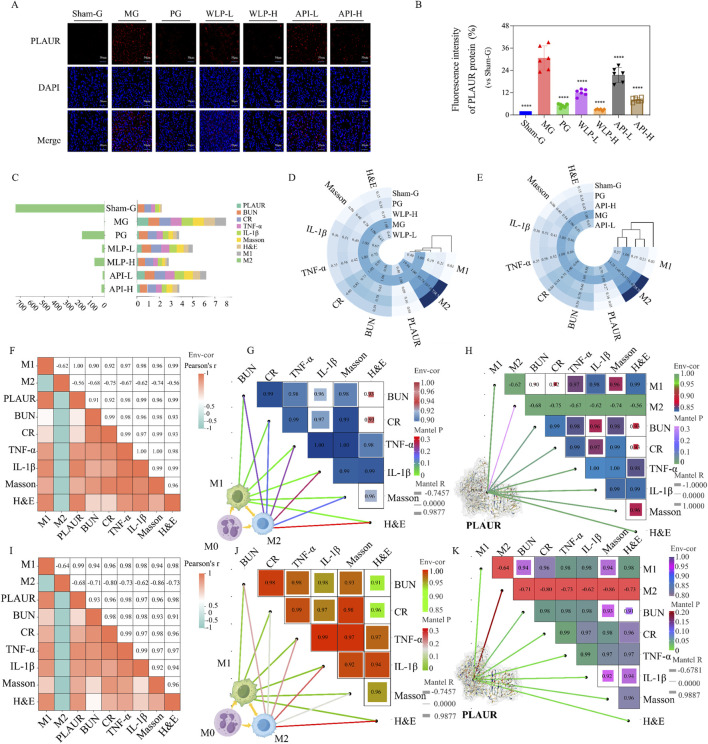
**(A)** Immunofluorescence staining of PLAUR protein in renal tissues from different groups. **(B)** Uantitative analysis and intergroup comparison of PLAUR immunofluorescence intensity in different intervention groups. **(C)** Stacked bar plot of expression levels of PLAUR, M1/M2 MP and inflammatory injury-related markers in renal tissues of each group. **(D)** Regulatory network analysis of PLAUR, M1/M2 MP and inflammatory injury under WLP intervention. **(E)** Regulatory network analysis of PLAUR, M1/M2 MP and inflammatory injury under API intervention. **(F)** Pearson correlation heatmap of PLAUR, M1/M2 MP and inflammatory injury markers in the WLP intervention group. **(G)** Co-expression network and correlation heatmap of M1/M2 MP and inflammatory injury markers in the WLP intervention group. **(H)** Co-expression network and correlation heatmap of PLAUR, M1/M2 MP and inflammatory injury markers in the WLP intervention group. **(I)** Pearson correlation heatmap of PLAUR, M1/M2 MP and inflammatory injury markers in the API intervention group. **(J)** Co-expression network and correlation heatmap of M1/M2 MP and inflammatory injury markers in the API intervention group. **(K)**. Co-expression network and correlation heatmap of PLAUR, M1/M2 MP and inflammatory injury markers in the API intervention group.

Immunofluorescence staining assays was summarized in (Figures 10A,B). Figures 10A,B revealed that PLAUR fluorescence intensity was markedly higher in the MG than in the Sham-G (mean difference = 29.70, 95% CI = 24.99 to 34.41, *P* < 0.0001). After intervention with WLP-L/WLP-H, and API-L/API-H the PG, the abnormally high expression of PLAUR in the MG group was notably reversed, and regulatory effects of WLP and 3,4-dihydroxybenzaldehyde exhibited a dose-dependent pattern (all *P* < 0.0001 vs. MG). Notably, the PLAUR expression level in WLP-H was the closest to that in the Sham-G group. These data demonstrated that WLP and 3,4-dihydroxybenzaldehyde can significantly inhibit the expression of PLAUR protein.

### 3.11 Multivariate statistical and correlation analysis

To verify that WLP and its main active compound 3,4-dihydroxybenzaldehyde alleviate R-IRI by regulating the expression of related core proteins to improve MP, we conducted a systematic multivariate statistical and correlation analysis. At the same time, in order to ensure the comparability of data for various indicators, the parameters of each evaluation index have been normalized relative to MG. The results of multivariate statistical analysis in Figure 10C indicated that there were significant differences in R-IRI pathological parameters, MP pointers, and core protein expression among different groups. And, they all had similar patterns. Compared with Sham-G, all the indicators of MG had become pathologically and injuriously aggravated. However, WLP and 3,4-dihydroxybenzaldehyde could dose-dependently reverse the abnormal expression of all indicators. The clustering results of Figures 10D,E indicated that not only did the parameters of different groups have similar changing trends, but also their data showed certain clustering patterns. WLP and 3,4-dihydroxybenzaldehyde could dose-dependently reverse the abnormal pathological indicators of MG, gradually improving them to approach those of the Sham-G group. These data (Figures 10C–E) indirectly suggested that there may be correlations in the regulation of MP, R-IRI and core proteins. On this basis, in order to further confirm the correlation between them, we conducted a Pearson correlation analysis of each parameter within a group and a Mantel test. The results of intragroup correlation analysis from (Figures 10F,I) confirmed that the absolute values of the Pearson correlation coefficients of the changes induced by WLP and 3,4-dihydroxybenzaldehyde among each parameter were all above 0.56 indicating a significant correlation among the parameters. Meanwhile, the results of inter-group Mantel test for correlation analysis from Figures 10G,J showed that the absolute values of the correlation coefficients between MP and R-IRI parameters were all greater than 0.91. These data confirmed that the improvement and regulation of WLP and 3,4-dihydroxybenzaldehyde on R-IRI were closely related to the modulation of MP. Furthermore, the results of inter-group Mantel test for correlation analysis from Figures 10H,K further revealed that the absolute values of the correlation coefficients between the MP and R-IRI parameters and the expression of the core protein PLAUR were all greater than 0.56, thereby further confirming that the effect of WLP and 3,4-dihydroxybenzaldehyde on regulating MP to improve R-IRI was closely related to the regulation of the core protein.

## 4 Discussion

TCM has long been used in the clinic and has been considered an important therapy for the prevention and treatment of CKD (Shan et al., 2024; Zhong et al., 2024; Gao et al., 2025; Zhao et al., 2025; Miao et al., 2022). Extensive research has demonstrated that the active components of TCM are an important source of new drugs (Pan et al., 2024; Ajibade et al., 2024; Miao and Vaziri, 2025; Liu et al., 2026; Ben-Azu et al., 2025; Zheng et al., 2025; Mahboubi et al., 2025). WLP is a classic formula of TCM with a long-standing pharmacological history of application for inducing diuresis to resolve dampness and warming yang to activate qi movement (Mou et al., 2022). It has been clinically administered for the treatment of disorders associated with internal retention of dampness and stagnation of Qi activity, including renal insufficiency (Ding et al., 2013). Current research on the renoprotective effects of WLP has predominantly focused on its ability to mitigate oxidative stress and modulate the expression and release of pro-inflammatory mediators (Lu et al., 2024). These mechanisms are consistent with the established pathological hallmarks of R-IRI, including oxidative damage and a dysregulated inflammatory response. And, as a central regulatory node in the pathogenesis and progression of R-IRI (Hu et al., 2021), MP has rarely been reported to be associated with the therapeutic effects of WLP. This gap impedes a comprehensive understanding of the molecular mechanisms underlying WLP’s renoprotective effects, particularly within the conceptual framework of TCM’s holistic regulatory theory. The present study has three novel aspects: we first identify PLAUR, HSPA1A, and FOS as the potential core targets of WLP in regulating MP; we systematically clarify the molecular mechanism by which WLP protects against R-IRI by modulating MP; and we provide a new potential therapeutic strategy for the clinical treatment of R-IRI. Recent studies on R-IRI have demonstrated that MP dysregulation—a pathophysiological state characterized by preferential polarization toward pro-inflammatory M1 macrophages and defective differentiation into anti-inflammatory M2 macrophages—exacerbates renal injury through sustained overproduction of pro-inflammatory cytokines (e.g., TNF-α) and reactive oxygen species (ROS), while concurrently suppressing endogenous renal repair and tissue remodeling processes (Hu et al., 2021; Wang et al., 2022). Although multiple studies have established the pivotal role of MP in the pathogenesis of R-IRI, research examining whether TCM prescriptions confer renoprotection by modulating this regulatory axis remains limited. This gap contrasts with conventional approaches to elucidating the holistic mechanisms of TCM, which typically rely on single-omics analyses or network pharmacology—methodologies often lacking rigorous experimental cross-validation. In the present study, a comprehensive integrative strategy was adopted, which incorporated mining of public GEO datasets, screening of database retrieval from the GeneCards and OMIM databases, as well as identification of blood-entering components of WLP and their corresponding targets. Machine learning algorithms were applied to prioritize core targets. This approach systematically narrowed down the intersection of R-IRI-related genes, MP-related genes and WLP targets, thereby effectively addressing the complexity challenge of “multi-component and multi-target” in TCM research. Compared with previous studies that only identified the targets of WLP via network pharmacology or analysis of a single dataset (Mou et al., 2022), the integration strategy of this study improved the reliability of core target identification, and through molecular docking, molecular dynamics simulation, and *in vivo* experiments, the role of PLAUR as a key mediator has been consistently verified.

The mechanistic findings of the present study supplement and expand the existing understanding of the renoprotective effects of WLP against R-IRI. Previous studies have demonstrated that WLP exerts renal protection by regulating pathways including nuclear factor E2-related factor 2/antioxidant response element and mitogen-activated protein kinase, thereby suppressing oxidative stress and apoptosis (Bondi et al., 2022). However, the present study revealed an additional regulatory mechanism, which WLP modulates the balance of MP by targeting core regulatory factors such as PLAUR, thereby inhibiting the activation of pro-inflammatory M1 macrophages, promoting the differentiation of anti-inflammatory M2 macrophages, reducing renal inflammatory infiltration, and accelerating the repair of renal tissues. Notably, the aforementioned core targets including PLAUR, HSPA1A, and FOS are functionally associated with the NF-κB and PI3K/Akt signaling pathways, both of which are central regulators of macrophage polarization and inflammatory responses in R-IRI. WLP may inhibit the overactivation of the NF-κB pathway, thereby suppressing the transcription of pro-inflammatory cytokines and shifting macrophage polarization toward the anti-inflammatory M2 phenotype. Meanwhile, WLP may activate the PI3K/Akt signaling pathway to promote cell survival and alleviate renal tubular injury and oxidative stress. By coordinately regulating these canonical signaling pathways, WLP further ameliorates inflammatory damage and promotes tissue repair during R-IRI. This is consistent with the findings of Hu et al. (2021), who reported that restoring the M1/M2 macrophage balance can alleviate R-IRI by suppressing inflammatory responses and promoting tissue regeneration (Hu et al., 2021). However, this study was the first to link this regulatory process to the efficacy of WLP, filling the research gap between MP biology and TCM pharmacology in the field of kidney disease treatment. It could furnish a scientific rationale for the pharmacological utilization of WLP in the personalized clinical treatment of R-IRI. On this basis, *in vivo* validation was performed for WLP and its individual bioactive components. In terms of compound selection, this approach does not negate the multi-component synergistic effects of TCM. Instead, it adheres to a research paradigm of progressive focusing. From the blood-entering components of WLP, we prioritized the selection of compounds with molecular docking scores close to −5 kcal/mol. This value indicated that its binding affinity with PLAUR was relatively weak. Components with even lower docking scores may also possess therapeutic potential. Focusing on a single compound can exclude confounding factors associated with the multi-component system, precisely dissect its direct regulatory effects on PLAUR and MP, and simplify the intricate research framework of TCM. Therefore, this approach ensures experimental rigor, establishes a causal relationship among the compound, its target, and its renoprotective activity against R-IRI, and lays the foundation for subsequent investigations into the multi-component synergistic effects of WLP.

From a pharmacological perspective, the present study has built a bridge between the traditional theories of TCM and modern molecular biology. In the pathogenesis of R-IRI, the TCM theory of “dampness stagnation and Qi activity obstruction” can be manifested as renal tissue damage, inflammatory infiltration and water metabolism disorder induced by MP imbalance (analogous to the state of “Dampness stagnation” and “Qi obstruction”) (Li G. et al., 2024; Zhang et al., 2022). This corresponding relationship provides a modern molecular-level interpretation of the traditional efficacy of WLP, further verifying the scientific connotation of the holistic treatment concept of TCM in the field of kidney diseases. Meanwhile, the efficacy of WLP in inducing diuresis to resolve dampness and warming yang to activate Qi movement is reflected by its capacity to restore the balance of MP, suppress inflammatory responses, and promote renal tissue repair (alleviating “Dampness stagnation” via regulating the circulation of “Qi activity”). This corresponding relationship provides a modern molecular interpretation for the traditional efficacy of WLP, further verifying the scientific connotation of the holistic treatment concept of TCM in the field of kidney diseases.

Although valuable findings have been obtained in the setting of the current study, several limitations require consideration. First, although the regulatory effects of 3,4-dihydroxybenzaldehyde on PLAUR and MP have been verified, the synergistic effects of multiple blood-entering components of WLP remain to be fully elucidated. Future studies should design combinatorial experiments to explore the interactions among different components and their integrated regulatory roles in the MP pathway. Second, the assessment of renal function in this study was primarily restricted to serum BUN, creatinine levels, and histopathological scoring. More comprehensive and sensitive functional indicators, including glomerular filtration rate, urine albumin/creatinine ratio, and renal tubular injury markers such as Kidney Injury Molecule-1 and Neutrophil Gelatinase-Associated Lipocalin, were not evaluated in the current work. This limitation may reduce the comprehensiveness of renal function evaluation in the present study. Third, the identification of MP phenotypes mainly relied on CD86 (M1) and CD206 (M2) in the current study. Additional representative phenotypic markers are still required to achieve a more systematic, reliable, and in-depth characterization of MP status. Fourth, the present study demonstrates a close association between modulated MP and renoprotection, but definitive causal evidence has not been established. Alternative renoprotective mechanisms, including anti-inflammatory effects, antioxidant effects, and direct renal epithelial protection, cannot be fully excluded and may act independently or cooperatively. Fifth, the *in vivo* experiments were conducted based on a specific animal model of R-IRI, and the applicability of the study findings to human R-IRI needs to be further validated by large-sample clinical trials. Third, the downstream signaling pathways through which PLAUR, HSPA1A, and FOS modulates MP and renal tissue repair still require in-depth investigation, so as to construct a more comprehensive molecular network underlying the renoprotective effects of WLP against R-IRI.

In summary, this study clarified the specific mechanism by which Wuling Powder attenuates R-IRI by regulating macrophage polarization, providing new evidence for its clinical application. Specifically, WLP exerted its protective action by modulating MP balance through targeted regulation of PLAUR, HSPA1A, and FOS, thereby attenuating inflammatory responses and facilitating renal tissue repair. This integrated research strategy addresses the complexity challenge of the multi-component system of TCM, and the differential *in vivo* experimental design ensures both the depth of mechanistic exploration and clinical relevance. Furthermore, this work establishes a pharmacological link bridging TCM theoretical principles and modern molecular biology, building a firm groundwork for the further exploitation and clinical translation of WLP in the management of R-IRI. Future research focusing on multi-component synergistic effects and clinical translation will further deepen the understanding of the therapeutic prospect of TCM in the management of kidney diseases.

## 5 Conclusion

**FIGURE 11:**
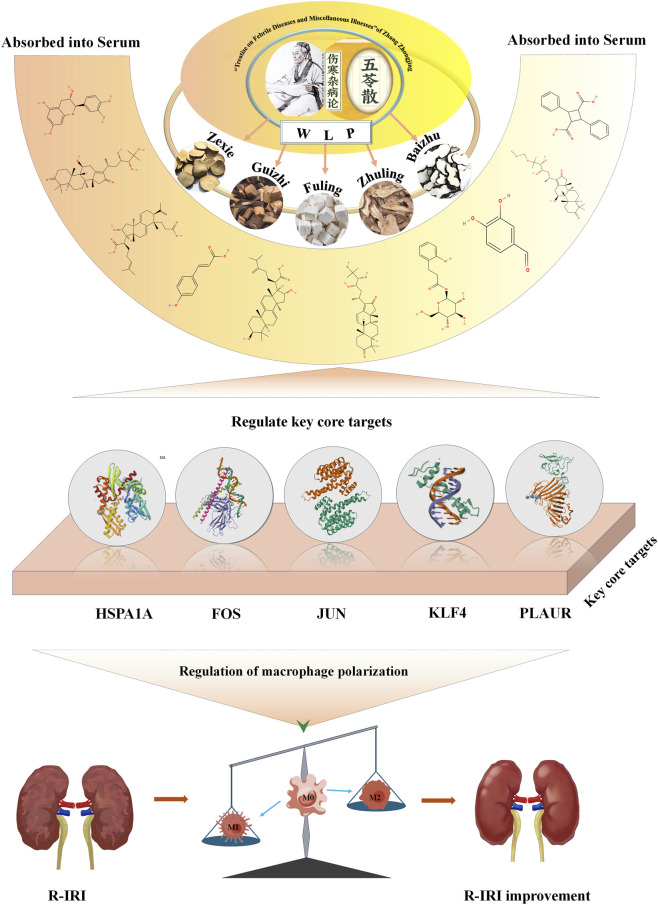
Potential mechanism by which WLP regulates MP to alleviate R-IRI.

This study applied an integrated strategy of computational pharmacology and experimental validation to reveal the mechanism of Wuling Powder in ameliorating R-IRI by regulating macrophage polarization. This strategy fully integrated the advantages of bioinformatics, machine learning, and traditional experimental pharmacology, thus verifying our initial research hypothesis. By targeting the intersection of MP-related proteins, R-IRI-associated proteins, and target proteins of its own bioactive components, WLP modulates the M1/M2 MP balance, thereby alleviating the progression of R-IRI (Figure 11). This study integrated the pharmacological theory of TCM with modern molecular biology. It not only deepened the understanding of the therapeutic mechanism of WLP, but also provided a valuable methodological framework for the mechanistic investigation of other TCM formulae. The findings underscore the prospect of targeting MP as a novel therapeutic avenue for R-IRI, while reinforcing the scientific significance of pharmacological legacies in guiding modern drug development.

Despite the remarkable achievements outlined above, the present study still has inherent limitations that warrant further exploration in subsequent research. First, although PLAUR, HSPA1A, and FOS have been identified as potential core targets, the synergistic effects of multiple blood-entering components of WLP on MP have not been fully elucidated—considering that the therapeutic efficacy of TCM is typically attributed to the synergistic actions of multiple components. Second, the *in vivo* experiments were confined to a specific animal model of R-IRI, and thus validation in other R-IRI models or clinical specimens is required to confirm the generalizability of the study findings to human R-IRI. In addition, definitive causal evidence linking MP to the renoprotective effects of WLP remains to be established. Further studies using genetic strategies (e.g., macrophage-specific knockout or adoptive transfer) are necessary to rigorously verify the causal role of MP in mediating renal protection. Finally, the downstream signaling pathways through which WLP modulates MP still demand in-depth investigation to decipher the complete regulatory network.

Future research should focus on the isolation and identification of key bioactive components in WLP that synergistically regulate MP, as well as the exploration of their interactions with core protein (PLAUR, HSPA1A, and FOS) and related signaling pathways. Meanwhile, clinical trials need to be conducted to verify the correlation between core protein expression levels and the severity of R-IRI in human patients, together with the therapeutic effects of WLP on MP sin clinical settings. In addition, the integration of multi-scale computational systems pharmacology with single-cell sequencing technology could enable more precise and in-depth dissection of the cell-type-specific regulatory mechanisms underlying WLP-mediated modulation of MP.

## Data Availability

The datasets presented in this study can be found in online repositories. The names of the repository/repositories and accession number(s) can be found in the article/Supplementary Material.

## Glossary

AI: Artificial intelligence
AP-1: Transcription Factor Subunit
APL-H: The high-dose 3,4−Dihydroxybenzaldehyde groups
APL-L: The low-dose 3,4−Dihydroxybenzaldehyde groups
DDIT3: DNA Damage Inducible Transcript 3
DEGs: Differentially expressed genes
FOS: Fos Proto-Oncogene
GO: Gene Ontology
HSPA1A: A (Hsp70) Member 1A
IL-1β: Interleukin-1β
JUN: Jun Proto-Oncogene
KEGG: Kyoto Encyclopedia of Genes and Genomes
KLF4: KLF Transcription Factor 4
LASSO: Least Absolute Shrinkage and Selection Operator
MG: Model group
MP: Macrophage Polarization
PG: Positive drug control group
PLAUR: Urokinase Plasminogen Activator Receptor
Rg: Radius of gyration
R-IRI: Renal ischemia-reperfusion injury
RMSD: the root-mean-square deviation
RMSF: root-mean-square fluctuation
ROS: Reactive oxygen species
TVM-RFE: Support vector machine-recursive feature elimination
TCM: Traditional Chinese Medicine
TNF-α: Tumor Necrosis Factor-α
WLP: Wuling powder
WLP-G: Wuling powder high-dose group
WLP-L: Wuling powder low-dose group
