# Supplementary material for: Mechanistic elucidation of Wuling Powder targeting macrophage polarization to ameliorate renal ischemia-reperfusion injury via multidimensional computational systems pharmacology coupled with experimental validation
Source: Front Pharmacol. 2026 Jun 4;17:1811709. doi: 10.3389/fphar.2026.1811709 (PMC13275466; doi:10.3389/fphar.2026.1811709)
Supplement: Supplementary file 1 [file Supplementaryfile1.docx]

**Mechanistic elucidation of Wuling Powder targeting macrophage polarization to ameliorate renal ischemia-reperfusion injury via multidimensional computational systems pharmacology coupled with experimental validation**

Qian-Qian Wang^a^,[[1]](#footnote-1)1, Yu-Fan Gong^a,1^, Zi-Feng Wang^a,1^, Xin Pu^b^, Xiang Li^a^, Lin Bai^a^, Jie Zhang^a^, Juan Xie^a^, Jing Li^a^, Wei Jiang^a^, Li-Min Liu^c^, Jia-Wei Zuo^d,*^, Ying-Yong Zhao^c,e,*^, Dong-Hui Zheng^a,*^, Hai-Lun Li^a^,[[2]](#footnote-2)*

^a^ Department of Nephrology, The Affiliated Huai’an Hospital of Xuzhou Medical University & The Second People’s Hospital of Huai’an, Jiangsu, China

^b^ Department of Nephrology, Jiangdu People’s Hospital Affiliated to Yangzhou University, Jiangsu , China

^c^ Faculty of Life Science & Medicine, Northwest University, Xi’an, Shaanxi, China

^d^ Department of Radiotherapy, The Affiliated Huai’an Hospital of Xuzhou Medical University & The Second People’s Hospital of Huai’an, Jiangsu, China

^e^ School of Pharmaceutical Sciences, Zhejiang Chinese Medical University, Hangzhou, Zhejiang, China

**Table S1 Blood-entering components of WLP**

| NO | Name | NO | Name |
| --- | --- | --- | --- |
| 1 | 3,4-Dihydroxybenzaldehyde | **6** | Coumaric acid |
| 2 | Catechin | **7** | Dehydrotumulosic acid |
| 3 | 16-oxo-Alisol A | **8** | Dihydrocinnacasside |
| 4 | 16-oxo-11-deoxy-Alisol A | **9** | Poricoic acid B |
| 5 | 16-oxo-Alisol A 23-acetate | **10** | Truxillic Acid |

**Table S2 Targets of WLP**

| NO | Target | No | Target | NO | Target | NO | Target |
| --- | --- | --- | --- | --- | --- | --- | --- |
| 1 | SRD5A2 | **1096** | PTPN1 | **2191** | STS | **3285** | AR |
| 2 | HSD11B2 | **1097** | ACE | **2192** | PTPN2 | **3286** | KCNH2 |
| 3 | OPRK1 | **1098** | PTGFR | **2193** | Chrm2 | **3287** | CYP17A1 |
| 4 | Nos2 | **1099** | PPO2 | **2194** | PLA2G1B | **3288** | ache |
| 5 | ALOX15 | **1100** | APOBEC3A | **2195** | CHRM4 | **3289** | DYRK1A |
| 6 | HDAC4 | **1101** | SRD5A1 | **2196** | NR1H4 | **3290** | HSD11B1 |
| 7 | BCL2A1 | **1102** | GPBAR1 | **2197** | NR2F2 | **3291** | PTPN7 |
| 8 | Adra2c | **1103** | PLIN1 | **2198** | CHRM1 | **3292** | RIPK2 |
| 9 | GALR3 | **1104** | CDC25B | **2199** | DNMT1 | **3293** | ALPL |
| 10 | TLR9 | **1105** | CES2 | **2200** | HMGCR | **3294** | CYP19A1 |
| 11 | HSD17B2 | **1106** | PYGL | **2201** | PYGM | **3295** | NR3C1 |
| 12 | F2R | **1107** | PTGS2 | **2202** | PGR | **3296** | NR3C2 |
| 13 | CCR1 | **1108** | PSEN2 | **2203** | SCN9A | **3297** | GABRA2 |
| 14 | MMP1 | **1109** | PER2 | **2204** | MTOR | **3298** | PIK3CA |
| 15 | NLRP3 | **1110** | C5AR1 | **2205** | MAPK14 | **3299** | MAP3K11 |
| 16 | CHUK | **1111** | PDE10A | **2206** | LIMK2 | **3300** | AVPR1A |
| 17 | MTNR1A | **1112** | MTNR1B | **2207** | HPGDS | **3301** | IDH1 |
| 18 | AKT1 | **1113** | JAK3 | **2208** | SMO | **3302** | ABL1 |
| 19 | MAPK1 | **1114** | PDGFRB | **2209** | KIT | **3303** | HTR2A |
| 20 | PLA2G2A | **1115** | CDK2 | **2210** | AURKA | **3304** | FYN |
| 21 | LCK | **1116** | NTRK1 | **2211** | MAP3K9 | **3305** | MAP3K10 |
| 22 | POLA1 | **1117** | CETP | **2212** | HSP90AA1 | **3306** | CDC7 |
| 23 | CSF1R | **1118** | GCK | **2213** | MERTK | **3307** | PRCP |
| 24 | DRD3 | **1119** | MMP3 | **2214** | PIK3CB | **3308** | SMYD2 |
| 25 | APP | **1120** | HTR2C | **2215** | ADA | **3309** | BACE2 |
| 26 | CTSD | **1121** | MDM2 | **2216** | CCND1 | **3310** | CCNE2 |
| 27 | PFKFB3 | **1122** | P2RX7 | **2217** | DUT | **3311** | MAP3K14 |
| 28 | PDE2A | **1123** | GRM5 | **2218** | KCNK3 | **3312** | ADORA2A |
| 29 | P2RX3 | **1124** | CCNB3 | **2219** | TACR1 | **3313** | GRM1 |
| 30 | AKR1C3 | **1125** | CDK1 | **2220** | NPY5R | **3314** | POLB |
| 31 | CRHR1 | **1126** | IGF1R | **2221** | ITK | **3315** | IL6ST |
| 32 | S1PR3 | **1127** | TTK | **2222** | GSK3B | **3316** | F10 |
| 33 | PSENEN | **1128** | GABRB2 | **2223** | CDK4 | **3317** | CCNA1 |
| 34 | NCSTN | **1129** | APH1A | **2224** | PSEN1 | **3318** | APH1B |
| 35 | GABRG2 | **1130** | CCNE1 | **2225** | CCNB1 | **3319** | CCNA2 |
| 36 | CCNB2 | **1131** | HTR1E | **2226** | KCNA3 | **3320** | PLIN5 |
| 37 | PTGER2 | **1132** | PRKCA | **2227** | PRKCD | **3321** | PRKCG |
| 38 | PRKCB | **1133** | PRKCE | **2228** | PRKCH | **3322** | PRKCQ |
| 39 | TRPV4 | **1134** | VAV1 | **2229** | SYK | **3323** | FNTA |
| 40 | ABCB1 | **1135** | PDE4D | **2230** | PGGT1B | **3324** | CALCRL |
| 41 | KDR | **1136** | ROCK2 | **2231** | LIMK1 | **3325** | HCRTR2 |
| 42 | HCRTR1 | **1137** | ALOX5 | **2232** | ROCK1 | **3326** | EGFR |
| 43 | PDGFRA | **1138** | EPHB3 | **2233** | F2RL1 | **3327** | MC4R |
| 44 | MC1R | **1139** | MC5R | **2234** | MC3R | **3328** | PIK3CD |
| 45 | NR1I2 | **1140** | PRKD1 | **2235** | IKBKB | **3329** | EDNRB |
| 46 | CHEK1 | **1141** | REN | **2236** | AMPD2 | **3330** | CTSE |
| 47 | PGA5 | **1142** | JAK1 | **2237** | CDK5 | **3331** | AURKB |
| 48 | AURKAIP1 | **1143** | PGC | **2238** | SLC10A2 | **3332** | ACACB |
| 49 | MAP2K1 | **1144** | BTK | **2239** | S1PR2 | **3333** | FNTB |
| 50 | SIRT2 | **1145** | RORA | **2240** | SHBG | **3334** | FDFT1 |
| 51 | CNR2 | **1146** | SERPINA6 | **2241** | BACE1 | **3335** | NR1H3 |
| 52 | FKBP1A | **1147** | PTGES | **2242** | PARP1 | **3336** | ADAM17 |
| 53 | RORC | **1148** | MAPK3 | **2243** | IDO1 | **3337** | BRD4 |
| 54 | SORD | **1149** | CCNC | **2244** | CDK8 | **3338** | CREBBP |
| 55 | ESR2 | **1150** | SLC6A4 | **2245** | LRRK2 | **3339** | MAPK11 |
| 56 | ADORA3 | **1151** | TYRO3 | **2246** | NPC1L1 | **3340** | JAK2 |
| 57 | CLK4 | **1152** | CLK1 | **2247** | CLK2 | **3341** | DYRK1B |
| 58 | DRD2 | **1153** | PDE4B | **2248** | CD81 | **3342** | FLT4 |
| 59 | SLC6A3 | **1154** | SLC5A2 | **2249** | PAK1 | **3343** | CCKBR |
| 60 | GYS1 | **1155** | SIGMAR1 | **2250** | PDE5A | **3344** | MAPK8 |
| 61 | MAPK10 | **1156** | IRAK4 | **2251** | MAPK9 | **3345** | HDAC8 |
| 62 | PTPRC | **1157** | ESR1 | **2252** | Cnr1 | **3346** | PTK2 |
| 63 | PTPN22 | **1158** | CYP1A2 | **2253** | Rac1 | **3347** | AKR1B1 |
| 64 | THRA | **1159** | RARB | **2254** | THRB | **3348** | RARA |
| 65 | SIRT1 | **1160** | TUBB2B | **2255** | MMP2 | **3349** | ADRA2B |
| 66 | S1PR1 | **1161** | MCL1 | **2256** | CHRFAM7A | **3350** | HCAR2 |
| 67 | CYP11B2 | **1162** | DUSP3 | **2257** | Htr3a | **3351** | Drd4 |
| 68 | PLAT | **1163** | RELA | **2258** | RARG | **3352** | OPRD1 |
| 69 | DRD5 | **1164** | CA5A | **2259** | ICAM1 | **3353** | ampC |
| 70 | PIK3CG | **1165** | DHODH | **2260** | Grin2b | **3354** | XDH |
| 71 | TNF | **1166** | TRPV1 | **2261** | MAOB | **3355** | CTSG |
| 72 | HTR1F | **1167** | CHRM5 | **2262** | ADRA2A | **3356** | Hrh1 |
| 73 | CA12 | **1168** | CA14 | **2263** | PRSS2 | **3357** | Nos1 |
| 74 | NOS3 | **1169** | PRSS1 | **2264** | MAOA | **3358** | PTGS1 |
| 75 | DRD1 | **1170** | APOBEC3G | **2265** | KIF11 | **3359** | CA9 |
| 76 | MIF | **1171** | FOLH1 | **2266** | HDAC1 | **3360** | PLAU |
| 77 | CTDSP1 | **1172** | Htr1b | **2267** | S1PR5 | **3361** | GSK3A |
| 78 | AHR | **1173** | CES1 | **2268** | HDAC3 | **3362** | PIM3 |
| 79 | CACNA1B | **1174** | CYP2C19 | **2269** | HTR5A | **3363** | NR2E3 |
| 80 | Chrm3 | **1175** | SLC6A2 | **2270** | CHEK2 | **3364** | HNF4A |
| 81 | CASP9 | **1176** | TAAR1 | **2271** | Htr6 | **3365** | Chrna4 |
| 82 | RET | **1177** | SELE | **2272** | MMP9 | **3366** | ABCG2 |
| 83 | CA2 | **1178** | ATP4A | **2273** | CA1 | **3367** | CA7 |
| 84 | CYP11B1 | **1179** | GPR35 | **2274** | FDPS | **3368** | CSNK2A1 |
| 85 | Oprm1 | **1180** | MMP12 | **2275** | Grin1 | **3369** | HSD17B1 |
| 86 | GRIA2 | **1181** | MGLL | **2276** | TUBA1A | **3370** | FFAR1 |
| 87 | RPS6KA3 | **1182** | PNMT | **2277** | CA13 | **3371** | HDAC2 |
| 88 | SERPINE1 | **1183** | lef | **2278** | BCHE | **3372** | GRM4 |
| 89 | TERT | **1184** | HDAC6 | **2279** | RXRG | **3373** | PIM1 |
| 90 | CA5B | **1185** | CA4 | **2280** | CA6 | **3374** | PRKACA |
| 91 | S1PR4 | **1186** | Chrna7 | **2281** | HSD17B3 | **3375** | CA3 |
| 92 | FUT7 | **1187** | TYR | **2282** | ERN1 | **3376** | COMT |
| 93 | DUSP1 | **1188** | YWHAG | **2283** | AKR1B10 | **3377** | AKR1C4 |
| 94 | GFER | **1189** | APEX1 | **2284** | HSPE1 | **3378** | HSPD1 |
| 95 | CYP2A6 | **1190** | CYP2C8 | **2285** | DAPK2 | **3379** | QDPR |
| 96 | DNM1 | **1191** | ELAVL1 | **2286** | ELAVL3 | **3380** | EP300 |
| 97 | ERCC1 | **1192** | FOS | **2287** | KDM4E | **3381** | GLO1 |
| 98 | MYOC | **1193** | NEK6 | **2288** | NFE2L2 | **3382** | NFKB1 |
| 99 | NOX4 | **1194** | NSD2 | **2289** | ALPI | **3383** | ALPG |
| 100 | PSMD14 | **1195** | PTPsigma | **2290** | CXCL12 | **3384** | ST6GAL1 |
| 101 | STK17B | **1196** | SNCA | **2291** | MAPT | **3385** | TUBB1 |
| 102 | TTR | **1197** | ERCC4 | **2292** | ADRA1A | **3386** | SLC5A1 |
| 103 | BCL2 | **1198** | SRC | **2293** | FGFR1 | **3387** | MET |
| 104 | MMP13 | **1199** | PGF | **2294** | VEGFA | **3388** | PGD |
| 105 | ST3GAL3 | **1200** | FUT4 | **2295** | STAT1 | **3389** | SQLE |
| 106 | GABRA1 | **1201** | HIF1A | **2296** | CYP1B1 | **3390** | ERBB2 |
| 107 | FLT1 | **1202** | MMP14 | **2297** | ADORA1 | **3391** | KLK1 |
| 108 | KLK2 | **1203** | ABCC1 | **2298** | CBR1 | **3392** | TAS2R31 |
| 109 | MMP7 | **1204** | MMP8 | **2299** | CBS | **3393** | PGAM1 |
| 110 | IL1B | **1205** | CASP3 | **2300** | BMAL1 | **3394** | CAT |
| 111 | BAX | **1206** | IL6 | **2301** | HMOX1 | **3395** | LEP |
| 112 | SOD2 | **1207** | TP53 | **2302** | SOD1 | **3396** | EGF |
| 113 | NQO1 | **1208** | CYP1A1 | **2303** | CCL2 | **3397** | MPO |
| 114 | CREB1 | **1209** | CTNNB1 | **2304** | CASP1 | **3398** | DDIT3 |
| 115 | GPX1 | **1210** | BDNF | **2305** | CDKN1A | **3399** | INS1 |
| 116 | PDGFBB | **1211** | TRP53 | **2306** | CYP2E1 | **3400** | EDN1 |
| 117 | FAS | **1212** | STAT3 | **2307** | TLR4 | **3401** | BCL2L1 |
| 118 | F2 | **1213** | GSR | **2308** | S100B | **3402** | AGT |
| 119 | CDH1 | **1214** | COX2 | **2309** | IL10 | **3403** | NR0B2 |
| 120 | TFEB | **1215** | TGFB1 | **2310** | TNFSF11 | **3404** | ABCB11 |
| 121 | ACTA2 | **1216** | ALB | **2311** | BECN1 | **3405** | CASP8 |
| 122 | CLOCK | **1217** | F3 | **2312** | GPT | **3406** | HRAS |
| 123 | MFGE8 | **1218** | MFN1 | **2313** | MFN2 | **3407** | MYC |
| 124 | BNIP3 | **1219** | COX4 | **2314** | COX6C | **3408** | CXCL8 |
| 125 | CYFIP2 | **1220** | FIS1 | **2315** | GLI1 | **3409** | H2AX |
| 126 | INSR | **1221** | IRS1 | **2316** | JUN | **3410** | KLF4 |
| 127 | MAP1LC3B | **1222** | MIR122 | **2317** | MUC5AC | **3411** | ND1 |
| 128 | NDUFS1 | **1223** | NFKBIA | **2318** | PCK1 | **3412** | PPARGC1A |
| 129 | PYCARD | **1224** | SLC31A1 | **2319** | TXNRD1 | **3413** | UQCRC2 |
| 130 | APOB | **1225** | AQP3 | **2320** | CDKN1B | **3414** | CDKN2A |
| 131 | CYCS | **1226** | CYP2A5 | **2321** | CYP2B10 | **3415** | CYP2C29 |
| 132 | CYP2D22 | **1227** | CYP3A11 | **2322** | CYP3A13 | **3416** | FOXO1 |
| 133 | GADD45A | **1228** | GCLC | **2323** | MT2A | **3417** | NCF1 |
| 134 | PDHA1 | **1229** | PIK3R1 | **2324** | RB1 | **3418** | SLC2A3 |
| 135 | SLCO1A2 | **1230** | SREBF1 | **2325** | VCAM1 | **3419** | ADAMTS5 |
| 136 | APC | **1231** | APOE | **2326** | ATG12 | **3420** | BAD |
| 137 | CXCL1 | **1232** | CYBB | **2327** | DHFR | **3421** | DNMT3A |
| 138 | DNMT3B | **1233** | EGR1 | **2328** | ENOX2 | **3422** | FASN |
| 139 | FN1 | **1234** | GCLM | **2329** | GLUD1 | **3423** | GYS2 |
| 140 | HGF | **1235** | IL1A | **2330** | MLH1 | **3424** | MT1X |
| 141 | PER1 | **1236** | RNASE1 | **2331** | RUNX2 | **3425** | SFRP1 |
| 142 | SLC2A4 | **1237** | SLC51A | **2332** | SMAD4 | **3426** | SP1 |
| 143 | SQSTM1 | **1238** | TCAF2 | **2333** | TOP2A | **3427** | UBA7 |
| 144 | VIM | **1239** | XRCC3 | **2334** | ACTB | **3428** | ADIPOQ |
| 145 | AGER | **1240** | AGTR1 | **2335** | AKT3 | **3429** | APOV1 |
| 146 | ARNT | **1241** | ATG5 | **2336** | ATP6 | **3430** | BRCA1 |
| 147 | BTG1 | **1242** | CDH2 | **2337** | COL2A1 | **3431** | COL3A1 |
| 148 | COX17 | **1243** | CRY1 | **2338** | CRY2 | **3432** | CYP7A1 |
| 149 | DAPK1 | **1244** | DHCR7 | **2339** | DNM1L | **3433** | FADD |
| 150 | FBN1 | **1245** | FOXO3 | **2340** | G6PC1 | **3434** | GDF9 |
| 151 | GPAM | **1246** | GSDMD | **2341** | GSTM2 | **3435** | GSTO1 |
| 152 | GUSB | **1247** | H1-5 | **2342** | HIVEP1 | **3436** | HMGCS1 |
| 153 | HSPA1A | **1248** | IL18 | **2343** | IL2 | **3437** | IRS2 |
| 154 | LPL | **1249** | LSM6 | **2344** | MAP2K2 | **3438** | MAP3K8 |
| 155 | MCTS1 | **1250** | MGMT | **2345** | MIR33A | **3439** | MYOD1 |
| 156 | ND2 | **1251** | NRG1 | **2346** | OGG1 | **3440** | PDLIM5 |
| 157 | PLD1 | **1252** | PLD2 | **2347** | POLE2 | **3441** | PPARG |
| 158 | PPP1R15A | **1253** | RHOBTB1 | **2348** | RPS3A | **3442** | SCD1 |
| 159 | SERPINB5 | **1254** | SLC30A1 | **2349** | SLC51B | **3443** | SMAD3 |
| 160 | SPRY4 | **1255** | TRAF6 | **2350** | TRANK1 | **3444** | TXN |
| 161 | XBP1 | **1256** | XRCC1 | **2351** | ABCA1 | **3445** | ABCC2 |
| 162 | ABCD3 | **1257** | ABCG1 | **2352** | ACACA | **3446** | ACADM |
| 163 | ACAN | **1258** | ADAMTS15 | **2353** | ADSS2 | **3447** | AFF1 |
| 164 | AHCY | **1259** | AKAP13 | **2354** | AKT | **3448** | AKT2 |
| 165 | ALDH3A2 | **1260** | AMH | **2355** | AMMECR1 | **3449** | AMPD1 |
| 166 | ANAPC10 | **1261** | ANG | **2356** | APAF1 | **3450** | AQP1 |
| 167 | ARHGAP12 | **1262** | ATAD2B | **2357** | ATF4 | **3451** | ATP2B4 |
| 168 | ATXN7L1 | **1263** | AVIL | **2358** | BAAT | **3452** | BAG1 |
| 169 | BAZ1A | **1264** | BCL2L11 | **2359** | BCL3 | **3453** | BGLAP |
| 170 | BID | **1265** | BIRC3 | **2360** | BIRC5 | **3454** | BLTP1 |
| 171 | BTBD8 | **1266** | BTN3A1 | **2361** | C2CD2L | **3455** | CASP7 |
| 172 | CBLB | **1267** | CCDC138 | **2362** | CCDC167 | **3456** | CCDC62 |
| 173 | CCDC91 | **1268** | CCL3 | **2363** | CCL4 | **3457** | CCL5 |
| 174 | CCN2 | **1269** | CCNG1 | **2364** | CD19 | **3458** | CD44 |
| 175 | CD58 | **1270** | CD9 | **2365** | CDC42 | **3459** | CDK5R1 |
| 176 | CDK6 | **1271** | CDON | **2366** | CEBPA | **3460** | CEP15 |
| 177 | CFAP141 | **1272** | CHAC1 | **2367** | CHAC2 | **3461** | CHCHD1 |
| 178 | CLP1 | **1273** | CNKSR2 | **2368** | CNOT2 | **3462** | COA6 |
| 179 | COL1A1 | **1274** | COXFA4L3 | **2369** | CPT1A | **3463** | CRADD |
| 180 | CRP | **1275** | CRYBB2 | **2370** | CRYZL1 | **3464** | CSPG4 |
| 181 | CXCL10 | **1276** | CYB561D2 | **2371** | DDX60 | **3465** | DENND2C |
| 182 | DGKH | **1277** | DIABLO | **2372** | DNAAF1 | **3466** | DNAJC19 |
| 183 | DRC7 | **1278** | DUSP5 | **2373** | DYNLT1 | **3467** | EFNB2 |
| 184 | EHHADH | **1279** | EHMT1 | **2374** | ELK1 | **3468** | ELOVL1 |
| 185 | EPG5 | **1280** | ERRFI1 | **2375** | ETFRF1 | **3469** | FAM162A |
| 186 | FAM177B | **1281** | FAN1 | **2376** | FAT1 | **3470** | FAT4 |
| 187 | FCN3 | **1282** | FGF15 | **2377** | FGF18 | **3471** | FGF2 |
| 188 | FILIP1L | **1283** | FLRT2 | **2378** | FNDC7 | **3472** | FOXP1 |
| 189 | FRY | **1284** | FUCA2 | **2379** | GIN1 | **3473** | GLCCI1 |
| 190 | GLI3 | **1285** | GLIS3 | **2380** | GPR21 | **3474** | GRN |
| 191 | GSDME | **1286** | GSS | **2381** | GTF3C2 | **3475** | H2AC21 |
| 192 | H2AC6 | **1287** | H2BC9 | **2382** | HAUS1 | **3476** | HBEGF |
| 193 | HEATR5A | **1288** | HERC1 | **2383** | HERPUD1 | **3477** | HES1 |
| 194 | HEXIM1 | **1289** | HHIPL2 | **2384** | HIVEP2 | **3478** | HK2 |
| 195 | HMGA1 | **1290** | HMGA2 | **2385** | HOXC10 | **3479** | HSPA5 |
| 196 | HTATIP2 | **1291** | HTR1D | **2386** | IFIH1 | **3480** | IFIT1 |
| 197 | IFITM3 | **1292** | IFNG | **2387** | IFNLR1 | **3481** | IGF1 |
| 198 | IL10RB | **1293** | IL13 | **2388** | IL27RA | **3482** | IL2RA |
| 199 | IP6K2 | **1294** | ITGA6 | **2389** | ITGB3BP | **3483** | ITGB4 |
| 200 | ITPR2 | **1295** | KATNIP | **2390** | KCNJ6 | **3484** | KCNT1 |
| 201 | KEAP1 | **1296** | KMT2C | **2391** | KNTC1 | **3485** | LACTB2 |
| 202 | LDLR | **1297** | LINC00324 | **2392** | LMNB1 | **3486** | LPCAT4 |
| 203 | LRR1 | **1298** | LRRFIP1 | **2393** | LSMEM1 | **3487** | LY96 |
| 204 | MACF1 | **1299** | MAGEB1 | **2394** | MARCHF3 | **3488** | MBD5 |
| 205 | MID1 | **1300** | MKX | **2395** | MOAP1 | **3489** | MOCOS |
| 206 | MOS | **1301** | MRPL16 | **2396** | MRPL33 | **3490** | MSMO1 |
| 207 | MX1 | **1302** | MYCBP2 | **2397** | MYD88 | **3491** | MYO1A |
| 208 | NCF2 | **1303** | NCOA1 | **2398** | NCOA2 | **3492** | NF1 |
| 209 | NFIA | **1304** | NNMT | **2399** | NPHS1 | **3493** | NPHS2 |
| 210 | NR4A2 | **1305** | NSMCE2 | **2400** | NUP37 | **3494** | NXF5 |
| 211 | ODC1 | **1306** | OLIG1 | **2401** | OR3A3 | **3495** | OVAL |
| 212 | P4HB | **1307** | PABPC1 | **2402** | PABPC3 | **3496** | PARK |
| 213 | PCDH18 | **1308** | PDCD10 | **2403** | PDCD5 | **3497** | PEAK1 |
| 214 | PECAM1 | **1309** | PHF19 | **2404** | PIGA | **3498** | PIK3C3 |
| 215 | PLCE1 | **1310** | PLEC | **2405** | PLOD3 | **3499** | PLS1 |
| 216 | PNPLA2 | **1311** | POC5 | **2406** | PPP1R12A | **3500** | PPP2R3A |
| 217 | PRDX2 | **1312** | PRICKLE2 | **2407** | PRKCSH | **3501** | PROZ |
| 218 | PRPSAP2 | **1313** | PSMA2 | **2408** | PTP4A1 | **3502** | RAB1C |
| 219 | RAB33B | **1314** | RAN | **2409** | RASAL2 | **3503** | RASGRP3 |
| 220 | RBX1 | **1315** | RGCC | **2410** | RGL1 | **3504** | RIDA |
| 221 | RNF213 | **1316** | ROR2 | **2411** | RPL13 | **3505** | RPL17 |
| 222 | RPL27A | **1317** | RPL37A | **2412** | RPTOR | **3506** | RRP8 |
| 223 | SAFB2 | **1318** | SAMD9L | **2413** | SASH1 | **3507** | SCD |
| 224 | SELP | **1319** | SELPLG | **2414** | SERF1B | **3508** | SESN3 |
| 225 | SF1 | **1320** | SH3PXD2B | **2415** | SHTN1 | **3509** | SIDT2 |
| 226 | SIPA1L1 | **1321** | SIRT3 | **2416** | SKN-1 | **3510** | SLC10A1 |
| 227 | SLC20A1 | **1322** | SLC25A2 | **2417** | SLC2A1 | **3511** | SLC39A14 |
| 228 | SLC40A1 | **1323** | SLC50A1 | **2418** | SLC6A13 | **3512** | SLPI |
| 229 | SLX9 | **1324** | SMARCAL1 | **2419** | SMN2 | **3513** | SMUG1 |
| 230 | SNAI1 | **1325** | SNHG32 | **2420** | SNORA28 | **3514** | SNORA60 |
| 231 | SNORD15A | **1326** | SNORD21 | **2421** | SNORD44 | **3515** | SNORD49A |
| 232 | SNORD49B | **1327** | SNORD50A | **2422** | SNORD51 | **3516** | SNORD55 |
| 233 | SNORD58A | **1328** | SNORD74 | **2423** | SNORD75 | **3517** | SNORD78 |
| 234 | SNORD79 | **1329** | SNORD82 | **2424** | SNORD83B | **3518** | SOD3 |
| 235 | SOX9 | **1330** | SREBF2 | **2425** | SRGAP1 | **3519** | ST6GALNAC5 |
| 236 | STXBP6 | **1331** | SYNE2 | **2426** | TACO1 | **3520** | TANC2 |
| 237 | TBC1D32 | **1332** | TEAD4 | **2427** | TEFM | **3521** | TERF2 |
| 238 | TEX30 | **1333** | TH | **2428** | THOC1 | **3522** | TLR2 |
| 239 | TLR3 | **1334** | TMEM120B | **2429** | TMEM165 | **3523** | TMEM60 |
| 240 | TNFRSF8 | **1335** | TNFSF10 | **2430** | TOP1 | **3524** | TRERF1 |
| 241 | TSPAN15 | **1336** | TUBB8 | **2431** | TULP4 | **3525** | UGT1A10 |
| 242 | UGT1A6 | **1337** | UGT1A7 | **2432** | UGT1A8 | **3526** | UGT2B15 |
| 243 | UGT2B4 | **1338** | UGT2B7 | **2433** | UTRN | **3527** | VPS13D |
| 244 | VTG2 | **1339** | VWA3A | **2434** | WDFY3 | **3528** | WDHD1 |
| 245 | WNT5A | **1340** | XIAP | **2435** | XRCC4 | **3529** | YAE1 |
| 246 | ZFAND2A | **1341** | ZFAND6 | **2436** | ZFAT | **3530** | ZFP36L2 |
| 247 | ZMYM2 | **1342** | ZNF280C | **2437** | ZNF432 | **3531** | ZNF442 |
| 248 | ZNF804A | **1343** | ZSCAN9 | **2438** | A1CF | **3532** | AAMDC |
| 249 | AAR2 | **1344** | AASDH | **2439** | ABCB10 | **3533** | ABCC10 |
| 250 | ABCC4 | **1345** | ABCC5 | **2440** | ABCE1 | **3534** | ABHD6 |
| 251 | ABL2 | **1346** | ABR | **2441** | ACADSB | **3535** | ACAP2 |
| 252 | ACAP3 | **1347** | ACAT2 | **2442** | ACCSL | **3536** | ACE2 |
| 253 | ACKR5 | **1348** | ACLY | **2443** | ACOT1 | **3537** | ACOT13 |
| 254 | ACOT6 | **1349** | ACOX1 | **2444** | ACRBP | **3538** | ACSL1 |
| 255 | ACSM4 | **1350** | ACSS2 | **2445** | ACSS3 | **3539** | ACTBL2 |
| 256 | ACTL6B | **1351** | ACTR10 | **2446** | ADAD2 | **3540** | ADAM19 |
| 257 | ADAM20 | **1352** | ADAM30 | **2447** | ADAM32 | **3541** | ADAM33 |
| 258 | ADAMTS12 | **1353** | ADAMTS4 | **2448** | ADAMTSL4-AS1 | **3542** | ADARB1 |
| 259 | ADAT1 | **1354** | ADD2 | **2449** | ADGRB2 | **3543** | ADGRE1 |
| 260 | ADGRF2P | **1355** | ADGRG7 | **2450** | ADIPOR2 | **3544** | ADM |
| 261 | ADNP2 | **1356** | ADRA1B | **2451** | ADRB2 | **3545** | ADTRP |
| 262 | AFG1L | **1357** | AFP | **2452** | AGAP1 | **3546** | AGL |
| 263 | AGO1 | **1358** | AGO4 | **2453** | AGPAT3 | **3547** | AGPS |
| 264 | AIDA | **1359** | AIFM1 | **2454** | AIMP1 | **3548** | AIPL1 |
| 265 | AJAP1 | **1360** | AJUBA | **2455** | AKAP12 | **3549** | AKAP9 |
| 266 | AKIP1 | **1361** | AKIRIN2 | **2456** | AKR1C1 | **3550** | AKR1C2 |
| 267 | AKTIP | **1362** | ALDOA | **2457** | ALDOC | **3551** | ALG14 |
| 268 | ALG6 | **1363** | ALG8 | **2458** | ALLC | **3552** | ALPK2 |
| 269 | ALPK3 | **1364** | ALPPL2 | **2459** | ALS2 | **3553** | ALX1 |
| 270 | AMBP | **1365** | AMZ1 | **2460** | AMZ2P1 | **3554** | ANAPC1 |
| 271 | ANAPC4 | **1366** | ANAPC7 | **2461** | ANGPTL2 | **3555** | ANKIB1 |
| 272 | ANKMY2 | **1367** | ANKRD10 | **2462** | ANKRD20A20P | **3556** | ANKRD26 |
| 273 | ANKRD36 | **1368** | ANKRD39 | **2463** | ANKRD44 | **3557** | ANKUB1 |
| 274 | ANO1 | **1369** | ANO4 | **2464** | ANO6 | **3558** | ANP |
| 275 | ANXA11 | **1370** | ANXA13 | **2465** | ANXA2 | **3559** | ANXA2R |
| 276 | ANXA4 | **1371** | ANXA7 | **2466** | AOPEP | **3560** | AOX2P |
| 277 | AP1S3 | **1372** | AP2A2 | **2467** | AP3S1 | **3561** | AP5S1 |
| 278 | APBB2 | **1373** | APLN | **2468** | APOA4 | **3562** | APOL2 |
| 279 | APOL4 | **1374** | APPL2 | **2469** | AQR | **3563** | ARAP3 |
| 280 | ARC | **1375** | ARCN1 | **2470** | AREG | **3564** | ARFGEF1 |
| 281 | ARFGEF3 | **1376** | ARGLU1 | **2471** | ARHGAP10 | **3565** | ARHGAP11A |
| 282 | ARHGAP18 | **1377** | ARHGAP20 | **2472** | ARHGAP21 | **3566** | ARHGAP24 |
| 283 | ARHGAP28 | **1378** | ARHGAP29 | **2473** | ARHGAP4 | **3567** | ARHGAP42 |
| 284 | ARHGEF26 | **1379** | ARHGEF28 | **2474** | ARHGEF3 | **3568** | ARL15 |
| 285 | ARMC1 | **1380** | ARMC10 | **2475** | ARMC9 | **3569** | ARMS2 |
| 286 | ARPC5L | **1381** | ARPIN | **2476** | ARRB1 | **3570** | ARRDC3 |
| 287 | ARSI | **1382** | ARSJ | **2477** | ARSK | **3571** | ART4 |
| 288 | ARV1 | **1383** | ASAP1 | **2478** | ASAP2 | **3572** | ASAP3 |
| 289 | ASB15 | **1384** | ASB6 | **2479** | ASCC3 | **3573** | ASF1A |
| 290 | ASH2L | **1385** | ASIC4 | **2480** | ASPM | **3574** | ASXL2 |
| 291 | ATE1 | **1386** | ATF2 | **2481** | ATF3 | **3575** | ATF6 |
| 292 | ATG10 | **1387** | ATG4A | **2482** | ATG4C | **3576** | ATG4D |
| 293 | ATL1 | **1388** | ATM | **2483** | ATN1 | **3577** | ATOH1 |
| 294 | ATP10B | **1389** | ATP10D | **2484** | ATP13A1 | **3578** | ATP1A1 |
| 295 | ATP1A4 | **1390** | ATP1B1 | **2485** | ATP1B4 | **3579** | ATP2A1 |
| 296 | ATP2A2 | **1391** | ATP2B1 | **2486** | ATP2B2 | **3580** | ATP2B3 |
| 297 | ATP5MC3 | **1392** | ATP5ME | **2487** | ATP5MGL | **3581** | ATP6AP1L |
| 298 | ATP6V0C | **1393** | ATP7B | **2488** | ATP8B1 | **3582** | ATPAF1 |
| 299 | ATR | **1394** | ATXN2 | **2489** | ATXN2L | **3583** | ATXN3 |
| 300 | AXUD1 | **1395** | AZIN1 | **2490** | B3GALT2 | **3584** | B3GLCT |
| 301 | B4GALT1 | **1396** | BAG2 | **2491** | BAG3 | **3585** | BAG4 |
| 302 | BAG6 | **1397** | BAIAP2-DT | **2492** | BAK1 | **3586** | BARD1 |
| 303 | BARHL1 | **1398** | BAZ2B | **2493** | BBC3 | **3587** | BBS10 |
| 304 | BBS2 | **1399** | BBS4 | **2494** | BBX | **3588** | BCAS2 |
| 305 | BCL6 | **1400** | BCL7A | **2495** | BDKRB1 | **3589** | BDNF-AS |
| 306 | BHLHE22 | **1401** | BICD2 | **2496** | BIN3 | **3590** | BLID |
| 307 | BLTP3B | **1402** | BLVRA | **2497** | BMP15 | **3591** | BMP2K |
| 308 | BMP4 | **1403** | BMPER | **2498** | BMPR1A | **3592** | BMX |
| 309 | BNC1 | **1404** | BNIPL | **2499** | BNP | **3593** | BOLA1 |
| 310 | BOLA3 | **1405** | BOP1 | **2500** | BORA | **3594** | BPGM |
| 311 | BPNT2 | **1406** | BRCA2 | **2501** | BRCC3 | **3595** | BRD1 |
| 312 | BRD10 | **1407** | BRF1 | **2502** | BRIP1 | **3596** | BRPF3 |
| 313 | BRWD1 | **1408** | BRWD3 | **2503** | BSDC1 | **3597** | BTF3 |
| 314 | BTF3L4 | **1409** | BTLA | **2504** | BTN1A1 | **3598** | BTN3A2 |
| 315 | BTN3A3 | **1410** | BTRC | **2505** | BUB1 | **3599** | BUD31 |
| 316 | BYSL | **1411** | BZW2 | **2506** | C11ORF16 | **3600** | C12ORF50 |
| 317 | C14ORF119 | **1412** | C15ORF39 | **2507** | C16ORF46 | **3601** | C1GALT1 |
| 318 | C1ORF116 | **1413** | C1ORF162 | **2508** | C1ORF21 | **3602** | C1ORF52 |
| 319 | C1QTNF1 | **1414** | C1QTNF2 | **2509** | C22ORF39 | **3603** | C2CD3 |
| 320 | C2CD5 | **1415** | C2ORF69 | **2510** | C3 | **3604** | C3ORF20 |
| 321 | C3ORF22 | **1416** | C4ORF46 | **2511** | C5AR2 | **3605** | C6ORF47 |
| 322 | C8ORF58 | **1417** | C8ORF82 | **2512** | C9 | **3606** | C9ORF152 |
| 323 | CACNB1 | **1418** | CADM4 | **2513** | CALN1 | **3607** | CAMK2D |
| 324 | CAMKMT | **1419** | CAMLG | **2514** | CAND1 | **3608** | CAPN11 |
| 325 | CAPN2 | **1420** | CAPRIN2 | **2515** | CAPZA1 | **3609** | CARD11 |
| 326 | CARMIL1 | **1421** | CARMIL2 | **2516** | CARNMT1 | **3610** | CASC15 |
| 327 | CASC3 | **1422** | CASD1 | **2517** | CASP10 | **3611** | CASP2 |
| 328 | CASP6 | **1423** | CASS4 | **2518** | CAV2 | **3612** | CAVIN2 |
| 329 | CAVIN4 | **1424** | CBLL1 | **2519** | CBX1 | **3613** | CBX4 |
| 330 | CBY1 | **1425** | CC2D1B | **2520** | CC2D2A | **3614** | CC2D2B |
| 331 | CCDC102B | **1426** | CCDC107 | **2521** | CCDC125 | **3615** | CCDC126 |
| 332 | CCDC142 | **1427** | CCDC144A | **2522** | CCDC17 | **3616** | CCDC18 |
| 333 | CCDC6 | **1428** | CCDC7 | **2523** | CCDC70 | **3617** | CCDC71 |
| 334 | CCDC77 | **1429** | CCDC86 | **2524** | CCHCR1 | **3618** | CCL7 |
| 335 | CCNB1IP1 | **1430** | CCNF | **2525** | CCNG2 | **3619** | CCNH |
| 336 | CCNL1 | **1431** | CCNT2 | **2526** | CCNY | **3620** | CCR2 |
| 337 | CCR4 | **1432** | CCR6 | **2527** | CCSER2 | **3621** | CCT6A |
| 338 | CCT8 | **1433** | CD14 | **2528** | CD244 | **3622** | CD2AP |
| 339 | CD300E | **1434** | CD33 | **2529** | CD3G | **3623** | CD4 |
| 340 | CD40 | **1435** | CD68 | **2530** | CD69 | **3624** | CD80 |
| 341 | CD99L2 | **1436** | CDC20B | **2531** | CDC25A | **3625** | CDC25C |
| 342 | CDC27 | **1437** | CDC42EP1 | **2532** | CDC42EP3 | **3626** | CDC42SE2 |
| 343 | CDC73 | **1438** | CDCA3 | **2533** | CDH13 | **3627** | CDIN1 |
| 344 | CDK17 | **1439** | CDK19 | **2534** | CDK5RAP2 | **3628** | CDKL5 |
| 345 | CDKN2AIP | **1440** | CDX2 | **2535** | CDX4 | **3629** | CEACAM20 |
| 346 | CEACAM4 | **1441** | CEBPB | **2536** | CEBPD | **3630** | CEBPG |
| 347 | CELA3A | **1442** | CELP | **2537** | CEMIP2 | **3631** | CENPA |
| 348 | CENPE | **1443** | CENPI | **2538** | CENPP | **3632** | CEP112 |
| 349 | CEP128 | **1444** | CEP170 | **2539** | CEP290 | **3633** | CEP41 |
| 350 | CEP70 | **1445** | CEP83 | **2540** | CEPT1 | **3634** | CETN3 |
| 351 | CFAP161 | **1446** | CFAP206 | **2541** | CFAP276 | **3635** | CFAP47 |
| 352 | CFAP65 | **1447** | CFAP97D1 | **2542** | CFB | **3636** | CFD |
| 353 | CFLAR | **1448** | CHD5 | **2543** | CHD6 | **3637** | CHD7 |
| 354 | CHD9 | **1449** | CHKB | **2544** | CHMP6 | **3638** | CHRD |
| 355 | CHRNA3 | **1450** | CHRNE | **2545** | CHST10 | **3639** | CHST11 |
| 356 | CHST15 | **1451** | CIART | **2546** | CISD1 | **3640** | CIZ1 |
| 357 | CKAP4 | **1452** | CKAP5 | **2547** | CKS2 | **3641** | CLASP1 |
| 358 | CLCN1 | **1453** | CLCN7 | **2548** | CLCNKB | **3642** | CLDN16 |
| 359 | CLDN19 | **1454** | CLDN22 | **2549** | CLDN7 | **3643** | CLEC12A |
| 360 | CLEC4E | **1455** | CLEC5A | **2550** | CLEC7A | **3644** | CLIP4 |
| 361 | CLK3 | **1456** | CLMN | **2551** | CLMP | **3645** | CLPB |
| 362 | CLTCL1 | **1457** | CLUHP3 | **2552** | CMAS | **3646** | CMIP |
| 363 | CMPK2 | **1458** | CMTM2 | **2553** | CNBD1 | **3647** | CNBD2 |
| 364 | CNDP1 | **1459** | CNKSR3 | **2554** | CNNM1 | **3648** | CNNM2 |
| 365 | CNOT11 | **1460** | CNOT6L | **2555** | CNPY2 | **3649** | CNTLN |
| 366 | CNTRL | **1461** | COA3 | **2556** | COG2 | **3650** | COG6 |
| 367 | COG7 | **1462** | COL11A2 | **2557** | COL25A1-DT | **3651** | COL4A1 |
| 368 | COL6A1 | **1463** | COLCA1 | **2558** | COMMD4 | **3652** | COMTD1 |
| 369 | COP1 | **1464** | COQ2 | **2559** | COQ5 | **3653** | COQ6 |
| 370 | COQ9 | **1465** | COTL1 | **2560** | COX1 | **3654** | COX4I1 |
| 371 | CPA3 | **1466** | CPE | **2561** | CPEB2 | **3655** | CPLANE1 |
| 372 | CPLX3 | **1467** | CPNE8 | **2562** | CPSF3 | **3656** | CPTP |
| 373 | CR2 | **1468** | CREB3L1 | **2563** | CREBRF | **3657** | CREBZF |
| 374 | CROCCP2 | **1469** | CROT | **2564** | CRTC2 | **3658** | CRYAB |
| 375 | CSE1L | **1470** | CSF2 | **2565** | CSHL1 | **3659** | CSMD2 |
| 376 | CSNK2A2 | **1471** | CSTPP1 | **2566** | CTAGE4 | **3660** | CTIF |
| 377 | CTNNAL1 | **1472** | CTNND2 | **2567** | CTNS | **3661** | CTPS1 |
| 378 | CTPS2 | **1473** | CTSB | **2568** | CTSK | **3662** | CTSL |
| 379 | CWC25 | **1474** | CXCL2 | **2569** | CXXC5 | **3663** | CYB561D1 |
| 380 | CYB5D1 | **1475** | CYGB | **2570** | CYP1A | **3664** | CYP26A1 |
| 381 | CYP27B1 | **1476** | CYP2B6 | **2571** | CYP2C70 | **3665** | CYP2R1 |
| 382 | CYP3A4 | **1477** | CYP4B1 | **2572** | CYP4F12 | **3666** | CYP4F14 |
| 383 | CYP4F30P | **1478** | CYP51A1 | **2573** | CYREN | **3667** | CYRIB |
| 384 | CYTH3 | **1479** | DAAM1 | **2574** | DAB2 | **3668** | DAP3 |
| 385 | DAPK3 | **1480** | DARS2 | **2575** | DAZAP2 | **3669** | DCAF12L1 |
| 386 | DCAF4L1 | **1481** | DCBLD1 | **2576** | DCHS2 | **3670** | DCLK2 |
| 387 | DCLRE1A | **1482** | DCUN1D4 | **2577** | DDHD1 | **3671** | DDOST |
| 388 | DDX10 | **1483** | DDX20 | **2578** | DDX39A | **3672** | DEDD |
| 389 | DEFA10P | **1484** | DEFB113 | **2579** | DEK | **3673** | DELE1 |
| 390 | DENND2B | **1485** | DENND2D | **2580** | DENND3 | **3674** | DENND4A |
| 391 | DENND4C | **1486** | DENND5B | **2581** | DENND6A | **3675** | DENR |
| 392 | DEPDC4 | **1487** | DEPDC7 | **2582** | DET1 | **3676** | DFFA |
| 393 | DGKB | **1488** | DGKD | **2583** | DHRS9 | **3677** | DHX32 |
| 394 | DHX35 | **1489** | DHX57 | **2584** | DIAPH3 | **3678** | DIMT1L |
| 395 | DIO2 | **1490** | DIO3 | **2585** | DIP2A | **3679** | DIP2B |
| 396 | DIPK1A | **1491** | DIRAS1 | **2586** | DIS3L | **3680** | DIS3L2 |
| 397 | DISP1 | **1492** | DIXDC1 | **2587** | DKK1 | **3681** | DKK4 |
| 398 | DLD | **1493** | DLEU2 | **2588** | DLG1 | **3682** | DLGAP5 |
| 399 | DLK2 | **1494** | DLL4 | **2589** | DLX1 | **3683** | DMAP1 |
| 400 | DMXL1 | **1495** | DMXL2 | **2590** | DNA2 | **3684** | DNAAF19 |
| 401 | DNAAF2 | **1496** | DNAH11 | **2591** | DNAH12 | **3685** | DNAJB4 |
| 402 | DNAJB5 | **1497** | DNAJB9 | **2592** | DNAJC13 | **3686** | DNAJC14 |
| 403 | DNAJC28 | **1498** | DNAJC9 | **2593** | DNASE2B | **3687** | DNHD1 |
| 404 | DNM3 | **1499** | DNPEP | **2594** | DNTTIP1 | **3688** | DNTTIP2 |
| 405 | DOCK10 | **1500** | DOCK11 | **2595** | DOCK7 | **3689** | DOCK9 |
| 406 | DOK1 | **1501** | DOK2 | **2596** | DOP1A | **3690** | DOP1B |
| 407 | DPF3 | **1502** | DPH6 | **2597** | DPP4 | **3691** | DPPA3 |
| 408 | DPPA5 | **1503** | DPY19L2P1 | **2598** | DPY19L4 | **3692** | DR1 |
| 409 | DRAM1 | **1504** | DRC2 | **2599** | DROSHA | **3693** | DSCAML1 |
| 410 | DSCC1 | **1505** | DSCR10 | **2600** | DSE | **3694** | DSG3 |
| 411 | DST | **1506** | DTD2 | **2601** | DTNBP1 | **3695** | DTWD2 |
| 412 | DTYMK | **1507** | DUS2 | **2602** | DUSP12 | **3696** | DUSP4 |
| 413 | DUSP6 | **1508** | DUSP7 | **2603** | DUSP8 | **3697** | DYNC2I1 |
| 414 | DYNLT4 | **1509** | DZIP3 | **2604** | E2F1 | **3698** | EAF1 |
| 415 | EBAG9 | **1510** | EBLN1 | **2605** | EBP | **3699** | ECE1 |
| 416 | ECHDC2 | **1511** | ECHS1 | **2606** | ECI2 | **3700** | ECM1 |
| 417 | ECT2 | **1512** | EDEM3 | **2607** | EEA1 | **3701** | EEF1A1 |
| 418 | EEF1B2 | **1513** | EEF2K | **2608** | EEIG2 | **3702** | EFHC1 |
| 419 | EFL1 | **1514** | EFNA1 | **2609** | EFNA4 | **3703** | EFNA5 |
| 420 | EFNB1 | **1515** | EFR3A | **2610** | EFR3B | **3704** | EGLN1 |
| 421 | EHBP1 | **1516** | EHD2 | **2611** | EIF2AK4 | **3705** | EIF2S3 |
| 422 | EIF3B | **1517** | EIF3CL | **2612** | EIF3F | **3706** | EIF4A2 |
| 423 | EIF4B | **1518** | EIF4E1B | **2613** | EIF4EBP2 | **3707** | EIF5A |
| 424 | EIPR1 | **1519** | ELAC1 | **2614** | ELAVL2 | **3708** | ELF1 |
| 425 | ELL2 | **1520** | ELOA | **2615** | ELOA2 | **3709** | ELOF1 |
| 426 | ELOVL2 | **1521** | ELP4 | **2616** | EMC2 | **3710** | EMD |
| 427 | EMG1 | **1522** | EML4 | **2617** | ENDOV | **3711** | ENG |
| 428 | EOLA2 | **1523** | EPB41L4A-AS1 | **2618** | EPB41L4B | **3712** | EPHA4 |
| 429 | EPHA5 | **1524** | EPHA8 | **2619** | EPHB4 | **3713** | EPM2AIP1 |
| 430 | EPN2 | **1525** | EPPK1 | **2620** | EPS8 | **3714** | EPSTI1 |
| 431 | EPX | **1526** | ERAP1 | **2621** | ERC1 | **3715** | ERCC6L2 |
| 432 | ERGIC2 | **1527** | ERICH1 | **2622** | ERLEC1 | **3716** | ERMARD |
| 433 | ERMN | **1528** | ERP29 | **2623** | ERV18-1 | **3717** | ESD |
| 434 | ESPNP | **1529** | ESR2B | **2624** | ETAA1 | **3718** | ETV1 |
| 435 | ETV3 | **1530** | ETV5 | **2625** | ETV6 | **3719** | EVPL |
| 436 | EWSR1 | **1531** | EXOC1 | **2626** | EXOC2 | **3720** | EXOC4 |
| 437 | EXOC6 | **1532** | EXPH5 | **2627** | EYA2 | **3721** | EZHIP |
| 438 | F11 | **1533** | F13B | **2628** | F2RL3 | **3722** | F8A1 |
| 439 | F8A2 | **1534** | FABP12 | **2629** | FABP4 | **3723** | FADS1 |
| 440 | FADS3 | **1535** | FAIM | **2630** | FAM110B | **3724** | FAM114A1 |
| 441 | FAM118B | **1536** | FAM120A2P | **2631** | FAM129A | **3725** | FAM131A |
| 442 | FAM153A | **1537** | FAM180A | **2632** | FAM181A-AS1 | **3726** | FAM193B |
| 443 | FAM238A | **1538** | FAM3C | **2633** | FAM43B | **3727** | FAM50B |
| 444 | FAM72D | **1539** | FAM76A | **2634** | FAM83C | **3728** | FAM83G |
| 445 | FAM83H | **1540** | FANCC | **2635** | FANCM | **3729** | FAR2 |
| 446 | FARS2 | **1541** | FASLG | **2636** | FASN1 | **3730** | FASTKD1 |
| 447 | FASTKD2 | **1542** | FAU | **2637** | FAXDC2 | **3731** | FBH1 |
| 448 | FBN2 | **1543** | FBP1 | **2638** | FBXL12 | **3732** | FBXL16 |
| 449 | FBXL2 | **1544** | FBXL4 | **2639** | FBXL5 | **3733** | FBXL7 |
| 450 | FBXO27 | **1545** | FBXO28 | **2640** | FBXO48 | **3734** | FCAMR |
| 451 | FCHO2 | **1546** | FCHSD2 | **2641** | FCRL3 | **3735** | FCRL5 |
| 452 | FCRL6 | **1547** | FEM1A | **2642** | FEM1C | **3736** | FER |
| 453 | FERMT2 | **1548** | FERMT3 | **2643** | FGD2 | **3737** | FGD6 |
| 454 | FGF1 | **1549** | FGF12 | **2644** | FGF17 | **3738** | FGFBP1 |
| 455 | FGFR4 | **1550** | FIRRM | **2645** | FKBP10 | **3739** | FKBP9 |
| 456 | FLI1 | **1551** | FLNA | **2646** | FLT3LG | **3740** | FMN1 |
| 457 | FMNL2 | **1552** | FMO5 | **2647** | FNBP1L | **3741** | FNDC3A |
| 458 | FNDC3B | **1553** | FOCAD | **2648** | FOSB | **3742** | FOXD3 |
| 459 | FOXD4 | **1554** | FOXD4L1 | **2649** | FOXE3 | **3743** | FOXF1 |
| 460 | FOXG1 | **1555** | FOXL1 | **2650** | FOXP3 | **3744** | FOXQ1 |
| 461 | FRMD5 | **1556** | FRMD6 | **2651** | FRMD7 | **3745** | FRS2 |
| 462 | FRYL | **1557** | FRZB | **2652** | FSCN2 | **3746** | FSCN3 |
| 463 | FSTL4 | **1558** | FTH1 | **2653** | FTHL17 | **3747** | FUNDC1 |
| 464 | FUT3 | **1559** | FUT8 | **2654** | FXN | **3748** | FXYD5 |
| 465 | FZD1 | **1560** | FZD2 | **2655** | FZD5 | **3749** | G2E3 |
| 466 | G6PD | **1561** | GAA | **2656** | GAB1 | **3750** | GAB2 |
| 467 | GABRE | **1562** | GABRR1 | **2657** | GAD1 | **3751** | GAL |
| 468 | GALM | **1563** | GALNT1 | **2658** | GALNT14 | **3752** | GANAB |
| 469 | GAPT | **1564** | GARIN1A | **2659** | GARIN4 | **3753** | GASK1B |
| 470 | GATA3-AS1 | **1565** | GATB | **2660** | GBE1 | **3754** | GBP1 |
| 471 | GBP2 | **1566** | GBP3 | **2661** | GBP4 | **3755** | GCA |
| 472 | GCC1 | **1567** | GCKR | **2662** | GCNA | **3756** | GCNT1 |
| 473 | GCNT2 | **1568** | GCNT7 | **2663** | GDF15 | **3757** | GDF3 |
| 474 | GDPD1 | **1569** | GDPD3 | **2664** | GEMIN6 | **3758** | GEMIN7 |
| 475 | GFAP | **1570** | GFI1B | **2665** | GFOD1 | **3759** | GFPT2 |
| 476 | GFY | **1571** | GGACT | **2666** | GHSR | **3760** | GID8 |
| 477 | GIGYF2 | **1572** | GIMAP1 | **2667** | GINM1 | **3761** | GINS1 |
| 478 | GIT2 | **1573** | GJA4 | **2668** | GJA8 | **3762** | GJA9 |
| 479 | GJB1 | **1574** | GJC3 | **2669** | GJD4 | **3763** | GLDN |
| 480 | GLI2 | **1575** | GLIDR | **2670** | GLMN | **3764** | GLRX2 |
| 481 | GLS | **1576** | GLT1D1 | **2671** | GLT8D1 | **3765** | GLYATL2 |
| 482 | GLYATL3 | **1577** | GMPR | **2672** | GNA13 | **3766** | GNAI1 |
| 483 | GNAQ | **1578** | GNAT1 | **2673** | GNE | **3767** | GNL3 |
| 484 | GNPTAB | **1579** | GNRH1 | **2674** | GNRH2 | **3768** | GOLGA1 |
| 485 | GOLGA6A | **1580** | GOLIM4 | **2675** | GOLM2 | **3769** | GOT2 |
| 486 | GP5 | **1581** | GPAA1 | **2676** | GPATCH4 | **3770** | GPHN |
| 487 | GPR101 | **1582** | GPR107 | **2677** | GPR160 | **3771** | GPR162 |
| 488 | GPR176 | **1583** | GPR180 | **2678** | GPR19 | **3772** | GPR25 |
| 489 | GPR32 | **1584** | GPR39 | **2679** | GPR82 | **3773** | GPR83 |
| 490 | GPR87 | **1585** | GPR88 | **2680** | GPR89B | **3774** | GPRC5B |
| 491 | GPS2 | **1586** | GPSM2 | **2681** | GRAMD1B | **3775** | GRAMD2B |
| 492 | GREB1L | **1587** | GRHL3 | **2682** | GRIK4 | **3776** | GRIN2A |
| 493 | GRK3 | **1588** | GSAP | **2683** | GSDMC | **3777** | GSTA1 |
| 494 | GSTA4 | **1589** | GSTM1 | **2684** | GSTM5 | **3778** | GSTP1 |
| 495 | GSTS1 | **1590** | GTF2A2 | **2685** | GTF2H5 | **3779** | GTF2IRD1 |
| 496 | GTF3C4 | **1591** | GTPBP2 | **2686** | GTPBP6 | **3780** | GUCY2C |
| 497 | GULP1 | **1592** | H1-0 | **2687** | H1-1 | **3781** | H1-2 |
| 498 | H1-3 | **1593** | H1-4 | **2688** | H1-6 | **3782** | H1-8 |
| 499 | H2ACP1 | **1594** | H2AZ1 | **2689** | H2AZ2 | **3783** | H2BC1 |
| 500 | H2BC14 | **1595** | H2BC18 | **2690** | H2BC26 | **3784** | H2BC3 |
| 501 | H2BC8 | **1596** | H3C12 | **2691** | H3C3 | **3785** | H4C15 |
| 502 | H4C8 | **1597** | H4C9 | **2692** | HACD4 | **3786** | HAND1 |
| 503 | HAND2 | **1598** | HAPLN3 | **2693** | HAPSTR1 | **3787** | HAS1 |
| 504 | HAT1 | **1599** | HAVCR1 | **2694** | HAVCR2 | **3788** | HBA1 |
| 505 | HBP1 | **1600** | HCFC1 | **2695** | HDAC5 | **3789** | HEATR3 |
| 506 | HEATR5B | **1601** | HEATR6 | **2696** | HECA | **3790** | HEG1 |
| 507 | HEMK1 | **1602** | HEPACAM | **2697** | HERC4 | **3791** | HERC6 |
| 508 | HERPUD2 | **1603** | HEY1 | **2698** | HGD | **3792** | HGSNAT |
| 509 | HHATL | **1604** | HHEX | **2699** | HIBCH | **3793** | HIF1AN |
| 510 | HIKESHI | **1605** | HIP1 | **2700** | HLA-DOA | **3794** | HLCS |
| 511 | HLTF | **1606** | HMGB1 | **2701** | HMGCLL1 | **3795** | HMGCS2 |
| 512 | HMGN3 | **1607** | HMMR | **2702** | HMOX2 | **3796** | HMSD |
| 513 | HMX1 | **1608** | HMX3 | **2703** | HNRNPA1 | **3797** | HNRNPA1L2 |
| 514 | HNRNPF | **1609** | HNRNPH1 | **2704** | HNRNPH2 | **3798** | HOMER1 |
| 515 | HOOK2 | **1610** | HOPX | **2705** | HORMAD1 | **3799** | HORMAD2 |
| 516 | HOXA10 | **1611** | HOXB2 | **2706** | HOXB9 | **3800** | HOXC11 |
| 517 | HOXD3 | **1612** | HOXD9 | **2707** | HPF1 | **3801** | HPS3 |
| 518 | HPS5 | **1613** | HS3ST3B1 | **2708** | HS3ST5 | **3802** | HS6ST3 |
| 519 | HSD17B7P2 | **1614** | HSDL2 | **2709** | HSP90AA2P | **3803** | HSP90AB2P |
| 520 | HSP90B2P | **1615** | HSPA1B | **2710** | HSPA1L | **3804** | HSPA4 |
| 521 | HSPA4L | **1616** | HSPA8 | **2711** | HSPB7 | **3805** | HTRA2 |
| 522 | HUS1B | **1617** | HYLS1 | **2712** | IBTK | **3806** | ICE2 |
| 523 | ICOSLG | **1618** | ID2 | **2713** | ID3 | **3807** | IDE |
| 524 | IDI1 | **1619** | IDI2-AS1 | **2714** | IER2 | **3808** | IER5L |
| 525 | IFI27 | **1620** | IFI44 | **2715** | IFI6 | **3809** | IFIT3 |
| 526 | IFIT5 | **1621** | IFITM1 | **2716** | IFNA2 | **3810** | IFNA5 |
| 527 | IFT46 | **1622** | IFT70B | **2717** | IFT74 | **3811** | IFT80 |
| 528 | IFT81 | **1623** | IFT88 | **2718** | IGBP1 | **3812** | IGDCC3 |
| 529 | IGF2 | **1624** | IGF2BP3 | **2719** | IGFBP5 | **3813** | IGFBPL1 |
| 530 | IGSF1 | **1625** | IKBKG | **2720** | IKZF2 | **3814** | IL11 |
| 531 | IL12B | **1626** | IL17RA | **2721** | IL19 | **3815** | IL1R1 |
| 532 | IL23A | **1627** | IL2RB | **2722** | IL32 | **3816** | IL5 |
| 533 | IL5RA | **1628** | IL7 | **2723** | IL7R | **3817** | ILF2 |
| 534 | ILF3 | **1629** | IMMP1L | **2724** | IMPG2 | **3818** | INAVA |
| 535 | ING3 | **1630** | INHBA | **2725** | INPP5A | **3819** | INPP5F |
| 536 | INS | **1631** | INSIG1 | **2726** | INSM2 | **3820** | INSYN2B |
| 537 | INTS1 | **1632** | INTS10 | **2727** | INTS5 | **3821** | INTS8 |
| 538 | INTS9 | **1633** | INTU | **2728** | INVS | **3822** | IPO11 |
| 539 | IQCN | **1634** | IQUB | **2729** | IRAK1BP1 | **3823** | IRAK3 |
| 540 | IREB2 | **1635** | IRF2 | **2730** | IRF4 | **3824** | IRF9 |
| 541 | IRGC | **1636** | IRS4 | **2731** | ISCA2 | **3825** | ISL2 |
| 542 | ITCH | **1637** | ITGA2B | **2732** | ITGA4 | **3826** | ITGAL |
| 543 | ITGAV | **1638** | ITGB1BP2 | **2733** | ITGB2 | **3827** | ITGB6 |
| 544 | ITPA | **1639** | ITPRIP | **2734** | ITSN1 | **3828** | IVNS1ABP |
| 545 | IWS1 | **1640** | IZUMO1R | **2735** | JAG1 | **3829** | JAG2 |
| 546 | JARID2 | **1641** | JAZF1 | **2736** | JDP2 | **3830** | JOSD1 |
| 547 | KANK1 | **1642** | KANSL2 | **2737** | KAT14 | **3831** | KAT2B |
| 548 | KAT5 | **1643** | KAT6A | **2738** | KAT6B | **3832** | KATNAL1 |
| 549 | KATNBL1 | **1644** | KAZN | **2739** | KBTBD2 | **3833** | KCNC2 |
| 550 | KCNE3 | **1645** | KCNH1 | **2740** | KCNK7 | **3834** | KCNMB4 |
| 551 | KCNS3 | **1646** | KCNT2 | **2741** | KCTD10 | **3835** | KCTD16 |
| 552 | KCTD5 | **1647** | KCTD9 | **2742** | KDELR3 | **3836** | KDM4C |
| 553 | KDM5C | **1648** | KHDC4 | **2743** | KHK | **3837** | KIAA0586 |
| 554 | KIAA1328 | **1649** | KIAA1614 | **2744** | KIDINS220 | **3838** | KIF13A |
| 555 | KIF14 | **1650** | KIF15 | **2745** | KIF16B | **3839** | KIF18A |
| 556 | KIF20B | **1651** | KIF4A | **2746** | KIF4B | **3840** | KIFAP3 |
| 557 | KL | **1652** | KLF10 | **2747** | KLF5 | **3841** | KLF7 |
| 558 | KLHDC2 | **1653** | KLHDC3 | **2748** | KLHL12 | **3842** | KLHL15 |
| 559 | KLHL18 | **1654** | KLHL20 | **2749** | KLHL21 | **3843** | KLHL26 |
| 560 | KLHL8 | **1655** | KLHL9 | **2750** | KLK11 | **3844** | KLK14 |
| 561 | KLK3 | **1656** | KLRA1 | **2751** | KNSTRN | **3845** | KPNA2 |
| 562 | KPNB1 | **1657** | KRABD3 | **2752** | KRIT1 | **3846** | KRT1 |
| 563 | KRT77 | **1658** | KRT79 | **2753** | KRTAP10-5 | **3847** | KRTAP12-2 |
| 564 | KRTAP13-2 | **1659** | KRTAP19-4 | **2754** | KRTAP9-9 | **3848** | KRTCAP2 |
| 565 | L3MBTL3 | **1660** | LACTB | **2755** | LAIR1 | **3849** | LAMA4 |
| 566 | LAMC3 | **1661** | LAMP1 | **2756** | LAMTOR2 | **3850** | LANCL3 |
| 567 | LAT2 | **1662** | LATS2 | **2757** | LCAT | **3851** | LDAH |
| 568 | LDB2 | **1663** | LDHA | **2758** | LDHAL6A | **3852** | LDHAL6B |
| 569 | LDLRAD3 | **1664** | LEAP2 | **2759** | LENG8 | **3853** | LGALS1 |
| 570 | LGALS3BP | **1665** | LGR4 | **2760** | LGR5 | **3854** | LHFPL6 |
| 571 | LHX3 | **1666** | LIG1 | **2761** | LIG3 | **3855** | LILRB1 |
| 572 | LILRB5 | **1667** | LIMD1 | **2762** | LIN37 | **3856** | LIN9 |
| 573 | LINC00265 | **1668** | LINC00596 | **2763** | LINC00700 | **3857** | LINC00705 |
| 574 | LINC00899 | **1669** | LINC00906 | **2764** | LINC01002 | **3858** | LINC01004 |
| 575 | LINC01141 | **1670** | LINC01165 | **2765** | LINC01312 | **3859** | LINC01551 |
| 576 | LINC01554 | **1671** | LINC01587 | **2766** | LINC02210 | **3860** | LINC02817 |
| 577 | LINC03122 | **1672** | LIPE | **2767** | LMAN1L | **3861** | LMAN2L |
| 578 | LMBR1 | **1673** | LMBRD1 | **2768** | LNPEP | **3862** | LOX |
| 579 | LOXL2 | **1674** | LPAR4 | **2769** | LPIN1 | **3863** | LRBA |
| 580 | LRCH1 | **1675** | LRCH3 | **2770** | LRP11 | **3864** | LRP1B |
| 581 | LRRC18 | **1676** | LRRC2 | **2771** | LRRC28 | **3865** | LRRC40 |
| 582 | LRRC45 | **1677** | LRRC49 | **2772** | LRRC61 | **3866** | LRRC66 |
| 583 | LRRC7 | **1678** | LRRC8A | **2773** | LRRC8D | **3867** | LRRCC1 |
| 584 | LRRD1 | **1679** | LRRIQ1 | **2774** | LRSAM1 | **3868** | LRWD1 |
| 585 | LSM10 | **1680** | LSR | **2775** | LXN | **3869** | LY9 |
| 586 | LYN | **1681** | LYPD6 | **2776** | LYRM9 | **3870** | LYST |
| 587 | LZTR1 | **1682** | M1AP | **2777** | MAB21L3 | **3871** | MAB21L4 |
| 588 | MACROH2A2 | **1683** | MAEL | **2778** | MAF | **3872** | MAFB |
| 589 | MAFF | **1684** | MAFG | **2779** | MAGEC3 | **3873** | MAGEL2 |
| 590 | MAGI1 | **1685** | MAL2 | **2780** | MALRD1 | **3874** | MALT1 |
| 591 | MAML2 | **1686** | MAMSTR | **2781** | MAN1B1 | **3875** | MAN2A2 |
| 592 | MAN2C1 | **1687** | MANEA | **2782** | MAP1LC3A | **3876** | MAP2K6 |
| 593 | MAP3K1 | **1688** | MAP3K20 | **2783** | MAP3K21 | **3877** | MAP4K3 |
| 594 | MAP7D1 | **1689** | MAPKAPK5 | **2784** | MAPRE3 | **3878** | MAT2A |
| 595 | MATN4 | **1690** | MBD2 | **2785** | MBIP | **3879** | MBNL2 |
| 596 | MBOAT1 | **1691** | MCF2 | **2786** | MCF2L2 | **3880** | MCFD2 |
| 597 | MCOLN2 | **1692** | MCRIP2 | **2787** | MCU | **3881** | MDH1 |
| 598 | MDH2 | **1693** | ME3 | **2788** | MED11 | **3882** | MED12L |
| 599 | MED13L | **1694** | MED14 | **2789** | MED26 | **3883** | MED7 |
| 600 | MED9 | **1695** | MEDAG | **2790** | MEF2B | **3884** | MEIS2 |
| 601 | MELK | **1696** | MELT | **2791** | MEP1B | **3885** | MESP2 |
| 602 | METTL15 | **1697** | METTL18 | **2792** | METTL4 | **3886** | METTL8 |
| 603 | MEX3C | **1698** | MGAM | **2793** | MGARP | **3887** | MGAT5 |
| 604 | MGST1 | **1699** | MIA | **2794** | MIA2 | **3888** | MICA |
| 605 | MICAL3 | **1700** | MICU3 | **2795** | MIEF1 | **3889** | MIER1 |
| 606 | MILIP | **1701** | MIPOL1 | **2796** | MIR98 | **3890** | MIR99AHG |
| 607 | MIS18A | **1702** | MITF | **2797** | MKLN1 | **3891** | MKRN1 |
| 608 | MLC1 | **1703** | MLLT10 | **2798** | MLNR | **3892** | MLXIP |
| 609 | MMACHC | **1704** | MMD2 | **2799** | MMP17 | **3893** | MNS1 |
| 610 | MON2 | **1705** | MORC2 | **2800** | MORF4L2 | **3894** | MORN1 |
| 611 | MORN3 | **1706** | MOSPD2 | **2801** | MOV10 | **3895** | MPC1 |
| 612 | MPHOSPH6 | **1707** | MPHOSPH9 | **2802** | MPI | **3896** | MPL |
| 613 | MPP2 | **1708** | MPV17 | **2803** | MPZL2 | **3897** | MRAP2 |
| 614 | MRE11 | **1709** | MRGPRX2 | **2804** | MROH2B | **3898** | MRPL11 |
| 615 | MRPL20 | **1710** | MRPL4 | **2805** | MRPL41 | **3899** | MRPL46 |
| 616 | MRPS11 | **1711** | MRPS16 | **2806** | MRPS18A | **3900** | MRPS18C |
| 617 | MRPS2 | **1712** | MRTFA | **2807** | MRTFB | **3901** | MS4A4A |
| 618 | MS4A5 | **1713** | MSH2 | **2808** | MSL3L2 | **3902** | MSR1 |
| 619 | MT1A | **1714** | MT1B | **2809** | MT1E | **3903** | MT1G |
| 620 | MT1H | **1715** | MT1L | **2810** | MT3 | **3904** | MTERF3 |
| 621 | MTFMT | **1716** | MTFP1 | **2811** | MTFR2 | **3905** | MTM1 |
| 622 | MTMR11 | **1717** | MTRES1 | **2812** | MTRF1L | **3906** | MTX2 |
| 623 | MUC13 | **1718** | MUC5B | **2813** | MUCL3 | **3907** | MUL1 |
| 624 | MVK | **1719** | MVP | **2814** | MX2 | **3908** | MYADML2 |
| 625 | MYBBP1A | **1720** | MYBL1 | **2815** | MYBPC3 | **3909** | MYBPHL |
| 626 | MYDGF | **1721** | MYEF2 | **2816** | MYH10 | **3910** | MYH11 |
| 627 | MYHC | **1722** | MYL10 | **2817** | MYLIP | **3911** | MYLK3 |
| 628 | MYO10 | **1723** | MYO1B | **2818** | MYO1E | **3912** | MYO1F |
| 629 | MYO5B | **1724** | MYO5C | **2819** | MYO6 | **3913** | MYO9A |
| 630 | MYOF | **1725** | MYOG | **2820** | MYPN | **3914** | MYSM1 |
| 631 | NAA15 | **1726** | NACA4P | **2821** | NADK2 | **3915** | NAE1 |
| 632 | NAGA | **1727** | NAGK | **2822** | NAGLU | **3916** | NALF2 |
| 633 | NANOG | **1728** | NAPEPLD | **2823** | NAPG | **3917** | NARS2 |
| 634 | NAT1 | **1729** | NAT10 | **2824** | NAT8L | **3918** | NBAS |
| 635 | NBEAL1 | **1730** | NBN | **2825** | NBR1 | **3919** | NCAPD3 |
| 636 | NCAPH | **1731** | NCF4 | **2826** | NCK1 | **3920** | NCKAP1L |
| 637 | NCOA7 | **1732** | NCR1 | **2827** | NCR3 | **3921** | ND6 |
| 638 | NDC80 | **1733** | NDOR1 | **2828** | NDRG1 | **3922** | NDST2 |
| 639 | NDUFAF1 | **1734** | NDUFB6 | **2829** | NDUFS8 | **3923** | NDUFV2 |
| 640 | NEAT1 | **1735** | NECAB1 | **2830** | NECAP2 | **3924** | NEDD1 |
| 641 | NEDD4 | **1736** | NEDD4L | **2831** | NEDD9 | **3925** | NEIL3 |
| 642 | NEK1 | **1737** | NEK3 | **2832** | NEURL1 | **3926** | NEUROG2 |
| 643 | NEXN-AS1 | **1738** | NFATC3 | **2833** | NFIL3 | **3927** | NFKB2 |
| 644 | NFKBIB | **1739** | NFS1 | **2834** | NFX1 | **3928** | NFYA |
| 645 | NFYB | **1740** | NGLY1 | **2835** | NGRN | **3929** | NIF3L1 |
| 646 | NIN | **1741** | NIT1 | **2836** | NIT2 | **3930** | NKAIN3 |
| 647 | NKX3-2 | **1742** | NLGN1 | **2837** | NLK | **3931** | NLRP13 |
| 648 | NLRP4 | **1743** | NLRP5 | **2838** | NME2 | **3932** | NME3 |
| 649 | NME7 | **1744** | NMI | **2839** | NMNAT1 | **3933** | NOB1 |
| 650 | NOC3L | **1745** | NOCT | **2840** | NOL10 | **3934** | NOLC1 |
| 651 | NOP53 | **1746** | NOTCH1 | **2841** | NOTUM | **3935** | NOVA2 |
| 652 | NOX5 | **1747** | NPB | **2842** | NPEPPS | **3936** | NPM1 |
| 653 | NR2C2AP | **1748** | NR4A1 | **2843** | NR4A3 | **3937** | NRDE2 |
| 654 | NREP | **1749** | NRIP1 | **2844** | NSF | **3938** | NSL1 |
| 655 | NSMAF | **1750** | NSMCE4A | **2845** | NSUN6 | **3939** | NT5C |
| 656 | NT5C1B | **1751** | NUAK1 | **2846** | NUBP1 | **3940** | NUDCD1 |
| 657 | NUDT12 | **1752** | NUDT4 | **2847** | NUF2 | **3941** | NUMA1 |
| 658 | NUMB | **1753** | NUP107 | **2848** | NUP133 | **3942** | NUP160 |
| 659 | NUP210 | **1754** | NUP85 | **2849** | NUP98 | **3943** | NUSAP1 |
| 660 | NUTM2A-AS1 | **1755** | NXF1 | **2850** | NXPE1 | **3944** | NXPH1 |
| 661 | NXPH4 | **1756** | OAS1 | **2851** | OAS2 | **3945** | OAS3 |
| 662 | OBP2A | **1757** | ODAD2 | **2852** | ODF2L | **3946** | ODF4 |
| 663 | ODR4 | **1758** | OGA | **2853** | OGDH | **3947** | OGT |
| 664 | OIP5 | **1759** | OLFML2B | **2854** | OLR1 | **3948** | OMG |
| 665 | ONECUT2 | **1760** | OPHN1 | **2855** | OPN1SW | **3949** | OR11H1 |
| 666 | OR1M1 | **1761** | OR2A25 | **2856** | OR2C1 | **3950** | OR2H1 |
| 667 | OR2K2 | **1762** | OR4C46 | **2857** | OR4Q3 | **3951** | OR51A2 |
| 668 | OR51G1 | **1763** | OR51I1 | **2858** | OR51I2 | **3952** | OR51Q1 |
| 669 | OR5B2 | **1764** | OR6T1 | **2859** | OR8K1 | **3953** | ORAI2 |
| 670 | ORM2 | **1765** | ORMDL2 | **2860** | OSBPL11 | **3954** | OSBPL3 |
| 671 | OSER1 | **1766** | OSGEP | **2861** | OSGEPL1 | **3955** | OSGIN2 |
| 672 | OSTM1 | **1767** | OTOL1 | **2862** | OTOR | **3956** | OTP |
| 673 | OTUD1 | **1768** | OTUD5 | **2863** | OTX2 | **3957** | OVGP1 |
| 674 | OXR1 | **1769** | OXSM | **2864** | P76 | **3958** | PA2G4 |
| 675 | PAAF1 | **1770** | PABPC1P2 | **2865** | PABPC4 | **3959** | PADI6 |
| 676 | PAGR1 | **1771** | PALS2 | **2866** | PAPOLG | **3960** | PARG |
| 677 | PARK7 | **1772** | PARP12 | **2867** | PARP4 | **3961** | PARP6 |
| 678 | PARP8 | **1773** | PARP9 | **2868** | PARVG | **3962** | PATE1 |
| 679 | PATL2 | **1774** | PAX3 | **2869** | PBDC1 | **3963** | PBRM1 |
| 680 | PBX2 | **1775** | PBX4 | **2870** | PCARE | **3964** | PCCA |
| 681 | PCDH10 | **1776** | PCDH11X | **2871** | PCDHA5 | **3965** | PCDHGC4 |
| 682 | PCF11 | **1777** | PCGF2 | **2872** | PCID2 | **3966** | PCK2 |
| 683 | PCNA | **1778** | PCNX1 | **2873** | PCP4L1 | **3967** | PCSK2 |
| 684 | PCSK7 | **1779** | PCSK9 | **2874** | PDC | **3968** | PDE11A |
| 685 | PDE3A | **1780** | PDE7B | **2875** | PDGFC | **3969** | PDGFRL |
| 686 | PDHB | **1781** | PDIA3 | **2876** | PDIA5 | **3970** | PDK1 |
| 687 | PDK3 | **1782** | PDRG1 | **2877** | PDS5B | **3971** | PDXP |
| 688 | PDZD2 | **1783** | PDZD9 | **2878** | PELI1 | **3972** | PEX11B |
| 689 | PEX14 | **1784** | PEX2 | **2879** | PEX3 | **3973** | PEX5L |
| 690 | PFKL | **1785** | PFN1 | **2880** | PGA3 | **3974** | PGCKA1 |
| 691 | PGGHG | **1786** | PGK1 | **2881** | PGK2 | **3975** | PHACTR2 |
| 692 | PHACTR4 | **1787** | PHAF1 | **2882** | PHB1 | **3976** | PHF11 |
| 693 | PHIP | **1788** | PHKG1 | **2883** | PHLDA2 | **3977** | PHLDB1 |
| 694 | PHOSPHO1 | **1789** | PHTF2 | **2884** | PHYHIP | **3978** | PI4KA |
| 695 | PI4KB | **1790** | PIAS1 | **2885** | PIBF1 | **3979** | PICALM |
| 696 | PICK1 | **1791** | PID1 | **2886** | PIDD1 | **3980** | PIEZO1 |
| 697 | PIGF | **1792** | PIGP | **2887** | PIGS | **3981** | PIGV |
| 698 | PIH1D1 | **1793** | PILRB | **2888** | PIM2 | **3982** | PIP4K2A |
| 699 | PKD1L1 | **1794** | PKD1L3 | **2889** | PKD2 | **3983** | PKD2L1 |
| 700 | PKDREJ | **1795** | PKN2 | **2890** | PKP2 | **3984** | PLA2G10 |
| 701 | PLA2G12B | **1796** | PLA2G2F | **2891** | PLAA | **3985** | PLAAT3 |
| 702 | PLAAT5 | **1797** | PLAGL1 | **2892** | PLAUR | **3986** | PLCB4 |
| 703 | PLCG1 | **1798** | PLCH1 | **2893** | PLCL2 | **3987** | PLEK2 |
| 704 | PLEKHA5 | **1799** | PLEKHA6 | **2894** | PLEKHA7 | **3988** | PLEKHG2 |
| 705 | PLGRKT | **1800** | PLIN2 | **2895** | PLIN4 | **3989** | PLK2 |
| 706 | PLPP1 | **1801** | PLPP2 | **2896** | PLPP3 | **3990** | PLPPR2 |
| 707 | PLS3 | **1802** | PLSCR5 | **2897** | PLXNA2 | **3991** | PMAIP1 |
| 708 | PMP2 | **1803** | PMP22 | **2898** | PMS1 | **3992** | PMS2P2 |
| 709 | PMS2P3 | **1804** | PNN | **2899** | PNP | **3993** | PNPT1 |
| 710 | POC1A | **1805** | POGK | **2900** | POLA2 | **3994** | POLE3 |
| 711 | POLI | **1806** | POLR1D | **2901** | POLR1F | **3995** | POLR1HASP |
| 712 | POLR2G | **1807** | POLR2M | **2902** | POLR3B | **3996** | POLR3H |
| 713 | POM121L10P | **1808** | POMC | **2903** | POP1 | **3997** | POP5 |
| 714 | POR | **1809** | POT1 | **2904** | POTEE | **3998** | POTEI |
| 715 | POTEKP | **1810** | POU5F1B | **2905** | PPARA | **3999** | PPARD |
| 716 | PPDPF | **1811** | PPEF2 | **2906** | PPFIBP1 | **4000** | PPHLN1 |
| 717 | PPIA | **1812** | PPIAL4G | **2907** | PPIL2 | **4001** | PPIL4 |
| 718 | PPIP5K2 | **1813** | PPM1E | **2908** | PPME1 | **4002** | PPP1CA |
| 719 | PPP1R18 | **1814** | PPP1R3C | **2909** | PPP2R3C | **4003** | PPP3CA |
| 720 | PPP3CC | **1815** | PPP4C | **2910** | PPP4R4 | **4004** | PPP6R3 |
| 721 | PPRC1 | **1816** | PPWD1 | **2911** | PRAP1 | **4005** | PRB4 |
| 722 | PRDX1 | **1817** | PRDX3 | **2912** | PRDX4 | **4006** | PREB |
| 723 | PRELID2 | **1818** | PREP | **2913** | PRIM1 | **4007** | PRIM2 |
| 724 | PRIMPOL | **1819** | PRKAG3 | **2914** | PRKD3 | **4008** | PRLR |
| 725 | PRMT3 | **1820** | PRO2268 | **2915** | PRODH | **4009** | PROKR1 |
| 726 | PRORP | **1821** | PRPF38B | **2916** | PRR27 | **4010** | PRRC2A |
| 727 | PRSS12 | **1822** | PRSS23 | **2917** | PRUNE1 | **4011** | PRXL2C |
| 728 | PSG3 | **1823** | PSIP1 | **2918** | PSMA1 | **4012** | PSMA4 |
| 729 | PSMA7 | **1824** | PSMB3 | **2919** | PSMC4 | **4013** | PSME3IP1 |
| 730 | PSMG4 | **1825** | PSRC1 | **2920** | PTAFR | **4014** | PTCRA |
| 731 | PTEN | **1826** | PTER | **2921** | PTGDR2 | **4015** | PTGDS |
| 732 | PTGER1 | **1827** | PTGES2-DT | **2922** | PTGIR | **4016** | PTGR1 |
| 733 | PTH | **1828** | PTPN12 | **2923** | PTPN13 | **4017** | PTPN3 |
| 734 | PTPN4 | **1829** | PTPRE | **2924** | PTPRJ | **4018** | PTPRM |
| 735 | PTRH1 | **1830** | PTTG3P | **2925** | PTX3 | **4019** | PUDP |
| 736 | PUS10 | **1831** | PUS7 | **2926** | PUS7L | **4020** | PWP1 |
| 737 | PWWP3A | **1832** | PXMP4 | **2927** | PYM1 | **4021** | PYY |
| 738 | QRSL1 | **1833** | QTGAL | **2928** | RAB11B | **4022** | RAB11FIP5 |
| 739 | RAB12 | **1834** | RAB15 | **2929** | RAB1A | **4023** | RAB27B |
| 740 | RAB28 | **1835** | RAB31 | **2930** | RAB39A | **4024** | RAB3D |
| 741 | RAB3GAP2 | **1836** | RAB3IP | **2931** | RAB44 | **4025** | RAB5A |
| 742 | RAB5C | **1837** | RAB6A | **2932** | RAB9BP1 | **4026** | RABEPK |
| 743 | RABGAP1 | **1838** | RABGAP1L | **2933** | RAC2 | **4027** | RAD23A |
| 744 | RAD50 | **1839** | RAD51 | **2934** | RAD51B | **4028** | RAD51C |
| 745 | RAD9B | **1840** | RAET1G | **2935** | RALGAPA2 | **4029** | RALGAPB |
| 746 | RALGPS2 | **1841** | RAP2C | **2936** | RAPGEF2 | **4030** | RAPGEF6 |
| 747 | RARRES1 | **1842** | RARS1 | **2937** | RASD1 | **4031** | RASGEF1B |
| 748 | RASL11A | **1843** | RASSF4 | **2938** | RASSF8 | **4032** | RBBP4 |
| 749 | RBFOX2 | **1844** | RBFOX3 | **2939** | RBM15B | **4033** | RBM23 |
| 750 | RBM45 | **1845** | RBM48 | **2940** | RBMS1 | **4034** | RBMX |
| 751 | RBP4 | **1846** | RBP7 | **2941** | RBPMS2 | **4035** | RCAN2 |
| 752 | RCAN3 | **1847** | RCL1 | **2942** | RCVRN | **4036** | RD3 |
| 753 | RECK | **1848** | RECQL4 | **2943** | RER1 | **4037** | RETN |
| 754 | REV3L | **1849** | REXO1L1P | **2944** | RFLNA | **4038** | RFT1 |
| 755 | RFX3 | **1850** | RFX6 | **2945** | RFXANK | **4039** | RGMB-AS1 |
| 756 | RGPD5 | **1851** | RGS1 | **2946** | RGS10 | **4040** | RGS18 |
| 757 | RGS2 | **1852** | RGS4 | **2947** | RGS5 | **4041** | RHBDD2 |
| 758 | RHBDL2 | **1853** | RHEX | **2948** | RHOD | **4042** | RHOH |
| 759 | RHOT2 | **1854** | RIC1 | **2949** | RIGI | **4043** | RING1 |
| 760 | RIOK3 | **1855** | RIOX2 | **2950** | RLF | **4044** | RMDN1 |
| 761 | RMND5A | **1856** | RMND5B | **2951** | RNASE10 | **4045** | RNASEH2A |
| 762 | RND1 | **1857** | RNF115 | **2952** | RNF13 | **4046** | RNF139 |
| 763 | RNF14 | **1858** | RNF144B | **2953** | RNF152 | **4047** | RNF170 |
| 764 | RNF180 | **1859** | RNF182 | **2954** | RNF19A | **4048** | RNF19B |
| 765 | RNF212 | **1860** | RNF216 | **2955** | RNF217 | **4049** | RNF26 |
| 766 | RNF34 | **1861** | RNF6 | **2956** | RNFT1 | **4050** | RNGTT |
| 767 | RNH1 | **1862** | RP9 | **2957** | RPAP1 | **4051** | RPGRIP1L |
| 768 | RPL10 | **1863** | RPL10A | **2958** | RPL14 | **4052** | RPL15 |
| 769 | RPL18 | **1864** | RPL18A | **2959** | RPL19 | **4053** | RPL23A |
| 770 | RPL24 | **1865** | RPL27AP6 | **2960** | RPL28 | **4054** | RPL29 |
| 771 | RPL30 | **1866** | RPL34 | **2961** | RPL36 | **4055** | RPL36A |
| 772 | RPL37 | **1867** | RPL39L | **2962** | RPL4 | **4056** | RPL5 |
| 773 | RPL6 | **1868** | RPL7 | **2963** | RPL7A | **4057** | RPN2 |
| 774 | RPP25 | **1869** | RPP25L | **2964** | RPS13 | **4058** | RPS15 |
| 775 | RPS16 | **1870** | RPS2 | **2965** | RPS26 | **4059** | RPS27 |
| 776 | RPS28 | **1871** | RPS4X | **2966** | RPS4Y1 | **4060** | RPS6 |
| 777 | RPS6KA5 | **1872** | RPS6KB1 | **2967** | RPS6KC1 | **4061** | RPSAP15 |
| 778 | RPUSD3 | **1873** | RPUSD4 | **2968** | RRAGA | **4062** | RRH |
| 779 | RRS1 | **1874** | RSPH1 | **2969** | RSPO1 | **4063** | RSPO2 |
| 780 | RSRC2 | **1875** | RTKN2 | **2970** | RTL3 | **4064** | RTN4IP1 |
| 781 | RTRAF | **1876** | RTTN | **2971** | RUFY1 | **4065** | RWDD2A |
| 782 | RXFP1 | **1877** | RXRA | **2972** | S100A11 | **4066** | S100A13 |
| 783 | S100A3 | **1878** | S100A6 | **2973** | S100P | **4067** | S100PBP |
| 784 | SACM1L | **1879** | SAMD13 | **2974** | SAMD9 | **4068** | SANBR |
| 785 | SAP18 | **1880** | SASS6 | **2975** | SAT1 | **4069** | SAT2 |
| 786 | SAYSD1 | **1881** | SBF1 | **2976** | SCAI | **4070** | SCAND3 |
| 787 | SCAPER | **1882** | SCARB1 | **2977** | SCARNA12 | **4071** | SCARNA13 |
| 788 | SCLY | **1883** | SCML1 | **2978** | SCN2A | **4072** | SCNN1B |
| 789 | SCRIB | **1884** | SCRN3 | **2979** | SCRT1 | **4073** | SCX |
| 790 | SCYL3 | **1885** | SDC2 | **2980** | SDHAF2 | **4074** | SDR42E1 |
| 791 | SEC13 | **1886** | SEC14L1 | **2981** | SEC22A | **4075** | SECISBP2 |
| 792 | SEH1L | **1887** | SELENON | **2982** | SELENOP | **4076** | SEMA6D |
| 793 | SENP7 | **1888** | SEPHS2 | **2983** | SERAC1 | **4077** | SERBP1 |
| 794 | SERGEF | **1889** | SERINC4 | **2984** | SERPINA3 | **4078** | SERPINA4 |
| 795 | SERPINB1 | **1890** | SERPINB10 | **2985** | SERPINB2 | **4079** | SERPINB9 |
| 796 | SERPINC1 | **1891** | SERTAD1 | **2986** | SERTAD2 | **4080** | SERTAD3 |
| 797 | SESTD1 | **1892** | SF3B4 | **2987** | SFMBT1 | **4081** | SFN |
| 798 | SFPQ | **1893** | SFRS16 | **2988** | SFTA2 | **4082** | SGK1 |
| 799 | SGMS1 | **1894** | SGO2 | **2989** | SGSM2 | **4083** | SH2D4A |
| 800 | SH2D4B | **1895** | SH2D7 | **2990** | SH3KBP1 | **4084** | SHLD2 |
| 801 | SHMT1 | **1896** | SHOC2 | **2991** | SIGIRR | **4085** | SIGLEC1 |
| 802 | SIK1 | **1897** | SIM1 | **2992** | SIN3B | **4086** | SIPA1L2 |
| 803 | SIRT7 | **1898** | SIVA1 | **2993** | SIX4 | **4087** | SKIC8 |
| 804 | SKP2 | **1899** | SLAMF8 | **2994** | SLC10A6 | **4088** | SLC10A7 |
| 805 | SLC11A2 | **1900** | SLC16A13 | **2995** | SLC16A3 | **4089** | SLC17A5 |
| 806 | SLC17A8 | **1901** | SLC17A9 | **2996** | SLC18A2 | **4090** | SLC22A11 |
| 807 | SLC22A13 | **1902** | SLC22A20 | **2997** | SLC22A5 | **4091** | SLC25A12 |
| 808 | SLC25A13 | **1903** | SLC25A20 | **2998** | SLC25A36 | **4092** | SLC25A46 |
| 809 | SLC26A2 | **1904** | SLC27A2 | **2999** | SLC27A4 | **4093** | SLC2A10 |
| 810 | SLC2A2 | **1905** | SLC2A6 | **3000** | SLC30A3 | **4094** | SLC30A4 |
| 811 | SLC33A1 | **1906** | SLC35A1 | **3001** | SLC35A5 | **4095** | SLC35B2 |
| 812 | SLC35D2 | **1907** | SLC35D4 | **3002** | SLC35E3 | **4096** | SLC35F3 |
| 813 | SLC35F5 | **1908** | SLC35G1 | **3003** | SLC38A4 | **4097** | SLC38A6 |
| 814 | SLC38A9 | **1909** | SLC39A1 | **3004** | SLC39A10 | **4098** | SLC39A4 |
| 815 | SLC3A2 | **1910** | SLC41A2 | **3005** | SLC43A3 | **4099** | SLC45A2 |
| 816 | SLC46A3 | **1911** | SLC4A7 | **3006** | SLC5A3 | **4100** | SLC5A6 |
| 817 | SLC66A1LP | **1912** | SLC67A1-AS | **3007** | SLC6A12 | **4101** | SLC6A15 |
| 818 | SLC6A20 | **1913** | SLC6A6 | **3008** | SLC6A7 | **4102** | SLC6A8 |
| 819 | SLC7A11 | **1914** | SLC7A6OS | **3009** | SLCO1B1 | **4103** | SLCO2B1 |
| 820 | SLCO3A1 | **1915** | SLFN12L | **3010** | SLIT1 | **4104** | SLK |
| 821 | SLMAP | **1916** | SLX4IP | **3011** | SMAD1 | **4105** | SMAD6 |
| 822 | SMARCA1 | **1917** | SMARCA2 | **3012** | SMARCAD1 | **4106** | SMARCB1 |
| 823 | SMC2 | **1918** | SMC6 | **3013** | SMCO4 | **4107** | SMIM10L2A |
| 824 | SMIM10L2B | **1919** | SMIM23 | **3014** | SMIM8 | **4108** | SMOX |
| 825 | SMPD4 | **1920** | SNAP29 | **3015** | SNAPIN | **4109** | SNHG1 |
| 826 | SNHG12 | **1921** | SNHG29 | **3016** | SNIP1 | **4110** | SNORA1 |
| 827 | SNORA14B | **1922** | SNORA29 | **3017** | SNORA2A | **4111** | SNORA33 |
| 828 | SNORA3B | **1923** | SNORA41 | **3018** | SNORA54 | **4112** | SNORA55 |
| 829 | SNORA58 | **1924** | SNORA5A | **3019** | SNORA5C | **4113** | SNORA6 |
| 830 | SNORA69 | **1925** | SNORA70B | **3020** | SNORA74A | **4114** | SNORA75 |
| 831 | SNORD12C | **1926** | SNORD13P2 | **3021** | SNORD1A | **4115** | SNORD20 |
| 832 | SNORD28 | **1927** | SNORD30 | **3022** | SNORD38A | **4116** | SNORD38B |
| 833 | SNORD42A | **1928** | SNORD42B | **3023** | SNORD43 | **4117** | SNORD45C |
| 834 | SNORD4A | **1929** | SNORD4B | **3024** | SNORD50B | **4118** | SNORD53 |
| 835 | SNORD54 | **1930** | SNORD6 | **3025** | SNORD63 | **4119** | SNORD73A |
| 836 | SNORD77 | **1931** | SNORD95 | **3026** | SNORD96A | **4120** | SNRNP40 |
| 837 | SNRPD2P2 | **1932** | SNRPD3 | **3027** | SNUPN | **4121** | SNURF |
| 838 | SNX13 | **1933** | SNX24 | **3028** | SNX25 | **4122** | SNX29 |
| 839 | SNX4 | **1934** | SNX8 | **3029** | SNX9 | **4123** | SOBP |
| 840 | SOS2 | **1935** | SOSTDC1 | **3030** | SOWAHA | **4124** | SOWAHB |
| 841 | SOWAHC | **1936** | SOX15 | **3031** | SOX18 | **4125** | SOX3 |
| 842 | SOX30 | **1937** | SP100 | **3032** | SP3 | **4126** | SP7 |
| 843 | SPACDR | **1938** | SPAG16 | **3033** | SPANXA2-OT1 | **4127** | SPARC |
| 844 | SPAST | **1939** | SPATA31G1 | **3034** | SPATA42 | **4128** | SPC24 |
| 845 | SPCS3 | **1940** | SPDL1 | **3035** | SPDYA | **4129** | SPDYC |
| 846 | SPDYE1 | **1941** | SPG11 | **3036** | SPINK2 | **4130** | SPIRE1 |
| 847 | SPMIP10 | **1942** | SPMIP6 | **3037** | SPP1 | **4131** | SPRED2 |
| 848 | SPRR1B | **1943** | SPRYD3 | **3038** | SPTLC2 | **4132** | SPTY2D1 |
| 849 | SPX | **1944** | SQOR | **3039** | SRBD1 | **4133** | SRGAP2 |
| 850 | SRI | **1945** | SRPK2 | **3040** | SRRM1 | **4134** | SRRM2-AS1 |
| 851 | SRSF2 | **1946** | SRSF6 | **3041** | SRXN1 | **4135** | SSBP2 |
| 852 | SSBP3 | **1947** | SSBP3-AS1 | **3042** | SSBP4 | **4136** | SSC4D |
| 853 | SSH1 | **1948** | SSH2 | **3043** | SSRP1 | **4137** | SST |
| 854 | SSU72 | **1949** | ST6GALNAC2 | **3044** | ST7 | **4138** | ST8SIA6 |
| 855 | STAB1 | **1950** | STAC2 | **3045** | STAG1 | **4139** | STAG2 |
| 856 | STAM2 | **1951** | STAMBPL1 | **3046** | STAP2 | **4140** | STARD13 |
| 857 | STAT2 | **1952** | STAT5B | **3047** | STAT6 | **4141** | STAU2 |
| 858 | STC2 | **1953** | STEAP1B | **3048** | STIL | **4142** | STK10 |
| 859 | STK11 | **1954** | STK17A | **3049** | STK3 | **4143** | STK31 |
| 860 | STK32B | **1955** | STK36 | **3050** | STK38 | **4144** | STK38L |
| 861 | STK4 | **1956** | STK40 | **3051** | STMN1 | **4145** | STPG1 |
| 862 | STPG4 | **1957** | STRADA | **3052** | STRADB | **4146** | STRN |
| 863 | STX11 | **1958** | STX17 | **3053** | STX4 | **4147** | STX7 |
| 864 | STXBP3 | **1959** | STXBP5 | **3054** | SUCLA2 | **4148** | SULT1A1 |
| 865 | SULT1A4 | **1960** | SUMO3 | **3055** | SUMO4 | **4149** | SUOX |
| 866 | SUPT3H | **1961** | SUSD1 | **3056** | SUZ12P1 | **4150** | SVBP |
| 867 | SVOPL | **1962** | SWT1 | **3057** | SYCE2 | **4151** | SYCP2 |
| 868 | SYCP3 | **1963** | SYF2 | **3058** | SYNC | **4152** | SYNDIG1L |
| 869 | SYNGR3 | **1964** | SYNJ2 | **3059** | SYNPO | **4153** | SYT14 |
| 870 | SYTL4 | **1965** | SZRD1 | **3060** | TAAR5 | **4154** | TAC4 |
| 871 | TACSTD2 | **1966** | TADA2A | **3061** | TAF1B | **4155** | TAF1D |
| 872 | TAF5L | **1967** | TAGLN | **3062** | TANC1 | **4156** | TANGO6 |
| 873 | TANK | **1968** | TAOK3 | **3063** | TAPBP | **4157** | TARS2 |
| 874 | TAS2R4 | **1969** | TAS2R50 | **3064** | TASOR | **4158** | TASP1 |
| 875 | TBC1D10A | **1970** | TBC1D12 | **3065** | TBC1D19 | **4159** | TBC1D2 |
| 876 | TBC1D9B | **1971** | TBL1XR1 | **3066** | TBR1 | **4160** | TBX18 |
| 877 | TBX3 | **1972** | TCAF1 | **3067** | TCAIM | **4161** | TCEAL8 |
| 878 | TCEANC2 | **1973** | TCF15 | **3068** | TCF4 | **4162** | TCF7 |
| 879 | TCP11L1 | **1974** | TCP11L2 | **3069** | TDGF1 | **4163** | TDP1 |
| 880 | TDRD3 | **1975** | TDRD7 | **3070** | TEC | **4164** | TECR |
| 881 | TECTA | **1976** | TEF | **3071** | TENM1 | **4165** | TERF2IP |
| 882 | TES | **1977** | TET2 | **3072** | TEX13A | **4166** | TEX15 |
| 883 | TEX2 | **1978** | TEX29 | **3073** | TEX41 | **4167** | TFAM |
| 884 | TFB1M | **1979** | TFCP2 | **3074** | TFDP2 | **4168** | TGFA |
| 885 | TGFBI | **1980** | TGFBR1 | **3075** | TGFBR2 | **4169** | TGFBR3 |
| 886 | TGIF1 | **1981** | THAP2 | **3076** | THAP5 | **4170** | THOC2 |
| 887 | THSD1P1 | **1982** | THYN1 | **3077** | TIA1 | **4171** | TIAL1 |
| 888 | TIAM1 | **1983** | TIGD2 | **3078** | TIMMDC1 | **4172** | TIMP1 |
| 889 | TIMP3 | **1984** | TINF2 | **3079** | TJP2 | **4173** | TK1 |
| 890 | TK2 | **1985** | TLCD1 | **3080** | TLCD4 | **4174** | TLE1 |
| 891 | TLE4 | **1986** | TLL2 | **3081** | TM4SF1 | **4175** | TM7SF2 |
| 892 | TMCC1 | **1987** | TMED7-TICAM2 | **3082** | TMEM11 | **4176** | TMEM117 |
| 893 | TMEM119 | **1988** | TMEM126A | **3083** | TMEM131L | **4177** | TMEM132E |
| 894 | TMEM135 | **1989** | TMEM138 | **3084** | TMEM14A | **4178** | TMEM150B |
| 895 | TMEM150C | **1990** | TMEM151A | **3085** | TMEM154 | **4179** | TMEM164 |
| 896 | TMEM167 | **1991** | TMEM171 | **3086** | TMEM177 | **4180** | TMEM178B |
| 897 | TMEM19 | **1992** | TMEM198B | **3087** | TMEM201 | **4181** | TMEM204 |
| 898 | TMEM240 | **1993** | TMEM254 | **3088** | TMEM267 | **4182** | TMEM37 |
| 899 | TMEM40 | **1994** | TMEM45B | **3089** | TMEM62 | **4183** | TMEM67 |
| 900 | TMEM97 | **1995** | TMPRSS7 | **3090** | TMTC1 | **4184** | TMX4 |
| 901 | TNFAIP2 | **1996** | TNFAIP8 | **3091** | TNFRSF10A | **4185** | TNFRSF10B |
| 902 | TNFRSF10D | **1997** | TNFRSF11A | **3092** | TNFRSF12A | **4186** | TNFRSF19 |
| 903 | TNFRSF1A | **1998** | TNFRSF1B | **3093** | TNFRSF9 | **4187** | TNIK |
| 904 | TNRC6B | **1999** | TNRC6C | **3094** | TNS3 | **4188** | TNXB |
| 905 | TOB1 | **2000** | TOGARAM2 | **3095** | TOMM20 | **4189** | TOP2B |
| 906 | TOR1AIP1 | **2001** | TOR3A | **3096** | TOX | **4190** | TP53AIP1 |
| 907 | TP53BP1 | **2002** | TP53TG3 | **3097** | TPD52L1 | **4191** | TPD52L3 |
| 908 | TPP1 | **2003** | TPR | **3098** | TPST1 | **4192** | TPST2 |
| 909 | TPTEP1 | **2004** | TRAF3IP2 | **3099** | TRAF3IP3 | **4193** | TRAF5 |
| 910 | TRAM2 | **2005** | TRAPPC11 | **3100** | TRAPPC12 | **4194** | TRAPPC2L |
| 911 | TRAPPC3 | **2006** | TRDMT1 | **3101** | TREM2 | **4195** | TREX2 |
| 912 | TRIB1 | **2007** | TRIL | **3102** | TRIM13 | **4196** | TRIM21 |
| 913 | TRIM22 | **2008** | TRIM24 | **3103** | TRIM28 | **4197** | TRIM31 |
| 914 | TRIM35 | **2009** | TRIM36 | **3104** | TRIM37 | **4198** | TRIM44 |
| 915 | TRIM46 | **2010** | TRIM47 | **3105** | TRIM5 | **4199** | TRIM55 |
| 916 | TRIM59 | **2011** | TRIM63 | **3106** | TRIM72 | **4200** | TRIOBP |
| 917 | TRIP13 | **2012** | TRIQK | **3107** | TRMT10A | **4201** | TRMT2B |
| 918 | TRPC4 | **2013** | TRPC4AP | **3108** | TRPC6 | **4202** | TRPM6 |
| 919 | TRPV5 | **2014** | TSBP1 | **3109** | TSC22D1-AS1 | **4203** | TSC22D2 |
| 920 | TSG101 | **2015** | TSPAN13 | **3110** | TSPAN18 | **4204** | TSPAN5 |
| 921 | TSPAN6 | **2016** | TSPO2 | **3111** | TSR1 | **4205** | TSR2 |
| 922 | TTC22 | **2017** | TTC27 | **3112** | TTC35 | **4206** | TTC39C |
| 923 | TTC41P | **2018** | TTC8 | **3113** | TTLL1 | **4207** | TTPAL |
| 924 | TUBA1C | **2019** | TUBAL3 | **3114** | TUBB6 | **4208** | TUBB8B |
| 925 | TUBD1 | **2020** | TUBGCP5 | **3115** | TUBGCP6 | **4209** | TUFM |
| 926 | TUSC5 | **2021** | TWNK | **3116** | TXLNA | **4210** | TXNL1 |
| 927 | TXNL4B | **2022** | TXNRD3 | **3117** | TYRP1 | **4211** | UACA |
| 928 | UBA1 | **2023** | UBA3 | **3118** | UBALD2 | **4212** | UBE2A |
| 929 | UBE2D4 | **2024** | UBE2L6 | **3119** | UBE2Q2 | **4213** | UBE2Z |
| 930 | UBFD1 | **2025** | UBQLN2 | **3120** | UBQLN4 | **4214** | UBR1 |
| 931 | UBR2 | **2026** | UBR3 | **3121** | UBTF | **4215** | UBXN1 |
| 932 | UCP1 | **2027** | UCP2 | **3122** | UCP3 | **4216** | UFL1 |
| 933 | UGCG | **2028** | UGDH | **3123** | UGT1A1 | **4217** | UGT1A9 |
| 934 | ULK3 | **2029** | UMODL1 | **3124** | UNC13C | **4218** | UNC50 |
| 935 | UNC5A | **2030** | UPF3B | **3125** | UPRT | **4219** | UQCC2 |
| 936 | UQCRB | **2031** | UQCRC1 | **3126** | USF3 | **4220** | USP13 |
| 937 | USP24 | **2032** | USP25 | **3127** | USP32 | **4221** | USP34 |
| 938 | USP36 | **2033** | USP5 | **3128** | USP6NL | **4222** | USP9X |
| 939 | UTP14A | **2034** | UTP3 | **3129** | UTP6 | **4223** | UTS2 |
| 940 | UVRAG | **2035** | VAC14 | **3130** | VAMP8 | **4224** | VANGL1 |
| 941 | VAV2 | **2036** | VCL | **3131** | VCPKMT | **4225** | VCX2 |
| 942 | VDAC2 | **2037** | VIL1 | **3132** | VKORC1L1 | **4226** | VMA12 |
| 943 | VMA22 | **2038** | VN1R3 | **3133** | VNN2 | **4227** | VPS13A |
| 944 | VPS13B | **2039** | VPS13C | **3134** | VPS16 | **4228** | VPS18 |
| 945 | VPS25 | **2040** | VPS33B | **3135** | VPS36 | **4229** | VPS50 |
| 946 | VPS54 | **2041** | VPS8 | **3136** | VPS9D1-AS1 | **4230** | VRK1 |
| 947 | VRK2 | **2042** | VSIG2 | **3137** | VSNL1 | **4231** | VSX2 |
| 948 | VTN | **2043** | VWA5A | **3138** | VWA5B1 | **4232** | VWC2 |
| 949 | WARS1 | **2044** | WARS2 | **3139** | WASF2 | **4233** | WASHC1 |
| 950 | WASHC4 | **2045** | WASHC5 | **3140** | WBP4 | **4234** | WDFY2 |
| 951 | WDFY3-AS2 | **2046** | WDR12 | **3141** | WDR19 | **4235** | WDR25 |
| 952 | WDR38 | **2047** | WDR4 | **3142** | WDR44 | **4236** | WDR7 |
| 953 | WDR70 | **2048** | WDR82 | **3143** | WDR86 | **4237** | WDR87 |
| 954 | WHAMMP3 | **2049** | WNT16 | **3144** | WNT4 | **4238** | WNT6 |
| 955 | WNT7A | **2050** | WT1 | **3145** | WWP1 | **4239** | XAF1 |
| 956 | XCR1 | **2051** | XPO1 | **3146** | XPO6 | **4240** | XPR1 |
| 957 | XRCC5 | **2052** | XYLB | **3147** | XYLT2 | **4241** | YARS1 |
| 958 | YBX1P2 | **2053** | YEATS4 | **3148** | YIPF4 | **4242** | YPEL1 |
| 959 | YRDC | **2054** | YTHDC1 | **3149** | YTHDC2 | **4243** | YTHDF1 |
| 960 | ZAR1 | **2055** | ZAR1L | **3150** | ZBED10P | **4244** | ZBTB22 |
| 961 | ZBTB38 | **2056** | ZC2HC1A | **3151** | ZCCHC18 | **4245** | ZCCHC3 |
| 962 | ZCCHC9 | **2057** | ZCWPW2 | **3152** | ZDHHC13 | **4246** | ZDHHC16 |
| 963 | ZFAND2B | **2058** | ZFAS1 | **3153** | ZFYVE16 | **4247** | ZFYVE19 |
| 964 | ZFYVE26 | **2059** | ZFYVE28 | **3154** | ZHX3 | **4248** | ZIK1 |
| 965 | ZKSCAN1 | **2060** | ZMIZ1 | **3155** | ZMIZ2 | **4249** | ZMYM3 |
| 966 | ZMYM4 | **2061** | ZMYND8 | **3156** | ZNF12 | **4250** | ZNF14 |
| 967 | ZNF154 | **2062** | ZNF160 | **3157** | ZNF217 | **4251** | ZNF229 |
| 968 | ZNF252P | **2063** | ZNF266 | **3158** | ZNF280D | **4252** | ZNF287 |
| 969 | ZNF296 | **2064** | ZNF302 | **3159** | ZNF32 | **4253** | ZNF343 |
| 970 | ZNF354A | **2065** | ZNF354B | **3160** | ZNF367 | **4254** | ZNF37A |
| 971 | ZNF382 | **2066** | ZNF385C | **3161** | ZNF408 | **4255** | ZNF419 |
| 972 | ZNF423 | **2067** | ZNF429 | **3162** | ZNF431 | **4256** | ZNF438 |
| 973 | ZNF473 | **2068** | ZNF485 | **3163** | ZNF512B | **4257** | ZNF518A |
| 974 | ZNF529 | **2069** | ZNF534 | **3164** | ZNF551 | **4258** | ZNF556 |
| 975 | ZNF567 | **2070** | ZNF57 | **3165** | ZNF572 | **4259** | ZNF574 |
| 976 | ZNF583 | **2071** | ZNF593 | **3166** | ZNF608 | **4260** | ZNF609 |
| 977 | ZNF622 | **2072** | ZNF623 | **3167** | ZNF624 | **4261** | ZNF641 |
| 978 | ZNF658 | **2073** | ZNF671 | **3168** | ZNF678 | **4262** | ZNF681 |
| 979 | ZNF691 | **2074** | ZNF705A | **3169** | ZNF71 | **4263** | ZNF74 |
| 980 | ZNF77 | **2075** | ZNF770 | **3170** | ZNF791 | **4264** | ZNF827 |
| 981 | ZNF830 | **2076** | ZNF85 | **3171** | ZP2 | **4265** | ZRANB3 |
| 982 | ZRSR2P1 | **2077** | ZSCAN5C | **3172** | ZYG11B | **4266** | ZYX |
| 983 | LTB4R | **2078** | METAP2 | **3173** | TGM2 | **4267** | RXRB |
| 984 | PTGER4 | **2079** | PTGER3 | **3174** | ANPEP | **4268** | FAAH |
| 985 | Mme | **2080** | CYP2C9 | **3175** | UL80 | **4269** | TRPA1 |
| 986 | PAM | **2081** | CYP26B1 | **3176** | CTBP2 | **4270** | ESRRG |
| 987 | G6PC | **2082** | SLC37A4 | **3177** | ABAT | **4271** | GNG2 |
| 988 | GNB1 | **2083** | GPR183 | **3178** | HDAC10 | **4272** | HDAC11 |
| 989 | HDAC7 | **2084** | HDAC9 | **3179** | LPAR2 | **4273** | MB |
| 990 | NQO2 | **2085** | PTPN11 | **3180** | RBBP9 | **4274** | RHOA |
| 991 | SLC12A2 | **2086** | ALDH5A1 | **3181** | TRPM2 | **4275** | MIR17 |
| 992 | STAR | **2087** | EIF2AK3 | **3182** | EIF2S1 | **4276** | IL17A |
| 993 | MAPK15 | **2088** | MIR21 | **3183** | SULT2A1 | **4277** | ADRB1 |
| 994 | CALR | **2089** | CLDN1 | **3184** | CXCR4 | **4278** | IL22 |
| 995 | MIR20B | **2090** | OCLN | **3185** | TJP1 | **4279** | UGT1A3 |
| 996 | ACADVL | **2091** | ACO2 | **3186** | ACOT2 | **4280** | ACPH-1 |
| 997 | ACSL1B | **2092** | ACSS1 | **3187** | ACT-2 | **4281** | ACTL6A |
| 998 | AFP4 | **2093** | AHNAK | **3188** | AK1 | **4282** | ALAD |
| 999 | ALDH18A1 | **2094** | ALDH9A1A.1 | **3189** | ALDH9A1B | **4283** | ALDOAA |
| 1000 | ALDOB | **2095** | AND1 | **3190** | AND2 | **4284** | ANPEPB.1 |
| 1001 | ANXA13L | **2096** | ANXA1A | **3191** | ANXA1C | **4285** | ANXA6 |
| 1002 | APOA2 | **2097** | APOBB.1 | **3192** | APODA.2 | **4286** | APOEA |
| 1003 | APOEB | **2098** | ARHGEF19 | **3193** | ARHGEF2 | **4287** | ARL3 |
| 1004 | ATG7 | **2099** | ATIC | **3194** | ATP1B3A | **4288** | ATP2A1L |
| 1005 | ATP5F1B | **2100** | ATP5L | **3195** | ATP5PO | **4289** | B0285.3 |
| 1006 | B0391.14 | **2101** | B0511.11 | **3196** | BCL2L2 | **4290** | BCL9L |
| 1007 | C18E3.1 | **2102** | C24H12.8 | **3197** | C48D1.9 | **4291** | CALB2A |
| 1008 | CALB2B | **2103** | CALM1A | **3198** | CANT1B | **4292** | CAP1 |
| 1009 | CAPNS1A | **2104** | CASKIN1 | **3199** | CCT2 | **4293** | CCT5 |
| 1010 | CHIA.1 | **2105** | CKMT1 | **3200** | CKMT2B | **4294** | CLPXA |
| 1011 | COL1A2 | **2106** | COL2A1A | **3201** | COPE | **4295** | COPG2 |
| 1012 | CPA5 | **2107** | CPB1 | **3202** | CPT1AB | **4296** | CRKL |
| 1013 | CRYAA | **2108** | CRYGN2 | **3203** | CSNK1A1 | **4297** | CTGF |
| 1014 | CTH | **2109** | CTPS1A | **3204** | CTRL | **4298** | CYCSB |
| 1015 | CYP11A1 | **2110** | CYP6AS2 | **3205** | CYP6AS5 | **4299** | DAO.1 |
| 1016 | DDP-1 | **2111** | DDX39AB | **3206** | DES | **4300** | DHHC-3 |
| 1017 | DHTKD1 | **2112** | DPYDB | **3207** | DPYSL5A | **4301** | DYNC1H1 |
| 1018 | EEF2B | **2113** | EIF3I | **3208** | EIF3S10 | **4302** | EIF4BB |
| 1019 | EIF4EBP1 | **2114** | EIF5B | **3209** | ELA3L | **4303** | EMC10 |
| 1020 | ERLIN1 | **2115** | ERP44 | **3210** | ETF1B | **4304** | ETFA |
| 1021 | F22E5.20 | **2116** | FABP | **3211** | FABP1B.1 | **4305** | FABP3 |
| 1022 | FABP6 | **2117** | FAHD2A | **3212** | FAM53B | **4306** | FAM78A |
| 1023 | FARSB | **2118** | FBN3 | **3213** | FDX1 | **4307** | FGF21 |
| 1024 | FGG | **2119** | FOXO6 | **3214** | FSHB | **4308** | FUBP1 |
| 1025 | FUBP3 | **2120** | GAMT | **3215** | GART | **4309** | GCH2 |
| 1026 | GLULB | **2121** | GMPS | **3216** | GNAT2 | **4310** | GNB3B |
| 1027 | GRAMD2A | **2122** | GSTA.1 | **3217** | GSTP2 | **4311** | HADH |
| 1028 | HADHAB | **2123** | HAO1 | **3218** | HBAE1.1 | **4312** | HBBE2 |
| 1029 | HCFC1A | **2124** | HLH-30 | **3219** | HNRNPA0A | **4313** | IDH3B |
| 1030 | IGF2BP1 | **2125** | IL11RA1 | **3220** | IL11RA2 | **4314** | IL13RA1 |
| 1031 | IL17B | **2126** | IL17RB | **3221** | IL18RAP | **4315** | IL1F10 |
| 1032 | IL20 | **2127** | IL20RB | **3222** | IL22RA1 | **4316** | IL27 |
| 1033 | IL33 | **2128** | IL36A | **3223** | IL36B | **4317** | IL36G |
| 1034 | IL36RN | **2129** | IL9R | **3224** | IMPDH1B | **4318** | IRAK2 |
| 1035 | KRT15 | **2130** | KRT94 | **3225** | LEMD3 | **4319** | LHB |
| 1036 | LMNA | **2131** | LMTK3 | **3226** | LPO | **4320** | LZTS1 |
| 1037 | MCM4 | **2132** | MICAL1 | **3227** | MIR101A | **4321** | MIR10A |
| 1038 | MIR181C | **2133** | MIR200A | **3228** | MIR214 | **4322** | MIR25 |
| 1039 | MIR27B | **2134** | MIR29C | **3229** | MIR30A | **4323** | MIR324 |
| 1040 | MIR339 | **2135** | MIR34A | **3230** | MIR351 | **4324** | MIR450A1 |
| 1041 | MIR667 | **2136** | MIR687 | **3231** | MIR760 | **4325** | MPHOSPH8 |
| 1042 | MRPS-15 | **2137** | MTA2 | **3232** | MTDHA | **4326** | MTHFD1A |
| 1043 | MTHFD1L | **2138** | MTHFD2 | **3233** | MYBPHB | **4327** | MYH11A |
| 1044 | MYL1 | **2139** | MYOZ1B | **3234** | NAXE | **4328** | NCAPG |
| 1045 | NDUFA2 | **2140** | NDUFS8A | **3235** | NDUFV1 | **4329** | NEXN |
| 1046 | NME2B.2 | **2141** | NOL4L | **3236** | NOP56 | **4330** | NOP58 |
| 1047 | NUDT21 | **2142** | OLA1 | **3237** | OPN1MW1 | **4331** | P2RY6 |
| 1048 | PABPC1A | **2143** | PADI2 | **3238** | PAICS | **4332** | PFAS |
| 1049 | PFKLA | **2144** | PGLS | **3239** | PHPT1 | **4333** | POSTNB |
| 1050 | PPARB | **2145** | PPIAB | **3240** | PQN-59 | **4334** | PRDX5 |
| 1051 | PRDX6 | **2146** | PRPS1A | **3241** | PRR5 | **4335** | PRRT2 |
| 1052 | PSAT1 | **2147** | PSMB4 | **3242** | PTBP1A | **4336** | PVALB2 |
| 1053 | PVALB8 | **2148** | R04A9.7 | **3243** | RACK1 | **4337** | RBM4.3 |
| 1054 | RCVRN3 | **2149** | RDH5 | **3244** | RNF145 | **4338** | RPA3 |
| 1055 | RPL11 | **2150** | RPL21 | **3245** | RPL32 | **4339** | RPLP0 |
| 1056 | RPS10 | **2151** | RPS12 | **3246** | RPS19 | **4340** | RPS21 |
| 1057 | RPS8A | **2152** | RPSA | **3247** | RRM1 | **4341** | RTCB |
| 1058 | SAE1 | **2153** | SCP2B | **3248** | SDHB | **4342** | SDR16C5B |
| 1059 | SEMA7A | **2154** | SESN2 | **3249** | SETD3 | **4343** | SLC1A4 |
| 1060 | SLMAPA | **2155** | SMARCA5 | **3250** | SMYHC1 | **4344** | SNAI2 |
| 1061 | SNCB | **2156** | SNCGA | **3251** | SND1 | **4345** | SNED1 |
| 1062 | SNRNP200 | **2157** | SRCIN1 | **3252** | SRSF1A | **4346** | SSP-9 |
| 1063 | STARD4 | **2158** | STOML2 | **3253** | STRA6 | **4347** | SULT1ST1 |
| 1064 | SUPT6H | **2159** | SYNJ1 | **3254** | T02G5.15 | **4348** | T16H12.2 |
| 1065 | T21C12.4 | **2160** | TBXA2R | **3255** | TFA | **4349** | TKTB |
| 1066 | TMOD4 | **2161** | TNNC1B | **3256** | TNNC2 | **4350** | TNNT3B |
| 1067 | TOP1A | **2162** | TPI1B | **3257** | TPMA | **4351** | TRIB3 |
| 1068 | TYRP1B | **2163** | U2AF2B | **3258** | UBAP2B | **4352** | UBAP2L |
| 1069 | UCHL5 | **2164** | UGP2A | **3259** | UGT1AB | **4353** | UGT2B17 |
| 1070 | UMPS | **2165** | UNC45B | **3260** | UOX | **4354** | UPB1 |
| 1071 | UQCRC2B | **2166** | USP9 | **3261** | VCP | **4355** | VPS35 |
| 1072 | VTG1 | **2167** | VWA11 | **3262** | Y39B6A.25 | **4356** | Y57G11C.8 |
| 1073 | Y94H6A.10 | **2168** | YBX1 | **3263** | YWHAZ | **4357** | PTPRF |
| 1074 | ACP1 | **2169** | VDR | **3264** | ALOX5AP | **4358** | CYSLTR1 |
| 1075 | ALOX12 | **2170** | PTGDR | **3265** | EDNRA | **4359** | FABP1 |
| 1076 | MMP10 | **2171** | TYMS | **3266** | TRPM8 | **4360** | ITGB1 |
| 1077 | PTPN6 | **2172** | SLC29A1 | **3267** | GBA | **4361** | ADK |
| 1078 | ADORA2B | **2173** | GAPDH | **3268** | GRK1 | **4362** | TYMP |
| 1079 | HK1 | **2174** | MANBA | **3269** | CDA | **4363** | DAO |
| 1080 | IMPDH1 | **2175** | IMPDH2 | **3270** | ALDH1A2 | **4364** | ALDH1B1 |
| 1081 | AMY2A | **2176** | GJB2 | **3271** | LGALS3 | **4365** | LGALS4 |
| 1082 | LGALS9 | **2177** | LGALS7 | **3272** | LGALS8 | **4366** | SELL |
| 1083 | P2RY4 | **2178** | P2RY14 | **3273** | NRAS | **4367** | SLC28A3 |
| 1084 | SLC5A4 | **2179** | SLC5A11 | **3274** | TNNI3 | **4368** | TNNC1 |
| 1085 | TNNT2 | **2180** | LGALS7B | **3275** | SCN5A | **4369** | GRM2 |
| 1086 | CXCR2 | **2181** | HRH2 | **3276** | PDE7A | **4370** | KCNA5 |
| 1087 | CASR | **2182** | SLC9A1 | **3277** | CACNA1H | **4371** | Soat1 |
| 1088 | SLC6A9 | **2183** | CYP2D6 | **3278** | DPP7 | **4372** | PDE4A |
| 1089 | DGAT1 | **2184** | CPB2 | **3279** | CPA1 | **4373** | SLC13A5 |
| 1090 | GRIK1 | **2185** | EGLN3 | **3280** | SLC15A1 | **4374** | PARP2 |
| 1091 | CPN1 | **2186** | PARP15 | **3281** | PARP10 | **4375** | WDR5 |
| 1092 | PDE9A | **2187** | KDM6B | **3282** | KDM4A | **4376** | KDM4D |
| 1093 | TAS1R3 | **2188** | RPA1 | **3283** | TAS1R1 | **4377** | DBH |
| 1094 | FABP5 | **2189** | FABP7 | **3284** | GRM3 | **4378** | GRM6 |
| 1095 | EPHX1 | **2190** | KDM1A |  |  |  |  |

**Table S3** **The characteristic targets of R-IRI from GeneCards**

| NO | Target | NO | Target | NO | Target | NO | Target |
| --- | --- | --- | --- | --- | --- | --- | --- |
| 1 | ACE | **256** | HNF1B | **511** | VHL | **765** | PAX2 |
| 2 | SLC5A2 | **257** | MTOR | **512** | AGT | **766** | IL6 |
| 3 | TNF | **258** | REN | **513** | SLC22A12 | **767** | PTEN |
| 4 | WT1 | **259** | UMOD | **514** | AGTR1 | **768** | LCN2 |
| 5 | TP53 | **260** | TGFB1 | **515** | TSC2 | **769** | EGF |
| 6 | VEGFA | **261** | H19 | **516** | NLRP3 | **770** | TUG1 |
| 7 | FGF23 | **262** | HIF1A | **517** | EGFR | **771** | BDNF-AS |
| 8 | HAVCR1 | **263** | SDHB | **518** | HMOX1 | **772** | IL10 |
| 9 | MALAT1 | **264** | SLC2A9 | **519** | ALB | **773** | TLR4 |
| 10 | CFH | **265** | ATM | **520** | STAT3 | **774** | CST3 |
| 11 | PVT1 | **266** | CASR | **521** | C3 | **775** | NFE2L2 |
| 12 | CASP3 | **267** | CCL2 | **522** | MUC1 | **776** | THBD |
| 13 | IFNG | **268** | CTNNB1 | **523** | AKT1 | **777** | EPO |
| 14 | COL4A5 | **269** | SETD2 | **524** | NEAT1 | **778** | MIR21 |
| 15 | IL1B | **270** | CRP | **525** | INS | **779** | SOD1 |
| 16 | GDNF | **271** | SOD2-OT1 | **526** | CXCL8 | **780** | TRPC6 |
| 17 | SMARCA4 | **272** | CTLA4 | **527** | SLC17A5 | **781** | MYH9 |
| 18 | BMP4 | **273** | NPPA | **528** | PTGS2 | **782** | APOA1 |
| 19 | ICAM1 | **274** | KCNJ1 | **529** | MME | **783** | CD46 |
| 20 | GAS5 | **275** | FRAS1 | **530** | SDHD | **784** | CDKN2A |
| 21 | TERT | **276** | LRP2 | **531** | XDH | **785** | ADAMTS13 |
| 22 | ACTN4 | **277** | CDKN1B | **532** | HMGB1 | **786** | MEG3 |
| 23 | FOXP3 | **278** | SALL1 | **533** | LAMB2 | **787** | ITGAM |
| 24 | NOS3 | **279** | CERNA3 | **534** | PGR-AS1 | **788** | MMP9 |
| 25 | BCL2 | **280** | IGF1 | **535** | S100B | **789** | SIRT1 |
| 26 | KIT | **281** | TFEB | **536** | CCND1 | **790** | ABCB1 |
| 27 | MPO | **282** | IL2 | **537** | MAPK1 | **791** | OGG1 |
| 28 | NOTCH2 | **283** | COL4A1 | **538** | ACE2 | **792** | NAGLU |
| 29 | CD274 | **284** | KL | **539** | PIK3CA | **793** | NOS2 |
| 30 | MIR494 | **285** | FN1 | **540** | SERPINE1 | **794** | WNT4 |
| 31 | SMAD4 | **286** | F2 | **541** | BRCA2 | **795** | IL18 |
| 32 | EDN1 | **287** | HSPA5 | **542** | CXCR4 | **796** | VWF |
| 33 | CFB | **288** | BAX | **543** | MYC | **797** | PARK7 |
| 34 | GFAP | **289** | CCN2 | **544** | BRCA1 | **798** | SLC2A1 |
| 35 | CUBN | **290** | MMP2 | **545** | JAK2 | **799** | CBS |
| 36 | NF2 | **291** | GPT | **546** | SLC5A1 | **800** | SELP |
| 37 | RUNX1 | **292** | CLU | **547** | JAG1 | **801** | NPPB |
| 38 | STK11 | **293** | ADIPOQ | **548** | TRIM8 | **802** | FGA |
| 39 | FASLG | **294** | PARP1 | **549** | CFTR | **803** | SIX2 |
| 40 | NOTCH1 | **295** | SPP1 | **550** | IL1RN | **804** | SNHG14 |
| 41 | BMP7 | **296** | ENO2 | **551** | FABP1 | **805** | VDR |
| 42 | CALCA | **297** | GSK3B | **552** | TRAP1 | **806** | IL4 |
| 43 | KDR | **298** | PDGFRB | **553** | AQP1 | **807** | MTHFR |
| 44 | YAP1 | **299** | TIMP2 | **554** | HGF | **808** | MIR126 |
| 45 | GJA1 | **300** | NOX4 | **555** | CXCL10 | **809** | MIR145 |
| 46 | PPARG | **301** | FOXC1 | **556** | CXCL12 | **810** | HOTAIR |
| 47 | FLT1 | **302** | TLR2 | **557** | CYP3A4 | **811** | STAT1 |
| 48 | MIR210 | **303** | PIK3CG | **558** | MIR320A | **812** | NR3C2 |
| 49 | KCNQ1OT1 | **304** | CAT | **559** | SNHG12 | **813** | SERPINA1 |
| 50 | FAS | **305** | SMAD3 | **560** | MAPK8 | **814** | MB |
| 51 | SNHG1 | **306** | LEP | **561** | CDKN2B-AS1 | **815** | NTRK1 |
| 52 | ABCC2 | **307** | BDNF | **562** | IL1A | **816** | ABCC8 |
| 53 | HSPB1 | **308** | EGLN3 | **563** | SMAD2 | **817** | F5 |
| 54 | KEAP1 | **309** | SOX9 | **564** | SHH | **818** | CAV1 |
| 55 | MAPT | **310** | MIAT | **565** | BMP2 | **819** | ELANE |
| 56 | DNM1L | **311** | SERPINC1 | **566** | TIMP1 | **820** | XIST |
| 57 | LINC02605 | **312** | F3 | **567** | SLC22A6 | **821** | FGFR2 |
| 58 | TNFRSF1A | **313** | ABCG2 | **568** | COMT | **822** | ROBO1 |
| 59 | SOD2 | **314** | ACTA2 | **569** | SELE | **823** | PTPRO |
| 60 | MIR125A | **315** | MIR17 | **570** | BIRC5 | **824** | NOD2 |
| 61 | TF | **316** | FGF2 | **571** | VCAM1 | **825** | SGK1 |
| 62 | CUL3 | **317** | CASP9 | **572** | PLG | **826** | PIK3C2A |
| 63 | KMT2D | **318** | EPAS1 | **573** | AGER | **827** | RELA |
| 64 | EIF2AK3 | **319** | FOXC2 | **574** | MAPK14 | **828** | DNASE1 |
| 65 | MAP2K1 | **320** | MIR155 | **575** | MAPK3 | **829** | HAMP |
| 66 | MIR122 | **321** | HBEGF | **576** | BRD4 | **830** | ITGB2 |
| 67 | CD40LG | **322** | NGF | **577** | IGF1R | **831** | MIR29A |
| 68 | CDKN1A | **323** | FOXO3 | **578** | HSPG2 | **832** | DNMT1 |
| 69 | KNG1 | **324** | MDM2 | **579** | FTO | **833** | CASP8 |
| 70 | CASP5 | **325** | PCSK9 | **580** | PON1 | **834** | GDF15 |
| 71 | SLIT2 | **326** | G6PC1 | **581** | CRNDE | **835** | CD44 |
| 72 | SCARB1 | **327** | SLC22A2 | **582** | CCR2 | **836** | AGTR2 |
| 73 | MIR150 | **328** | EGR1 | **583** | LGALS3 | **837** | MIR532 |
| 74 | MUTYH | **329** | NFKB1 | **584** | AKT3 | **838** | APP |
| 75 | NFKBIA | **330** | DCDC2 | **585** | MIF | **839** | AIFM1 |
| 76 | EZH2 | **331** | WNT5A | **586** | PRKAG2 | **840** | GCK |
| 77 | CPT2 | **332** | KCNJ11 | **587** | NOS1 | **841** | CYBB |
| 78 | AMBP | **333** | PDGFB | **588** | MECP2 | **842** | MIR15A |
| 79 | ADM | **334** | JUN | **589** | MYD88 | **843** | ACTB |
| 80 | MIR106B | **335** | BCL2L1 | **590** | CCL5 | **844** | GFER |
| 81 | DHCR7 | **336** | YRDC | **591** | DDIT3 | **845** | MIR214 |
| 82 | ZEB2 | **337** | ESR1 | **592** | ATP7B | **846** | PTGS1 |
| 83 | CASP4 | **338** | ALDH1A2 | **593** | DPP4 | **847** | TNFSF10 |
| 84 | TGFB2 | **339** | MIR141 | **594** | MIR27A | **848** | SQSTM1 |
| 85 | ZFAS1 | **340** | IL17A | **595** | MBL2 | **849** | RHOA |
| 86 | CD36 | **341** | LMNA | **596** | SIRT3 | **850** | C5 |
| 87 | FOXO1 | **342** | GAPDH | **597** | THBS1 | **851** | PPARGC1A |
| 88 | CXCR3 | **343** | SNHG6 | **598** | CYCS | **852** | ZNF609 |
| 89 | MIR133B | **344** | PTPRC | **599** | CDC42 | **853** | PTPN11 |
| 90 | SNHG7 | **345** | TGFBR1 | **600** | APOB | **854** | PCNA |
| 91 | SNCA | **346** | CD40 | **601** | NTN1 | **855** | BECN1 |
| 92 | ADAM17 | **347** | PLA2G6 | **602** | MMP3 | **856** | AQP4 |
| 93 | FLNA | **348** | C5AR1 | **603** | KISS1 | **857** | SOCS1 |
| 94 | ALDH2 | **349** | MIR146B | **604** | FGF7 | **858** | HAVCR2 |
| 95 | APLN | **350** | ADA | **605** | SLC1A1 | **859** | SRC |
| 96 | OCLN | **351** | BMP6 | **606** | MAP4K4 | **860** | GSTP1 |
| 97 | PCAT1 | **352** | XIAP | **607** | VTN | **861** | HP |
| 98 | COL1A1 | **353** | MIR146A | **608** | SDC1 | **862** | DNMT3A |
| 99 | GGT1 | **354** | ANGPT1 | **609** | METTL3 | **863** | UCP2 |
| 100 | ITCH | **355** | MIR142 | **610** | JAK1 | **864** | ITPR1 |
| 101 | IL33 | **356** | CHI3L1 | **611** | CP | **865** | KLF4 |
| 102 | MIR30A | **357** | SELL | **612** | SLPI | **866** | ADCY10 |
| 103 | FOS | **358** | MIR34A | **613** | FGD5-AS1 | **867** | MAFB |
| 104 | MIR140 | **359** | HSPA4 | **614** | CXCL1 | **868** | TRAF3 |
| 105 | MIR211 | **360** | RAC1 | **615** | HCP5 | **869** | SLC6A19 |
| 106 | TNFAIP3 | **361** | TJP1 | **616** | FGB | **870** | PPARA |
| 107 | STING1 | **362** | CDK6 | **617** | TRA-TGC7-1 | **871** | CXCL2 |
| 108 | CCR5 | **363** | SOCS3 | **618** | MIR191 | **872** | PTHLH |
| 109 | TNFRSF11B | **364** | PTK2 | **619** | CREB1 | **873** | FMR1 |
| 110 | G6PD | **365** | NEU1 | **620** | NQO1 | **874** | RUNX2 |
| 111 | ANGPT2 | **366** | PROM1 | **621** | FTX | **875** | TNFRSF1B |
| 112 | HDAC4 | **367** | PGK1 | **622** | PDPN | **876** | ELAVL1 |
| 113 | GHRL | **368** | C4B | **623** | CD55 | **877** | TLR7 |
| 114 | TNXB | **369** | IRF1 | **624** | YY1 | **878** | F10 |
| 115 | RIPK3 | **370** | AHSG | **625** | PLAUR | **879** | CD59 |
| 116 | PLAT | **371** | CYP2C9 | **626** | MIR106A | **880** | SLC9A1 |
| 117 | FLT3 | **372** | MIR424 | **627** | TYROBP | **881** | GZMB |
| 118 | MIR221 | **373** | MASP1 | **628** | ECE1 | **882** | CRBN |
| 119 | COL3A1 | **374** | F8 | **629** | MIR342 | **883** | SEMA3A |
| 120 | CYP1B1 | **375** | ATF3 | **630** | MIR378A | **884** | CCL3 |
| 121 | LINC01234 | **376** | TRPV1 | **631** | IL13 | **885** | HSPA1A |
| 122 | PRKN | **377** | CSF3 | **632** | PECAM1 | **886** | PWAR1 |
| 123 | IDH1 | **378** | MIRLET7C | **633** | TXNIP | **887** | KRT18 |
| 124 | TNNT2 | **379** | IRAK1 | **634** | GLUD1 | **888** | AXL |
| 125 | SLC7A11 | **380** | CD47 | **635** | MIR144 | **889** | HIF1A-AS2 |
| 126 | JAK3 | **381** | MT-CYB | **636** | HSPD1 | **890** | TRIM28 |
| 127 | MIR199A1 | **382** | GNA11 | **637** | SLC6A4 | **891** | TXN |
| 128 | IRF5 | **383** | MIR193A | **638** | DUSP6 | **892** | PRKDC |
| 129 | LPL | **384** | CSF1 | **639** | NORAD | **893** | MAOA |
| 130 | GRIN2B | **385** | MIR4435-2HG | **640** | CAMK2B | **894** | GP1BA |
| 131 | CSF2 | **386** | CTSD | **641** | IL15 | **895** | FNDC5 |
| 132 | FASN | **387** | DKK1 | **642** | AKR1B1 | **896** | UCA1 |
| 133 | ALOX5 | **388** | LOX | **643** | RIPK1 | **897** | GSTM1 |
| 134 | CPT1A | **389** | KDM1A | **644** | STIM1 | **898** | TNNI3 |
| 135 | CYBA | **390** | GUSB | **645** | LAMP2 | **899** | PSEN1 |
| 136 | TRPV4 | **391** | NPY | **646** | COL4A2 | **900** | CR1 |
| 137 | MIR34C | **392** | AIF1 | **647** | TET2 | **901** | SNHG4 |
| 138 | FOXM1 | **393** | TLR3 | **648** | COL18A1 | **902** | MIRLET7B |
| 139 | MIR93 | **394** | NEDD4L | **649** | TBK1 | **903** | USP7 |
| 140 | HSP90AA1 | **395** | EDNRB | **650** | SCGB1A1 | **904** | PIK3CB |
| 141 | BSG | **396** | PRKCD | **651** | SLC12A2 | **905** | HDAC1 |
| 142 | GLP1R | **397** | GJB1 | **652** | AKT2 | **906** | MIR9-1 |
| 143 | IL1R1 | **398** | PLAU | **653** | IL1RL1 | **907** | TNFSF12 |
| 144 | FABP7 | **399** | GSR | **654** | SLC22A8 | **908** | SERPING1 |
| 145 | KCNJ5 | **400** | COLEC11 | **655** | TRPS1 | **909** | CKB |
| 146 | MIR22 | **401** | IL7 | **656** | TRPM7 | **910** | GRIN1 |
| 147 | CXCL9 | **402** | ABCA1 | **657** | CBL | **911** | CYTOR |
| 148 | ITGB1 | **403** | STUB1 | **658** | NR4A1 | **912** | GPX3 |
| 149 | SLC1A2 | **404** | HDAC6 | **659** | TKT | **913** | TTN |
| 150 | C1S | **405** | MASP2 | **660** | RYR1 | **914** | GLUL |
| 151 | MIR19A | **406** | MIR98 | **661** | BNIP3 | **915** | HMGCR |
| 152 | ORAI1 | **407** | PRKCA | **662** | HTRA2 | **916** | VEGFC |
| 153 | MIR335 | **408** | NGFR | **663** | MIR23A | **917** | GCG |
| 154 | SMPD1 | **409** | TLR9 | **664** | PINK1 | **918** | DAPK1 |
| 155 | MANF | **410** | CASP7 | **665** | LINC-ROR | **919** | SNHG16 |
| 156 | S100A9 | **411** | CDKN3 | **666** | CLDN5 | **920** | FGF17 |
| 157 | MIR192 | **412** | CX3CL1 | **667** | DCN | **921** | NES |
| 158 | SNAI1 | **413** | NBAS | **668** | CALR | **922** | CLDN7 |
| 159 | MFN2 | **414** | KCNQ1 | **669** | ROCK1 | **923** | MAP1LC3A |
| 160 | ATF4 | **415** | SGPP1 | **670** | SLC6A3 | **924** | GATA4 |
| 161 | MIR486-1 | **416** | TIMP3 | **671** | CFP | **925** | IRS1 |
| 162 | SHC1 | **417** | CRYAB | **672** | KLK1 | **926** | GSDMD |
| 163 | SPTAN1 | **418** | CXCL5 | **673** | ERN1 | **927** | MIR451A |
| 164 | ADNP | **419** | CDK1 | **674** | MMP14 | **928** | FABP3 |
| 165 | EPOR | **420** | MIR7-3HG | **675** | FABP2 | **929** | SREBF1 |
| 166 | FLRT3 | **421** | IRF3 | **676** | CD34 | **930** | LEF1-AS1 |
| 167 | CFLAR | **422** | PSAP | **677** | HSPA8 | **931** | MIR20B |
| 168 | C3AR1 | **423** | RTN4 | **678** | VCP | **932** | KCNMA1 |
| 169 | CASP1 | **424** | TGIF1 | **679** | SMAD7 | **933** | CPOX |
| 170 | CTSB | **425** | FBXW7 | **680** | ATP7A | **934** | CLDN1 |
| 171 | MIR216A | **426** | NDUFS4 | **681** | DRD2 | **935** | MIR204 |
| 172 | NT5E | **427** | YY1AP1 | **682** | MCL1 | **936** | KLF11 |
| 173 | HDAC9 | **428** | ADORA2A | **683** | GRN | **937** | NR4A2 |
| 174 | IDO1 | **429** | ATF6 | **684** | MIR24-1 | **938** | NEUROD1 |
| 175 | GPNMB | **430** | AIM2 | **685** | CD14 | **939** | MIR18A |
| 176 | MIR25 | **431** | MIR15B | **686** | CHAT | **940** | FGF21 |
| 177 | MERTK | **432** | FGF1 | **687** | OIP5-AS1 | **941** | LAMB1 |
| 178 | IGF2BP2 | **433** | S100A8 | **688** | FADD | **942** | ERBB4 |
| 179 | IKBKB | **434** | UGT1A1 | **689** | ABCC9 | **943** | PTK2B |
| 180 | MLKL | **435** | MIR30B | **690** | EPHA2 | **944** | PCK1 |
| 181 | ABL1 | **436** | ANXA5 | **691** | DDX3X | **945** | AHR |
| 182 | MAP3K5 | **437** | APEX1 | **692** | MIR29B1 | **946** | IL11 |
| 183 | ATP5F1A | **438** | CDON | **693** | ENTPD1 | **947** | VLDLR |
| 184 | ANXA2 | **439** | SLC25A4 | **694** | SNORD15A | **948** | DIABLO |
| 185 | SOD3 | **440** | SOS1 | **695** | RPTOR | **949** | CX3CR1 |
| 186 | E2F1 | **441** | CXCL16 | **696** | NR3C1 | **950** | CACNA1C |
| 187 | ADGRG1 | **442** | ERCC2 | **697** | COL7A1 | **951** | PTX3 |
| 188 | MIR377 | **443** | ADAMTS9 | **698** | EPRS1 | **952** | MDH2 |
| 189 | ATR | **444** | TNFRSF10B | **699** | TRIM72 | **953** | CDH5 |
| 190 | LRRK2 | **445** | MRE11 | **700** | ABCB7 | **954** | BAK1 |
| 191 | SLTM | **446** | FCGR3B | **701** | WAC | **955** | DIAPH1 |
| 192 | CXCR2 | **447** | CHKA | **702** | TSPO | **956** | FUS |
| 193 | RMRP | **448** | MIR200A | **703** | RELN | **957** | LAMA2 |
| 194 | MIR124-1 | **449** | MIR132 | **704** | SIRT6 | **958** | SCARNA5 |
| 195 | P2RX7 | **450** | PDCD4 | **705** | F2R | **959** | ACSL4 |
| 196 | MGAT3-AS1 | **451** | CYP2E1 | **706** | FOXP2 | **960** | APAF1 |
| 197 | MIR92A1 | **452** | COX5A | **707** | PIK3R1 | **961** | ITGA2B |
| 198 | ATG7 | **453** | CIITA | **708** | ADAM10 | **962** | NR1I2 |
| 199 | IL22 | **454** | GPX1 | **709** | GPX4 | **963** | WNT3A |
| 200 | ADRB2 | **455** | MIR328 | **710** | SLC1A3 | **964** | PRDX1 |
| 201 | PTGDS | **456** | MIR125B1 | **711** | ULK1 | **965** | NID1 |
| 202 | TP63 | **457** | MAP3K7 | **712** | AOC3 | **966** | SOX4 |
| 203 | TEK | **458** | ETS1 | **713** | VDAC1 | **967** | BSCL2 |
| 204 | SPARC | **459** | BGN | **714** | CKM | **968** | LIF |
| 205 | MIR381 | **460** | MCOLN1 | **715** | ANXA1 | **969** | LGALS1 |
| 206 | S1PR1 | **461** | ADM2 | **716** | MIR139 | **970** | LTF |
| 207 | IFNB1 | **462** | ALOX12 | **717** | ALDH5A1 | **971** | LRP6 |
| 208 | IL7R | **463** | MCAM | **718** | ENO1 | **972** | NRG1 |
| 209 | MIR10A | **464** | PIAS1 | **719** | SESN2 | **973** | HCRT |
| 210 | PRKCB | **465** | GLS | **720** | EPHB2 | **974** | SNAP25 |
| 211 | IL6ST | **466** | WTAP | **721** | TAB2 | **975** | VCAN |
| 212 | CORIN | **467** | STAT4 | **722** | MCU | **976** | SYK |
| 213 | XBP1 | **468** | IL3 | **723** | GAD1 | **977** | SIRT2 |
| 214 | TNC | **469** | MIR222 | **724** | IL37 | **978** | IGFBP7 |
| 215 | PXN | **470** | INTU | **725** | HTR2A | **979** | DAG1 |
| 216 | TET3 | **471** | ADCYAP1 | **726** | TRAF6 | **980** | LONP1 |
| 217 | LIMK1 | **472** | IKZF1 | **727** | CRH | **981** | SCO1 |
| 218 | GPBAR1 | **473** | MIR149 | **728** | TREM1 | **982** | PPIG |
| 219 | NEFL | **474** | NOL3 | **729** | DKK3 | **983** | OPA1 |
| 220 | CCR7 | **475** | PRKAA1 | **730** | BBC3 | **984** | CCK |
| 221 | DYSF | **476** | CD9 | **731** | ACVR2B | **985** | CD81 |
| 222 | DLG4 | **477** | EIF4EBP1 | **732** | GDF11 | **986** | IKBKG |
| 223 | ADORA1 | **478** | IRAK4 | **733** | P2RY12 | **987** | CLCN3 |
| 224 | LRG1 | **479** | TNFRSF12A | **734** | BTG2 | **988** | CYP2J2 |
| 225 | NOX1 | **480** | C1QBP | **735** | GRK4 | **989** | REG3A |
| 226 | SERPINA3 | **481** | PPM1D | **736** | TYK2 | **990** | HULC |
| 227 | ATG5 | **482** | MT-ND3 | **737** | PROCR | **991** | NPPC |
| 228 | AGRN | **483** | ZC3H12A | **738** | SMN1 | **992** | CHRM3 |
| 229 | RAP1B | **484** | HAX1 | **739** | ANGPTL4 | **993** | C1R |
| 230 | CCR6 | **485** | PRKCZ | **740** | MIRLET7F2 | **994** | MIRLET7A1 |
| 231 | STAT5B | **486** | CD80 | **741** | METTL14 | **995** | CLEC7A |
| 232 | MPL | **487** | MIR183 | **742** | IBSP | **996** | OXSR1 |
| 233 | ABAT | **488** | EIF4E | **743** | SLCO2A1 | **997** | GRIN2A |
| 234 | GADD45A | **489** | SERPINF1 | **744** | UTS2 | **998** | H2AX |
| 235 | GAL | **490** | IL12A | **745** | COPA | **999** | EGLN1 |
| 236 | ZAP70 | **491** | ROCK2 | **746** | SULT1A3 | **1000** | ATP2A2 |
| 237 | CACNA1D | **492** | BCHE | **747** | TMEM126B | **1001** | IL1RAPL2 |
| 238 | CXCL13 | **493** | MIR101-1 | **748** | FGF4 | **1002** | S1PR2 |
| 239 | GAST | **494** | COL6A1 | **749** | MIR590 | **1003** | CHUK |
| 240 | TNFRSF10A | **495** | ETHE1 | **750** | MIR195 | **1004** | RYR2 |
| 241 | KCNA2 | **496** | YBX1 | **751** | NCF1 | **1005** | SEMA7A |
| 242 | MIR485 | **497** | PLOD2 | **752** | IL17RA | **1006** | MIR130B |
| 243 | LTBP4 | **498** | LAMA1 | **753** | GRK2 | **1007** | SH2B3 |
| 244 | CTSG | **499** | FGF19 | **754** | PFKFB3 | **1008** | MIR30C1 |
| 245 | BAD | **500** | PLA2G7 | **755** | HOXA11 | **1009** | YWHAG |
| 246 | MAP1LC3B | **501** | RACK1 | **756** | MIR215 | **1010** | SLC40A1 |
| 247 | PWAR4 | **502** | IL5 | **757** | VSIG4 | **1011** | ADIPOR1 |
| 248 | MIR133A1 | **503** | SCN5A | **758** | KIF3B | **1012** | CHMP2B |
| 249 | BCKDK | **504** | MIR143 | **759** | SRSF1 | **1013** | TNIP1 |
| 250 | MIR483 | **505** | MSTN | **760** | APLNR | **1014** | ANO1 |
| 251 | ANK3 | **506** | TAC1 | **761** | TRE-TTC3-1 | **1015** | MIR188 |
| 252 | PRDM16 | **507** | MIR501 | **762** | MIR181A2 | **1016** | PKD1L1 |
| 253 | KCNN4 | **508** | MT-ND6 | **763** | SNX10 | **1017** | SARM1 |
| 254 | KITLG | **509** | PNPT1 | **764** | TRAF2 | **1018** | SIRPA |
| 255 | MYBPC3 | **510** | TGM2 |  |  |  |  |

**Table S4 The characteristic targets of R-IRI from OMIM**

| NO | Target | NO | Target | NO | Target | NO | Target |
| --- | --- | --- | --- | --- | --- | --- | --- |
| **1** | BDKRB1 | **44** | BDKRB2 | **87** | FOXO3 | **130** | YRDC |
| **2** | TLR4 | **45** | ADIPOQ | **88** | G6PD | **131** | RNF182 |
| **3** | TMBIM6 | **46** | EPO | **89** | HAVCR1 | **132** | BCL2L14 |
| **4** | MIR496 | **47** | FGB | **90** | PRKCE | **133** | HNF1B |
| **5** | HSF1 | **48** | CD300LB | **91** | THBS1 | **134** | PAPRS |
| **6** | RHDA1 | **49** | TET2 | **92** | ZNRF1 | **135** | NINJ1 |
| **7** | TMEM123 | **50** | NINJ2 | **93** | HLRCC | **136** | HOMG2 |
| **8** | ARCS1 | **51** | HOMG3 | **94** | DRTA2 | **137** | RTD |
| **9** | PRTAO | **52** | TNFAIP8L2 | **95** | RAPGEF3 | **138** | ECHDC2 |
| **10** | CAMK2A | **53** | LCN2 | **96** | STIM1 | **139** | HDRS |
| **11** | DRTA1 | **54** | MTDPS8A | **97** | DRTA4 | **140** | HOMG4 |
| **12** | AI1G | **55** | RHUC1 | **98** | HOMG5 | **141** | EPM4 |
| **13** | SLSN1 | **56** | STAR | **99** | RCC | **142** | GLYS |
| **14** | MARCH | **57** | NFSRA | **100** | RFH1 | **143** | AABT |
| **15** | DPEP1 | **58** | RNF139 | **101** | MIF | **144** | SLC4A3 |
| **16** | PRKAA1 | **59** | DHCR7 | **102** | SHC3 | **145** | PARP1 |
| **17** | PPIF | **60** | ZNF667 | **103** | HTRA2 | **146** | TXNRD2 |
| **18** | PARP16 | **61** | LPHDST | **104** | AMYLD2 | **147** | TBS1 |
| **19** | FRTS1 | **62** | RCAD | **105** | HLTRS | **148** | XRN |
| **20** | RHUC2 | **63** | MVCD2 | **106** | MVCD3 | **149** | TSC2 |
| **21** | ARCS2 | **64** | CAKUT1 | **107** | HUPRAS | **150** | HOMG6 |
| **22** | NPHS5 | **65** | CMM8 | **108** | SHDRA | **151** | RENI |
| **23** | VCRL1 | **66** | VCRL2 | **109** | RHDA3 | **152** | NEDMCR |
| **24** | MFRG | **67** | RHPD2 | **110** | RHDA2 | **153** | WEDAS |
| **25** | CYSRD | **68** | RVCLS | **111** | RHPD1 | **154** | CDSP |
| **26** | FBS | **69** | BARTS4A | **112** | DRTA3 | **155** | FSGS10 |
| **27** | OPTB3 | **70** | SRTD9 | **113** | PRLMNS | **156** | MKS7 |
| **28** | RCDFRD | **71** | SLOS | **114** | MVCD1 | **157** | RCCP1 |
| **29** | RCCX1 | **72** | GTPTS | **115** | CAKUT2 | **158** | BNAR |
| **30** | VCRL3 | **73** | VCTERL | **116** | NECRC | **159** | BRENS |
| **31** | RHDA4 | **74** | HOMG7 | **117** | BMRS | **160** | DEDCRF |
| **32** | TKCR | **75** | MURCS | **118** | KLK1 | **161** | ADRA2C |
| **33** | PRCC | **76** | SLC17A1 | **119** | SLC34A1 | **162** | SLC5A2 |
| **34** | KCNJ1 | **77** | CEP83 | **120** | C4ORF46 | **163** | SLC14A2 |
| **35** | NPHS1 | **78** | SLC49A4 | **121** | MOK | **164** | DIRC1 |
| **36** | SFMBT1 | **79** | DIRC3 | **122** | FAM107A | **165** | NOX4 |
| **37** | GTPBP4 | **80** | PKD1 | **123** | TGFB1 | **166** | EGFR |
| **38** | WT1 | **81** | AGTR1 | **124** | DACH1 | **167** | TLR2 |
| **39** | C3 | **82** | PTGS2 | **125** | AQP4 | **168** | TNF |
| **40** | FGFR1 | **83** | TNFRSF1A | **126** | TNFRSF1B | **169** | MTOR |
| **41** | TMIGD1 | **84** | CCND1 | **127** | SOSTDC1 | **170** | IL10 |
| **42** | CXCR4 | **85** | SOCS3 | **128** | FGA | **171** | MET |
| **43** | ALGS1 | **86** | IL1A | **129** | TTC36 | **172** | APOE |

**Table S5 Characteristic targets intersection of R-IRI from GeneCards and OMIM after deduplication**

| NO | Targets | NO | Targets | NO | Targets | NO | Targets |
| --- | --- | --- | --- | --- | --- | --- | --- |
| 1 | ACE | **299** | HNF1B | **597** | VHL | **894** | PAX2 |
| 2 | SLC5A2 | **300** | MTOR | **598** | AGT | **895** | IL6 |
| 3 | TNF | **301** | REN | **599** | SLC22A12 | **896** | PTEN |
| 4 | WT1 | **302** | UMOD | **600** | AGTR1 | **897** | LCN2 |
| 5 | TP53 | **303** | TGFB1 | **601** | TSC2 | **898** | EGF |
| 6 | VEGFA | **304** | H19 | **602** | NLRP3 | **899** | TUG1 |
| 7 | FGF23 | **305** | HIF1A | **603** | EGFR | **900** | BDNF-AS |
| 8 | HAVCR1 | **306** | SDHB | **604** | HMOX1 | **901** | IL10 |
| 9 | MALAT1 | **307** | SLC2A9 | **605** | ALB | **902** | TLR4 |
| 10 | CFH | **308** | ATM | **606** | STAT3 | **903** | CST3 |
| 11 | PVT1 | **309** | CASR | **607** | C3 | **904** | NFE2L2 |
| 12 | CASP3 | **310** | CCL2 | **608** | MUC1 | **905** | THBD |
| 13 | IFNG | **311** | CTNNB1 | **609** | AKT1 | **906** | EPO |
| 14 | COL4A5 | **312** | SETD2 | **610** | NEAT1 | **907** | MIR21 |
| 15 | IL1B | **313** | CRP | **611** | INS | **908** | SOD1 |
| 16 | GDNF | **314** | SOD2-OT1 | **612** | CXCL8 | **909** | TRPC6 |
| 17 | SMARCA4 | **315** | CTLA4 | **613** | SLC17A5 | **910** | MYH9 |
| 18 | BMP4 | **316** | NPPA | **614** | PTGS2 | **911** | APOA1 |
| 19 | ICAM1 | **317** | KCNJ1 | **615** | MME | **912** | CD46 |
| 20 | GAS5 | **318** | FRAS1 | **616** | SDHD | **913** | CDKN2A |
| 21 | TERT | **319** | LRP2 | **617** | XDH | **914** | ADAMTS13 |
| 22 | ACTN4 | **320** | CDKN1B | **618** | HMGB1 | **915** | MEG3 |
| 23 | FOXP3 | **321** | SALL1 | **619** | LAMB2 | **916** | ITGAM |
| 24 | NOS3 | **322** | CERNA3 | **620** | PGR-AS1 | **917** | MMP9 |
| 25 | BCL2 | **323** | IGF1 | **621** | S100B | **918** | SIRT1 |
| 26 | KIT | **324** | TFEB | **622** | CCND1 | **919** | ABCB1 |
| 27 | MPO | **325** | IL2 | **623** | MAPK1 | **920** | OGG1 |
| 28 | NOTCH2 | **326** | COL4A1 | **624** | ACE2 | **921** | NAGLU |
| 29 | CD274 | **327** | KL | **625** | PIK3CA | **922** | NOS2 |
| 30 | MIR494 | **328** | FN1 | **626** | SERPINE1 | **923** | WNT4 |
| 31 | SMAD4 | **329** | F2 | **627** | BRCA2 | **924** | IL18 |
| 32 | EDN1 | **330** | HSPA5 | **628** | CXCR4 | **925** | VWF |
| 33 | CFB | **331** | BAX | **629** | MYC | **926** | PARK7 |
| 34 | GFAP | **332** | CCN2 | **630** | BRCA1 | **927** | SLC2A1 |
| 35 | CUBN | **333** | MMP2 | **631** | JAK2 | **928** | CBS |
| 36 | NF2 | **334** | GPT | **632** | SLC5A1 | **929** | SELP |
| 37 | RUNX1 | **335** | CLU | **633** | JAG1 | **930** | NPPB |
| 38 | STK11 | **336** | ADIPOQ | **634** | TRIM8 | **931** | FGA |
| 39 | FASLG | **337** | PARP1 | **635** | CFTR | **932** | SIX2 |
| 40 | NOTCH1 | **338** | SPP1 | **636** | IL1RN | **933** | SNHG14 |
| 41 | BMP7 | **339** | ENO2 | **637** | FABP1 | **934** | VDR |
| 42 | CALCA | **340** | GSK3B | **638** | TRAP1 | **935** | IL4 |
| 43 | KDR | **341** | PDGFRB | **639** | AQP1 | **936** | MTHFR |
| 44 | YAP1 | **342** | TIMP2 | **640** | HGF | **937** | MIR126 |
| 45 | GJA1 | **343** | NOX4 | **641** | CXCL10 | **938** | MIR145 |
| 46 | PPARG | **344** | FOXC1 | **642** | CXCL12 | **939** | HOTAIR |
| 47 | FLT1 | **345** | TLR2 | **643** | CYP3A4 | **940** | STAT1 |
| 48 | MIR210 | **346** | PIK3CG | **644** | MIR320A | **941** | NR3C2 |
| 49 | KCNQ1OT1 | **347** | CAT | **645** | SNHG12 | **942** | SERPINA1 |
| 50 | FAS | **348** | SMAD3 | **646** | MAPK8 | **943** | MB |
| 51 | SNHG1 | **349** | LEP | **647** | CDKN2B-AS1 | **944** | NTRK1 |
| 52 | ABCC2 | **350** | BDNF | **648** | IL1A | **945** | ABCC8 |
| 53 | HSPB1 | **351** | EGLN3 | **649** | SMAD2 | **946** | F5 |
| 54 | KEAP1 | **352** | SOX9 | **650** | SHH | **947** | CAV1 |
| 55 | MAPT | **353** | MIAT | **651** | BMP2 | **948** | ELANE |
| 56 | DNM1L | **354** | SERPINC1 | **652** | TIMP1 | **949** | XIST |
| 57 | LINC02605 | **355** | F3 | **653** | SLC22A6 | **950** | FGFR2 |
| 58 | TNFRSF1A | **356** | ABCG2 | **654** | COMT | **951** | ROBO1 |
| 59 | SOD2 | **357** | ACTA2 | **655** | SELE | **952** | PTPRO |
| 60 | MIR125A | **358** | MIR17 | **656** | BIRC5 | **953** | NOD2 |
| 61 | TF | **359** | FGF2 | **657** | VCAM1 | **954** | SGK1 |
| 62 | CUL3 | **360** | CASP9 | **658** | PLG | **955** | PIK3C2A |
| 63 | KMT2D | **361** | EPAS1 | **659** | AGER | **956** | RELA |
| 64 | EIF2AK3 | **362** | FOXC2 | **660** | MAPK14 | **957** | DNASE1 |
| 65 | MAP2K1 | **363** | MIR155 | **661** | MAPK3 | **958** | HAMP |
| 66 | MIR122 | **364** | HBEGF | **662** | BRD4 | **959** | ITGB2 |
| 67 | CD40LG | **365** | NGF | **663** | IGF1R | **960** | MIR29A |
| 68 | CDKN1A | **366** | FOXO3 | **664** | HSPG2 | **961** | DNMT1 |
| 69 | KNG1 | **367** | MDM2 | **665** | FTO | **962** | CASP8 |
| 70 | CASP5 | **368** | PCSK9 | **666** | PON1 | **963** | GDF15 |
| 71 | SLIT2 | **369** | G6PC1 | **667** | CRNDE | **964** | CD44 |
| 72 | SCARB1 | **370** | SLC22A2 | **668** | CCR2 | **965** | AGTR2 |
| 73 | MIR150 | **371** | EGR1 | **669** | LGALS3 | **966** | MIR532 |
| 74 | MUTYH | **372** | NFKB1 | **670** | AKT3 | **967** | APP |
| 75 | NFKBIA | **373** | DCDC2 | **671** | MIF | **968** | AIFM1 |
| 76 | EZH2 | **374** | WNT5A | **672** | PRKAG2 | **969** | GCK |
| 77 | CPT2 | **375** | KCNJ11 | **673** | NOS1 | **970** | CYBB |
| 78 | AMBP | **376** | PDGFB | **674** | MECP2 | **971** | MIR15A |
| 79 | ADM | **377** | JUN | **675** | MYD88 | **972** | ACTB |
| 80 | MIR106B | **378** | BCL2L1 | **676** | CCL5 | **973** | GFER |
| 81 | DHCR7 | **379** | YRDC | **677** | DDIT3 | **974** | MIR214 |
| 82 | ZEB2 | **380** | ESR1 | **678** | ATP7B | **975** | PTGS1 |
| 83 | CASP4 | **381** | ALDH1A2 | **679** | DPP4 | **976** | TNFSF10 |
| 84 | TGFB2 | **382** | MIR141 | **680** | MIR27A | **977** | SQSTM1 |
| 85 | ZFAS1 | **383** | IL17A | **681** | MBL2 | **978** | RHOA |
| 86 | CD36 | **384** | LMNA | **682** | SIRT3 | **979** | C5 |
| 87 | FOXO1 | **385** | GAPDH | **683** | THBS1 | **980** | PPARGC1A |
| 88 | CXCR3 | **386** | SNHG6 | **684** | CYCS | **981** | ZNF609 |
| 89 | MIR133B | **387** | PTPRC | **685** | CDC42 | **982** | PTPN11 |
| 90 | SNHG7 | **388** | TGFBR1 | **686** | APOB | **983** | PCNA |
| 91 | SNCA | **389** | CD40 | **687** | NTN1 | **984** | BECN1 |
| 92 | ADAM17 | **390** | PLA2G6 | **688** | MMP3 | **985** | AQP4 |
| 93 | FLNA | **391** | C5AR1 | **689** | KISS1 | **986** | SOCS1 |
| 94 | ALDH2 | **392** | MIR146B | **690** | FGF7 | **987** | HAVCR2 |
| 95 | APLN | **393** | ADA | **691** | SLC1A1 | **988** | SRC |
| 96 | OCLN | **394** | BMP6 | **692** | MAP4K4 | **989** | GSTP1 |
| 97 | PCAT1 | **395** | XIAP | **693** | VTN | **990** | HP |
| 98 | COL1A1 | **396** | MIR146A | **694** | SDC1 | **991** | DNMT3A |
| 99 | GGT1 | **397** | ANGPT1 | **695** | METTL3 | **992** | UCP2 |
| 100 | ITCH | **398** | MIR142 | **696** | JAK1 | **993** | ITPR1 |
| 101 | IL33 | **399** | CHI3L1 | **697** | CP | **994** | KLF4 |
| 102 | MIR30A | **400** | SELL | **698** | SLPI | **995** | ADCY10 |
| 103 | FOS | **401** | MIR34A | **699** | FGD5-AS1 | **996** | MAFB |
| 104 | MIR140 | **402** | HSPA4 | **700** | CXCL1 | **997** | TRAF3 |
| 105 | MIR211 | **403** | RAC1 | **701** | HCP5 | **998** | SLC6A19 |
| 106 | TNFAIP3 | **404** | TJP1 | **702** | FGB | **999** | PPARA |
| 107 | STING1 | **405** | CDK6 | **703** | TRA-TGC7-1 | **1000** | CXCL2 |
| 108 | CCR5 | **406** | SOCS3 | **704** | MIR191 | **1001** | PTHLH |
| 109 | TNFRSF11B | **407** | PTK2 | **705** | CREB1 | **1002** | FMR1 |
| 110 | G6PD | **408** | NEU1 | **706** | NQO1 | **1003** | RUNX2 |
| 111 | ANGPT2 | **409** | PROM1 | **707** | FTX | **1004** | TNFRSF1B |
| 112 | HDAC4 | **410** | PGK1 | **708** | PDPN | **1005** | ELAVL1 |
| 113 | GHRL | **411** | C4B | **709** | CD55 | **1006** | TLR7 |
| 114 | TNXB | **412** | IRF1 | **710** | YY1 | **1007** | F10 |
| 115 | RIPK3 | **413** | AHSG | **711** | PLAUR | **1008** | CD59 |
| 116 | PLAT | **414** | CYP2C9 | **712** | MIR106A | **1009** | SLC9A1 |
| 117 | FLT3 | **415** | MIR424 | **713** | TYROBP | **1010** | GZMB |
| 118 | MIR221 | **416** | MASP1 | **714** | ECE1 | **1011** | CRBN |
| 119 | COL3A1 | **417** | F8 | **715** | MIR342 | **1012** | SEMA3A |
| 120 | CYP1B1 | **418** | ATF3 | **716** | MIR378A | **1013** | CCL3 |
| 121 | LINC01234 | **419** | TRPV1 | **717** | IL13 | **1014** | HSPA1A |
| 122 | PRKN | **420** | CSF3 | **718** | PECAM1 | **1015** | PWAR1 |
| 123 | IDH1 | **421** | MIRLET7C | **719** | TXNIP | **1016** | KRT18 |
| 124 | TNNT2 | **422** | IRAK1 | **720** | GLUD1 | **1017** | AXL |
| 125 | SLC7A11 | **423** | CD47 | **721** | MIR144 | **1018** | HIF1A-AS2 |
| 126 | JAK3 | **424** | MT-CYB | **722** | HSPD1 | **1019** | TRIM28 |
| 127 | MIR199A1 | **425** | GNA11 | **723** | SLC6A4 | **1020** | TXN |
| 128 | IRF5 | **426** | MIR193A | **724** | DUSP6 | **1021** | PRKDC |
| 129 | LPL | **427** | CSF1 | **725** | NORAD | **1022** | MAOA |
| 130 | GRIN2B | **428** | MIR4435-2HG | **726** | CAMK2B | **1023** | GP1BA |
| 131 | CSF2 | **429** | CTSD | **727** | IL15 | **1024** | FNDC5 |
| 132 | FASN | **430** | DKK1 | **728** | AKR1B1 | **1025** | UCA1 |
| 133 | ALOX5 | **431** | LOX | **729** | RIPK1 | **1026** | GSTM1 |
| 134 | CPT1A | **432** | KDM1A | **730** | STIM1 | **1027** | TNNI3 |
| 135 | CYBA | **433** | GUSB | **731** | LAMP2 | **1028** | PSEN1 |
| 136 | TRPV4 | **434** | NPY | **732** | COL4A2 | **1029** | CR1 |
| 137 | MIR34C | **435** | AIF1 | **733** | TET2 | **1030** | SNHG4 |
| 138 | FOXM1 | **436** | TLR3 | **734** | COL18A1 | **1031** | MIRLET7B |
| 139 | MIR93 | **437** | NEDD4L | **735** | TBK1 | **1032** | USP7 |
| 140 | HSP90AA1 | **438** | EDNRB | **736** | SCGB1A1 | **1033** | PIK3CB |
| 141 | BSG | **439** | PRKCD | **737** | SLC12A2 | **1034** | HDAC1 |
| 142 | GLP1R | **440** | GJB1 | **738** | AKT2 | **1035** | MIR9-1 |
| 143 | IL1R1 | **441** | PLAU | **739** | IL1RL1 | **1036** | TNFSF12 |
| 144 | FABP7 | **442** | GSR | **740** | SLC22A8 | **1037** | SERPING1 |
| 145 | KCNJ5 | **443** | COLEC11 | **741** | TRPS1 | **1038** | CKB |
| 146 | MIR22 | **444** | IL7 | **742** | TRPM7 | **1039** | GRIN1 |
| 147 | CXCL9 | **445** | ABCA1 | **743** | CBL | **1040** | CYTOR |
| 148 | ITGB1 | **446** | STUB1 | **744** | NR4A1 | **1041** | GPX3 |
| 149 | SLC1A2 | **447** | HDAC6 | **745** | TKT | **1042** | TTN |
| 150 | C1S | **448** | MASP2 | **746** | RYR1 | **1043** | GLUL |
| 151 | MIR19A | **449** | MIR98 | **747** | BNIP3 | **1044** | HMGCR |
| 152 | ORAI1 | **450** | PRKCA | **748** | HTRA2 | **1045** | VEGFC |
| 153 | MIR335 | **451** | NGFR | **749** | MIR23A | **1046** | GCG |
| 154 | SMPD1 | **452** | TLR9 | **750** | PINK1 | **1047** | DAPK1 |
| 155 | MANF | **453** | CASP7 | **751** | LINC-ROR | **1048** | SNHG16 |
| 156 | S100A9 | **454** | CDKN3 | **752** | CLDN5 | **1049** | FGF17 |
| 157 | MIR192 | **455** | CX3CL1 | **753** | DCN | **1050** | NES |
| 158 | SNAI1 | **456** | NBAS | **754** | CALR | **1051** | CLDN7 |
| 159 | MFN2 | **457** | KCNQ1 | **755** | ROCK1 | **1052** | MAP1LC3A |
| 160 | ATF4 | **458** | SGPP1 | **756** | SLC6A3 | **1053** | GATA4 |
| 161 | MIR486-1 | **459** | TIMP3 | **757** | CFP | **1054** | IRS1 |
| 162 | SHC1 | **460** | CRYAB | **758** | KLK1 | **1055** | GSDMD |
| 163 | SPTAN1 | **461** | CXCL5 | **759** | ERN1 | **1056** | MIR451A |
| 164 | ADNP | **462** | CDK1 | **760** | MMP14 | **1057** | FABP3 |
| 165 | EPOR | **463** | MIR7-3HG | **761** | FABP2 | **1058** | SREBF1 |
| 166 | FLRT3 | **464** | IRF3 | **762** | CD34 | **1059** | LEF1-AS1 |
| 167 | CFLAR | **465** | PSAP | **763** | HSPA8 | **1060** | MIR20B |
| 168 | C3AR1 | **466** | RTN4 | **764** | VCP | **1061** | KCNMA1 |
| 169 | CASP1 | **467** | TGIF1 | **765** | SMAD7 | **1062** | CPOX |
| 170 | CTSB | **468** | FBXW7 | **766** | ATP7A | **1063** | CLDN1 |
| 171 | MIR216A | **469** | NDUFS4 | **767** | DRD2 | **1064** | MIR204 |
| 172 | NT5E | **470** | YY1AP1 | **768** | MCL1 | **1065** | KLF11 |
| 173 | HDAC9 | **471** | ADORA2A | **769** | GRN | **1066** | NR4A2 |
| 174 | IDO1 | **472** | ATF6 | **770** | MIR24-1 | **1067** | NEUROD1 |
| 175 | GPNMB | **473** | AIM2 | **771** | CD14 | **1068** | MIR18A |
| 176 | MIR25 | **474** | MIR15B | **772** | CHAT | **1069** | FGF21 |
| 177 | MERTK | **475** | FGF1 | **773** | OIP5-AS1 | **1070** | LAMB1 |
| 178 | IGF2BP2 | **476** | S100A8 | **774** | FADD | **1071** | ERBB4 |
| 179 | IKBKB | **477** | UGT1A1 | **775** | ABCC9 | **1072** | PTK2B |
| 180 | MLKL | **478** | MIR30B | **776** | EPHA2 | **1073** | PCK1 |
| 181 | ABL1 | **479** | ANXA5 | **777** | DDX3X | **1074** | AHR |
| 182 | MAP3K5 | **480** | APEX1 | **778** | MIR29B1 | **1075** | IL11 |
| 183 | ATP5F1A | **481** | CDON | **779** | ENTPD1 | **1076** | VLDLR |
| 184 | ANXA2 | **482** | SLC25A4 | **780** | SNORD15A | **1077** | DIABLO |
| 185 | SOD3 | **483** | SOS1 | **781** | RPTOR | **1078** | CX3CR1 |
| 186 | E2F1 | **484** | CXCL16 | **782** | NR3C1 | **1079** | CACNA1C |
| 187 | ADGRG1 | **485** | ERCC2 | **783** | COL7A1 | **1080** | PTX3 |
| 188 | MIR377 | **486** | ADAMTS9 | **784** | EPRS1 | **1081** | MDH2 |
| 189 | ATR | **487** | TNFRSF10B | **785** | TRIM72 | **1082** | CDH5 |
| 190 | LRRK2 | **488** | MRE11 | **786** | ABCB7 | **1083** | BAK1 |
| 191 | SLTM | **489** | FCGR3B | **787** | WAC | **1084** | DIAPH1 |
| 192 | CXCR2 | **490** | CHKA | **788** | TSPO | **1085** | FUS |
| 193 | RMRP | **491** | MIR200A | **789** | RELN | **1086** | LAMA2 |
| 194 | MIR124-1 | **492** | MIR132 | **790** | SIRT6 | **1087** | SCARNA5 |
| 195 | P2RX7 | **493** | PDCD4 | **791** | F2R | **1088** | ACSL4 |
| 196 | MGAT3-AS1 | **494** | CYP2E1 | **792** | FOXP2 | **1089** | APAF1 |
| 197 | MIR92A1 | **495** | COX5A | **793** | PIK3R1 | **1090** | ITGA2B |
| 198 | ATG7 | **496** | CIITA | **794** | ADAM10 | **1091** | NR1I2 |
| 199 | IL22 | **497** | GPX1 | **795** | GPX4 | **1092** | WNT3A |
| 200 | ADRB2 | **498** | MIR328 | **796** | SLC1A3 | **1093** | PRDX1 |
| 201 | PTGDS | **499** | MIR125B1 | **797** | ULK1 | **1094** | NID1 |
| 202 | TP63 | **500** | MAP3K7 | **798** | AOC3 | **1095** | SOX4 |
| 203 | TEK | **501** | ETS1 | **799** | VDAC1 | **1096** | BSCL2 |
| 204 | SPARC | **502** | BGN | **800** | CKM | **1097** | LIF |
| 205 | MIR381 | **503** | MCOLN1 | **801** | ANXA1 | **1098** | LGALS1 |
| 206 | S1PR1 | **504** | ADM2 | **802** | MIR139 | **1099** | LTF |
| 207 | IFNB1 | **505** | ALOX12 | **803** | ALDH5A1 | **1100** | LRP6 |
| 208 | IL7R | **506** | MCAM | **804** | ENO1 | **1101** | NRG1 |
| 209 | MIR10A | **507** | PIAS1 | **805** | SESN2 | **1102** | HCRT |
| 210 | PRKCB | **508** | GLS | **806** | EPHB2 | **1103** | SNAP25 |
| 211 | IL6ST | **509** | WTAP | **807** | TAB2 | **1104** | VCAN |
| 212 | CORIN | **510** | STAT4 | **808** | MCU | **1105** | SYK |
| 213 | XBP1 | **511** | IL3 | **809** | GAD1 | **1106** | SIRT2 |
| 214 | TNC | **512** | MIR222 | **810** | IL37 | **1107** | IGFBP7 |
| 215 | PXN | **513** | INTU | **811** | HTR2A | **1108** | DAG1 |
| 216 | TET3 | **514** | ADCYAP1 | **812** | TRAF6 | **1109** | LONP1 |
| 217 | LIMK1 | **515** | IKZF1 | **813** | CRH | **1110** | SCO1 |
| 218 | GPBAR1 | **516** | MIR149 | **814** | TREM1 | **1111** | PPIG |
| 219 | NEFL | **517** | NOL3 | **815** | DKK3 | **1112** | OPA1 |
| 220 | CCR7 | **518** | PRKAA1 | **816** | BBC3 | **1113** | CCK |
| 221 | DYSF | **519** | CD9 | **817** | ACVR2B | **1114** | CD81 |
| 222 | DLG4 | **520** | EIF4EBP1 | **818** | GDF11 | **1115** | IKBKG |
| 223 | ADORA1 | **521** | IRAK4 | **819** | P2RY12 | **1116** | CLCN3 |
| 224 | LRG1 | **522** | TNFRSF12A | **820** | BTG2 | **1117** | CYP2J2 |
| 225 | NOX1 | **523** | C1QBP | **821** | GRK4 | **1118** | REG3A |
| 226 | SERPINA3 | **524** | PPM1D | **822** | TYK2 | **1119** | HULC |
| 227 | ATG5 | **525** | MT-ND3 | **823** | PROCR | **1120** | NPPC |
| 228 | AGRN | **526** | ZC3H12A | **824** | SMN1 | **1121** | CHRM3 |
| 229 | RAP1B | **527** | HAX1 | **825** | ANGPTL4 | **1122** | C1R |
| 230 | CCR6 | **528** | PRKCZ | **826** | MIRLET7F2 | **1123** | MIRLET7A1 |
| 231 | STAT5B | **529** | CD80 | **827** | METTL14 | **1124** | CLEC7A |
| 232 | MPL | **530** | MIR183 | **828** | IBSP | **1125** | OXSR1 |
| 233 | ABAT | **531** | EIF4E | **829** | SLCO2A1 | **1126** | GRIN2A |
| 234 | GADD45A | **532** | SERPINF1 | **830** | UTS2 | **1127** | H2AX |
| 235 | GAL | **533** | IL12A | **831** | COPA | **1128** | EGLN1 |
| 236 | ZAP70 | **534** | ROCK2 | **832** | SULT1A3 | **1129** | ATP2A2 |
| 237 | CACNA1D | **535** | BCHE | **833** | TMEM126B | **1130** | IL1RAPL2 |
| 238 | CXCL13 | **536** | MIR101-1 | **834** | FGF4 | **1131** | S1PR2 |
| 239 | GAST | **537** | COL6A1 | **835** | MIR590 | **1132** | CHUK |
| 240 | TNFRSF10A | **538** | ETHE1 | **836** | MIR195 | **1133** | RYR2 |
| 241 | KCNA2 | **539** | YBX1 | **837** | NCF1 | **1134** | SEMA7A |
| 242 | MIR485 | **540** | PLOD2 | **838** | IL17RA | **1135** | MIR130B |
| 243 | LTBP4 | **541** | LAMA1 | **839** | GRK2 | **1136** | SH2B3 |
| 244 | CTSG | **542** | FGF19 | **840** | PFKFB3 | **1137** | MIR30C1 |
| 245 | BAD | **543** | PLA2G7 | **841** | HOXA11 | **1138** | YWHAG |
| 246 | MAP1LC3B | **544** | RACK1 | **842** | MIR215 | **1139** | SLC40A1 |
| 247 | PWAR4 | **545** | IL5 | **843** | VSIG4 | **1140** | ADIPOR1 |
| 248 | MIR133A1 | **546** | SCN5A | **844** | KIF3B | **1141** | CHMP2B |
| 249 | BCKDK | **547** | MIR143 | **845** | SRSF1 | **1142** | TNIP1 |
| 250 | MIR483 | **548** | MSTN | **846** | APLNR | **1143** | ANO1 |
| 251 | ANK3 | **549** | TAC1 | **847** | TRE-TTC3-1 | **1144** | MIR188 |
| 252 | PRDM16 | **550** | MIR501 | **848** | MIR181A2 | **1145** | PKD1L1 |
| 253 | KCNN4 | **551** | MT-ND6 | **849** | SNX10 | **1146** | SARM1 |
| 254 | KITLG | **552** | PNPT1 | **850** | TRAF2 | **1147** | SIRPA |
| 255 | MYBPC3 | **553** | TGM2 | **851** | BDKRB1 | **1148** | BDKRB2 |
| 256 | FOXO3 | **554** | YRDC | **852** | TLR4 | **1149** | ADIPOQ |
| 257 | G6PD | **555** | RNF182 | **853** | TMBIM6 | **1150** | EPO |
| 258 | HAVCR1 | **556** | BCL2L14 | **854** | MIR496 | **1151** | FGB |
| 259 | PRKCE | **557** | HNF1B | **855** | HSF1 | **1152** | CD300LB |
| 260 | THBS1 | **558** | PAPRS | **856** | RHDA1 | **1153** | TET2 |
| 261 | ZNRF1 | **559** | NINJ1 | **857** | TMEM123 | **1154** | NINJ2 |
| 262 | HLRCC | **560** | HOMG2 | **858** | ARCS1 | **1155** | HOMG3 |
| 263 | DRTA2 | **561** | RTD | **859** | PRTAO | **1156** | TNFAIP8L2 |
| 264 | RAPGEF3 | **562** | ECHDC2 | **860** | CAMK2A | **1157** | LCN2 |
| 265 | STIM1 | **563** | HDRS | **861** | DRTA1 | **1158** | MTDPS8A |
| 266 | DRTA4 | **564** | HOMG4 | **862** | AI1G | **1159** | RHUC1 |
| 267 | HOMG5 | **565** | EPM4 | **863** | SLSN1 | **1160** | STAR |
| 268 | RCC | **566** | GLYS | **864** | MARCH | **1161** | NFSRA |
| 269 | RFH1 | **567** | AABT | **865** | DPEP1 | **1162** | RNF139 |
| 270 | MIF | **568** | SLC4A3 | **866** | PRKAA1 | **1163** | DHCR7 |
| 271 | SHC3 | **569** | PARP1 | **867** | PPIF | **1164** | ZNF667 |
| 272 | HTRA2 | **570** | TXNRD2 | **868** | PARP16 | **1165** | LPHDST |
| 273 | AMYLD2 | **571** | TBS1 | **869** | FRTS1 | **1166** | RCAD |
| 274 | HLTRS | **572** | XRN | **870** | RHUC2 | **1167** | MVCD2 |
| 275 | MVCD3 | **573** | TSC2 | **871** | ARCS2 | **1168** | CAKUT1 |
| 276 | HUPRAS | **574** | HOMG6 | **872** | NPHS5 | **1169** | CMM8 |
| 277 | SHDRA | **575** | RENI | **873** | VCRL1 | **1170** | VCRL2 |
| 278 | RHDA3 | **576** | NEDMCR | **874** | MFRG | **1171** | RHPD2 |
| 279 | RHDA2 | **577** | WEDAS | **875** | CYSRD | **1172** | RVCLS |
| 280 | RHPD1 | **578** | CDSP | **876** | FBS | **1173** | BARTS4A |
| 281 | DRTA3 | **579** | FSGS10 | **877** | OPTB3 | **1174** | SRTD9 |
| 282 | PRLMNS | **580** | MKS7 | **878** | RCDFRD | **1175** | SLOS |
| 283 | MVCD1 | **581** | RCCP1 | **879** | RCCX1 | **1176** | GTPTS |
| 284 | CAKUT2 | **582** | BNAR | **880** | VCRL3 | **1177** | VCTERL |
| 285 | NECRC | **583** | BRENS | **881** | RHDA4 | **1178** | HOMG7 |
| 286 | BMRS | **584** | DEDCRF | **882** | TKCR | **1179** | MURCS |
| 287 | KLK1 | **585** | ADRA2C | **883** | PRCC | **1180** | SLC17A1 |
| 288 | SLC34A1 | **586** | SLC5A2 | **884** | KCNJ1 | **1181** | CEP83 |
| 289 | C4ORF46 | **587** | SLC14A2 | **885** | NPHS1 | **1182** | SLC49A4 |
| 290 | MOK | **588** | DIRC1 | **886** | SFMBT1 | **1183** | DIRC3 |
| 291 | FAM107A | **589** | NOX4 | **887** | GTPBP4 | **1184** | PKD1 |
| 292 | TGFB1 | **590** | EGFR | **888** | WT1 | **1185** | AGTR1 |
| 293 | DACH1 | **591** | TLR2 | **889** | C3 | **1186** | PTGS2 |
| 294 | AQP4 | **592** | TNF | **890** | FGFR1 | **1187** | TNFRSF1A |
| 295 | TNFRSF1B | **593** | MTOR | **891** | TMIGD1 | **1188** | CCND1 |
| 296 | SOSTDC1 | **594** | IL10 | **892** | CXCR4 | **1189** | SOCS3 |
| 297 | FGA | **595** | MET | **893** | ALGS1 | **1190** | IL1A |
| 298 | TTC36 | **596** | APOE |  |  |  |  |

**Table S6 Characteristic targets of R-IRI from GEO**

| NO | Target | NO | Target | NO | Target | NO | Target |
| --- | --- | --- | --- | --- | --- | --- | --- |
| 1 | TXNDC15 | **531** | RPL22L1 | **1061** | TUBB6 | **1591** | FOSL1 |
| 2 | CPQ | **532** | AKAP12 | **1062** | RPS5 | **1592** | CHAC1 |
| 3 | TRPC3 | **533** | CD44 | **1063** | MAFF | **1593** | SPHK1 |
| 4 | EPS8L3 | **534** | PLAUR | **1064** | CCL2 | **1594** | PKP3 |
| 5 | SOX9 | **535** | PLA2G3 | **1065** | KRT8 | **1595** | ARID5A |
| 6 | KLHL3 | **536** | PVR | **1066** | HACL1 | **1596** | IL34 |
| 7 | MMAA | **537** | SLC7A5P2 | **1067** | ZSCAN12 | **1597** | EIF6 |
| 8 | CSF1 | **538** | RPS25 | **1068** | EMP1 | **1598** | EPHA2 |
| 9 | PTP4A1 | **539** | SSB | **1069** | IGHM | **1599** | CDC42EP2 |
| 10 | AVPR2 | **540** | MYC | **1070** | ATP7B | **1600** | PTPRE |
| 11 | DRGX | **541** | HARS1 | **1071** | HARS | **1601** | MTHFD2 |
| 12 | VCAM1 | **542** | FGFBP1 | **1072** | CENPT | **1602** | SFN |
| 13 | REXO2 | **543** | CD14 | **1073** | CH25H | **1603** | IPCEF1 |
| 14 | DNTT | **544** | SPINT2 | **1074** | SOCS3 | **1604** | ARHGAP24 |
| 15 | FGD3 | **545** | HILPDA | **1075** | EDN1 | **1605** | RAP2B |
| 16 | MAP3K6 | **546** | BCL11B | **1076** | ATL1-gamma | **1606** | ATL1-alpha |
| 17 | APCDD1 | **547** | ICAM1 | **1077** | DUOX1 | **1607** | C2CD4A |
| 18 | NEK6 | **548** | GAST | **1078** | GAS | **1608** | FAM161B |
| 19 | TMEM210 | **549** | FRAS1 | **1079** | MIS18BP1 | **1609** | MT2A |
| 20 | LHX4 | **550** | EIF1AX | **1080** | BCL3 | **1610** | HMGA1 |
| 21 | MAP3K8 | **551** | MBOAT7 | **1081** | MBLAC2 | **1611** | CCDC61 |
| 22 | CENPO | **552** | PLEKHA6 | **1082** | TRIB3 | **1612** | CRIP3 |
| 23 | CIMAP1B | **553** | ODF3B | **1083** | MAMLD1 | **1613** | S100A10 |
| 24 | FOLR2 | **554** | PCDH12 | **1084** | EGR2 | **1614** | TLX2 |
| 25 | NXPH4 | **555** | NFKBIZ | **1085** | HES2 | **1615** | RHBDF2 |
| 26 | F3 | **556** | BDKRB1 | **1086** | BDKRB2 | **1616** | PDK4 |
| 27 | ECEL1 | **557** | KIF23 | **1087** | LILRB4 | **1617** | FRAT2 |
| 28 | P2RX3 | **558** | TMC8 | **1088** | PURG | **1618** | ETS2 |
| 29 | MTERF2 | **559** | ZFAND2A | **1089** | CROCC | **1619** | KCTD7 |
| 30 | RAB3A | **560** | MAFK | **1090** | NIBAN1 | **1620** | MGMT |
| 31 | STAT3 | **561** | TNFRSF12A | **1091** | FCHSD1 | **1621** | POLD1 |
| 32 | LRRC66 | **562** | RHOJ | **1092** | CDKN1A | **1622** | SLC10A6 |
| 33 | SLC25A33 | **563** | CTPS1 | **1093** | GIGYF2 | **1623** | RAB19 |
| 34 | S1PR1 | **564** | ODF4 | **1094** | MMD | **1624** | TUBD1 |
| 35 | APLNR | **565** | PRKRA | **1095** | SERPINE1 | **1625** | HSD3B1 |
| 36 | CLDN4 | **566** | TNFAIP3 | **1096** | KRTAP4-4 | **1626** | ZNF235 |
| 37 | TMEM117 | **567** | NOL3 | **1097** | ARC | **1627** | SLC7A5 |
| 38 | KLF6 | **568** | ADAMTS1 | **1098** | LCN2 | **1628** | SIK1 |
| 39 | H2AB1 | **569** | CEP131 | **1099** | AZI1 | **1629** | TSSK4 |
| 40 | KCP | **570** | ARG2 | **1100** | SLC6A11 | **1630** | TNFAIP8L1 |
| 41 | NIPAL4 | **571** | EFHD2 | **1101** | FGD6 | **1631** | DTNB |
| 42 | ADM | **572** | TMIE | **1102** | PTBP3 | **1632** | COLGALT2 |
| 43 | GLT25D2 | **573** | TXNRD1 | **1103** | KLK15 | **1633** | PPAN |
| 44 | JOSD2 | **574** | CTRB1 | **1104** | TPBG | **1634** | ANKS1B |
| 45 | GAS2L3 | **575** | SIM1 | **1105** | AGTR1 | **1635** | GEM |
| 46 | REC8 | **576** | ATF3 | **1106** | TBXAS1 | **1636** | TMEM177 |
| 47 | GPHA2 | **577** | GPA2 | **1107** | CRYGD | **1637** | KCNS2 |
| 48 | TRPS1 | **578** | GSC2 | **1108** | F9 | **1638** | RASA2 |
| 49 | RELA | **579** | CD68 | **1109** | TGM1 | **1639** | LGALS3 |
| 50 | DNAJB9 | **580** | TMEM45A | **1110** | WDR86 | **1640** | RHOBTB2 |
| 51 | CD5 | **581** | AMER3 | **1111** | VPS37B | **1641** | ADAMTS15 |
| 52 | RAB30 | **582** | FGL2 | **1112** | DAB2 | **1642** | NODAL |
| 53 | TGFBI | **583** | IER5 | **1113** | PPRC1 | **1643** | RIOK2 |
| 54 | SPCS2 | **584** | SPC25 | **1114** | PDS5B | **1644** | NLK |
| 55 | CRYBG2 | **585** | COL26A1 | **1115** | AZGP1 | **1645** | MAMSTR |
| 56 | BANP | **586** | TTC14 | **1116** | PCMTD2 | **1646** | ARHGEF25 |
| 57 | TGDS | **587** | THBD | **1117** | TGIF1 | **1647** | RASD1 |
| 58 | ENTPD1 | **588** | RIN1 | **1118** | MAP2K3 | **1648** | RBBP9 |
| 59 | BSND | **589** | FRS3 | **1119** | SLAMF9 | **1649** | CORT |
| 60 | FOXH1 | **590** | MTFP1 | **1120** | HRH3 | **1650** | MRPS2 |
| 61 | TCL1B | **591** | TCL1A | **1121** | DTX1 | **1651** | CCDC28B |
| 62 | HSD3B2 | **592** | PPCDC | **1122** | CDKL1 | **1652** | HDC |
| 63 | HECA | **593** | RELB | **1123** | TBCCD1 | **1653** | NFIL3 |
| 64 | TRPV6 | **594** | ZFAB | **1124** | CYB561A3 | **1654** | SHC4 |
| 65 | GNPDA1 | **595** | IL27 | **1125** | ZFP14 | **1655** | BAHCC1 |
| 66 | SEMA6B | **596** | LY6G5C | **1126** | TIFAB | **1656** | FZD1 |
| 67 | MPP3 | **597** | DLG3 | **1127** | PAPPA | **1657** | JAML |
| 68 | AMICA1 | **598** | DIO1 | **1128** | ADGRF5 | **1658** | GPR116 |
| 69 | CAMK2D | **599** | SPRR2F | **1129** | GALNT12 | **1659** | UNC93B1 |
| 70 | GADD45A | **600** | TMEM25 | **1130** | SIX5 | **1660** | GALR3 |
| 71 | MGAT3 | **601** | CPAP | **1131** | CENPJ | **1661** | GSG1 |
| 72 | RHBDL1 | **602** | YBEY | **1132** | MALL | **1662** | PPP2R2C |
| 73 | MCL1 | **603** | MC2R | **1133** | RHBDL3 | **1663** | POLE2 |
| 74 | TMEM80 | **604** | NUDT11 | **1134** | SLC25A32 | **1664** | CRY1 |
| 75 | KANK4 | **605** | SLC35E4 | **1135** | POLI | **1665** | PIK3R5 |
| 76 | ALPG | **606** | NRG1 | **1136** | CD53 | **1666** | NFE2L2 |
| 77 | RLIM | **607** | TDO2 | **1137** | ASCL1 | **1667** | B4GALNT4 |
| 78 | YRDC | **608** | SOSTDC1 | **1138** | HSPB1 | **1668** | STOX2 |
| 79 | PRKCQ | **609** | CYP1B1 | **1139** | GADD45B | **1669** | ZFP41 |
| 80 | PURA | **610** | EVC2 | **1140** | ESRRA | **1670** | DEPDC7 |
| 81 | TMEM72 | **611** | CCNA2 | **1141** | FEM1B | **1671** | BTG3 |
| 82 | CTNS | **612** | POGLUT2 | **1142** | KDELC1 | **1672** | EN2 |
| 83 | EHD3 | **613** | ADRA1D | **1143** | PCBP1 | **1673** | RASIP1 |
| 84 | GMCL1 | **614** | FLJ13057 | **1144** | LST1 | **1674** | SLCO1B1 |
| 85 | DCAF4 | **615** | GREM2 | **1145** | IGF2BP2 | **1675** | PLXNA2 |
| 86 | ATF6 | **616** | CTRL | **1146** | ADAM5 | **1676** | CTSC |
| 87 | DDHD1 | **617** | BARHL1 | **1147** | SCRN1 | **1677** | PROP1 |
| 88 | LYZL4 | **618** | LYZA | **1148** | RND3 | **1678** | UGCG |
| 89 | RTL8B | **619** | DUSP4 | **1149** | GLIPR2 | **1679** | ENTREP3 |
| 90 | CLCF1 | **620** | NFKBIA | **1150** | MYBL2 | **1680** | LTB |
| 91 | CALML3 | **621** | JUNB | **1151** | CHST12 | **1681** | NPFF |
| 92 | SEMA7A | **622** | ATP1A4 | **1152** | LHX1 | **1682** | KCNJ1 |
| 93 | SLC26A1 | **623** | KLHL17 | **1153** | PLA2G5 | **1683** | SALL2 |
| 94 | KLF11 | **624** | DET1 | **1154** | MNS1 | **1684** | PAQR6 |
| 95 | RBL1 | **625** | CBX8 | **1155** | WNT10A | **1685** | SPACA7 |
| 96 | COPZ2 | **626** | CRYZL1 | **1156** | INTS3 | **1686** | TPPP3 |
| 97 | CD164L2 | **627** | RYR3 | **1157** | STRC | **1687** | PDE10A |
| 98 | GEMIN8 | **628** | TSPAN18 | **1158** | BAG3 | **1688** | LY6G6D |
| 99 | LY6G6F | **629** | TRIB1 | **1159** | TMPRSS3 | **1689** | TMPRSS4 |
| 100 | FAM181B | **630** | NAGPA | **1160** | INHBB | **1690** | MPPE1 |
| 101 | GNAZ | **631** | RIOK1 | **1161** | METTL3 | **1691** | CREB5 |
| 102 | CXADR | **632** | STING1 | **1162** | TMEM173 | **1692** | KCNC4 |
| 103 | LBX2 | **633** | NFKB2 | **1163** | CEBPB | **1693** | SLC2A3 |
| 104 | CHAC2 | **634** | KLF4 | **1164** | TMEM221 | **1694** | ANO8 |
| 105 | GATM | **635** | SLC45A1 | **1165** | CBY2 | **1695** | HIBCH |
| 106 | KLHDC7A | **636** | FREM2 | **1166** | LITAF | **1696** | TSC22D1 |
| 107 | APOBR | **637** | HYKK | **1167** | VWA5B2 | **1697** | TMEM121 |
| 108 | BPIFB6 | **638** | PPP1R15A | **1168** | STX11 | **1698** | ZBTB12 |
| 109 | ZMYM6 | **639** | CABP1 | **1169** | FAM169BP | **1699** | CD93 |
| 110 | HPCAL4 | **640** | CPN1 | **1170** | CPNE1 | **1700** | ELL2 |
| 111 | VIM | **641** | FOS | **1171** | HEL113 | **1701** | TDH |
| 112 | CYP2W1 | **642** | ARHGEF17 | **1172** | CLNK | **1702** | SLC12A4 |
| 113 | SKA2 | **643** | FAM33A | **1173** | KYNU | **1703** | RCOR2 |
| 114 | KIF5A | **644** | PAK6 | **1174** | OTOS | **1704** | GALK2 |
| 115 | GK2 | **645** | DUSP14 | **1175** | DEFB103A; DEFB103B | **1705** | DYRK3 |
| 116 | RPH3A | **646** | B3GALNT2 | **1176** | FBXO27 | **1706** | USHBP1 |
| 117 | HASPIN | **647** | GSG2 | **1177** | SPATA31D3 | **1707** | FAM75D3 |
| 118 | MLKL | **648** | NLRP6 | **1178** | FAM13A | **1708** | PTH2 |
| 119 | PTRH2 | **649** | IFT70B | **1179** | PRKCZ | **1709** | ENO2 |
| 120 | GDF15 | **650** | SP6 | **1180** | KLF14 | **1710** | LYRM7 |
| 121 | HS3ST3B1 | **651** | SPEG | **1181** | CSRP1 | **1711** | HCN4 |
| 122 | FRAT1 | **652** | GTF3C1 | **1182** | SPNS3 | **1712** | VAV1 |
| 123 | B9D1 | **653** | XRCC3 | **1183** | PRRX2 | **1713** | NRIP2 |
| 124 | NPHP4 | **654** | TRIM46 | **1184** | SLC51B | **1714** | TTC39B |
| 125 | DUSP2 | **655** | SYTL4 | **1185** | AK1 | **1715** | EVPL |
| 126 | LY9 | **656** | CCL17 | **1186** | PABPC1 | **1716** | SLC38A7 |
| 127 | HS3ST1 | **657** | OXSR1 | **1187** | OSR1 | **1717** | TBX2 |
| 128 | PGPEP1 | **658** | SHISA3 | **1188** | FABP1 | **1718** | FOXL2 |
| 129 | IQCC | **659** | VWC2 | **1189** | PPM1J | **1719** | EFNA4 |
| 130 | TMEM218 | **660** | TNIP1 | **1190** | SLC1A6 | **1720** | NFATC4 |
| 131 | HCRT | **661** | HDHD3 | **1191** | RNF138 | **1721** | NARF |
| 132 | MTNR1B | **662** | LCTL | **1192** | EMX1 | **1722** | CLEC4D |
| 133 | GUCA1B | **663** | TTC12 | **1193** | GCK | **1723** | MAP4K2 |
| 134 | RIPPLY3 | **664** | LMNA | **1194** | SUN2 | **1724** | PNPLA5 |
| 135 | HIPK1 | **665** | TTC9C | **1195** | FBH1 | **1725** | FBXO18 |
| 136 | IGFBP5 | **666** | ZYX | **1196** | TRIM66 | **1726** | WDR76 |
| 137 | FNDC4 | **667** | TMCC3 | **1197** | GGN | **1727** | FAM89A |
| 138 | SCG3 | **668** | CTTNBP2NL | **1198** | CNPY4 | **1728** | GIMAP1 |
| 139 | ADAM8 | **669** | CCDC142 | **1199** | RAMP3 | **1729** | COL2A1 |
| 140 | SLC2A1 | **670** | SLC39A3 | **1200** | CCDC120 | **1730** | CMA1 |
| 141 | FUT10 | **671** | SLX1A; SLX1B | **1201** | SLX1B | **1731** | PHYHIP |
| 142 | SEMA6D | **672** | ACOT11 | **1202** | HSPBAP1 | **1732** | BBS10 |
| 143 | ZMAT1 | **673** | CDR2 | **1203** | MBD1 | **1733** | CCDC88C |
| 144 | POLR1G | **674** | CAST | **1204** | RFTN2 | **1734** | TMEM132A |
| 145 | KRT18 | **675** | RASL10B | **1205** | KLHL6 | **1735** | MAPK6 |
| 146 | KLF5 | **676** | CKLF | **1206** | CXCL1 | **1736** | PPM1E |
| 147 | PPM1M | **677** | CCDC6 | **1207** | POMT1 | **1737** | MSH6 |
| 148 | SERTAD1 | **678** | FNDC7 | **1208** | CIRBP | **1738** | SLC38A2 |
| 149 | SIRT4 | **679** | BID | **1209** | SLITRK6 | **1739** | SLC13A2 |
| 150 | FGF11 | **680** | FGF1D | **1210** | RAB3IL1 | **1740** | TAF9 |
| 151 | RASGRP3 | **681** | MAP3K5 | **1211** | DIO3 | **1741** | SOX10 |
| 152 | ARRB1 | **682** | ADGRF3 | **1212** | BBC3 | **1742** | ASIC2 |
| 153 | ACCN1 | **683** | ATF4 | **1213** | PPARGC1A | **1743** | PARP11 |
| 154 | ID4 | **684** | CBFA2T3 | **1214** | LHX3 | **1744** | SDCBP2 |
| 155 | PELI3 | **685** | SLC6A20 | **1215** | SIT1 | **1745** | ERN2 |
| 156 | GNL3 | **686** | RFX4 | **1216** | STAB2 | **1746** | TFCP2 |
| 157 | CCR3 | **687** | SYT17 | **1217** | ASNS | **1747** | PLCG2 |
| 158 | S100A8 | **688** | DESI2 | **1218** | PPPDE1 | **1748** | ZNF112 |
| 159 | ZFP112 | **689** | IL4R | **1219** | RANBP3L | **1749** | CHEK2 |
| 160 | TET1 | **690** | ZFP36L1 | **1220** | FANCD2 | **1750** | MTHFD1L |
| 161 | PCDH9 | **691** | CD300LF | **1221** | CENPL | **1751** | SLC8A2 |
| 162 | CMTM8 | **692** | B3GNTL1 | **1222** | B3GNT8 | **1752** | TUBB3 |
| 163 | PGM1 | **693** | TMEM54 | **1223** | CBX7 | **1753** | GABRB2 |
| 164 | ITGA1 | **694** | HAVCR2 | **1224** | FUT9 | **1754** | MZF1 |
| 165 | CCN1 | **695** | CYR61 | **1225** | CUL9 | **1755** | CTDP1 |
| 166 | ROBO4 | **696** | COA4 | **1226** | KRTAP5-3 | **1756** | TENM4 |
| 167 | ODZ4 | **697** | KLK10 | **1227** | DNAAF10 | **1757** | NHERF4 |
| 168 | PDZD3 | **698** | NCAPD3 | **1228** | GSX1 | **1758** | STK17B |
| 169 | MAF | **699** | LYPLAL1 | **1229** | RAMP2 | **1759** | TNIP2 |
| 170 | STARD8 | **700** | NEURL1B | **1230** | FAS | **1760** | FASN |
| 171 | MUC6 | **701** | CD19 | **1231** | FGD2 | **1761** | RIMS1 |
| 172 | COL25A1 | **702** | TMEM26 | **1232** | SLC28A3 | **1762** | KCNE4 |
| 173 | TMEM107 | **703** | ERCC1 | **1233** | NPC1 | **1763** | ANKRD1 |
| 174 | KCNK12 | **704** | STMN4 | **1234** | TMPRSS2 | **1764** | FAM168A |
| 175 | ATP6V1C2 | **705** | SLC45A4 | **1235** | PIGY | **1765** | CFHR2 |
| 176 | CASP6 | **706** | CBX6 | **1236** | MAP3K14 | **1766** | CAMK2N2 |
| 177 | THRA | **707** | RIBC1 | **1237** | TNP1 | **1767** | PRR15 |
| 178 | LOC222171 | **708** | PHLDA1 | **1238** | RTN4RL1 | **1768** | GPR176 |
| 179 | SMAD1 | **709** | SLC2A12 | **1239** | PDE6G | **1769** | DUSP9 |
| 180 | ADGRA2 | **710** | CBR1 | **1240** | HMX3 | **1770** | PELP1 |
| 181 | HSPA12B | **711** | ATP23 | **1241** | XRCC6BP1 | **1771** | SLC29A2 |
| 182 | NEUROG3 | **712** | RAB11FIP3 | **1242** | FANCC | **1772** | MDM2 |
| 183 | CIT | **713** | QRICH2 | **1243** | ST6GAL2 | **1773** | PTP4A3 |
| 184 | PLCXD2 | **714** | PPP1R14A | **1244** | SLC7A8 | **1774** | DGKH |
| 185 | TMEM59L | **715** | DEPDC1B | **1245** | CDSN | **1775** | FAM120B |
| 186 | KCNK1 | **716** | RHCG | **1246** | SFXN4 | **1776** | PACC1 |
| 187 | PDLIM7 | **717** | MAVS | **1247** | ZGPAT | **1777** | GPR156 |
| 188 | KCNJ12 | **718** | KCNJ17 | **1248** | SH3BP1 | **1778** | LINGO4 |
| 189 | FAM110C | **719** | PPP1R27 | **1249** | GPRC5A | **1779** | B4GALT6 |
| 190 | DENND2A | **720** | LRRC8C | **1250** | TBC1D31 | **1780** | WDR67 |
| 191 | CPA4 | **721** | AMELX | **1251** | ST8SIA5 | **1781** | SEL1L |
| 192 | SAMSN1 | **722** | CYP17A1 | **1252** | PIGF | **1782** | PFKFB4 |
| 193 | TRIL | **723** | DNAAF4 | **1253** | DYX1C1 | **1783** | PROK1 |
| 194 | CD274 | **724** | RTN3 | **1254** | CARS1 | **1784** | CARS |
| 195 | KLK11 | **725** | HOXC6 | **1255** | TINAGL1 | **1785** | TPSB2 |
| 196 | B3GALNT1 | **726** | OGT | **1256** | MANSC1 | **1786** | NEMP2 |
| 197 | FOXI2 | **727** | SIVA1 | **1257** | NR4A1 | **1787** | LYPD3 |
| 198 | CMTM5 | **728** | SLC19A2 | **1258** | CHRNA4 | **1788** | NAT14 |
| 199 | S100A9 | **729** | INF2 | **1259** | DNAJC28 | **1789** | PDE6D |
| 200 | CLCNKB | **730** | SMIM10L2A | **1260** | BGLAP | **1790** | DAPK2 |
| 201 | GULP1 | **731** | GUCY2F | **1261** | RALGDS | **1791** | FLJ00185 |
| 202 | GLT1D1 | **732** | CLRN3 | **1262** | PLIN2 | **1792** | IL1R1 |
| 203 | NGF | **733** | PEX26 | **1263** | CRTC3 | **1793** | GPR119 |
| 204 | TMEM41A | **734** | VIPR1 | **1264** | TMEM158 | **1794** | ANGPTL6 |
| 205 | NRM | **735** | MKKS | **1265** | EDAR | **1795** | KIFC2 |
| 206 | FGF21 | **736** | GBF1 | **1266** | CEL | **1796** | CELL |
| 207 | HBEGF | **737** | CXCL10 | **1267** | FOSB | **1797** | ATAT1 |
| 208 | CRB2 | **738** | CAMK1G | **1268** | MEF2D | **1798** | TMEM79 |
| 209 | ART4 | **739** | NOB1 | **1269** | DO | **1799** | DLEU7 |
| 210 | NOS3 | **740** | NANOS3 | **1270** | H2BC21 | **1800** | MTHFSD |
| 211 | PRDM16 | **741** | GHRH | **1271** | KLF3 | **1801** | TBXA2R |
| 212 | SPRR2E | **742** | SENP8 | **1272** | CC2D2A | **1802** | PTAFR |
| 213 | CIMIP2B | **743** | FAM166B | **1273** | HDAC11 | **1803** | TTLL1 |
| 214 | CHST14 | **744** | TRIM16 | **1274** | PWWP3B | **1804** | ALKBH7 |
| 215 | PLCD1 | **745** | ENGASE | **1275** | SMYD1 | **1805** | VPREB1 |
| 216 | SAMD5 | **746** | GRIN2C | **1276** | KRTAP9-1 | **1806** | SCGB1A1 |
| 217 | WDR25 | **747** | WDR43 | **1277** | LRRC25 | **1807** | CRYGA |
| 218 | BAZ1A | **748** | COL9A2 | **1278** | NEK4 | **1808** | CD4 |
| 219 | KCND3 | **749** | HIVEP3 | **1279** | TMEM169 | **1809** | HIVEP2 |
| 220 | CDR2L | **750** | RNF32 | **1280** | SLCO2B1 | **1810** | DDIT3 |
| 221 | CYS1 | **751** | ZKSCAN2 | **1281** | CHIC2 | **1811** | TERT |
| 222 | ANGPTL3 | **752** | AATK | **1282** | SLC25A19 | **1812** | DKFZp667O1614 |
| 223 | DUSP15 | **753** | SNX22 | **1283** | SEMA3F | **1813** | PTCD3 |
| 224 | MYCBPAP | **754** | MAPK8IP1 | **1284** | TIPARP | **1814** | CIITA |
| 225 | LAIR1 | **755** | CTF1 | **1285** | LRRC38 | **1815** | RBM12B |
| 226 | ENOX2 | **756** | PFAS | **1286** | CASP4 | **1816** | MEX3A |
| 227 | MDH1B | **757** | TMEM64 | **1287** | LRRC31 | **1817** | GUCY1B2 |
| 228 | PLXNB3 | **758** | PRPS1L1 | **1288** | TJP3 | **1818** | RAD1 |
| 229 | RNASE6 | **759** | EPYC | **1289** | AANAT | **1819** | MDFI |
| 230 | ANGPTL4 | **760** | CITED1 | **1290** | ROCK2 | **1820** | BIRC3 |
| 231 | CLCA2 | **761** | FAM20C | **1291** | SH3BP4 | **1821** | CAPN10 |
| 232 | TCF19 | **762** | ARVCF | **1292** | SLC25A22 | **1822** | PRKCB |
| 233 | IGLON5 | **763** | PTPN22 | **1293** | USP27X | **1823** | PPM1K |
| 234 | PBX1 | **764** | MMP11 | **1294** | DCLK3 | **1824** | RHOB |
| 235 | ZSWIM3 | **765** | THBS1 | **1295** | HSPA1L | **1825** | MICALL2 |
| 236 | NOXO1 | **766** | ZMYM1 | **1296** | CCHCR1 | **1826** | SPRR4 |
| 237 | MUC5AC | **767** | AMER1 | **1297** | IFNA2 | **1827** | ACMSD |
| 238 | AMZ1 | **768** | KIAA1950 | **1298** | NLRC4 | **1828** | XPO1 |
| 239 | SYBU | **769** | RRM2B | **1299** | TPM4 | **1829** | KLHL21 |
| 240 | TACR1 | **770** | SAV1 | **1300** | SH3BGR | **1830** | SGMS2 |
| 241 | BHLHE40 | **771** | PLCH1 | **1301** | CPB1 | **1831** | TCF15 |
| 242 | SDCCAG8 | **772** | COX7C | **1302** | ZFP30 | **1832** | CRYAA |
| 243 | TAF6L | **773** | RAPGEFL1 | **1303** | ARMCX6 | **1833** | TBC1D15 |
| 244 | OTUB2 | **774** | pp9457 | **1304** | GRHL1 | **1834** | GAPDHS |
| 245 | TCEAL1 | **775** | GPR162 | **1305** | ACSL4 | **1835** | DKKL1 |
| 246 | WFDC1 | **776** | FBXL16 | **1306** | CPE | **1836** | GDF10 |
| 247 | FYN | **777** | RORA | **1307** | NKX6-1 | **1837** | CAPZB |
| 248 | ACOT9 | **778** | MMP10 | **1308** | BCL2L1 | **1838** | DEFB119 |
| 249 | PROCR | **779** | PTCHD3 | **1309** | ENDOU | **1839** | SLC4A4 |
| 250 | SGSM1 | **780** | CARD6 | **1310** | CDH5 | **1840** | SH2D6 |
| 251 | TACSTD2 | **781** | SHD | **1311** | USP40 | **1841** | SAP30 |
| 252 | TEAD4 | **782** | MASP1 | **1312** | RPAIN | **1842** | STX2 |
| 253 | CTXN3 | **783** | MYH7 | **1313** | CD22 | **1843** | BANF2 |
| 254 | OC90 | **784** | EMX2 | **1314** | RILPL1 | **1844** | NT5DC3 |
| 255 | FAM133B | **785** | TTR | **1315** | ADCYAP1 | **1845** | CNTROB |
| 256 | C1QL2 | **786** | ADII | **1316** | PDGFA | **1846** | SIRPA |
| 257 | ADAM30 | **787** | PIF1 | **1317** | CTTNBP2 | **1847** | SALL4 |
| 258 | RETREG1 | **788** | PRMT2 | **1318** | FZD9 | **1848** | FZD3 |
| 259 | PLA2G2D | **789** | WDR81 | **1319** | Dnahc11 | **1849** | SEPTIN4 |
| 260 | SEPT4 | **790** | GCNT2 | **1320** | GNA14 | **1850** | RNF152 |
| 261 | CDK5R1 | **791** | TMPPE | **1321** | PLD6 | **1851** | MLLT11 |
| 262 | AF1Q | **792** | ELF3 | **1322** | MMD2 | **1852** | LIG3 |
| 263 | LRIG3 | **793** | GCM2 | **1323** | NICN1 | **1853** | ARF6 |
| 264 | PTPN23 | **794** | GATA1 | **1324** | IGFALS | **1854** | SRGN |
| 265 | TYW3 | **795** | WDFY4 | **1325** | PTPN12 | **1855** | IMPA2 |
| 266 | CD244 | **796** | SYCN | **1326** | MYO5C | **1856** | DRD3 |
| 267 | AKNAD1 | **797** | SLC8A1 | **1327** | GABRE | **1857** | MTARC1 |
| 268 | NOSTRIN | **798** | PHLDB1 | **1328** | LDB2 | **1858** | PPP1R18 |
| 269 | GPER1 | **799** | GPR30 | **1329** | FAM167B | **1859** | TEK |
| 270 | SDC1 | **800** | PYCARD | **1330** | PLCB2 | **1860** | NINL |
| 271 | FOSL2 | **801** | RFLNA | **1331** | DEFB115 | **1861** | ADPRHL1 |
| 272 | PLAAT5 | **802** | HRASLS5 | **1332** | PLA2G4C | **1862** | TFAP2B |
| 273 | APLP2 | **803** | APPL2 | **1333** | RASSF8 | **1863** | SLC35F1 |
| 274 | TFAP2E | **804** | GPR171 | **1334** | C1RL | **1864** | VEGFC |
| 275 | KRT16 | **805** | SRD5A2 | **1335** | KLF17 | **1865** | HNF4G |
| 276 | CEBPD | **806** | IL15 | **1336** | QRFP | **1866** | KDM3A |
| 277 | YIPF7 | **807** | ODAD3 | **1337** | CCDC151 | **1867** | FRMD4B |
| 278 | TFF1 | **808** | TOGARAM2 | **1338** | WSCD1 | **1868** | IGSF9 |
| 279 | GPRC6A | **809** | GCH1 | **1339** | FGF9 | **1869** | PTGFR |
| 280 | IKBKE | **810** | NR4A2 | **1340** | TRMT61A | **1870** | EXOC3L2 |
| 281 | CKS2 | **811** | BEST2 | **1341** | FABP7 | **1871** | ATM |
| 282 | CD7 | **812** | SLC6A14 | **1342** | ANKRD2 | **1872** | PRSS37 |
| 283 | STK40 | **813** | SH2D5 | **1343** | DOT1L | **1873** | DEFB1 |
| 284 | LECT2 | **814** | CLEC4G | **1344** | TRIM37 | **1874** | SLC27A3 |
| 285 | SLC18A1 | **815** | VAT1 | **1345** | ARHGAP6 | **1875** | RUNX1 |
| 286 | RPA3 | **816** | DDC | **1346** | UTY | **1876** | PUS3 |
| 287 | OSM | **817** | SLC17A8 | **1347** | HERPUD1 | **1877** | NCAPD2 |
| 288 | IL7R | **818** | PLP1 | **1348** | XKR8 | **1878** | CACNA1S |
| 289 | H2AC25 | **819** | ASGR1 | **1349** | PLPP7 | **1879** | PPP1R1B |
| 290 | S100A3 | **820** | MAPK15 | **1350** | KIF26A | **1880** | SPO11 |
| 291 | RYBP | **821** | FBXL8 | **1351** | INSM2 | **1881** | NPR3 |
| 292 | NPRC | **822** | MYLIP | **1352** | FGF14 | **1882** | FGF1B |
| 293 | MYL7 | **823** | STC1 | **1353** | OSGIN1 | **1883** | PITPNM2 |
| 294 | HECW2 | **824** | MYOZ3 | **1354** | FMN2 | **1884** | SYT13 |
| 295 | GDAP1L1 | **825** | WDR6 | **1355** | PDE4B | **1885** | IRX3 |
| 296 | TLR2 | **826** | CIB4 | **1356** | CEP85 | **1886** | GOLGA7B |
| 297 | MFSD4A | **827** | MFSD4 | **1357** | SRXN1 | **1887** | FAM156A; FAM156B |
| 298 | RAPSN | **828** | CYTH1 | **1358** | ACRBP | **1888** | MTFR2 |
| 299 | ZSCAN22 | **829** | IDUA | **1359** | RGS16 | **1889** | HAVCR1 |
| 300 | EFS | **830** | RECQL | **1360** | SSBP1 | **1890** | MFAP2 |
| 301 | NUPR1 | **831** | FJX1 | **1361** | RRAD | **1891** | IGDCC3 |
| 302 | KRTAP4-16 | **832** | CACNA1H | **1362** | SLC17A2 | **1892** | SLC34A1 |
| 303 | SLCO4A1 | **833** | CALHM2 | **1363** | SGCZ | **1893** | SEC14L1 |
| 304 | RGS3 | **834** | DENND4A | **1364** | SGTB | **1894** | SYN3 |
| 305 | NEB | **835** | ANXA2 | **1365** | STAMBPL1 | **1895** | COL11A1 |
| 306 | CO11A1 | **836** | IL22RA1 | **1366** | ASTN1 | **1896** | BATF |
| 307 | ATP13A3 | **837** | TNRC18 | **1367** | ADAMTS16 | **1897** | S100A5 |
| 308 | KRT20 | **838** | KRTAP8-1 | **1368** | SFRP1 | **1898** | CHST10 |
| 309 | DPF1 | **839** | DMAC2L | **1369** | HDDC3 | **1899** | GPRASP2 |
| 310 | MAP3K13 | **840** | SLC9A4 | **1370** | VSTM2L | **1900** | TSTD2 |
| 311 | JUN | **841** | ZPBP2 | **1371** | RUNX1T1 | **1901** | MTG8 |
| 312 | PCDHB8 | **842** | ASAP3 | **1372** | P2RX5 | **1902** | SERPINE3 |
| 313 | KIF12 | **843** | PDXP | **1373** | TRAPPC2 | **1903** | ZNF547 |
| 314 | CPNE8 | **844** | KRT34 | **1374** | KIF20A | **1904** | ARHGDIG |
| 315 | GNAS | **845** | PLA2G4B | **1375** | NTHL1 | **1905** | IER2 |
| 316 | IER3 | **846** | PRG1 | **1376** | FKBP5 | **1906** | CLIC3 |
| 317 | MYO1E | **847** | OMP | **1377** | ANKRD24 | **1907** | MAGEL2 |
| 318 | HTR2A | **848** | DOC2A | **1378** | VASN | **1908** | ARMC9 |
| 319 | SLC34A3 | **849** | OR2H2 | **1379** | SRPK3 | **1909** | TAS1R1 |
| 320 | FAM171A2 | **850** | BARHL2 | **1380** | TMEM53 | **1910** | PDZD7 |
| 321 | SCYL2 | **851** | KLK7 | **1381** | KLK6 | **1911** | FFAR4 |
| 322 | SNAI2 | **852** | SAXO2 | **1382** | GPR158 | **1912** | PAX7 |
| 323 | TCF21 | **853** | PCDHGA7 | **1383** | RHOU | **1913** | OSBPL6 |
| 324 | KCNH6 | **854** | PTGER4 | **1384** | GDF9 | **1914** | SYN2 |
| 325 | RNF144B | **855** | CGA | **1385** | TAAR2 | **1915** | PRL |
| 326 | CLCA1 | **856** | ADAMTSL4 | **1386** | FAM53B | **1916** | TLE6 |
| 327 | LYL1 | **857** | WRAP73 | **1387** | WDR8 | **1917** | CBFA2T2 |
| 328 | LMO2 | **858** | MAFB | **1388** | SAMD4A | **1918** | CDK6 |
| 329 | ITGA5 | **859** | DUSP1 | **1389** | ELK3 | **1919** | GPRC5D |
| 330 | GPR161 | **860** | OPA3 | **1390** | FADS6 | **1920** | FAM107B |
| 331 | TSPAN32 | **861** | SPATA21 | **1391** | KRT23 | **1921** | FSTL5 |
| 332 | STON1 | **862** | RTL5 | **1392** | AIF1 | **1922** | PTCHD1 |
| 333 | PKD1L2 | **863** | LRRC3 | **1393** | TMX4 | **1923** | GUCY1A1 |
| 334 | LRRC56 | **864** | THADA | **1394** | MFSD9 | **1924** | LMO3 |
| 335 | NUSAP1 | **865** | COL11A2 | **1395** | ANXA3 | **1925** | LMOD3 |
| 336 | KLHL36 | **866** | CLIP3 | **1396** | NR3C2 | **1926** | EFCAB3 |
| 337 | TGM5 | **867** | SLC24A4 | **1397** | BCAR1 | **1927** | SOWAHA |
| 338 | KCNN1 | **868** | SOHLH2 | **1398** | RTN4R | **1928** | SETDB2 |
| 339 | JAM2 | **869** | DDX11 | **1399** | NLRC3 | **1929** | PARS2 |
| 340 | RNF125 | **870** | PRSS3 | **1400** | NEDD4L | **1930** | ACTL9 |
| 341 | FSHB | **871** | ADGRD1 | **1401** | TCERG1 | **1931** | H2AC11; H2AC13; H2AC15; H2AC16; H2AC17 |
| 342 | HIST1H2AK | **872** | ENKUR | **1402** | ZC3H12A | **1932** | GANC |
| 343 | PRLR | **873** | SLAMF6 | **1403** | SSTR3 | **1933** | BRSK2 |
| 344 | DENND1C | **874** | RILPL2 | **1404** | DRD5 | **1934** | MMP3 |
| 345 | PDZD4 | **875** | IRS4 | **1405** | CRYBB2 | **1935** | CCL18 |
| 346 | SLC35G3 | **876** | SHB | **1406** | SMPDL3B | **1936** | LY6G6C |
| 347 | SMAD3 | **877** | NCAN | **1407** | VEPH1 | **1937** | PLK3 |
| 348 | GPR135 | **878** | POU3F1 | **1408** | KLHL38 | **1938** | EZR |
| 349 | SMAD9 | **879** | LRRC20 | **1409** | RAD51C | **1939** | CCRL2 |
| 350 | CCR6 | **880** | ANKRD34B | **1410** | ZFHX2 | **1940** | HUNK |
| 351 | TTC24 | **881** | OVOL1 | **1411** | RASSF1 | **1941** | MYOM3 |
| 352 | WDR89 | **882** | PPP1R14D | **1412** | SRL | **1942** | TRANK1 |
| 353 | REPS1 | **883** | EFNA3 | **1413** | GAS1 | **1943** | SLC26A6 |
| 354 | SH2D2A | **884** | CLDN9 | **1414** | CLIC4 | **1944** | GTF3C6 |
| 355 | TMEM150B | **885** | INPP5J | **1415** | GRIN2D | **1945** | TRIM15 |
| 356 | POLA1 | **886** | POU3F3 | **1416** | TMEM270 | **1946** | RTP3 |
| 357 | DIRAS1 | **887** | IL36B | **1417** | ATP2B4 | **1947** | ATP2B2 |
| 358 | MTMR7 | **888** | CPNE6 | **1418** | PDIA5 | **1948** | RGS14 |
| 359 | TENT5B | **889** | GTDC1 | **1419** | KRT32 | **1949** | CRKL |
| 360 | NKX2-1 | **890** | TTF1 | **1420** | SPDEF | **1950** | ANGPTL7 |
| 361 | SHF | **891** | ITGA6 | **1421** | IL1B | **1951** | IL27RA |
| 362 | NGRN | **892** | GTSF1L | **1422** | BBS12 | **1952** | CLEC10A |
| 363 | SLC4A1 | **893** | IL36RN | **1423** | SGSH | **1953** | BEND5 |
| 364 | PI16 | **894** | SLC10A7 | **1424** | ARHGEF10L | **1954** | HCN2 |
| 365 | INSC | **895** | SQSTM1 | **1425** | FATE1 | **1955** | PHF19 |
| 366 | CAND2 | **896** | KCTD19 | **1426** | CDKN2B | **1956** | RAB17 |
| 367 | BTG2 | **897** | STRA6 | **1427** | PODNL1 | **1957** | CACNB1 |
| 368 | DSP | **898** | CAPN2 | **1428** | FKBP11 | **1958** | H1-7 |
| 369 | VGLL4 | **899** | CDK5R2 | **1429** | NEFM | **1959** | SLURP1 |
| 370 | VCAN | **900** | NCBP2 | **1430** | EXO5 | **1960** | CLDN23 |
| 371 | KCNA2 | **901** | CDCA4 | **1431** | DEUP1 | **1961** | MYT1 |
| 372 | PKMYT1 | **902** | SSPOP | **1432** | FAT3 | **1962** | PER3 |
| 373 | DIPK1B | **903** | OLFM3 | **1433** | NKX2-4 | **1963** | TUBB4B |
| 374 | CYP2U1 | **904** | CCDC157 | **1434** | GDF5 | **1964** | ASB15 |
| 375 | SH2D7 | **905** | TEKT1 | **1435** | EMILIN2 | **1965** | NKAIN3 |
| 376 | EFCAB8 | **906** | CYB5B | **1436** | DNAI7 | **1966** | SPIN2A |
| 377 | SPIN2B | **907** | LSM11 | **1437** | MEF2C | **1967** | ADORA2B |
| 378 | DUOXA1 | **908** | NIP | **1438** | TLNRD1 | **1968** | CCN2 |
| 379 | CTGF | **909** | RAB9B | **1439** | PF4 | **1969** | ARHGAP4 |
| 380 | TMEM86A | **910** | BCL2L15 | **1440** | RXFP4 | **1970** | USP12 |
| 381 | POU4F1 | **911** | DUSP13A | **1441** | DUSP13B | **1971** | SLC25A30 |
| 382 | GCM1 | **912** | NCAPG | **1442** | CDH15 | **1972** | RASGEF1B |
| 383 | HSPA5 | **913** | IL1R2 | **1443** | CPT1C | **1973** | HECTD2 |
| 384 | APEX1 | **914** | HAP1 | **1444** | KRT19 | **1974** | MYPOP |
| 385 | FNIP2 | **915** | SUCNR1 | **1445** | TBC1D9 | **1975** | CEP290 |
| 386 | LRRC23 | **916** | MEGF11 | **1446** | RTL6 | **1976** | LRRN3 |
| 387 | CNPY1 | **917** | DNA2 | **1447** | ELL | **1977** | RASGRP2 |
| 388 | NLRC5 | **918** | TSPY26P | **1448** | LTN1 | **1978** | PLK2 |
| 389 | TWNK | **919** | FAM25C | **1449** | SOX14 | **1979** | TRUB1 |
| 390 | IL4I1 | **920** | STARD13 | **1450** | MORN3 | **1980** | FCRL5 |
| 391 | KRTAP5-4 | **921** | SLC15A2 | **1451** | CCDC74A | **1981** | PLA2G15 |
| 392 | SLC14A2 | **922** | UGT1A1 | **1452** | DKK2 | **1982** | KMT5C |
| 393 | ALDH1A3 | **923** | FAM107A | **1453** | TU3A | **1983** | TOX3 |
| 394 | KIFC1 | **924** | PHETA1 | **1454** | ZKSCAN4 | **1984** | LAMB3 |
| 395 | PSTPIP1 | **925** | ELAVL3 | **1455** | DKFZp547J036 | **1985** | TRIP12 |
| 396 | PIM1 | **926** | KLHL30 | **1456** | PLEC | **1986** | HOXA11 |
| 397 | DPYSL5 | **927** | F7 | **1457** | ATG4D | **1987** | MED13 |
| 398 | COQ3 | **928** | ADM2 | **1458** | RABGAP1 | **1988** | CBX2 |
| 399 | RBFOX1 | **929** | F2RL1 | **1459** | CABYR | **1989** | UBXN11 |
| 400 | PWWP2A | **930** | GDF1 | **1460** | H4C16 | **1990** | ST3GAL1 |
| 401 | LY86 | **931** | KCNN2 | **1461** | MPP2 | **1991** | DLG2 |
| 402 | DPP6 | **932** | PIGW | **1462** | DNASE2 | **1992** | MLF1 |
| 403 | KNDC1 | **933** | IMMP1L | **1463** | TEAD2 | **1993** | CDH24 |
| 404 | CYP27B1 | **934** | FKTN | **1464** | REP15 | **1994** | FGF7 |
| 405 | SCD5 | **935** | ALDH1A1 | **1465** | INHBA | **1995** | BMP2 |
| 406 | GLIS3 | **936** | TBX4 | **1466** | MED28 | **1996** | PKNOX2 |
| 407 | BCL6B | **937** | SDF2L1 | **1467** | PLEKHN1 | **1997** | EGR1 |
| 408 | TYK2 | **938** | TFCP2L1 | **1468** | RPRD1A | **1998** | SGPP2 |
| 409 | VCL | **939** | HEL114 | **1469** | RIPK3 | **1999** | ANKRD60 |
| 410 | SH3BP2 | **940** | TM4SF1 | **1470** | GFRA4 | **2000** | XBP1 |
| 411 | AOC2 | **941** | ORM1 | **1471** | SFRP2 | **2001** | PRIMA1 |
| 412 | SET | **942** | POLA2 | **1472** | SLC39A14 | **2002** | IFNGR2 |
| 413 | HOXB4 | **943** | CETN1 | **1473** | SOX12 | **2003** | SOX15 |
| 414 | ADRA2C | **944** | STK36 | **1474** | HTR2B | **2004** | PIK3CB |
| 415 | ADGRE1 | **945** | PKD2L2 | **1475** | NR1D1 | **2005** | PKIB |
| 416 | CALHM6 | **946** | MKRN3 | **1476** | KLHDC8B | **2006** | VPS26C |
| 417 | MGAT1 | **947** | PPFIA2 | **1477** | NPHS2 | **2007** | TLR7 |
| 418 | ZRANB2 | **948** | TINF2 | **1478** | SERPINB12 | **2008** | ZC2HC1C |
| 419 | SESN1 | **949** | NFYA | **1479** | SKIL | **2009** | FOXP2 |
| 420 | KLHDC1 | **950** | ITPKC | **1480** | CD300LB | **2010** | CFAP58 |
| 421 | TAC1 | **951** | BNC2 | **1481** | SCARF2 | **2011** | EDIL3 |
| 422 | S100A6 | **952** | FCGR3B | **1482** | FCGR3A | **2012** | HCAR2 |
| 423 | NKX3-1 | **953** | RNF180 | **1483** | CLEC1B | **2013** | TPPP |
| 424 | FREP1 | **954** | TOP2A | **1484** | STXBP4 | **2014** | SLC39A5 |
| 425 | VANGL2 | **955** | SRBD1 | **1485** | HHATL | **2015** | SALL3 |
| 426 | MYO1F | **956** | APLF | **1486** | IL20RB | **2016** | PRRG4 |
| 427 | SSTR1 | **957** | H3C12 | **1487** | SMOC1 | **2017** | ZBTB3 |
| 428 | RELL1 | **958** | MINDY4B | **1488** | B3GAT2 | **2018** | SLC7A1 |
| 429 | PLA2G2F | **959** | ATXN2 | **1489** | CRTAM | **2019** | LPAR4 |
| 430 | AGAP2 | **960** | AIRE | **1490** | GPR4 | **2020** | THAP2 |
| 431 | RAVER2 | **961** | CACNG4 | **1491** | PRDM2 | **2021** | H1-1 |
| 432 | B3GNT4 | **962** | CCDC62 | **1492** | PAK3 | **2022** | ST6GALNAC3 |
| 433 | UTS2R | **963** | GPR143 | **1493** | ALDH18A1 | **2023** | GPATCH3 |
| 434 | SMPX | **964** | PPP1R3C | **1494** | KRIT1 | **2024** | CCM1 |
| 435 | TAT | **965** | ARHGEF19 | **1495** | PCDHA9 | **2025** | LARGE2 |
| 436 | GYLTL1B | **966** | RSPO1 | **1496** | TBCK | **2026** | ZC3H8 |
| 437 | SYT10 | **967** | CITED4 | **1497** | NKTR | **2027** | G0S2 |
| 438 | LAG3 | **968** | SLC25A25 | **1498** | HS6ST3 | **2028** | TMCC2 |
| 439 | PFN3 | **969** | WDR38 | **1499** | ZBTB49 | **2029** | SPRN |
| 440 | SPATC1 | **970** | FOXO4 | **1500** | C1QTNF2 | **2030** | TMEM145 |
| 441 | ARF4 | **971** | ABL2 | **1501** | ADAMTS10 | **2031** | UPF3B |
| 442 | BARX2 | **972** | ERBB2 | **1502** | HER-2 | **2032** | ITGA10 |
| 443 | FLT3 | **973** | ANKRD26 | **1503** | TMEM204 | **2033** | RTBDN |
| 444 | XDH | **974** | OGDHL | **1504** | PTPRO | **2034** | PTPRU |
| 445 | NGLY1 | **975** | TUBB2A | **1505** | RAB11FIP4 | **2035** | COL12A1 |
| 446 | KRT7 | **976** | LRRC14 | **1506** | EPHB6 | **2036** | CLASP1 |
| 447 | MAST1 | **977** | POLE | **1507** | PSMB9 | **2037** | CX3CR1 |
| 448 | PRSS55 | **978** | GFI1B | **1508** | AQP2 | **2038** | LPCAT1 |
| 449 | DDIT4 | **979** | PKDREJ | **1509** | WNT9A | **2039** | LRRC3B |
| 450 | CFD | **980** | CMTR2 | **1510** | SERTAD4 | **2040** | ACOT2 |
| 451 | ANKRD33B | **981** | CDC25B | **1511** | OSMR | **2041** | MMP28 |
| 452 | RFTN1 | **982** | WEE1 | **1512** | OXT | **2042** | MPHOSPH9 |
| 453 | TMPRSS13 | **983** | CD40 | **1513** | CD83 | **2043** | DUSP18 |
| 454 | RARG | **984** | SNX4 | **1514** | TMEM161A | **2044** | OTUB1 |
| 455 | KCNK10 | **985** | NQO2 | **1515** | MED23 | **2045** | HRH1 |
| 456 | SLC10A2 | **986** | NECAB2 | **1516** | RACGAP1 | **2046** | TBC1D16 |
| 457 | CHRNA2 | **987** | RAD50 | **1517** | TNFRSF25 | **2047** | IDO1 |
| 458 | VPS13B | **988** | TEX9 | **1518** | LEFTY1 | **2048** | KCNH2 |
| 459 | WNT2 | **989** | HOXD3 | **1519** | NOCT | **2049** | GINS3 |
| 460 | ATOH8 | **990** | MYL11 | **1520** | HSD17B12 | **2050** | FBXO43 |
| 461 | ATF7IP | **991** | FBXL5 | **1521** | SGSM2 | **2051** | MYO1H |
| 462 | ASXL1 | **992** | CDKL5 | **1522** | HYDIN | **2052** | GUCY2C |
| 463 | STAR | **993** | KLHL24 | **1523** | CMKLR2 | **2053** | THY1 |
| 464 | FHAD1 | **994** | RCAN1 | **1524** | DUSP10 | **2054** | PREX2 |
| 465 | SYNPO | **995** | STAP1 | **1525** | SPSB4 | **2055** | FASTKD1 |
| 466 | IKZF2 | **996** | TUB | **1526** | PBK | **2056** | CNTD1 |
| 467 | SLC12A1 | **997** | HK2 | **1527** | KLK2 | **2057** | PSORS1C2 |
| 468 | SPR1 | **998** | ZCCHC8 | **1528** | ZBTB42 | **2058** | KRT33B |
| 469 | RND1 | **999** | WNT5A | **1529** | ALPI | **2059** | FCGR1A |
| 470 | LGR5 | **1000** | GPR49 | **1530** | CHKA | **2060** | LAMP2 |
| 471 | SPSB1 | **1001** | KRT1 | **1531** | HCRTR1 | **2061** | ACOT6 |
| 472 | TCF23 | **1002** | MYPN | **1532** | C2CD3 | **2062** | SREBF2 |
| 473 | TTC22 | **1003** | PBX2 | **1533** | ANKLE1 | **2063** | TRO |
| 474 | LRP8 | **1004** | DCAF17 | **1534** | PDGFB | **2064** | KCNC3 |
| 475 | ABCD2 | **1005** | CFAP100 | **1535** | CORO2A | **2065** | GORASP1 |
| 476 | RNF144A | **1006** | LILRB3 | **1536** | PLB1 | **2066** | AMPD3 |
| 477 | FAM110A | **1007** | ZC4H2 | **1537** | GRHL2 | **2067** | NKAIN4 |
| 478 | PLA2R1 | **1008** | CEND1 | **1538** | CHIC1 | **2068** | CSTA |
| 479 | ALX4 | **1009** | NAV2 | **1539** | LZTFL1 | **2069** | HEATR5B |
| 480 | TRIM13 | **1010** | NF1 | **1540** | HIP1R | **2070** | OLFML2B |
| 481 | ZC3H10 | **1011** | SLAMF8 | **1541** | IFIT2 | **2071** | HSPA1A |
| 482 | HEL-S-103 | **1012** | ZC3H12D | **1542** | IL1RL2 | **2072** | MMP14 |
| 483 | CSRNP1 | **1013** | CLEC1A | **1543** | ST6GALNAC2 | **2073** | CERK |
| 484 | AOC1 | **1014** | ABP1 | **1544** | VPREB3 | **2074** | MELK |
| 485 | SLC13A5 | **1015** | MFSD8 | **1545** | DGUOK | **2075** | NPY |
| 486 | RAB31 | **1016** | OSTF1 | **1546** | PMS1 | **2076** | GJA1 |
| 487 | AGRN | **1017** | SOX7 | **1547** | MGAM | **2077** | CIBAR2 |
| 488 | SBSN | **1018** | UPF3A | **1548** | KRT6B | **2078** | DERL2 |
| 489 | ENTPD7 | **1019** | TRIM29 | **1549** | ZMYM3 | **2079** | JUND |
| 490 | TIAM2 | **1020** | NUTF2 | **1550** | UBA7 | **2080** | LOXL2 |
| 491 | RCSD1 | **1021** | DCK | **1551** | ZBED3 | **2081** | PPBP |
| 492 | SLFN5 | **1022** | SLFN12L | **1552** | MID1 | **2082** | MPG |
| 493 | SLCO1C1 | **1023** | CALCR | **1553** | ITIH4 | **2083** | RRP7A |
| 494 | RNF26 | **1024** | ZNF692 | **1554** | NSUN7 | **2084** | METTL16 |
| 495 | CYTH3 | **1025** | MEIS1 | **1555** | MESD | **2085** | TM4SF5 |
| 496 | FOXE3 | **1026** | NTRK3 | **1556** | RGL1 | **2086** | RAD51AP1 |
| 497 | TBC1D17 | **1027** | CSNK1E | **1557** | GNGT2 | **2087** | GNG8 |
| 498 | PROCA1 | **1028** | RFX2 | **1558** | PLEKHO2 | **2088** | DNAJC5 |
| 499 | SPRR1A | **1029** | MFSD14A | **1559** | HIAT1 | **2089** | UNC13B |
| 500 | PMS2 | **1030** | AP3D1 | **1560** | TIMP1 | **2090** | CENPF |
| 501 | MORN4 | **1031** | SOX18 | **1561** | NPNT | **2091** | ASB18 |
| 502 | LRRTM1 | **1032** | JADE3 | **1562** | DMBX1 | **2092** | NBEA |
| 503 | RRP12 | **1033** | STC2 | **1563** | EGR3 | **2093** | MCTP2 |
| 504 | SOCS7 | **1034** | SOCS4 | **1564** | CDCP1 | **2094** | CAV3 |
| 505 | SLC1A3 | **1035** | RNF19B | **1565** | RICTOR | **2095** | DACH1 |
| 506 | NR2F2 | **1036** | USP43 | **1566** | EPPIN | **2096** | SPINLW1 |
| 507 | KIF2A | **1037** | ARL5B | **1567** | ZDHHC14 | **2097** | FGF13 |
| 508 | STYK1 | **1038** | GPR137 | **1568** | SURF6 | **2098** | SHPRH |
| 509 | PPFIA1 | **1039** | COL22A1 | **1569** | PCGF3 | **2099** | BACH1 |
| 510 | BRIP1 | **1040** | SPEF1 | **1570** | THOC7 | **2100** | PYGL |
| 511 | SH3PXD2B | **1041** | KCTD1 | **1571** | BTG1 | **2101** | GADD45GIP1 |
| 512 | RPL11 | **1042** | TRIM68 | **1572** | RPAP1 | **2102** | THSD1 |
| 513 | FILIP1 | **1043** | IDO2 | **1573** | ERV3-1 | **2103** | CST8 |
| 514 | MMP24 | **1044** | GLRX2 | **1574** | NTF3 | **2104** | EFNB3 |
| 515 | MFSD6L | **1045** | MAP4K5 | **1575** | EVX2 | **2105** | ZNF823 |
| 516 | ZFP36 | **1046** | STIM2 | **1576** | KCNF1 | **2106** | SORBS2 |
| 517 | IPPK | **1047** | FBXW9 | **1577** | DGKE | **2107** | P2RY2 |
| 518 | BMF | **1048** | EXOC3L1 | **1578** | DCUN1D2 | **2108** | MCPH1 |
| 519 | NAPG | **1049** | PLAAT1 | **1579** | ROR2 | **2109** | TXLNA |
| 520 | NAV3 | **1050** | GRPEL1 | **1580** | AUTS2 | **2110** | EED |
| 521 | ZFYVE9 | **1051** | EFL1 | **1581** | SAMHD1 | **2111** | COL5A3 |
| 522 | CENPE | **1052** | GPR17 | **1582** | EPGN | **2112** | SF3B5 |
| 523 | SLC29A3 | **1053** | COPS7A | **1583** | TNFRSF1B | **2113** | KIF3C |
| 524 | PER1 | **1054** | CCL5 | **1584** | CPNE5 | **2114** | TIMM9 |
| 525 | ABHD8 | **1055** | CISH | **1585** | MAPKAPK2 | **2115** | MTIF2 |
| 526 | IFT56 | **1056** | TTC26 | **1586** | NUMA1 | **2116** | TWSG1 |
| 527 | VAMP5 | **1057** | NIP7 | **1587** | NOL12 | **2117** | COL3A1 |
| 528 | IFT46 | **1058** | PANK1 | **1588** | FABP4 | **2118** | HOXB2 |
| 529 | BUB3 | **1059** | TSEN2 | **1589** | MAP3K4 | **2119** | MTX3 |
| 530 | A1CF | **1060** | KLC1 | **1590** | RNGTT | **2120** | TNFRSF21 |

**Table S7 Intersection targets of databases and GEO forR-IRI**

| NO | Target | NO | Target | NO | Target | NO | Target |
| --- | --- | --- | --- | --- | --- | --- | --- |
| 1 | AGTR1 | **45** | LCN2 | **89** | HAVCR1 | **132** | ATM |
| 2 | STAT3 | **46** | NFE2L2 | **90** | CCL2 | **133** | THBD |
| 3 | IL1B | **47** | ICAM1 | **91** | KCNJ1 | **134** | FRAS1 |
| 4 | TERT | **48** | XDH | **92** | NOS3 | **135** | CD274 |
| 5 | SERPINE1 | **49** | EDN1 | **93** | HSPA5 | **136** | MYC |
| 6 | CCN2 | **50** | SLC2A1 | **94** | RUNX1 | **137** | ENO2 |
| 7 | FABP1 | **51** | GJA1 | **95** | CXCL10 | **138** | TLR2 |
| 8 | NR3C2 | **52** | FAS | **96** | SMAD3 | **139** | HSPB1 |
| 9 | SOX9 | **53** | BMP2 | **97** | TIMP1 | **140** | F3 |
| 10 | PTPRO | **54** | VCAM1 | **98** | RELA | **141** | HBEGF |
| 11 | NGF | **55** | CDKN1A | **99** | MDM2 | **142** | GDF15 |
| 12 | CD44 | **56** | EGR1 | **100** | LGALS3 | **143** | NFKBIA |
| 13 | WNT5A | **57** | GCK | **101** | PDGFB | **144** | ADM |
| 14 | JUN | **58** | BCL2L1 | **102** | CCL5 | **145** | YRDC |
| 15 | DDIT3 | **59** | ATP7B | **103** | CASP4 | **146** | SQSTM1 |
| 16 | LMNA | **60** | THBS1 | **104** | PPARGC1A | **147** | CD40 |
| 17 | MMP3 | **61** | FGF7 | **105** | HAVCR2 | **148** | SDC1 |
| 18 | METTL3 | **62** | KLF4 | **106** | FOS | **149** | MAFB |
| 19 | CXCL1 | **63** | TNFAIP3 | **107** | STING1 | **150** | CDK6 |
| 20 | SOCS3 | **64** | TNFRSF1B | **108** | TLR7 | **151** | RIPK3 |
| 21 | PLAUR | **65** | FLT3 | **109** | MASP1 | **152** | COL3A1 |
| 22 | CYP1B1 | **66** | ATF3 | **110** | HSPA1A | **153** | KRT18 |
| 23 | CSF1 | **67** | IL15 | **111** | FASN | **154** | LAMP2 |
| 24 | NPY | **68** | AIF1 | **112** | NEDD4L | **155** | SCGB1A1 |
| 25 | PIK3CB | **69** | IL1R1 | **113** | FABP7 | **156** | TRPS1 |
| 26 | NR4A1 | **70** | VEGFC | **114** | S100A9 | **157** | ATF4 |
| 27 | MMP14 | **71** | TGIF1 | **115** | MCL1 | **158** | KLF11 |
| 28 | NR4A2 | **72** | IDO1 | **116** | ATF6 | **159** | CD14 |
| 29 | FGF21 | **73** | IGF2BP2 | **117** | S100A8 | **160** | UGT1A1 |
| 30 | MLKL | **74** | EPHA2 | **118** | MAP3K5 | **161** | APEX1 |
| 31 | ENTPD1 | **75** | ANXA2 | **119** | CX3CR1 | **162** | CDH5 |
| 32 | FCGR3B | **76** | CHKA | **120** | ACSL4 | **163** | FOXP2 |
| 33 | CIITA | **77** | SLC1A3 | **121** | TEK | **164** | S1PR1 |
| 34 | ADM2 | **78** | IL7R | **122** | NRG1 | **165** | HCRT |
| 35 | PRKCB | **79** | VCAN | **123** | XBP1 | **166** | HTR2A |
| 36 | ADCYAP1 | **80** | NOL3 | **124** | BBC3 | **167** | TNFRSF12A |
| 37 | BTG2 | **81** | TYK2 | **125** | PROCR | **168** | AGRN |
| 38 | ZC3H12A | **82** | ANGPTL4 | **126** | CCR6 | **169** | PRKCZ |
| 39 | OXSR1 | **83** | GADD45A | **127** | ROCK2 | **170** | GAST |
| 40 | KCNA2 | **84** | SEMA7A | **128** | HOXA11 | **171** | TNIP1 |
| 41 | APLNR | **85** | TAC1 | **129** | PRDM16 | **172** | SIRPA |
| 42 | BDKRB1 | **86** | BDKRB2 | **130** | CD300LB | **173** | STAR |
| 43 | ADRA2C | **87** | SLC34A1 | **131** | SLC14A2 | **174** | FAM107A |
| 44 | DACH1 | **88** | SOSTDC1 |  | 0 |  | 0 |

**Table S8 MP targets of OMIM**

| NO | Target | NO | Target | NO | Target | NO | Target |
| --- | --- | --- | --- | --- | --- | --- | --- |
| 1 | OCSTAMP | **47** | SLC6A8 | **93** | AFAP1AS1 | **139** | MRC1 |
| 2 | CXCR3 | **48** | SHPK | **94** | MAPK8 | **140** | PPARG |
| 3 | CD209 | **49** | CLEC7A | **95** | MCUB | **141** | PIK3CG |
| 4 | VNN1 | **50** | AIP | **96** | FOSL2 | **142** | HIF1A |
| 5 | ARG1 | **51** | VEGFA | **97** | RIPOR2 | **143** | RASSF5 |
| 6 | RIPOR1 | **52** | MST1 | **98** | MST1R | **144** | MAEA |
| 7 | PELATON | **53** | EGLN1 | **99** | MIR505 | **145** | EMILIN1 |
| 8 | CAPG | **54** | LGALS3 | **100** | MIF | **146** | MSR1 |
| 9 | CD68 | **55** | NOS2 | **101** | CES1 | **147** | CSF1 |
| 10 | CSF2 | **56** | CSF2RB | **102** | CXCL2 | **148** | CXCL3 |
| 11 | CCL3 | **57** | CCL4 | **103** | CSF2RY | **149** | CHIT1 |
| 12 | SLC11A1 | **58** | SLC11A2 | **104** | MFSD6 | **150** | MPEG1 |
| 13 | PAM16 | **59** | MMD2 | **105** | TRIM35 | **151** | CLEC12B |
| 14 | PSTPIP2 | **60** | MACIR | **106** | MMP12 | **152** | CCR1 |
| 15 | CCL15 | **61** | CCL3L1 | **107** | CCL20 | **153** | TNF |
| 16 | SIRPA | **62** | CD5L | **108** | SCARA3 | **154** | CCL22 |
| 17 | MMD | **63** | LILRB1 | **109** | LILRB2 | **155** | MARCO |
| 18 | CSF2RA | **64** | CLEC10A | **110** | SLAMF8 | **156** | ZBP1 |
| 19 | CCL19 | **65** | CCL18 | **111** | GDF15 | **157** | HCAR2 |
| 20 | CLEC4E | **66** | CLEC4D | **112** | CCL2 | **158** | SOCS3 |
| 21 | TMEM229B | **67** | CFTR | **113** | IL17A | **159** | MS |
| 22 | ELF4 | **68** | AIFBL2 | **114** | NR1H3 | **160** | MYD88 |
| 23 | IL10 | **69** | IER3 | **115** | IL4 | **161** | IL12B |
| 24 | VAV3 | **70** | NR1H2 | **116** | IFNG | **162** | TNFSF15 |
| 25 | RA | **71** | AD1 | **117** | SPP1 | **163** | RAC1 |
| 26 | LY9 | **72** | GFI1 | **118** | EHBP1L1 | **164** | ILK |
| 27 | APP | **73** | CTNNB1 | **119** | PLCA3 | **165** | HECTD3 |
| 28 | VANGL2 | **74** | PDGFRB | **120** | SHTN1 | **166** | PHLPP1 |
| 29 | CDC42 | **75** | CD46 | **121** | APC | **167** | TJP1 |
| 30 | OCLN | **76** | IQGAP1 | **122** | STK11 | **168** | ITGAL |
| 31 | IL25 | **77** | IL27 | **123** | NFAT5 | **169** | DOCK2 |
| 32 | ELMO1 | **78** | ICOS | **124** | TBX21 | **170** | TTD7 |
| 33 | FZD6 | **79** | CELSR1 | **125** | GSK3B | **171** | PARD3 |
| 34 | PARD6A | **80** | SCRIB | **126** | MYO5A | **172** | PRKCZ |
| 35 | RUFY3 | **81** | SYNE4 | **127** | PTK7 | **173** | DPYSL2 |
| 36 | GOLGA2 | **82** | PATJ | **128** | CD2AP | **174** | RBMX |
| 37 | AMOT | **83** | MYO5B | **129** | SUN1 | **175** | BRSK1 |
| 38 | JAM3 | **84** | TLR2 | **130** | TLR4 | **176** | NLRP3 |
| 39 | NLRC4 | **85** | CASP1 | **131** | CCR5 | **177** | SIGLEC1 |
| 40 | CYBB | **86** | CCR2 | **132** | TREM2 | **178** | ABCG1 |
| 41 | ETS2 | **87** | APOE | **133** | MMP9 | **179** | ZFP36 |
| 42 | CD36 | **88** | PRKCD | **134** | DMD | **180** | IL13 |
| 43 | ABCA1 | **89** | SLC40A1 | **135** | SEMA7A | **181** | ITGAM |
| 44 | MIR223 | **90** | TLR9 | **136** | IL23A | **182** | HFE |
| 45 | NFKB1 | **91** | CD44 | **137** | SERPINA1 | **183** | DNASE2 |
| 46 | CD47 | **92** | CASP4 | **138** | CLEC5A | **184** | TLR7 |

**Table S9 MP targets of Genecards**

| NO | Target | NO | Target | NO | Target | NO | Target |
| --- | --- | --- | --- | --- | --- | --- | --- |
| 1 | CSF1 | **1048** | CSF2 | **2095** | MIF | **3142** | TNF |
| 2 | IL10 | **1049** | MSR1 | **2096** | TLR4 | **3143** | IL1B |
| 3 | IL6 | **1050** | IFNG | **2097** | CCL2 | **3144** | CSF1R |
| 4 | STAT3 | **1051** | CCR5 | **2098** | CXCL8 | **3145** | TLR2 |
| 5 | IL4 | **1052** | NLRP3 | **2099** | CCL3 | **3146** | MARCO |
| 6 | ABCA1 | **1053** | CSF2RA | **2100** | MST1 | **3147** | STAT1 |
| 7 | MST1R | **1054** | CD163 | **2101** | CCL5 | **3148** | CCR2 |
| 8 | CD4 | **1055** | MMP9 | **2102** | CCL4 | **3149** | ITGAM |
| 9 | TGFB1 | **1056** | MAEA | **2103** | MRC1 | **3150** | CRYAB |
| 10 | CD274 | **1057** | CDC42 | **2104** | CD68 | **3151** | PARD3 |
| 11 | MMD | **1058** | CCR1 | **2105** | CXCR4 | **3152** | NLRC4 |
| 12 | CCL18 | **1059** | PTGS2 | **2106** | VEGFA | **3153** | HAVCR2 |
| 13 | CSF3 | **1060** | EPHA2 | **2107** | MMP12 | **3154** | SCRIB |
| 14 | PITX3 | **1061** | CHMP4B | **2108** | IL3 | **3155** | NOS2 |
| 15 | IL18 | **1062** | PPARG | **2109** | ABCG1 | **3156** | CD14 |
| 16 | JAK2 | **1063** | NFKB1 | **2110** | CXCL12 | **3157** | IL1A |
| 17 | CERNA3 | **1064** | IL2 | **2111** | WDPCP | **3158** | APOE |
| 18 | HMGB1 | **1065** | MAPK1 | **2112** | SLC11A1 | **3159** | CCL20 |
| 19 | FASLG | **1066** | CCL22 | **2113** | CRYBB2 | **3160** | STK11 |
| 20 | MACIR | **1067** | CD86 | **2114** | MTOR | **3161** | CRYAA |
| 21 | CD47 | **1068** | NR1H3 | **2115** | PARD6A | **3162** | CD36 |
| 22 | RIPOR1 | **1069** | UNC13D | **2116** | PRF1 | **3163** | RAC1 |
| 23 | ICAM1 | **1070** | PIK3CG | **2117** | HIF1A | **3164** | TNFSF11 |
| 24 | AKT1 | **1071** | WAS | **2118** | TLR3 | **3165** | LINC02605 |
| 25 | SIRPA | **1072** | IL13 | **2119** | CYBB | **3166** | RHOA |
| 26 | CFTR | **1073** | ITGB2 | **2120** | IL34 | **3167** | NFKBIA |
| 27 | IL33 | **1074** | CXCL10 | **2121** | SYK | **3168** | CX3CR1 |
| 28 | IL12B | **1075** | PDCD1 | **2122** | HLA-DRB1 | **3169** | STAT6 |
| 29 | TREM2 | **1076** | GDF15 | **2123** | CES1 | **3170** | LIPA |
| 30 | CD44 | **1077** | HMOX1 | **2124** | APOA1 | **3171** | PTEN |
| 31 | MYD88 | **1078** | LLGL2 | **2125** | IL5 | **3172** | CCR7 |
| 32 | SCARB1 | **1079** | EGFR | **2126** | VIM | **3173** | TP53 |
| 33 | CHI3L1 | **1080** | GBF1 | **2127** | MAP2K1 | **3174** | CASP3 |
| 34 | PRKCZ | **1081** | DVL2 | **2128** | LYST | **3175** | MEFV |
| 35 | XIAP | **1082** | IL2RA | **2129** | TNFRSF1A | **3176** | JUN |
| 36 | TLR9 | **1083** | BDNF-AS | **2130** | FAS | **3177** | CCR6 |
| 37 | TLR7 | **1084** | SOCS3 | **2131** | CTNNB1 | **3178** | NFE2L2 |
| 38 | LAMP1 | **1085** | CCL11 | **2132** | CCR3 | **3179** | SPI1 |
| 39 | IRF1 | **1086** | RAB27A | **2133** | CD40 | **3180** | P2RX7 |
| 40 | GSK3B | **1087** | IRF3 | **2134** | VIPAS39 | **3181** | SIGLEC1 |
| 41 | VANGL2 | **1088** | STXBP2 | **2135** | IDO1 | **3182** | PRKCA |
| 42 | LEMD2 | **1089** | WNT5A | **2136** | KLF4 | **3183** | IL1RN |
| 43 | CXCL2 | **1090** | IRF8 | **2137** | SGPP1 | **3184** | CD74 |
| 44 | MARK2 | **1091** | NOD2 | **2138** | FOXP3 | **3185** | FUZ |
| 45 | RELA | **1092** | PIK3R1 | **2139** | IL17A | **3186** | SOD2-OT1 |
| 46 | MAF | **1093** | MIP | **2140** | LPL | **3187** | PWAR1 |
| 47 | SLC7A7 | **1094** | ICAM3 | **2141** | CAPG | **3188** | PTK2 |
| 48 | HAMP | **1095** | CD81 | **2142** | MERTK | **3189** | YAP1 |
| 49 | CXCL1 | **1096** | TLR6 | **2143** | CHIT1 | **3190** | AIF1 |
| 50 | STING1 | **1097** | CRB1 | **2144** | CASP4 | **3191** | CXCR3 |
| 51 | OLR1 | **1098** | MUC1 | **2145** | CXCL9 | **3192** | IFNA1 |
| 52 | MAPK14 | **1099** | TJP1 | **2146** | COL12A1 | **3193** | STX11 |
| 53 | CCL7 | **1100** | CRP | **2147** | PTK2B | **3194** | CBS |
| 54 | CPLANE1 | **1101** | CCL19 | **2148** | IKBKB | **3195** | IL12A |
| 55 | SOCS1 | **1102** | CD209 | **2149** | LIF | **3196** | PARD6B |
| 56 | SLC40A1 | **1103** | TLR8 | **2150** | CCR8 | **3197** | CD5L |
| 57 | CCL17 | **1104** | PLAUR | **2151** | MAPK8 | **3198** | IRF5 |
| 58 | PRKCD | **1105** | IKBKG | **2152** | CYBA | **3199** | BMP6 |
| 59 | CCL21 | **1106** | MAPK3 | **2153** | IFNB1 | **3200** | CNR2 |
| 60 | SPP1 | **1107** | IL4R | **2154** | RAC2 | **3201** | MIRLET7C |
| 61 | SMAD3 | **1108** | IL23A | **2155** | BTK | **3202** | KITLG |
| 62 | SLC11A2 | **1109** | PYCARD | **2156** | SHH | **3203** | NR1H2 |
| 63 | S100A9 | **1110** | GATA3 | **2157** | TREM1 | **3204** | MYC |
| 64 | SIRT1 | **1111** | IL12RB1 | **2158** | PRKCI | **3205** | HCK |
| 65 | ADIPOQ | **1112** | INPPL1 | **2159** | PATJ | **3206** | SRC |
| 66 | MIR125A | **1113** | MMP2 | **2160** | ITGAL | **3207** | C5AR1 |
| 67 | SMAD2 | **1114** | BRAF | **2161** | TYROBP | **3208** | NCF2 |
| 68 | SH2D1A | **1115** | TNFRSF11A | **2162** | GJA3 | **3209** | STAT5A |
| 69 | HSPA5 | **1116** | PTPN11 | **2163** | MIR7-3HG | **3210** | CRYBA1 |
| 70 | SCARNA5 | **1117** | GAS5 | **2164** | LGALS3 | **3211** | CTLA4 |
| 71 | IL18R1 | **1118** | CD80 | **2165** | SFTPD | **3212** | CTSD |
| 72 | IFNGR1 | **1119** | MSN | **2166** | SMPD1 | **3213** | NPC1 |
| 73 | FOS | **1120** | PLA2G7 | **2167** | IL15 | **3214** | DLG1 |
| 74 | CASP8 | **1121** | LYN | **2168** | LLGL1 | **3215** | CX3CL1 |
| 75 | CYP27A1 | **1122** | MIR146B | **2169** | LDLR | **3216** | S100A8 |
| 76 | PTPRC | **1123** | KDM6B | **2170** | CETP | **3217** | GBA1 |
| 77 | FPR2 | **1124** | SOD1 | **2171** | MIR155 | **3218** | PGR-AS1 |
| 78 | NOTCH1 | **1125** | SERPINE1 | **2172** | CD9 | **3219** | ADA |
| 79 | ARF6 | **1126** | ERCC3 | **2173** | GATA2 | **3220** | MMP14 |
| 80 | H19 | **1127** | TIMP1 | **2174** | FCGR3A | **3221** | FCGR1A |
| 81 | PIK3CA | **1128** | CRB2 | **2175** | CLEC10A | **3222** | MIR21 |
| 82 | SAMHD1 | **1129** | SFTPA1 | **2176** | ROCK1 | **3223** | ITK |
| 83 | PANX1 | **1130** | CLEC7A | **2177** | NCKAP1L | **3224** | RHOG |
| 84 | CEBPB | **1131** | ARG1 | **2178** | FN1 | **3225** | FABP4 |
| 85 | LACC1 | **1132** | PTPN6 | **2179** | CTSL | **3226** | CCR4 |
| 86 | CREB1 | **1133** | TF | **2180** | GRB2 | **3227** | CORO1A |
| 87 | ACTA2 | **1134** | MMP1 | **2181** | CDH1 | **3228** | RAF1 |
| 88 | AP3B1 | **1135** | FLT1 | **2182** | SFTPB | **3229** | NPC2 |
| 89 | BCL2L1 | **1136** | PPARA | **2183** | FCGR2A | **3230** | MCL1 |
| 90 | ELAVL1 | **1137** | MPO | **2184** | VANGL1 | **3231** | IL2RG |
| 91 | NR4A1 | **1138** | PIK3CD | **2185** | STAT2 | **3232** | PLA2G4A |
| 92 | ERBB2 | **1139** | CASP1 | **2186** | AHR | **3233** | LILRB1 |
| 93 | ESR1 | **1140** | EZH2 | **2187** | LGALS9 | **3234** | BAX |
| 94 | IL4I1 | **1141** | TBK1 | **2188** | EGR1 | **3235** | ZFP36 |
| 95 | ITGB1 | **1142** | IL32 | **2189** | TFRC | **3236** | PLA2G5 |
| 96 | INPP5D | **1143** | ERCC2 | **2190** | ACP5 | **3237** | TNFAIP3 |
| 97 | RAB8A | **1144** | CMKLR1 | **2191** | TFEB | **3238** | BSG |
| 98 | MAFB | **1145** | MFHAS1 | **2192** | ZC3H12A | **3239** | MPL |
| 99 | IGF1 | **1146** | IRF7 | **2193** | FLT3 | **3240** | SOAT1 |
| 100 | CTSK | **1147** | CXCR2 | **2194** | CXCL16 | **3241** | TRAF6 |
| 101 | IL1RL1 | **1148** | EPO | **2195** | TGFB2 | **3242** | NEU1 |
| 102 | TNFSF10 | **1149** | SLAMF1 | **2196** | ANPEP | **3243** | CD40LG |
| 103 | MAPK10 | **1150** | ALOX15 | **2197** | TPP1 | **3244** | GUSB |
| 104 | SFTPC | **1151** | CAV1 | **2198** | ELN | **3245** | CR1 |
| 105 | CLEC4E | **1152** | LILRB2 | **2199** | SQSTM1 | **3246** | FOLR2 |
| 106 | GSDMD | **1153** | CD70 | **2200** | ACE2 | **3247** | ADAM17 |
| 107 | PARP14 | **1154** | APP | **2201** | FMNL1 | **3248** | CARD9 |
| 108 | JAK1 | **1155** | ELANE | **2202** | APOB | **3249** | TIAM1 |
| 109 | GC | **1156** | HGF | **2203** | CCL1 | **3250** | EP300 |
| 110 | TARS1 | **1157** | ABCB1 | **2204** | IL11 | **3251** | GPNMB |
| 111 | ITGAX | **1158** | IRAK1 | **2205** | BST2 | **3252** | CTSB |
| 112 | VCAM1 | **1159** | CAT | **2206** | CLEC4D | **3253** | CASP9 |
| 113 | ABL1 | **1160** | DAB2 | **2207** | JAK3 | **3254** | ALOX5 |
| 114 | ETS1 | **1161** | BCL2 | **2208** | IL37 | **3255** | CHUK |
| 115 | PARP1 | **1162** | IRGM | **2209** | QKI | **3256** | F3 |
| 116 | CCL14 | **1163** | HSF4 | **2210** | SERPINA1 | **3257** | HDAC1 |
| 117 | PF4 | **1164** | FOXO1 | **2211** | SUCNR1 | **3258** | STK4 |
| 118 | STAB1 | **1165** | RHOD | **2212** | IRAK3 | **3259** | PCSK9 |
| 119 | FADD | **1166** | JMJD6 | **2213** | CXCL11 | **3260** | ANXA1 |
| 120 | DAAM1 | **1167** | IL6R | **2214** | HBEGF | **3261** | CASP5 |
| 121 | MAPT | **1168** | LEP | **2215** | FCGR2B | **3262** | PATL2 |
| 122 | HRAS | **1169** | MMP3 | **2216** | VAV1 | **3263** | CD63 |
| 123 | HSPD1 | **1170** | TRA-TGC7-1 | **2217** | OSM | **3264** | ALB |
| 124 | TLR5 | **1171** | TICAM1 | **2218** | DPP4 | **3265** | ANXA2 |
| 125 | GZMB | **1172** | IL1R1 | **2219** | PJA2 | **3266** | CXCL5 |
| 126 | CLEC5A | **1173** | GSN | **2220** | TNFSF13B | **3267** | RIGI |
| 127 | HDAC6 | **1174** | SHC1 | **2221** | C3 | **3268** | EZR |
| 128 | PLAU | **1175** | KRAS | **2222** | PTGS1 | **3269** | HLA-G |
| 129 | NCF4 | **1176** | PWAR4 | **2223** | IL7 | **3270** | GPBAR1 |
| 130 | CD200R1 | **1177** | CXCL13 | **2224** | SP1 | **3271** | CSF3R |
| 131 | ITPR1 | **1178** | AXL | **2225** | IGHE | **3272** | CARMIL2 |
| 132 | CCL8 | **1179** | TGFBR1 | **2226** | PON1 | **3273** | FPR1 |
| 133 | DEFB4A | **1180** | IFIH1 | **2227** | NRAS | **3274** | CD27 |
| 134 | AIM2 | **1181** | MRC2 | **2228** | VAV3 | **3275** | HFE |
| 135 | CD8A | **1182** | STAT5B | **2229** | PLIN2 | **3276** | METTL3 |
| 136 | CBL | **1183** | LCK | **2230** | SCARB2 | **3277** | MPP1 |
| 137 | ZAP70 | **1184** | NUMA1 | **2231** | NPHP1 | **3278** | HSPA4 |
| 138 | ABCC2 | **1185** | CPLANE2 | **2232** | LILRB4 | **3279** | RIPK1 |
| 139 | PTK7 | **1186** | THBS1 | **2233** | CTSA | **3280** | DOCK8 |
| 140 | PPBP | **1187** | IL3RA | **2234** | PRKAA1 | **3281** | FAM76B |
| 141 | ZEB1 | **1188** | CCL23 | **2235** | COL4A3 | **3282** | ABCG2 |
| 142 | ARHGEF2 | **1189** | MIR146A | **2236** | ETS2 | **3283** | PLA2G2A |
| 143 | ROCK2 | **1190** | TLR1 | **2237** | TRPV4 | **3284** | MYO18A |
| 144 | CXCR1 | **1191** | MAPK9 | **2238** | RARRES2 | **3285** | IL27 |
| 145 | CCL15 | **1192** | PRKD1 | **2239** | FOXO3 | **3286** | MYDGF |
| 146 | FGL2 | **1193** | SELL | **2240** | ACTG1 | **3287** | CXCL14 |
| 147 | CD1C | **1194** | ITIH4 | **2241** | GRN | **3288** | PRKCE |
| 148 | APC | **1195** | PPP1CA | **2242** | CCN2 | **3289** | BRD4 |
| 149 | PTPN2 | **1196** | LCN2 | **2243** | POSTN | **3290** | GLB1 |
| 150 | ACOD1 | **1197** | FRYL | **2244** | AGER | **3291** | IRF4 |
| 151 | ATP7B | **1198** | SI | **2245** | CIITA | **3292** | CD46 |
| 152 | CDK6 | **1199** | MIR98 | **2246** | CD69 | **3293** | PDGFRB |
| 153 | SLC2A1 | **1200** | CD1A | **2247** | DYSF | **3294** | KIT |
| 154 | CGAS | **1201** | LIN7A | **2248** | TBXAS1 | **3295** | CCL26 |
| 155 | RACK1 | **1202** | FGR | **2249** | PRTN3 | **3296** | CD247 |
| 156 | TSPO | **1203** | SREBF1 | **2250** | RAB11A | **3297** | MYO5B |
| 157 | VEGFC | **1204** | PSAP | **2251** | FCER2 | **3298** | ABCC1 |
| 158 | TNFAIP8L2 | **1205** | APOBEC3G | **2252** | LY96 | **3299** | CLEC12A |
| 159 | AMOT | **1206** | KLRK1 | **2253** | PTCH1 | **3300** | CD83 |
| 160 | MAP1LC3A | **1207** | NAGA | **2254** | RAP1B | **3301** | IKZF1 |
| 161 | RARA | **1208** | MPP7 | **2255** | ACE | **3302** | SLC22A5 |
| 162 | KRIT1 | **1209** | AKT2 | **2256** | IFNGR2 | **3303** | TGM2 |
| 163 | BMP4 | **1210** | MDM2 | **2257** | STUB1 | **3304** | PLCG2 |
| 164 | RAP1A | **1211** | RUNX2 | **2258** | TGFA | **3305** | CXCR6 |
| 165 | MMP7 | **1212** | IL9 | **2259** | CSK | **3306** | PXN |
| 166 | LTA | **1213** | PRKCB | **2260** | NAIP | **3307** | CXCL3 |
| 167 | CTSG | **1214** | ITGAV | **2261** | LTF | **3308** | TNFAIP6 |
| 168 | CD1D | **1215** | TIMP3 | **2262** | CYP27B1 | **3309** | OCLN |
| 169 | ERN1 | **1216** | FGFR1 | **2263** | TIRAP | **3310** | ARSA |
| 170 | NPM1 | **1217** | S100B | **2264** | MBL2 | **3311** | CD276 |
| 171 | PECAM1 | **1218** | COPA | **2265** | DMBT1 | **3312** | PDPK1 |
| 172 | CCNT1 | **1219** | HBB | **2266** | BMP2 | **3313** | SLC3A2 |
| 173 | SMURF1 | **1220** | LIN7B | **2267** | ACTR2 | **3314** | SMAD4 |
| 174 | AP3D1 | **1221** | TGFBR2 | **2268** | SNCA | **3315** | CD3E |
| 175 | DDIT3 | **1222** | HDAC2 | **2269** | GALC | **3316** | VSIG4 |
| 176 | HLA-DRA | **1223** | HLA-DPB1 | **2270** | NOD1 | **3317** | PLA2G6 |
| 177 | FES | **1224** | DCAF1 | **2271** | RB1 | **3318** | CD38 |
| 178 | NR3C1 | **1225** | MIR155HG | **2272** | CTSS | **3319** | NHERF1 |
| 179 | LGALS1 | **1226** | PTGER4 | **2273** | IGF2BP2 | **3320** | VPS33B |
| 180 | MAP3K8 | **1227** | IL6ST | **2274** | MPLKIP | **3321** | GJB2 |
| 181 | RASGRP1 | **1228** | SLC48A1 | **2275** | PTPN1 | **3322** | PKHD1 |
| 182 | CCL25 | **1229** | LAMP2 | **2276** | SNAI1 | **3323** | FLT3LG |
| 183 | BECN1 | **1230** | CYCS | **2277** | CD33 | **3324** | SLC29A3 |
| 184 | VTCN1 | **1231** | ARHGAP35 | **2278** | CDKN3 | **3325** | S100A4 |
| 185 | B2M | **1232** | MFGE8 | **2279** | ARG2 | **3326** | JAG1 |
| 186 | VDR | **1233** | TRIM32 | **2280** | NOX4 | **3327** | TSC22D3 |
| 187 | TGFB3 | **1234** | ACTR3 | **2281** | SLC9A1 | **3328** | CD84 |
| 188 | ANXA5 | **1235** | CREBBP | **2282** | ITGB3 | **3329** | ACTA1 |
| 189 | IKBKE | **1236** | TNFRSF1B | **2283** | NFATC1 | **3330** | LBP |
| 190 | SLPI | **1237** | HLA-DPA1 | **2284** | TNFRSF11B | **3331** | PARK7 |
| 191 | OAS1 | **1238** | MARCKS | **2285** | FMN2 | **3332** | HCFC1 |
| 192 | PNP | **1239** | PROM1 | **2286** | UCP2 | **3333** | COPS5 |
| 193 | FGF2 | **1240** | CD28 | **2287** | TRIB1 | **3334** | TNFSF9 |
| 194 | HSP90AA1 | **1241** | INS | **2288** | MBP | **3335** | HLA-DMA |
| 195 | PLCG1 | **1242** | CAMP | **2289** | WWTR1 | **3336** | ATG5 |
| 196 | TUBB3 | **1243** | HLA-DQA1 | **2290** | TUBB | **3337** | HLA-C |
| 197 | LYZ | **1244** | PIK3CB | **2291** | DNASE2 | **3338** | IL10RA |
| 198 | RIPK3 | **1245** | TRC-GCA24-1 | **2292** | PAK1 | **3339** | TXNIP |
| 199 | ALOX15B | **1246** | RPGRIP1L | **2293** | ADGRB1 | **3340** | KIF5B |
| 200 | CEBPD | **1247** | DOCK7 | **2294** | DMD | **3341** | BBS2 |
| 201 | IL23R | **1248** | MIRLET7B | **2295** | PLA2G10 | **3342** | C1QBP |
| 202 | IL16 | **1249** | ZEB2 | **2296** | TTC7A | **3343** | JAM3 |
| 203 | CALCA | **1250** | LRP1 | **2297** | ATF3 | **3344** | RORA |
| 204 | PRKDC | **1251** | CDH5 | **2298** | CEACAM5 | **3345** | RETN |
| 205 | CTSC | **1252** | MAPK7 | **2299** | KCTD7 | **3346** | LOX |
| 206 | CCND1 | **1253** | DPYSL2 | **2300** | SIGLEC9 | **3347** | F2RL1 |
| 207 | NR1H4 | **1254** | RAB13 | **2301** | SNORD15A | **3348** | ITGA4 |
| 208 | S100A12 | **1255** | PKM | **2302** | TYR | **3349** | ACTB |
| 209 | ENG | **1256** | SLC30A1 | **2303** | EGF | **3350** | CCL24 |
| 210 | PFN1 | **1257** | IL12RB2 | **2304** | MOS | **3351** | COL4A5 |
| 211 | LIPE | **1258** | NCF1 | **2305** | CCL13 | **3352** | LYVE1 |
| 212 | FYN | **1259** | MYH9 | **2306** | HRG | **3353** | LMNA |
| 213 | RAB7A | **1260** | COL1A1 | **2307** | ETV3 | **3354** | CD248 |
| 214 | CFH | **1261** | APOC1 | **2308** | PDGFB | **3355** | PDPN |
| 215 | PGF | **1262** | BGN | **2309** | ARSB | **3356** | HDAC9 |
| 216 | RNF114 | **1263** | ADORA2A | **2310** | TNC | **3357** | NPHP3 |
| 217 | IRS2 | **1264** | FLNA | **2311** | ATM | **3358** | MMP13 |
| 218 | STXBP3 | **1265** | MET | **2312** | CDK9 | **3359** | NCOR1 |
| 219 | GFI1 | **1266** | LGALS3BP | **2313** | IDS | **3360** | CD82 |
| 220 | TNFSF15 | **1267** | LCAT | **2314** | SFRP5 | **3361** | STAT4 |
| 221 | GREM1 | **1268** | MIR511 | **2315** | FCGR3B | **3362** | HLA-DMB |
| 222 | PRKCQ | **1269** | HBA1 | **2316** | RICTOR | **3363** | ATG16L1 |
| 223 | MALT1 | **1270** | RAB5A | **2317** | LSP1 | **3364** | GAB3 |
| 224 | SOCS2 | **1271** | RELB | **2318** | CTPS1 | **3365** | IGF2BP1 |
| 225 | ITGB4 | **1272** | IL7R | **2319** | TUG1 | **3366** | RHOC |
| 226 | NF2 | **1273** | NR1D1 | **2320** | GPR55 | **3367** | ALKBH5 |
| 227 | PEX5 | **1274** | HP | **2321** | IL1RAPL2 | **3368** | IL36G |
| 228 | CPM | **1275** | MYO5A | **2322** | TSLP | **3369** | MANF |
| 229 | SIRT6 | **1276** | EDN1 | **2323** | ADAM8 | **3370** | GNAS-AS1 |
| 230 | LRRK2 | **1277** | BIRC2 | **2324** | CEACAM3 | **3371** | BAD |
| 231 | CHRNA7 | **1278** | CALR | **2325** | PON2 | **3372** | TAOK1 |
| 232 | MIR34A | **1279** | TRE-TTC3-1 | **2326** | RXRA | **3373** | RUNX1 |
| 233 | LRP5 | **1280** | PMEL | **2327** | SELE | **3374** | CYLD |
| 234 | DLL4 | **1281** | BCAR1 | **2328** | ATP8B1 | **3375** | TNFSF13 |
| 235 | BIRC3 | **1282** | PLTP | **2329** | GTF2H5 | **3376** | CLEC4A |
| 236 | SND1 | **1283** | CST3 | **2330** | BDNF | **3377** | PTGER2 |
| 237 | MAP3K7 | **1284** | MIR494 | **2331** | RIPK2 | **3378** | TRAF3 |
| 238 | PPP1R9B | **1285** | SGCB | **2332** | NR4A1AS | **3379** | MIR24-1 |
| 239 | F11R | **1286** | AIRE | **2333** | CD3G | **3380** | YBX1 |
| 240 | ZNF335 | **1287** | PSEN1 | **2334** | TIMD4 | **3381** | KEAP1 |
| 241 | BRSK1 | **1288** | PPT1 | **2335** | HLA-DQB1 | **3382** | GLA |
| 242 | RTN4 | **1289** | CDKN2A | **2336** | STAR | **3383** | CEBPA |
| 243 | HBA2 | **1290** | WIPF1 | **2337** | PIEZO1 | **3384** | VWF |
| 244 | BCL2A1 | **1291** | VIP | **2338** | NTN1 | **3385** | MADD |
| 245 | TYMP | **1292** | MAP2K4 | **2339** | AKT3 | **3386** | NPHS1 |
| 246 | GAPDH | **1293** | AREG | **2340** | DNM1L | **3387** | DLG4 |
| 247 | KDR | **1294** | TET2 | **2341** | KCNN4 | **3388** | SIPA1L3 |
| 248 | SPARC | **1295** | HSPG2 | **2342** | ECT2 | **3389** | TAFA4 |
| 249 | CTSF | **1296** | NGF | **2343** | MARCKSL1 | **3390** | TXN |
| 250 | MID1 | **1297** | MX1 | **2344** | SEMA4A | **3391** | MUC5B |
| 251 | SENP3 | **1298** | SIGLEC7 | **2345** | FHL2 | **3392** | ACAT1 |
| 252 | RNASE3 | **1299** | IRAK4 | **2346** | MIR145 | **3393** | DLG5 |
| 253 | SLTM | **1300** | PLEKHO2 | **2347** | GPR32 | **3394** | TEAD1 |
| 254 | TIFAB | **1301** | MAP2K2 | **2348** | ADAM10 | **3395** | ATF4 |
| 255 | SLC16A4 | **1302** | STX3 | **2349** | MAP7 | **3396** | PTX3 |
| 256 | FASN | **1303** | AGFG1 | **2350** | PHGDH | **3397** | WASF2 |
| 257 | MCM4 | **1304** | LRBA | **2351** | CXCR5 | **3398** | MIR221 |
| 258 | LIPG | **1305** | BCL6 | **2352** | FSCN1 | **3399** | CBLB |
| 259 | MIR130A | **1306** | ARFGEF1 | **2353** | PTPN22 | **3400** | HPS6 |
| 260 | PAFAH1B1 | **1307** | H2AX | **2354** | GPR183 | **3401** | PTGES |
| 261 | BCR | **1308** | HSPA8 | **2355** | FHL1 | **3402** | HMGCR |
| 262 | PLCB1 | **1309** | MPDZ | **2356** | AARS1 | **3403** | IL31 |
| 263 | BIRC5 | **1310** | NINJ1 | **2357** | NAGLU | **3404** | PDCD1LG2 |
| 264 | KARS1 | **1311** | TNFSF12 | **2358** | CD244 | **3405** | GRK2 |
| 265 | DUSP1 | **1312** | USP7 | **2359** | CD79A | **3406** | TWIST1 |
| 266 | TUBA1B | **1313** | FTH1 | **2360** | ARL8B | **3407** | SPN |
| 267 | POMT1 | **1314** | IL19 | **2361** | TUBA1A | **3408** | TSC2 |
| 268 | MAP3K5 | **1315** | RDX | **2362** | OTOF | **3409** | HNF1A-AS1 |
| 269 | KLF6 | **1316** | PPARGC1A | **2363** | MAPRE1 | **3410** | CNR1 |
| 270 | NOS1 | **1317** | IDUA | **2364** | P2RY2 | **3411** | ARRB2 |
| 271 | PDE4A | **1318** | MMAB | **2365** | MALAT1 | **3412** | AZU1 |
| 272 | NBAS | **1319** | NEAT1 | **2366** | CD207 | **3413** | CRTAM |
| 273 | FTO | **1320** | SLC15A1 | **2367** | CEACAM8 | **3414** | MIR451A |
| 274 | PLP1 | **1321** | SIN3A | **2368** | MIR222 | **3415** | LATS1 |
| 275 | THBD | **1322** | LDHA | **2369** | FZD5 | **3416** | IL22 |
| 276 | SHPK | **1323** | CCL14 | **2370** | ALDH2 | **3417** | HSF1 |
| 277 | YY1 | **1324** | MTCL1 | **2371** | VSIR | **3418** | PTAFR |
| 278 | PADI4 | **1325** | YWHAZ | **2372** | IGF2 | **3419** | ADAM9 |
| 279 | WNT1 | **1326** | CLASP2 | **2373** | SDCCAG8 | **3420** | MIR223 |
| 280 | VCL | **1327** | HDAC8 | **2374** | TMEM67 | **3421** | AEBP1 |
| 281 | FZD4 | **1328** | SFRP1 | **2375** | NOX1 | **3422** | PRMT1 |
| 282 | SLC37A4 | **1329** | TAP1 | **2376** | MYH11 | **3423** | CD3D |
| 283 | HLA-A | **1330** | TSC1 | **2377** | RASSF5 | **3424** | ICAM2 |
| 284 | CYP1A1 | **1331** | NAMPT | **2378** | PLSCR1 | **3425** | ARL13B |
| 285 | NLRP1 | **1332** | WDR1 | **2379** | SLC2A3 | **3426** | NT5E |
| 286 | SMAD7 | **1333** | PRKCH | **2380** | CTHRC1 | **3427** | MMP8 |
| 287 | CLN3 | **1334** | CD200 | **2381** | TEK | **3428** | TERT |
| 288 | DKK1 | **1335** | GJA1 | **2382** | AURKA | **3429** | SEMA7A |
| 289 | STK26 | **1336** | SLC7A11 | **2383** | EEA1 | **3430** | LAMC2 |
| 290 | LRP2 | **1337** | ABCD1 | **2384** | ANGPTL4 | **3431** | PRKAA2 |
| 291 | TMEM114 | **1338** | SKI | **2385** | RBPJ | **3432** | HSD17B4 |
| 292 | DEFB1 | **1339** | IDH2 | **2386** | MAP2K6 | **3433** | NDUFS4 |
| 293 | RAPGEF3 | **1340** | AP1B1 | **2387** | PTPRO | **3434** | DIABLO |
| 294 | ITGA5 | **1341** | NCOR2 | **2388** | TYK2 | **3435** | DDB1 |
| 295 | MIR99B | **1342** | HNRNPA2B1 | **2389** | CDK4 | **3436** | ADAR |
| 296 | CD1B | **1343** | SOD2 | **2390** | HLA-DQB2 | **3437** | TCF4 |
| 297 | GALNS | **1344** | TNFRSF6B | **2391** | TOLLIP | **3438** | ANGPT1 |
| 298 | KCNQ1 | **1345** | ARHGAP24 | **2392** | SCO2 | **3439** | IL2RB |
| 299 | RPN2 | **1346** | ILF3 | **2393** | LITAF | **3440** | G6PD |
| 300 | COL4A4 | **1347** | MCCC2 | **2394** | TAP2 | **3441** | PNPLA2 |
| 301 | CLDN7 | **1348** | TRIP10 | **2395** | CTNNA1 | **3442** | UGCG |
| 302 | VCAN | **1349** | NCL | **2396** | IL31RA | **3443** | HSPB8 |
| 303 | IDH1 | **1350** | XDH | **2397** | CYP3A4 | **3444** | MIR320A |
| 304 | PTPRJ | **1351** | FGF4 | **2398** | FBF1 | **3445** | STX4 |
| 305 | DCN | **1352** | EIF2AK2 | **2399** | SIGLEC15 | **3446** | ZBTB20 |
| 306 | BRCA1 | **1353** | CRKL | **2400** | CYP1B1 | **3447** | SNAP23 |
| 307 | MLKL | **1354** | GLI1 | **2401** | TTR | **3448** | MCAM |
| 308 | DDB2 | **1355** | CPVL | **2402** | MPI | **3449** | EIF4A1 |
| 309 | GNAI3 | **1356** | RAB11FIP2 | **2403** | MLLT3 | **3450** | IQGAP1 |
| 310 | TNFSF4 | **1357** | AHI1 | **2404** | NCAM1 | **3451** | DHCR7 |
| 311 | KDM1A | **1358** | PLCB3 | **2405** | MAT2A | **3452** | ARNT |
| 312 | OXA1L | **1359** | DICER1 | **2406** | BCL2L11 | **3453** | HSPA1A |
| 313 | WEE1 | **1360** | WNT3A | **2407** | MAP2K3 | **3454** | FCGRT |
| 314 | MTDH | **1361** | TGOLN2 | **2408** | SBNO2 | **3455** | FOSL2 |
| 315 | ADORA2B | **1362** | SELP | **2409** | SDC1 | **3456** | IFNA2 |
| 316 | TIGIT | **1363** | DEFB103A | **2410** | SLC29A1 | **3457** | MLC1 |
| 317 | TUBA1C | **1364** | AP1G1 | **2411** | GCA | **3458** | PVT1 |
| 318 | IL10RB | **1365** | MAPKAPK2 | **2412** | ALOX5AP | **3459** | PHB1 |
| 319 | MYH14 | **1366** | WNT7A | **2413** | IGF2BP3 | **3460** | WNT7B |
| 320 | SREBF2 | **1367** | SPTBN1 | **2414** | HNRNPD | **3461** | ISG15 |
| 321 | PRDX1 | **1368** | MKI67 | **2415** | SLC4A7 | **3462** | EGR2 |
| 322 | COL3A1 | **1369** | ACP1 | **2416** | IL21 | **3463** | CLN8 |
| 323 | SMARCA4 | **1370** | GSK3A | **2417** | ADGRE5 | **3464** | ADIPOR2 |
| 324 | FGF7 | **1371** | UMOD | **2418** | EIF2AK4 | **3465** | PRKAB1 |
| 325 | MIR27A | **1372** | YWHAE | **2419** | DBT | **3466** | RPS3 |
| 326 | TNFRSF9 | **1373** | METTL14 | **2420** | DNMT3A | **3467** | TRIM62 |
| 327 | FOXM1 | **1374** | TRIM21 | **2421** | YY1AP1 | **3468** | FANCI |
| 328 | HDAC5 | **1375** | CAPNS1 | **2422** | FAT4 | **3469** | PAK4 |
| 329 | GLP1R | **1376** | FYCO1 | **2423** | ITGAD | **3470** | SLC25A13 |
| 330 | DNASE1L3 | **1377** | DCSTAMP | **2424** | L1CAM | **3471** | MDK |
| 331 | TIMP2 | **1378** | FURIN | **2425** | CDKN1A | **3472** | HDAC3 |
| 332 | DHX9 | **1379** | MEF2D | **2426** | RAB8B | **3473** | SERPINB2 |
| 333 | ADRB2 | **1380** | NFAT5 | **2427** | NOTCH3 | **3474** | KMT2A |
| 334 | PCCA | **1381** | PDIA3 | **2428** | VTN | **3475** | RPS6KA1 |
| 335 | TAFA3 | **1382** | IKZF2 | **2429** | INPP5E | **3476** | HSPB1 |
| 336 | ELP1 | **1383** | FAT1 | **2430** | ADAMDEC1 | **3477** | PPIF |
| 337 | FLNB | **1384** | AR | **2431** | HADHA | **3478** | ADIPOR1 |
| 338 | KAT2B | **1385** | TRIM65 | **2432** | BCL3 | **3479** | AGTR1 |
| 339 | RAB10 | **1386** | CACNA1C | **2433** | RAB5C | **3480** | SLC26A4 |
| 340 | GLI3 | **1387** | ABCG5 | **2434** | PLOD1 | **3481** | CFLAR |
| 341 | M6PR | **1388** | SELPLG | **2435** | COL7A1 | **3482** | PRKN |
| 342 | SH3BP2 | **1389** | CYTH1 | **2436** | DOCK2 | **3483** | EPHB2 |
| 343 | GLUL | **1390** | COL5A1 | **2437** | LCP1 | **3484** | PADI2 |
| 344 | CASP7 | **1391** | HSP90B1 | **2438** | LGMN | **3485** | STAP1 |
| 345 | SERPINF1 | **1392** | PML | **2439** | ADCY10 | **3486** | HLA-B |
| 346 | SLC4A1 | **1393** | ILK | **2440** | ST14 | **3487** | CDH2 |
| 347 | CD22 | **1394** | ADGRE2 | **2441** | ZBP1 | **3488** | TAT |
| 348 | HTRA2 | **1395** | TNIP1 | **2442** | HDAC11 | **3489** | HHLA2 |
| 349 | WNT4 | **1396** | MMACHC | **2443** | BLM | **3490** | IRF9 |
| 350 | VASP | **1397** | NLRP7 | **2444** | CPT1A | **3491** | LZTR1 |
| 351 | GPC3 | **1398** | PIGR | **2445** | KPNA2 | **3492** | IL17F |
| 352 | MFN2 | **1399** | CD163L1 | **2446** | DDR1 | **3493** | SPIRE1 |
| 353 | NEDD4L | **1400** | NUP85 | **2447** | LOXL2 | **3494** | MT-TP |
| 354 | TNPO1 | **1401** | ODC1 | **2448** | ADA2 | **3495** | NR4A3 |
| 355 | F9 | **1402** | PTBP1 | **2449** | MLANA | **3496** | EXOC4 |
| 356 | KPNA3 | **1403** | XCL1 | **2450** | DNMT1 | **3497** | HDLBP |
| 357 | COX5A | **1404** | ARPC1B | **2451** | PCCB | **3498** | PRKACA |
| 358 | MYO6 | **1405** | MIR379 | **2452** | THOC5 | **3499** | MIR214 |
| 359 | HSPA12A | **1406** | SPINT1 | **2453** | RGS12 | **3500** | TP63 |
| 360 | FBN1 | **1407** | TMEM106A | **2454** | LTB4R | **3501** | BRIP1 |
| 361 | GBP2 | **1408** | PLCB4 | **2455** | SLC26A2 | **3502** | LDAH |
| 362 | MRTFA | **1409** | NEB | **2456** | MIR619 | **3503** | MIR3135B |
| 363 | TKT | **1410** | ACADVL | **2457** | NLRP12 | **3504** | ADCYAP1 |
| 364 | CIRBP | **1411** | EFEMP2 | **2458** | NOTCH2 | **3505** | SNX27 |
| 365 | CPEB4 | **1412** | PANK4 | **2459** | TRAF2 | **3506** | MYLK |
| 366 | PON3 | **1413** | PRNP | **2460** | MME | **3507** | TNFRSF8 |
| 367 | SLC7A1 | **1414** | SLC5A1 | **2461** | TUBG1 | **3508** | YWHAQ |
| 368 | SLC39A14 | **1415** | IL18RAP | **2462** | F13A1 | **3509** | AQP4 |
| 369 | MKS1 | **1416** | F2 | **2463** | CD48 | **3510** | IFNAR1 |
| 370 | SLC38A3 | **1417** | FZD2 | **2464** | EBAG9 | **3511** | RPS6KB1 |
| 371 | SAA1 | **1418** | CRK | **2465** | NEDD9 | **3512** | TNFRSF18 |
| 372 | DAG1 | **1419** | ITLN1 | **2466** | PLK3 | **3513** | MTTP |
| 373 | ITGA6 | **1420** | FGFR2 | **2467** | ISG20 | **3514** | ASL |
| 374 | TMSB4X | **1421** | ENO1 | **2468** | CCDC88C | **3515** | HNRNPA1 |
| 375 | TOR1A | **1422** | HPSE | **2469** | LGALS8 | **3516** | ADAMTSL1 |
| 376 | ICOS | **1423** | C5AR2 | **2470** | TXNRD1 | **3517** | MUC4 |
| 377 | MAN2B1 | **1424** | CAMK2A | **2471** | EXOC3 | **3518** | CEP290 |
| 378 | PPP2CA | **1425** | WNT11 | **2472** | CTCF | **3519** | HCG18 |
| 379 | BMPR2 | **1426** | PIK3R2 | **2473** | PEBP1 | **3520** | ELMO1 |
| 380 | GNAI1 | **1427** | CALCR | **2474** | PDHX | **3521** | PODXL |
| 381 | CUL4A | **1428** | MIR210 | **2475** | EIF4E | **3522** | RUFY3 |
| 382 | SCN5A | **1429** | LIG4 | **2476** | TRPA1 | **3523** | DEFB103B |
| 383 | CSTB | **1430** | NPHS2 | **2477** | ABCC8 | **3524** | ABR |
| 384 | AMOTL1 | **1431** | NR4A2 | **2478** | CLEC6A | **3525** | DLK1 |
| 385 | CD34 | **1432** | TPI1 | **2479** | TRIM22 | **3526** | OPTN |
| 386 | NUDC | **1433** | PFKFB3 | **2480** | SIGIRR | **3527** | LRPPRC |
| 387 | TRPV1 | **1434** | HLA-DQA2 | **2481** | TRP-AGG2-5 | **3528** | ADAMTS4 |
| 388 | PTGER3 | **1435** | SLC46A2 | **2482** | KIF26B | **3529** | GIT1 |
| 389 | PPIA | **1436** | STK24 | **2483** | DYNC2H1 | **3530** | TACR1 |
| 390 | CD2 | **1437** | CYP2J2 | **2484** | RGCC | **3531** | GZMA |
| 391 | SNHG1 | **1438** | SLC17A5 | **2485** | ASPH | **3532** | ERVW-1 |
| 392 | TP53BP2 | **1439** | FERMT3 | **2486** | CYP21A2 | **3533** | SLC22A4 |
| 393 | PHB2 | **1440** | CKAP5 | **2487** | SPON2 | **3534** | CLDN1 |
| 394 | RAB20 | **1441** | FLII | **2488** | ASS1 | **3535** | HLA-DRB3 |
| 395 | FCER1G | **1442** | P2RX4 | **2489** | SETD2 | **3536** | NEDD8 |
| 396 | CDH23 | **1443** | RELN | **2490** | WNT2 | **3537** | HMGA2 |
| 397 | NCOA4 | **1444** | YWHAG | **2491** | ITM2B | **3538** | MUC2 |
| 398 | SOAT2 | **1445** | PROS1 | **2492** | GBP1 | **3539** | ARHGAP31 |
| 399 | CYP19A1 | **1446** | GATA1 | **2493** | IL17RA | **3540** | MIR199B |
| 400 | PSTPIP1 | **1447** | TYRO3 | **2494** | LIPC | **3541** | USF1 |
| 401 | TGIF1 | **1448** | CYP24A1 | **2495** | IL26 | **3542** | MCPH1 |
| 402 | DIAPH1 | **1449** | TFR2 | **2496** | REL | **3543** | SOX2 |
| 403 | ZFP36L1 | **1450** | AMOTL2 | **2497** | FMR1 | **3544** | SEPTIN2 |
| 404 | VIM-AS1 | **1451** | RORC | **2498** | HOXB8 | **3545** | CD24 |
| 405 | CTNND1 | **1452** | MARK3 | **2499** | MAP1LC3B | **3546** | EPHA3 |
| 406 | PLEC | **1453** | CDX2 | **2500** | TUBB2A | **3547** | DCTN2 |
| 407 | SIRT3 | **1454** | GSR | **2501** | S1PR2 | **3548** | IGF1R |
| 408 | ANK3 | **1455** | ACKR3 | **2502** | SDCBP | **3549** | ALDH3A2 |
| 409 | ARHGEF11 | **1456** | BAK1 | **2503** | MIR1207 | **3550** | CHIA |
| 410 | CEL | **1457** | NUMB | **2504** | CA9 | **3551** | NTRK1 |
| 411 | POMT2 | **1458** | XPC | **2505** | UBC | **3552** | RRAS |
| 412 | EBP | **1459** | XCR1 | **2506** | CD58 | **3553** | GAA |
| 413 | EEF1A1 | **1460** | YTHDF2 | **2507** | HMGCL | **3554** | HMGA1 |
| 414 | KIF3A | **1461** | TUBA3C | **2508** | EPOR | **3555** | NDUFA13 |
| 415 | F10 | **1462** | CALM1 | **2509** | RHO | **3556** | SLC16A1 |
| 416 | PDHB | **1463** | AQP1 | **2510** | RS1 | **3557** | POLR2A |
| 417 | CXCL6 | **1464** | NIFK-AS1 | **2511** | SENP1 | **3558** | PEX6 |
| 418 | APOC2 | **1465** | AP1M1 | **2512** | P2RY12 | **3559** | PPP1CB |
| 419 | MEF2A | **1466** | FZD7 | **2513** | VAV2 | **3560** | ERBB3 |
| 420 | IRF2BP2 | **1467** | CD19 | **2514** | NOTCH4 | **3561** | MYH10 |
| 421 | TET1 | **1468** | TUBA4A | **2515** | OSBPL8 | **3562** | BMAL1 |
| 422 | DDX20 | **1469** | TTC8 | **2516** | ABCA2 | **3563** | HSD11B1 |
| 423 | DNAH8 | **1470** | KCNH2 | **2517** | BBS1 | **3564** | CCR5AS |
| 424 | IFNA21 | **1471** | SNHG16 | **2518** | MAVS | **3565** | ABL2 |
| 425 | ULK1 | **1472** | PKD1 | **2519** | ADGRE3 | **3566** | FNDC5 |
| 426 | GNAS | **1473** | SDC2 | **2520** | SH2B3 | **3567** | HS2ST1 |
| 427 | PRDM1 | **1474** | ARL4C | **2521** | CUX1 | **3568** | CSPG4 |
| 428 | SMN1 | **1475** | PPARGC1B | **2522** | CSNK2A1 | **3569** | PLEKHO1 |
| 429 | PLK1 | **1476** | F2R | **2523** | TRIM25 | **3570** | IL36RN |
| 430 | DST | **1477** | PRDX2 | **2524** | MAPKAP1 | **3571** | SLAMF7 |
| 431 | KLK2 | **1478** | PRICKLE4 | **2525** | UHRF1 | **3572** | LCP2 |
| 432 | EIF2AK3 | **1479** | XRCC6 | **2526** | MKKS | **3573** | B9D2 |
| 433 | HSP90AB1 | **1480** | MYO1D | **2527** | TP73 | **3574** | MIR373 |
| 434 | SLC5A6 | **1481** | SRSF2 | **2528** | TJP2 | **3575** | LOC112533672 |
| 435 | FTL | **1482** | CDK2 | **2529** | SH2D1B | **3576** | BGLAP |
| 436 | NOS3 | **1483** | ARRB1 | **2530** | ADM | **3577** | MCCC1 |
| 437 | MIR33B | **1484** | AMH | **2531** | RALA | **3578** | TPX2 |
| 438 | ST6GAL1 | **1485** | MIR29A | **2532** | LAMA3 | **3579** | BID |
| 439 | SEC24B | **1486** | SRGN | **2533** | MGLL | **3580** | ALMS1 |
| 440 | TLN1 | **1487** | EREG | **2534** | DAPK1 | **3581** | HVCN1 |
| 441 | RASA1 | **1488** | SCN8A | **2535** | GAS6 | **3582** | MFSD6 |
| 442 | CTTN | **1489** | ESR2 | **2536** | BMP7 | **3583** | SLC23A2 |
| 443 | SDC4 | **1490** | TCF7L2 | **2537** | TAB2 | **3584** | SLC1A3 |
| 444 | PKN2 | **1491** | KIF14 | **2538** | MAP3K1 | **3585** | UNC13B |
| 445 | LMNB1 | **1492** | JUNB | **2539** | NCR1 | **3586** | ABI1 |
| 446 | HLA-DOB | **1493** | RPS7 | **2540** | HDAC4 | **3587** | AARS2 |
| 447 | STIM1 | **1494** | SLC9A3 | **2541** | WT1 | **3588** | BPI |
| 448 | WASF1 | **1495** | KIF2C | **2542** | ARAF | **3589** | ATP7A |
| 449 | AGA | **1496** | RO60 | **2543** | IFI16 | **3590** | FBXW7 |
| 450 | MIR382 | **1497** | PSIP1 | **2544** | LBR | **3591** | ATP6V1B2 |
| 451 | POR | **1498** | NR2C2 | **2545** | MYB | **3592** | FOXE3 |
| 452 | KCNMA1 | **1499** | CLEC4M | **2546** | AGT | **3593** | CD151 |
| 453 | MIR142 | **1500** | SART1 | **2547** | DNM2 | **3594** | MAP2K7 |
| 454 | KRT18 | **1501** | PIK3C3 | **2548** | PGK1 | **3595** | GLS |
| 455 | SCD | **1502** | IGFBP2 | **2549** | OSBPL3 | **3596** | GPR132 |
| 456 | NGFR | **1503** | ALPL | **2550** | HEXIM1 | **3597** | CWC27 |
| 457 | ADORA3 | **1504** | AIFM1 | **2551** | TAB1 | **3598** | DACT1 |
| 458 | PRL | **1505** | MIR33A | **2552** | LAMP3 | **3599** | PLCB2 |
| 459 | TAX1BP1 | **1506** | IGF2R | **2553** | EPSTI1 | **3600** | COL5A2 |
| 460 | MED12 | **1507** | INHBA | **2554** | G3BP1 | **3601** | PEX19 |
| 461 | GTF2H2 | **1508** | SYT7 | **2555** | BBS9 | **3602** | HEXA |
| 462 | HAVCR1 | **1509** | ABCC4 | **2556** | PLPP3 | **3603** | ANK2 |
| 463 | ATP8A1 | **1510** | TAPBP | **2557** | CD55 | **3604** | GRIP1 |
| 464 | NFKB2 | **1511** | KLRD1 | **2558** | HDAC7 | **3605** | SACS |
| 465 | HLA-E | **1512** | TERF2IP | **2559** | HLA-DRB5 | **3606** | SEMA3A |
| 466 | FSTL1 | **1513** | MMP28 | **2560** | USP14 | **3607** | NBN |
| 467 | GPR84 | **1514** | FIRRE | **2561** | HYOU1 | **3608** | GFAP |
| 468 | FKRP | **1515** | PTPN3 | **2562** | PSMC6 | **3609** | MTHFD1 |
| 469 | SESN2 | **1516** | SKAP2 | **2563** | ACTN1 | **3610** | PLIN1 |
| 470 | NDUFS7 | **1517** | TFAP2A | **2564** | SLC2A5 | **3611** | SLC15A2 |
| 471 | TLR10 | **1518** | GPT | **2565** | TMEM119 | **3612** | TRPM2 |
| 472 | NPPA | **1519** | USP9X | **2566** | MIR122 | **3613** | PLG |
| 473 | HEXB | **1520** | SIX1 | **2567** | ZDHHC7 | **3614** | MIR30A |
| 474 | RNF213 | **1521** | CBX3 | **2568** | FMN1 | **3615** | PABPC1 |
| 475 | S1PR1 | **1522** | EGLN1 | **2569** | LRP6 | **3616** | SYMPK |
| 476 | ZFP36L2 | **1523** | MECP2 | **2570** | SOCS6 | **3617** | EIF4EBP1 |
| 477 | UCHL1 | **1524** | EEF1D | **2571** | KIR3DL1 | **3618** | EWSR1 |
| 478 | HNRNPDL | **1525** | PMM2 | **2572** | NRIP1 | **3619** | TUBA3D |
| 479 | KLF2 | **1526** | FUS | **2573** | FPR3 | **3620** | POU1F1 |
| 480 | ERBIN | **1527** | NQO1 | **2574** | ITGA3 | **3621** | PDHA1 |
| 481 | CAPN2 | **1528** | RMC1 | **2575** | PIP5K1C | **3622** | STRIP1 |
| 482 | PRPF8 | **1529** | NDUFS6 | **2576** | ACADM | **3623** | AP2B1 |
| 483 | FMNL2 | **1530** | CSNK2B | **2577** | GNLY | **3624** | MIR216A |
| 484 | AGPS | **1531** | DNASE1L1 | **2578** | LMNB2 | **3625** | NTRK2 |
| 485 | MIR34C | **1532** | MEF2C | **2579** | NEDD4 | **3626** | RP2 |
| 486 | AHSG | **1533** | POLG | **2580** | IFI30 | **3627** | YWHAB |
| 487 | NES | **1534** | AGO2 | **2581** | ICOSLG | **3628** | BBS12 |
| 488 | ORAI1 | **1535** | SERPINE2 | **2582** | APBA3 | **3629** | KPNB1 |
| 489 | PTPA | **1536** | ENTPD1 | **2583** | LAIR1 | **3630** | PDLIM2 |
| 490 | OTC | **1537** | FFAR4 | **2584** | NRG1 | **3631** | DCLRE1C |
| 491 | SF1 | **1538** | EN1 | **2585** | PIM1 | **3632** | STAB2 |
| 492 | FANCE | **1539** | MGAT5 | **2586** | SLC4A2 | **3633** | ORM1 |
| 493 | SEMA4D | **1540** | VAMP7 | **2587** | PDE2A | **3634** | P2RY1 |
| 494 | CLMP | **1541** | CP | **2588** | CALM2 | **3635** | EPB41L5 |
| 495 | IL17D | **1542** | USP18 | **2589** | PLXNB2 | **3636** | HIF1AN |
| 496 | PDP1 | **1543** | ALOX12 | **2590** | ARHGEF1 | **3637** | DOT1L |
| 497 | TNFAIP2 | **1544** | CTDSP2 | **2591** | CASK | **3638** | F8 |
| 498 | BEST1 | **1545** | PRRC2B | **2592** | RAB14 | **3639** | RHOB |
| 499 | SNX10 | **1546** | RAC3 | **2593** | KRT5 | **3640** | MITF |
| 500 | KAT5 | **1547** | STIL | **2594** | SLC9D1 | **3641** | MIR125B1 |
| 501 | MYO1C | **1548** | OTUD5 | **2595** | PGAM5 | **3642** | EPCAM |
| 502 | MIR126 | **1549** | CDK1 | **2596** | GLRX | **3643** | CELSR1 |
| 503 | TMOD3 | **1550** | ATG7 | **2597** | ACSL1 | **3644** | APPL1 |
| 504 | C5 | **1551** | MACF1 | **2598** | FLOT1 | **3645** | ALK |
| 505 | SPHK1 | **1552** | DSPP | **2599** | CTSH | **3646** | AZGP1 |
| 506 | PDGFA | **1553** | LAG3 | **2600** | NRP1 | **3647** | E2F1 |
| 507 | MYL6 | **1554** | ATF2 | **2601** | KPNA1 | **3648** | ANGPT2 |
| 508 | BACE1 | **1555** | AP2A1 | **2602** | WNT2B | **3649** | CD2AP |
| 509 | ACLY | **1556** | ATP1A1 | **2603** | SFPQ | **3650** | CEACAM6 |
| 510 | MRAS | **1557** | WASL | **2604** | SPRY2 | **3651** | OCSTAMP |
| 511 | PPM1A | **1558** | SOS1 | **2605** | SERPINB1 | **3652** | GLDC |
| 512 | JUND | **1559** | PDCD6IP | **2606** | BCKDHA | **3653** | TG |
| 513 | MGMT | **1560** | ACKR2 | **2607** | TOMM20 | **3654** | SLC1A2 |
| 514 | MYH2 | **1561** | HTT | **2608** | LPA | **3655** | SLC2A4 |
| 515 | TAC1 | **1562** | FLOT2 | **2609** | HSP90AA2P | **3656** | MAP1B |
| 516 | CAPN1 | **1563** | LINC02620 | **2610** | PLAT | **3657** | PLEK |
| 517 | RECK | **1564** | IFN1@ | **2611** | MIAT | **3658** | PEX1 |
| 518 | AKT1S1 | **1565** | DAXX | **2612** | PTGDS | **3659** | S100A11 |
| 519 | ANK1 | **1566** | SPTLC1 | **2613** | EXOC2 | **3660** | LAPTM5 |
| 520 | JMJD1C | **1567** | KIF4A | **2614** | HNRNPM | **3661** | ACTN4 |
| 521 | TRPM7 | **1568** | CLU | **2615** | HNRNPU | **3662** | SAV1 |
| 522 | ARID1A | **1569** | CLTC | **2616** | HSPA6 | **3663** | CALM3 |
| 523 | ADPRH | **1570** | LPCAT3 | **2617** | DUSP3 | **3664** | PGM3 |
| 524 | SNORD44 | **1571** | LAMB3 | **2618** | DSG2 | **3665** | LAMA1 |
| 525 | SLC19A3 | **1572** | NRP2 | **2619** | RUVBL1 | **3666** | CHGA |
| 526 | RPS6 | **1573** | RNASET2 | **2620** | RPLP2 | **3667** | NFATC2 |
| 527 | SLC22A2 | **1574** | SYNE4 | **2621** | RPS6KA5 | **3668** | SOD3 |
| 528 | EIF5A | **1575** | GFER | **2622** | NCK1 | **3669** | MIR9-2HG |
| 529 | HPRT1 | **1576** | ILF2 | **2623** | P4HB | **3670** | UBE2K |
| 530 | G6PC1 | **1577** | TEAD4 | **2624** | USH1G | **3671** | AURKB |
| 531 | TNFRSF12A | **1578** | IFT140 | **2625** | EDNRB | **3672** | SF3B2 |
| 532 | TNFSF18 | **1579** | DRD2 | **2626** | ELF4 | **3673** | TNFRSF14 |
| 533 | GPX1 | **1580** | IHH | **2627** | HDC | **3674** | IVD |
| 534 | ALCAM | **1581** | DYNLL1 | **2628** | XPA | **3675** | NEK7 |
| 535 | IL18BP | **1582** | FANCD2 | **2629** | SIGLEC10 | **3676** | ID3 |
| 536 | TRP-AGG2-6 | **1583** | WHRN | **2630** | FUCA1 | **3677** | DGAT1 |
| 537 | ADAM12 | **1584** | FOSL1 | **2631** | MS4A4A | **3678** | SPINT2 |
| 538 | MFN1 | **1585** | MIR124-1 | **2632** | GATA6 | **3679** | MYO10 |
| 539 | MT-CYB | **1586** | F2RL2 | **2633** | VAPA | **3680** | C4A |
| 540 | PIK3C2A | **1587** | SLC35A1 | **2634** | WWC1 | **3681** | SLC46A1 |
| 541 | PAEP | **1588** | EVC2 | **2635** | FNBP1 | **3682** | CD1E |
| 542 | FGFR3 | **1589** | UNC5B | **2636** | STMN1 | **3683** | ADM2 |
| 543 | SRSF3 | **1590** | IRF2 | **2637** | NONO | **3684** | COL8A1 |
| 544 | HCST | **1591** | P2RY11 | **2638** | APOBEC3F | **3685** | TMEM237 |
| 545 | DLD | **1592** | PAPPA-AS1 | **2639** | RPL11 | **3686** | MDM4 |
| 546 | EDN2 | **1593** | GNB1 | **2640** | P2RX5-TAX1BP3 | **3687** | PLIN3 |
| 547 | NR0B1 | **1594** | LIPF | **2641** | CGA | **3688** | C3AR1 |
| 548 | RLBP1 | **1595** | ARID3A | **2642** | PVR | **3689** | FLT4 |
| 549 | VPS26A | **1596** | ZC3HAV1 | **2643** | MBNL1 | **3690** | OFD1 |
| 550 | USP12 | **1597** | KCNA3 | **2644** | GNAI2 | **3691** | SMPD3 |
| 551 | CAMK2G | **1598** | ATP2A2 | **2645** | MDH2 | **3692** | N4BP1 |
| 552 | RBP4 | **1599** | IL13RA1 | **2646** | COL4A1 | **3693** | FLI1 |
| 553 | RPL29 | **1600** | SGK1 | **2647** | RANBP2 | **3694** | CYSLTR2 |
| 554 | NNMT | **1601** | TNFRSF13B | **2648** | DAAM2 | **3695** | ARHGEF7 |
| 555 | PEX11B | **1602** | SRF | **2649** | PARVA | **3696** | MSH6 |
| 556 | WDR5 | **1603** | DAGLB | **2650** | REG3A | **3697** | SST |
| 557 | FMNL3 | **1604** | ELK4 | **2651** | RAG1 | **3698** | C1S |
| 558 | CCL16 | **1605** | CLIC1 | **2652** | KPNA4 | **3699** | KRT17 |
| 559 | FABP7 | **1606** | RTP3 | **2653** | SLC7A5 | **3700** | GRK3 |
| 560 | CPS1 | **1607** | RPS27A | **2654** | RFX5 | **3701** | PRC1 |
| 561 | CRTC3 | **1608** | KLRB1 | **2655** | FREM2 | **3702** | CCDC85C |
| 562 | FYB1 | **1609** | SEPTIN9 | **2656** | GCLM | **3703** | CDK5 |
| 563 | PLEKHG5 | **1610** | PTPN13 | **2657** | AP1M2 | **3704** | KIAA0586 |
| 564 | GNB2 | **1611** | DCLK1 | **2658** | TRIM24 | **3705** | ADCY1 |
| 565 | TSPAN32 | **1612** | ATXN2 | **2659** | C1QA | **3706** | CEBPE |
| 566 | RPL18A | **1613** | S100A7 | **2660** | LUM | **3707** | POLD1 |
| 567 | FERMT1 | **1614** | MUC5AC | **2661** | AQP3 | **3708** | VDAC1 |
| 568 | SERPINC1 | **1615** | CEP131 | **2662** | CD300LF | **3709** | SLC6A8 |
| 569 | ANGPTL2 | **1616** | MIR193A | **2663** | RET | **3710** | DUSP6 |
| 570 | DLG2 | **1617** | DNM3 | **2664** | NR1I2 | **3711** | E2F4 |
| 571 | ARHGDIA | **1618** | FIP1L1 | **2665** | GALK1 | **3712** | TUBB6 |
| 572 | ERC1 | **1619** | JUP | **2666** | RPS3A | **3713** | USO1 |
| 573 | PPM1F | **1620** | RAB35 | **2667** | CMTM6 | **3714** | SULT2B1 |
| 574 | FOLR1 | **1621** | VIPR1 | **2668** | TGFBI | **3715** | GMNN |
| 575 | PITRM1 | **1622** | FLG | **2669** | UBA1 | **3716** | ST3GAL4 |
| 576 | FANCL | **1623** | SLC12A6 | **2670** | MYO1F | **3717** | CHD4 |
| 577 | TBC1D1 | **1624** | POLH | **2671** | UBA52 | **3718** | TNFRSF10A |
| 578 | RPL18 | **1625** | CEP55 | **2672** | DUOX2 | **3719** | MOB1A |
| 579 | ASGR1 | **1626** | CFL1 | **2673** | FOLH1 | **3720** | CASP2 |
| 580 | MIR486-1 | **1627** | TRPV2 | **2674** | PKP4 | **3721** | FGF1 |
| 581 | ARHGAP17 | **1628** | PRKAG1 | **2675** | GLI2 | **3722** | MIR375 |
| 582 | CYSLTR1 | **1629** | YWHAH | **2676** | CDK5RAP3 | **3723** | RAB39A |
| 583 | ALG1 | **1630** | NDUFV1 | **2677** | PEX16 | **3724** | PEX13 |
| 584 | SYVN1 | **1631** | KHDRBS1 | **2678** | RPS19 | **3725** | CNTF |
| 585 | GDNF | **1632** | SCNN1B | **2679** | CAP1 | **3726** | ABCC11 |
| 586 | S100A1 | **1633** | PRKD3 | **2680** | RPL7A | **3727** | MIR133B |
| 587 | AQP5 | **1634** | USP4 | **2681** | ABCC5 | **3728** | CYP17A1 |
| 588 | ENO2 | **1635** | ERBB4 | **2682** | YBX3 | **3729** | GPR137B |
| 589 | CAPZA1 | **1636** | HYLS1 | **2683** | DEF6 | **3730** | VHL |
| 590 | SDHA | **1637** | SAP30BP | **2684** | IMPDH2 | **3731** | ANXA6 |
| 591 | GCDH | **1638** | LEPR | **2685** | VIPR2 | **3732** | STK38 |
| 592 | OXER1 | **1639** | DNAJB1 | **2686** | RBM15 | **3733** | ADAMTS1 |
| 593 | LHFPL2 | **1640** | BYSL | **2687** | IL13RA2 | **3734** | IL1R2 |
| 594 | RPGR | **1641** | TRIB3 | **2688** | RPS18 | **3735** | GAB2 |
| 595 | HGS | **1642** | HTRA1 | **2689** | RNASEL | **3736** | GPER1 |
| 596 | CYP3A5 | **1643** | DDX39A | **2690** | PHLPP1 | **3737** | TMPO |
| 597 | RAB22A | **1644** | MPZ | **2691** | APOH | **3738** | AKAP8 |
| 598 | GPR65 | **1645** | CISH | **2692** | MIR181A1 | **3739** | MIR17 |
| 599 | DPYD | **1646** | APOBEC3A | **2693** | NTRK3 | **3740** | MCTS1 |
| 600 | FGF23 | **1647** | RPS15 | **2694** | SP3 | **3741** | SLC2A6 |
| 601 | PIK3R4 | **1648** | SATB1 | **2695** | ENAH | **3742** | CRMA |
| 602 | G3BP2 | **1649** | APOA2 | **2696** | SNHG7 | **3743** | LPCAT2 |
| 603 | MOG | **1650** | POLR3A | **2697** | CCN1 | **3744** | SYNC |
| 604 | LY9 | **1651** | CSN1S1 | **2698** | YES1 | **3745** | VAMP3 |
| 605 | RPL6 | **1652** | CFB | **2699** | PSMA6 | **3746** | CCN4 |
| 606 | MYCN | **1653** | WDR83 | **2700** | MATR3 | **3747** | IFIT3 |
| 607 | TRIM28 | **1654** | SIRT2 | **2701** | TFE3 | **3748** | APCS |
| 608 | EIF2S1 | **1655** | GIT2 | **2702** | CYRIB | **3749** | MAP4K4 |
| 609 | FGD5-AS1 | **1656** | ATP11C | **2703** | GGT1 | **3750** | SERPINA3 |
| 610 | PKP2 | **1657** | MLEC | **2704** | IL1F10 | **3751** | HK1 |
| 611 | HNRNPH1 | **1658** | PDLIM1 | **2705** | VPS51 | **3752** | APEX1 |
| 612 | DUOX1 | **1659** | DLG3 | **2706** | MIR150 | **3753** | HGFAC |
| 613 | PLXNA3 | **1660** | ERAP1 | **2707** | CYP11A1 | **3754** | KPNA5 |
| 614 | MIR128-1 | **1661** | OTULIN | **2708** | ENHO | **3755** | DLEU1 |
| 615 | MIR125B2 | **1662** | FHOD1 | **2709** | CCDC88A | **3756** | CDKN1B |
| 616 | RBL2 | **1663** | KIF20A | **2710** | APLNR | **3757** | AKR1B1 |
| 617 | CCNT2 | **1664** | HNRNPK | **2711** | NECTIN1 | **3758** | GPR15 |
| 618 | PLD1 | **1665** | CHEK2 | **2712** | HEPH | **3759** | MYO9A |
| 619 | TNS3 | **1666** | TNFRSF4 | **2713** | TMEM201 | **3760** | FMO3 |
| 620 | PCDH15 | **1667** | TUFM | **2714** | NEU3 | **3761** | TNS1 |
| 621 | MANBA | **1668** | NR5A2 | **2715** | ADNP | **3762** | VAC14 |
| 622 | ANLN | **1669** | KL | **2716** | RUVBL2 | **3763** | RPSA |
| 623 | DDX5 | **1670** | ADAD2-AS1 | **2717** | SRRT | **3764** | PF4V1 |
| 624 | RAB21 | **1671** | CPQ | **2718** | DPEP1 | **3765** | DAPK2 |
| 625 | SRSF1 | **1672** | GNA14 | **2719** | IL24 | **3766** | APOC3 |
| 626 | ANO6 | **1673** | EPRS1 | **2720** | PTS | **3767** | TXLNA |
| 627 | ATP5F1A | **1674** | RNF31 | **2721** | MEG3 | **3768** | RFTN1 |
| 628 | GNA13 | **1675** | WNK1 | **2722** | KRT8 | **3769** | RTEL1 |
| 629 | RPL13 | **1676** | CPOX | **2723** | PTPRZ1 | **3770** | TBCD |
| 630 | PRPF3 | **1677** | COPG1 | **2724** | GRAP2 | **3771** | LTB |
| 631 | MIR140 | **1678** | ITGB7 | **2725** | FERMT2 | **3772** | ENPP1 |
| 632 | IL27RA | **1679** | PLA2G4C | **2726** | CEACAM1 | **3773** | PLEKHM2 |
| 633 | CFAP298 | **1680** | CKB | **2727** | FOSB | **3774** | MMUT |
| 634 | SARM1 | **1681** | ANTXR1 | **2728** | FUT4 | **3775** | PRX |
| 635 | XRCC1 | **1682** | CCR9 | **2729** | ATP5F1B | **3776** | SLC23A1 |
| 636 | ECSIT | **1683** | LEF1 | **2730** | SCGB1A1 | **3777** | VAMP8 |
| 637 | CA2 | **1684** | LINC01194 | **2731** | PSMA5 | **3778** | ITGA2 |
| 638 | PFN2 | **1685** | ERCC4 | **2732** | AIMP1 | **3779** | NUP153 |
| 639 | MXD1 | **1686** | IER3 | **2733** | GSTP1 | **3780** | MCUB |
| 640 | CCNB1 | **1687** | APLN | **2734** | KPNA6 | **3781** | ACSL4 |
| 641 | EFHD2 | **1688** | SRP14 | **2735** | MIR143 | **3782** | NFKBIZ |
| 642 | MAP2 | **1689** | POU5F1 | **2736** | PTHLH | **3783** | GRK5 |
| 643 | AP1S1 | **1690** | PTMA | **2737** | PRG2 | **3784** | MIR301B |
| 644 | SLC6A3 | **1691** | SNHG12 | **2738** | BBIP1 | **3785** | PI3 |
| 645 | PSMD2 | **1692** | WWP2 | **2739** | RBM17 | **3786** | CTNS |
| 646 | TCF3 | **1693** | PIK3C2B | **2740** | RRAS2 | **3787** | PDCD10 |
| 647 | LDLRAP1 | **1694** | AFP | **2741** | IAPP | **3788** | UBE2L6 |
| 648 | NLRC3 | **1695** | SLC9A8 | **2742** | CCDC47 | **3789** | CARS1 |
| 649 | CHRFAM7A | **1696** | XBP1 | **2743** | ACTC1 | **3790** | CXADR |
| 650 | TRAF5 | **1697** | TTPA | **2744** | RHOH | **3791** | SRM |
| 651 | RNF128 | **1698** | PSMD4 | **2745** | PNKD | **3792** | RBSN |
| 652 | MICB | **1699** | UCP1 | **2746** | TTYH3 | **3793** | XRCC5 |
| 653 | ETV5 | **1700** | SIAH1 | **2747** | CLDN11 | **3794** | LATS2 |
| 654 | HOTAIRM1 | **1701** | NME1 | **2748** | FGF5 | **3795** | ATP1B1 |
| 655 | ALDOA | **1702** | ANXA4 | **2749** | MIR30B | **3796** | TNPO3 |
| 656 | ABCG8 | **1703** | CCND3 | **2750** | PROK2 | **3797** | LRG1 |
| 657 | GARS1 | **1704** | CLCA1 | **2751** | SPHK2 | **3798** | BAMBI |
| 658 | GSTO1 | **1705** | HNRNPL | **2752** | EPAS1 | **3799** | PDCD4 |
| 659 | VASH1 | **1706** | FOXC1 | **2753** | SMG6 | **3800** | MIR24-2 |
| 660 | COL11A2 | **1707** | PAH | **2754** | CLRN1 | **3801** | EHBP1L1 |
| 661 | PCNA | **1708** | FRAS1 | **2755** | SWAP70 | **3802** | VLDLR |
| 662 | SPTLC2 | **1709** | MIR200B | **2756** | TOP2A | **3803** | FAU |
| 663 | SYT11 | **1710** | MIR23A | **2757** | SMURF2 | **3804** | BBS10 |
| 664 | SP110 | **1711** | EDIL3 | **2758** | CYP2E1 | **3805** | CSTA |
| 665 | GDI2 | **1712** | MEN1 | **2759** | YTHDF1 | **3806** | FFAR2 |
| 666 | CUL3 | **1713** | AQP2 | **2760** | LINC01140 | **3807** | PRLR |
| 667 | USP15 | **1714** | USH1C | **2761** | PRRC2A | **3808** | MAD1L1 |
| 668 | CYP2A6 | **1715** | RALBP1 | **2762** | CDC42SE1 | **3809** | KLF3 |
| 669 | SLC9A9 | **1716** | ETV6 | **2763** | APOL1 | **3810** | MAP6 |
| 670 | ALYREF | **1717** | ITGB5 | **2764** | IGHG1 | **3811** | SLC39A8 |
| 671 | WARS1 | **1718** | A2M | **2765** | NLRX1 | **3812** | WDR26 |
| 672 | TCOF1 | **1719** | RAN | **2766** | ACACA | **3813** | ALDH3A1 |
| 673 | HNRNPC | **1720** | DCTN1 | **2767** | AGK | **3814** | CPSF2 |
| 674 | DNAJA1 | **1721** | EGLN3 | **2768** | PPP2R1A | **3815** | PRMT5 |
| 675 | MCU | **1722** | ECE1 | **2769** | BBS7 | **3816** | PYGB |
| 676 | SLC1A4 | **1723** | ANKLE2 | **2770** | NRIR | **3817** | OTUB1 |
| 677 | HRH2 | **1724** | CHI3L2 | **2771** | NLRP2 | **3818** | STAU1 |
| 678 | PLXNA1 | **1725** | NODAL | **2772** | ABCB11 | **3819** | MLST8 |
| 679 | ARPC5 | **1726** | DDR2 | **2773** | TRAF3IP2 | **3820** | ERCC5 |
| 680 | RNF113A | **1727** | PTP4A3 | **2774** | KIF13A | **3821** | TNIP2 |
| 681 | CDC42BPA | **1728** | PDE3B | **2775** | GSDME | **3822** | ASH1L |
| 682 | TUBB4A | **1729** | FXR1 | **2776** | HRH4 | **3823** | MIR204 |
| 683 | AFDN | **1730** | TFPI | **2777** | MS4A7 | **3824** | RPL35 |
| 684 | MYH4 | **1731** | IFNL1 | **2778** | MMP10 | **3825** | NOP56 |
| 685 | DYRK1A | **1732** | SUMO2 | **2779** | MIRLET7D | **3826** | OPRK1 |
| 686 | FBXO11 | **1733** | VCP | **2780** | TCIRG1 | **3827** | STK3 |
| 687 | APOA4 | **1734** | SLC1A5 | **2781** | NORAD | **3828** | SMARCB1 |
| 688 | CLCN7 | **1735** | UNC119 | **2782** | TEC | **3829** | WDR82 |
| 689 | ABCA4 | **1736** | RAB1B | **2783** | RPL13A | **3830** | TERF2 |
| 690 | MIR181D | **1737** | MIR9-1 | **2784** | KNG1 | **3831** | PDCD6 |
| 691 | DHX33 | **1738** | MYO9B | **2785** | CYP11B1 | **3832** | FAM161A |
| 692 | H6PD | **1739** | KCNJ2 | **2786** | CD300A | **3833** | RPL28 |
| 693 | RPS11 | **1740** | MIR106A | **2787** | ECM1 | **3834** | TPO |
| 694 | CHEK1 | **1741** | MAPRE3 | **2788** | MARS1 | **3835** | BACH1 |
| 695 | NPPB | **1742** | GINS1 | **2789** | HAND2 | **3836** | MVP |
| 696 | NOP53 | **1743** | ACHE | **2790** | EDC4 | **3837** | MIRLET7A1 |
| 697 | UBE2D2 | **1744** | NF1 | **2791** | HIP1 | **3838** | CELSR2 |
| 698 | NCOA5 | **1745** | TRIM29 | **2792** | BIN1 | **3839** | LY86 |
| 699 | SLC39A10 | **1746** | BPIFA1 | **2793** | RPL26 | **3840** | ARIH2 |
| 700 | CRLF2 | **1747** | DDX3X | **2794** | SLC25A5 | **3841** | TRPC6 |
| 701 | H4C4 | **1748** | MYH7 | **2795** | MAP4 | **3842** | GAB1 |
| 702 | KDM5B | **1749** | RAI14 | **2796** | PDGFRA | **3843** | TRIM33 |
| 703 | PACSIN2 | **1750** | MIR192 | **2797** | IL1RAP | **3844** | TFAM |
| 704 | ANXA11 | **1751** | HPGD | **2798** | SLC19A1 | **3845** | ADGRV1 |
| 705 | VPS13B | **1752** | TMEM216 | **2799** | PPP1CC | **3846** | THRIL |
| 706 | IL21R | **1753** | TBX21 | **2800** | ARHGEF6 | **3847** | CTSE |
| 707 | MTA1 | **1754** | OCA2 | **2801** | GBE1 | **3848** | PLEKHA4 |
| 708 | ADGRF5 | **1755** | CALCOCO2 | **2802** | PRCC | **3849** | AGO1 |
| 709 | HNRNPF | **1756** | CSN2 | **2803** | HMGN2 | **3850** | SLC9A6 |
| 710 | STX1A | **1757** | ARHGAP25 | **2804** | PRKCG | **3851** | PLCD1 |
| 711 | KCNN1 | **1758** | EPHB1 | **2805** | USP10 | **3852** | EIF3B |
| 712 | STRAP | **1759** | SLFN11 | **2806** | LPXN | **3853** | MIR302A |
| 713 | SSB | **1760** | RPA1 | **2807** | PAX5 | **3854** | TK1 |
| 714 | ZC3H7A | **1761** | SLAMF6 | **2808** | ELK1 | **3855** | LIMK1 |
| 715 | VPS33A | **1762** | NCR2 | **2809** | SLC25A19 | **3856** | SRRM2 |
| 716 | FAS-AS1 | **1763** | SETDB1 | **2810** | CKAP4 | **3857** | RRM2 |
| 717 | NUDT21 | **1764** | GRB10 | **2811** | BCAN | **3858** | UBR5 |
| 718 | MIR203A | **1765** | ATRX | **2812** | RAB1A | **3859** | HCAR2 |
| 719 | NBR1 | **1766** | LARRPM | **2813** | RPTOR | **3860** | PHKA2 |
| 720 | WTAP | **1767** | HECTD4 | **2814** | NTF3 | **3861** | ABHD12 |
| 721 | SLC29A2 | **1768** | NCOA3 | **2815** | CYBRD1 | **3862** | FEN1 |
| 722 | NUP214 | **1769** | ARHGAP45 | **2816** | PARVB | **3863** | RAPGEF6 |
| 723 | IL36B | **1770** | PPP1R12A | **2817** | IMMT | **3864** | GABARAP |
| 724 | WDR62 | **1771** | MYO1E | **2818** | RAD51 | **3865** | SLC16A3 |
| 725 | IGFBP3 | **1772** | TLE1 | **2819** | DHX30 | **3866** | SHOC2 |
| 726 | ZHX2 | **1773** | PIM2 | **2820** | COMT | **3867** | DDX17 |
| 727 | SULT1A1 | **1774** | H2AC21 | **2821** | HK2 | **3868** | PPFIA1 |
| 728 | GNAQ | **1775** | RBX1 | **2822** | PRKAR1A | **3869** | MGAM |
| 729 | PINK1 | **1776** | P2RX1 | **2823** | RACGAP1 | **3870** | SLC8A1 |
| 730 | FER | **1777** | XPO5 | **2824** | CFI | **3871** | MIR148B |
| 731 | CACNA2D1 | **1778** | EMD | **2825** | PIKFYVE | **3872** | DIDO1 |
| 732 | HNRNPR | **1779** | PEX14 | **2826** | SMAD5 | **3873** | PAPPA |
| 733 | GOLGA2 | **1780** | ADRM1 | **2827** | TMPRSS2 | **3874** | HSPE1 |
| 734 | MORC2 | **1781** | HYAL1 | **2828** | HES1 | **3875** | IDE |
| 735 | SPAST | **1782** | DHX15 | **2829** | KDM5A | **3876** | ITCH |
| 736 | FABP5 | **1783** | S100P | **2830** | PIN1 | **3877** | HNRNPAB |
| 737 | GOLM1 | **1784** | FAM120A | **2831** | EIF2B4 | **3878** | CANX |
| 738 | RGS1 | **1785** | PSME2 | **2832** | GNPAT | **3879** | H3C1 |
| 739 | SUN2 | **1786** | RAB31 | **2833** | GPI | **3880** | SUMO1 |
| 740 | C1QC | **1787** | KIF2A | **2834** | DLGAP5 | **3881** | SORL1 |
| 741 | ARL11 | **1788** | METAP1 | **2835** | ARCN1 | **3882** | SUV39H1 |
| 742 | BCLAF1 | **1789** | SBNO1 | **2836** | PRKRA | **3883** | PLGRKT |
| 743 | LRPAP1 | **1790** | RBMX | **2837** | IK | **3884** | MIR195 |
| 744 | NOP2 | **1791** | STX7 | **2838** | HRH1 | **3885** | IFI27 |
| 745 | ABCF1 | **1792** | RHOV | **2839** | STAM | **3886** | TRIM72 |
| 746 | SAMD9L | **1793** | LIN28B | **2840** | SPTAN1 | **3887** | GNG5 |
| 747 | FGF9 | **1794** | MAX | **2841** | LMLN | **3888** | PIAS1 |
| 748 | MUC16 | **1795** | ETV4 | **2842** | IFITM3 | **3889** | HOMER3 |
| 749 | PRKAR2A | **1796** | H2AC6 | **2843** | FLNC | **3890** | BNIP3 |
| 750 | ERCC1 | **1797** | CLCN1 | **2844** | TCTN2 | **3891** | HSPA9 |
| 751 | ERG | **1798** | TAFAZZIN | **2845** | SAFB | **3892** | SLC25A1 |
| 752 | EIF4B | **1799** | APLP2 | **2846** | OIT3 | **3893** | ADAM28 |
| 753 | ABCA12 | **1800** | TARDBP | **2847** | PLA2G1B | **3894** | NEURL4 |
| 754 | RAB6A | **1801** | AP2M1 | **2848** | RAD50 | **3895** | SLC27A1 |
| 755 | NCOA2 | **1802** | RTKN | **2849** | NIN | **3896** | GRK6 |
| 756 | KIFC1 | **1803** | IBTK | **2850** | IL25 | **3897** | LINC01094 |
| 757 | POLA1 | **1804** | MX2 | **2851** | PLXNB1 | **3898** | UBXN7 |
| 758 | RPL14 | **1805** | COLEC12 | **2852** | DNER | **3899** | MIR138-2 |
| 759 | WASHC5 | **1806** | DSC1 | **2853** | UBE2I | **3900** | CDCA4 |
| 760 | PDE4DIP | **1807** | NIPBL | **2854** | FLCN | **3901** | BRD9 |
| 761 | CCAR2 | **1808** | INPP4A | **2855** | CEP104 | **3902** | KLF7 |
| 762 | CAVIN1 | **1809** | MSRA | **2856** | NELFE | **3903** | EEF2 |
| 763 | SLC2A9 | **1810** | KIF22 | **2857** | BUD23 | **3904** | DNM1 |
| 764 | SRSF5 | **1811** | INVS | **2858** | LINC00467 | **3905** | TGFBR3 |
| 765 | ITPR3 | **1812** | GPX4 | **2859** | ATP5F1D | **3906** | TPM3 |
| 766 | SLC26A3 | **1813** | PFKL | **2860** | PLSCR3 | **3907** | HUWE1 |
| 767 | CYP2C8 | **1814** | BATF2 | **2861** | AMIGO2 | **3908** | UTRN |
| 768 | RIPPLY2 | **1815** | HLA-DOA | **2862** | MIR215 | **3909** | TIA1 |
| 769 | APOBEC1 | **1816** | SEC23B | **2863** | PRKAG2 | **3910** | IL20 |
| 770 | STC1 | **1817** | TNFRSF25 | **2864** | SCYL1 | **3911** | EBI3 |
| 771 | LACTB | **1818** | PLPP1 | **2865** | BAG3 | **3912** | CPEB1 |
| 772 | RABGAP1L | **1819** | AKR1B10 | **2866** | NUP37 | **3913** | ACIN1 |
| 773 | SMAD1 | **1820** | IL15RA | **2867** | AGXT | **3914** | KAT7 |
| 774 | RPL37A | **1821** | H3-3B | **2868** | PSPC1 | **3915** | BANF1 |
| 775 | SPARCL1 | **1822** | RPLP0 | **2869** | SIK3 | **3916** | RECQL |
| 776 | SF3A1 | **1823** | USP8 | **2870** | SUCLG1 | **3917** | CCT3 |
| 777 | MIR4306 | **1824** | EIF2B1 | **2871** | TNFRSF10B | **3918** | SLCO2B1 |
| 778 | RUBCN | **1825** | RAPGEF2 | **2872** | ICMT | **3919** | LIFR |
| 779 | FKBP1A | **1826** | DNASE1 | **2873** | KIAA0753 | **3920** | OSMR |
| 780 | MAD2L1 | **1827** | H2BC11 | **2874** | UNG | **3921** | GFOD3P |
| 781 | PTGDR2 | **1828** | MAPK12 | **2875** | TRAP1 | **3922** | HKDC1 |
| 782 | PDK1 | **1829** | PLD2 | **2876** | KIF1C | **3923** | MIR1246 |
| 783 | ELL | **1830** | HAUS5 | **2877** | CPSF6 | **3924** | NUP62 |
| 784 | NHERF2 | **1831** | HNRNPA0 | **2878** | MED1 | **3925** | NECTIN2 |
| 785 | ZFYVE27 | **1832** | CNMD | **2879** | NUPR1 | **3926** | MCM3 |
| 786 | DCT | **1833** | ADGRG1 | **2880** | CCT8 | **3927** | GAMT |
| 787 | ALDOB | **1834** | SLC25A20 | **2881** | HSD3B2 | **3928** | PEX3 |
| 788 | FANCB | **1835** | TMT1B | **2882** | PAG1 | **3929** | C9orf72 |
| 789 | KCNB1 | **1836** | SH3GL1 | **2883** | WLS | **3930** | DAZAP1 |
| 790 | DCD | **1837** | NUFIP2 | **2884** | GRP | **3931** | GNA11 |
| 791 | PIP5K1A | **1838** | HCLS1 | **2885** | DOCK1 | **3932** | ARHGEF18 |
| 792 | GTF2H1 | **1839** | RRBP1 | **2886** | DUSP19 | **3933** | WNK4 |
| 793 | XPO1 | **1840** | PLLP | **2887** | HMCN1 | **3934** | LPCAT1 |
| 794 | SVIL | **1841** | SYNCRIP | **2888** | PSMA7 | **3935** | FOXQ1 |
| 795 | CDK12 | **1842** | EPB41L3 | **2889** | TWIST2 | **3936** | UPF1 |
| 796 | HDGF | **1843** | OCRL | **2890** | H2AC12 | **3937** | CSE1L |
| 797 | SRI | **1844** | MIR30C1 | **2891** | UBB | **3938** | SBF2 |
| 798 | BZW1 | **1845** | CARD8 | **2892** | MRPS7 | **3939** | PABPC4 |
| 799 | KHSRP | **1846** | IL17RB | **2893** | RIOK1 | **3940** | ZYX |
| 800 | MNX1 | **1847** | PPP1R10 | **2894** | SIK2 | **3941** | MIR4435-2HG |
| 801 | CD93 | **1848** | CD59 | **2895** | JAM2 | **3942** | SFRP2 |
| 802 | RAB29 | **1849** | RPL10A | **2896** | MIR20A | **3943** | SF3A2 |
| 803 | PDE5A | **1850** | MIR200C | **2897** | LONP1 | **3944** | BAIAP2 |
| 804 | GNA12 | **1851** | MCM6 | **2898** | RNF2 | **3945** | RSL1D1 |
| 805 | BLK | **1852** | CALB2 | **2899** | SF3B3 | **3946** | RAB9A |
| 806 | AKR1C2 | **1853** | MIR423 | **2900** | SNHG14 | **3947** | RPS8 |
| 807 | VPS28 | **1854** | FDFT1 | **2901** | PRKG1 | **3948** | FFAR3 |
| 808 | EIF4A3 | **1855** | FBN2 | **2902** | SUMO3 | **3949** | DIP2B |
| 809 | LPAR3 | **1856** | HOXB2 | **2903** | DLST | **3950** | EIF2B2 |
| 810 | CCNB2 | **1857** | LOC117152610 | **2904** | LOC117152611 | **3951** | RPL31 |
| 811 | MIA | **1858** | RPA2 | **2905** | LNCARSR | **3952** | NFE2L3 |
| 812 | CLIC3 | **1859** | CACNA2D4 | **2906** | ABCE1 | **3953** | PPP1R15A |
| 813 | EDA | **1860** | SNHG29 | **2907** | KRT1 | **3954** | NPC1L1 |
| 814 | TGM3 | **1861** | METAP2 | **2908** | BTLA | **3955** | MNDA |
| 815 | ARFGAP3 | **1862** | H2BC21 | **2909** | BANCR | **3956** | DARS1 |
| 816 | EPHB4 | **1863** | CTAG1B | **2910** | IQGAP2 | **3957** | GM2A |
| 817 | CNOT8 | **1864** | TRA | **2911** | EIF4G1 | **3958** | SUPT16H |
| 818 | DIAPH3 | **1865** | GTF2E2 | **2912** | TUT7 | **3959** | SLC25A12 |
| 819 | OSCP1 | **1866** | HBG2 | **2913** | TCERG1 | **3960** | TAL1 |
| 820 | LRRC59 | **1867** | DDX21 | **2914** | APOBEC3H | **3961** | CLDN18 |
| 821 | MIR138-1 | **1868** | STOML2 | **2915** | MAPK11 | **3962** | RPL34 |
| 822 | RASGRF1 | **1869** | PCYT1A | **2916** | HSPA2 | **3963** | MTHFR |
| 823 | CUBN | **1870** | TUBA8 | **2917** | SLC39A7 | **3964** | MYO1A |
| 824 | PIK3R3 | **1871** | NAXE | **2918** | LARP1 | **3965** | IQSEC1 |
| 825 | MAP1S | **1872** | PSME1 | **2919** | BLVRB | **3966** | NCR3 |
| 826 | LPP | **1873** | SUN1 | **2920** | RASA2 | **3967** | RPS9 |
| 827 | DDX39B | **1874** | ID1 | **2921** | CSNK2A2 | **3968** | PURA |
| 828 | CHTOP | **1875** | SELENBP1 | **2922** | RPL32 | **3969** | PPP6R1 |
| 829 | KAT6A | **1876** | SLC6A12 | **2923** | UBE2D1 | **3970** | IBSP |
| 830 | NR3C2 | **1877** | ASGR2 | **2924** | COPZ1 | **3971** | SLC4A4 |
| 831 | CEP72 | **1878** | TRNT1 | **2925** | SNRPD2 | **3972** | PLPPR5-AS1 |
| 832 | VDAC2 | **1879** | FGF19 | **2926** | TBXT | **3973** | NBR2 |
| 833 | THY1 | **1880** | VTI1B | **2927** | CNOT9 | **3974** | BMPR1A |
| 834 | AHNAK | **1881** | STEAP3 | **2928** | VPS35 | **3975** | TAOK3 |
| 835 | TNFAIP8 | **1882** | CTNND2 | **2929** | RAB32 | **3976** | CACNA1A |
| 836 | NKRF | **1883** | TSR1 | **2930** | OIP5-AS1 | **3977** | ZNF148 |
| 837 | PTGDR | **1884** | MC1R | **2931** | GLMN | **3978** | RBM10 |
| 838 | MIR326 | **1885** | STEAP4 | **2932** | MKNK2 | **3979** | SRA1 |
| 839 | LCOR | **1886** | KRT19 | **2933** | MS4A1 | **3980** | DCP1A |
| 840 | TUBGCP6 | **1887** | PCAT6 | **2934** | MATK | **3981** | ARHGAP30 |
| 841 | SPTBN4 | **1888** | KDM6A | **2935** | CCL27 | **3982** | UTP18 |
| 842 | ALPK1 | **1889** | STK16 | **2936** | EPS15 | **3983** | PPIB |
| 843 | PAX6 | **1890** | GBA2 | **2937** | SPATA2 | **3984** | MCRS1 |
| 844 | OGT | **1891** | F2RL3 | **2938** | CASP6 | **3985** | FH |
| 845 | DDIT4 | **1892** | AHCY | **2939** | POMC | **3986** | RPL36 |
| 846 | RAD21 | **1893** | PGD | **2940** | PDE4D | **3987** | MIR381 |
| 847 | ACTBL2 | **1894** | AKAP12 | **2941** | UBE2D3 | **3988** | CACYBP |
| 848 | POF1B | **1895** | MAGI2 | **2942** | LPAR1 | **3989** | TWF1 |
| 849 | CA4 | **1896** | EIF4G2 | **2943** | BCOR | **3990** | TMEM30A |
| 850 | ATG4B | **1897** | SLC7A8 | **2944** | RAB11B | **3991** | ACACB |
| 851 | CTBP1 | **1898** | RALY | **2945** | CSNK1G2 | **3992** | TNIK |
| 852 | IPO7 | **1899** | NANOG | **2946** | TRIO | **3993** | RPL3 |
| 853 | BATF3 | **1900** | MIR9-2 | **2947** | HAS2 | **3994** | DPM1 |
| 854 | SGMS2 | **1901** | CACNA2D2 | **2948** | CACNA2D3 | **3995** | WDR83OS |
| 855 | SLC25A6 | **1902** | PGM1 | **2949** | OVOL2 | **3996** | MIRLET7E |
| 856 | MIR148A | **1903** | CACNA1H | **2950** | CACNB4 | **3997** | CNN2 |
| 857 | LINC00665 | **1904** | DRD5 | **2951** | LGALS13 | **3998** | TRIM5 |
| 858 | TNFSF8 | **1905** | RNF41 | **2952** | ITGA2B | **3999** | DCHS1 |
| 859 | NDC80 | **1906** | RPL23 | **2953** | CLIP1 | **4000** | HTR2B |
| 860 | SLIT2 | **1907** | BRD2 | **2954** | MIR497 | **4001** | RAB37 |
| 861 | SLC25A11 | **1908** | SERPINH1 | **2955** | CCAR1 | **4002** | PKP3 |
| 862 | SHARPIN | **1909** | PHLDB2 | **2956** | PORCN | **4003** | DCST1-AS1 |
| 863 | PSMA1 | **1910** | PSMA3 | **2957** | FOXK2 | **4004** | DNAJA2 |
| 864 | CDX1 | **1911** | AKAP13 | **2958** | ABCB4 | **4005** | SEC13 |
| 865 | SEPTIN7 | **1912** | RPL21 | **2959** | MLH1 | **4006** | DISP1 |
| 866 | KLHDC7B-DT | **1913** | ATP6V0D2 | **2960** | PPID | **4007** | ADD1 |
| 867 | KIF13B | **1914** | BLZF1 | **2961** | BHLHE40 | **4008** | DROSHA |
| 868 | SMG7 | **1915** | ELOC | **2962** | SFN | **4009** | SOX4 |
| 869 | SGMS1 | **1916** | SNHG20 | **2963** | LDHB | **4010** | PELP1 |
| 870 | AVPR2 | **1917** | LINC00240 | **2964** | ADORA1 | **4011** | DOK1 |
| 871 | LIG3 | **1918** | ATF7 | **2965** | PRKACB | **4012** | KRT7 |
| 872 | SQOR | **1919** | FLVCR1 | **2966** | PTPRE | **4013** | HNF1B |
| 873 | MDC1 | **1920** | RHBDF1 | **2967** | TOE1 | **4014** | RPS6KA6 |
| 874 | SLC2A10 | **1921** | PITX1 | **2968** | VARS1 | **4015** | EXOSC2 |
| 875 | MARK4 | **1922** | MIR4505 | **2969** | LINC01010 | **4016** | RCAN1 |
| 876 | BBC3 | **1923** | ERAP2 | **2970** | ALDH1A2 | **4017** | ARL1 |
| 877 | PKN1 | **1924** | ODF1 | **2971** | ETFA | **4018** | ATG12 |
| 878 | LPIN1 | **1925** | HSD17B10 | **2972** | ATP9A | **4019** | TUBGCP3 |
| 879 | IRF6 | **1926** | DEFA1 | **2973** | SIPA1 | **4020** | MYL9 |
| 880 | SLC39A5 | **1927** | ZMIZ1 | **2974** | GPR174 | **4021** | SLC12A2 |
| 881 | SERPING1 | **1928** | RPL22 | **2975** | CLDN4 | **4022** | RUNX3 |
| 882 | ZIC2 | **1929** | NAPSA | **2976** | PPP6C | **4023** | KCNA2 |
| 883 | CDC42SE2 | **1930** | SLC27A4 | **2977** | REN | **4024** | CYC1 |
| 884 | TRMT112 | **1931** | BRAP | **2978** | GJB6 | **4025** | YIPF5 |
| 885 | STX5 | **1932** | RPL23A | **2979** | HNF4A | **4026** | NFYA |
| 886 | ACBD3 | **1933** | PPL | **2980** | ARPC4 | **4027** | KIF16B |
| 887 | HAUS4 | **1934** | LEMD3 | **2981** | ITGB6 | **4028** | GRHL3 |
| 888 | CDK11B | **1935** | RPS17 | **2982** | ACVR1 | **4029** | PLXDC1 |
| 889 | DANCR | **1936** | SKP1 | **2983** | SYTL2 | **4030** | INSR |
| 890 | TM6SF1 | **1937** | BAG6 | **2984** | MLPH | **4031** | UBAP2L |
| 891 | IPO5 | **1938** | EDN3 | **2985** | PRDM2 | **4032** | OSBP |
| 892 | NUP133 | **1939** | MED17 | **2986** | AIMP2 | **4033** | PRDX6 |
| 893 | RING1 | **1940** | CD6 | **2987** | S100A6 | **4034** | KLK11 |
| 894 | SCAP | **1941** | OAT | **2988** | MIR4319 | **4035** | RCN1 |
| 895 | GEM | **1942** | TWF2 | **2989** | NEIL3 | **4036** | BRD1 |
| 896 | BCAT1 | **1943** | GP2 | **2990** | MYL12B | **4037** | TPM4 |
| 897 | EFEMP1 | **1944** | CERK | **2991** | SSR1 | **4038** | SPOP |
| 898 | CDK11A | **1945** | DHFR | **2992** | POU2F1 | **4039** | TCF12 |
| 899 | SRSF7 | **1946** | NSF | **2993** | ADAM1A | **4040** | PPIG |
| 900 | LRRFIP2 | **1947** | ATP5PB | **2994** | PLCD4 | **4041** | ADGRD1 |
| 901 | ATG13 | **1948** | LTB4R2 | **2995** | SNRNP70 | **4042** | MIR32 |
| 902 | ADCYAP1R1 | **1949** | HMMR-AS1 | **2996** | TAGLN2 | **4043** | CNP |
| 903 | RETNLB | **1950** | S100A10 | **2997** | TRAPPC14 | **4044** | SFRP4 |
| 904 | PUM1 | **1951** | CORO7 | **2998** | RPL19 | **4045** | CPNE8 |
| 905 | REG4 | **1952** | RPL5 | **2999** | PDIA6 | **4046** | KLHL21 |
| 906 | CEP170 | **1953** | SBDS | **3000** | MIR19B1 | **4047** | MAGEA3 |
| 907 | MIB1 | **1954** | CDC25C | **3001** | MCM5 | **4048** | MIR186 |
| 908 | BLVRA | **1955** | RPL17 | **3002** | TWNK | **4049** | DOCK11 |
| 909 | ST2 | **1956** | RBL1 | **3003** | GCH1 | **4050** | MIR22 |
| 910 | RRP1B | **1957** | LETM1 | **3004** | SYNE2 | **4051** | ASAP1 |
| 911 | CSPP1 | **1958** | SEC61B | **3005** | GHR | **4052** | MARVELD2 |
| 912 | HSPB6 | **1959** | GAP43 | **3006** | NXF1 | **4053** | PDIA5 |
| 913 | DLX6-AS1 | **1960** | KLK3 | **3007** | PRPS1 | **4054** | CORO1C |
| 914 | ECHS1 | **1961** | MIR30C2 | **3008** | AKAP8L | **4055** | TBC1D10A |
| 915 | LENG8 | **1962** | CACNB2 | **3009** | RTCA | **4056** | ZC3H11A |
| 916 | KIF11 | **1963** | SNAP29 | **3010** | LTBP1 | **4057** | CGB5 |
| 917 | SUSD2 | **1964** | MIR199A2 | **3011** | CDA | **4058** | KCMF1 |
| 918 | SAMD9 | **1965** | FAP | **3012** | RPL9 | **4059** | RCC1 |
| 919 | GLS2 | **1966** | POTEE | **3013** | PLXNC1 | **4060** | DGCR8 |
| 920 | IL36A | **1967** | C4B | **3014** | TRN-GTT2-1 | **4061** | CAMK4 |
| 921 | PRDX3 | **1968** | RAB11FIP1 | **3015** | PTGES2 | **4062** | NPEPPS |
| 922 | SOST | **1969** | MSRB1 | **3016** | RHEB | **4063** | AXIN1 |
| 923 | LTC4S | **1970** | TRIM27 | **3017** | STK38L | **4064** | PAICS |
| 924 | SLC6A2 | **1971** | PTPN9 | **3018** | RXFP1 | **4065** | FGF8 |
| 925 | NPHP3-ACAD11 | **1972** | TRIM63 | **3019** | PELI1 | **4066** | ADGRA3 |
| 926 | CYP4V2 | **1973** | UBASH3B | **3020** | MIR324 | **4067** | TUBB1 |
| 927 | MB | **1974** | LINC01150 | **3021** | FKBP15 | **4068** | CRTC2 |
| 928 | SLC39A1 | **1975** | EIF2B5 | **3022** | PDE8A | **4069** | DGKQ |
| 929 | PAK2 | **1976** | WRAP53 | **3023** | NCSTN | **4070** | CSTF2 |
| 930 | HNRNPA3 | **1977** | MAP3K11 | **3024** | CLDN23 | **4071** | RPS16 |
| 931 | ARHGEF12 | **1978** | HOXA9 | **3025** | COMP | **4072** | PRRC2C |
| 932 | RAPGEF1 | **1979** | PSMB10 | **3026** | TP53BP1 | **4073** | PRDX4 |
| 933 | ANAPC2 | **1980** | AGPAT1 | **3027** | MIR124-3 | **4074** | ALKBH3 |
| 934 | ARHGEF28 | **1981** | ZNF202 | **3028** | VAMP2 | **4075** | ARHGDIB |
| 935 | RARG | **1982** | LCN10 | **3029** | NFE2L1 | **4076** | ARHGAP5 |
| 936 | RETSAT | **1983** | WASHC4 | **3030** | ARL5B | **4077** | ALDH18A1 |
| 937 | RAB7B | **1984** | HLA-DRB4 | **3031** | SMC2 | **4078** | TBC1D15 |
| 938 | SAFB2 | **1985** | TULP1 | **3032** | ATG14 | **4079** | KRT79 |
| 939 | SPRY1 | **1986** | PER1 | **3033** | NSDHL | **4080** | MIR181B1 |
| 940 | ALDH1A1 | **1987** | USF2 | **3034** | DGAT2 | **4081** | PKP1 |
| 941 | APOBEC3C | **1988** | POLA2 | **3035** | TRN-GTT2-7 | **4082** | POLDIP3 |
| 942 | STX12 | **1989** | HAUS6 | **3036** | TPM1 | **4083** | DYNC1H1 |
| 943 | HSPA14 | **1990** | CRIPTO | **3037** | SKP2 | **4084** | SPINK1 |
| 944 | PAXIP1 | **1991** | CLUH | **3038** | NALT1 | **4085** | VGF |
| 945 | NCOA1 | **1992** | H4C16 | **3039** | GDI1 | **4086** | DSP |
| 946 | IGFBP4 | **1993** | RPS20 | **3040** | ARFGEF2 | **4087** | RHOU |
| 947 | POLR2B | **1994** | RPL24 | **3041** | LINC00662 | **4088** | LY75 |
| 948 | CASP14 | **1995** | FFAR1 | **3042** | SYTL1 | **4089** | EPS8 |
| 949 | ARHGAP19-SLIT1 | **1996** | CYP7A1 | **3043** | ITPR2 | **4090** | PSMF1 |
| 950 | SORBS1 | **1997** | STX2 | **3044** | DUT | **4091** | RNY3 |
| 951 | EFNA2 | **1998** | ANGPTL3 | **3045** | PLK2 | **4092** | GSTM2 |
| 952 | KRT77 | **1999** | DDA1 | **3046** | RAB3GAP1 | **4093** | PSMC2 |
| 953 | NECAB3 | **2000** | MIR92A2 | **3047** | NUP107 | **4094** | CYRIA |
| 954 | RAP2B | **2001** | MIR199A1 | **3048** | KRT85 | **4095** | NACA |
| 955 | FCHO2 | **2002** | TSG101 | **3049** | RPS4X | **4096** | CALML3 |
| 956 | ACTG2 | **2003** | MYCBP2 | **3050** | PRMT3 | **4097** | TNFRSF13C |
| 957 | ZFP91 | **2004** | ATP1B2 | **3051** | SON | **4098** | PLOD2 |
| 958 | CKLF | **2005** | MIR92A1 | **3052** | TNNT2 | **4099** | OXT |
| 959 | DCTN4 | **2006** | CTAGE1 | **3053** | SOX9 | **4100** | SMPX |
| 960 | GUK1 | **2007** | FIG4 | **3054** | INF2 | **4101** | VEZF1 |
| 961 | ATP11A | **2008** | NAAA | **3055** | RBM14 | **4102** | IDI1 |
| 962 | MPIG6B | **2009** | RAB34 | **3056** | TONSL | **4103** | RBCK1 |
| 963 | PPA1 | **2010** | DHX40 | **3057** | ACVR1B | **4104** | GSTT2B |
| 964 | MAST2 | **2011** | RPS10 | **3058** | RPL4 | **4105** | RPS13 |
| 965 | NPR1 | **2012** | HMOX2 | **3059** | STRN4 | **4106** | CDKN2B-AS1 |
| 966 | PSME3 | **2013** | RDH11 | **3060** | SNHG17 | **4107** | LMAN1 |
| 967 | HINFP | **2014** | MIR769 | **3061** | EVL | **4108** | RREB1 |
| 968 | DOCK4 | **2015** | TOP1 | **3062** | NDRG1 | **4109** | CLPB |
| 969 | AXIN2 | **2016** | CDC42EP1 | **3063** | STXBP1 | **4110** | MIR1291 |
| 970 | JAG2 | **2017** | PTH1R | **3064** | STK39 | **4111** | TRIM56 |
| 971 | HAR1B | **2018** | CCT6A | **3065** | LIMK2 | **4112** | WDR6 |
| 972 | MIR296 | **2019** | VEGFB | **3066** | EIF3A | **4113** | PTGES3 |
| 973 | GATAD2A | **2020** | XIST | **3067** | MARCHF2 | **4114** | GRIN1 |
| 974 | SLC22A12 | **2021** | GTF2I | **3068** | PEPD | **4115** | DES |
| 975 | ELMO2 | **2022** | HMMR | **3069** | SPC25 | **4116** | SH3KBP1 |
| 976 | GPR37 | **2023** | RPL35A | **3070** | RPS6KA3 | **4117** | CALCRL |
| 977 | RAB3B | **2024** | TFF2 | **3071** | THRAP3 | **4118** | RPS23 |
| 978 | COL11A1 | **2025** | EIF2S3 | **3072** | CBFB | **4119** | FALEC |
| 979 | GYPA | **2026** | CACNA1G | **3073** | TRPM4 | **4120** | SERF2 |
| 980 | CRNDE | **2027** | RPRD2 | **3074** | CDC42BPB | **4121** | OPA1 |
| 981 | MACC1 | **2028** | PTP4A1 | **3075** | MIR543 | **4122** | LAMA2 |
| 982 | SLC20A1 | **2029** | SERPINB9 | **3076** | TKTL1 | **4123** | MIR16-1 |
| 983 | BRCA2 | **2030** | GULP1 | **3077** | POLR1A | **4124** | MIR9-3 |
| 984 | STAMBP | **2031** | NDRG2 | **3078** | TNFRSF21 | **4125** | STAM2 |
| 985 | PRPF6 | **2032** | MYL2 | **3079** | NT5DC2 | **4126** | LAT |
| 986 | GLO1 | **2033** | SYNPO | **3080** | PEA15 | **4127** | PMP22 |
| 987 | WNT6 | **2034** | SPDEF | **3081** | TBL1XR1 | **4128** | PBRM1 |
| 988 | KLRC1 | **2035** | ZNF143 | **3082** | PPP1R7 | **4129** | DEFA5 |
| 989 | GAR1 | **2036** | TFPT | **3083** | HOTAIR | **4130** | NDUFV3 |
| 990 | UACA | **2037** | CNTNAP1 | **3084** | PROCR | **4131** | SRD5A1 |
| 991 | SLC6A4 | **2038** | CPSF7 | **3085** | RPS29 | **4132** | MIR206 |
| 992 | RPS2 | **2039** | PRKG2 | **3086** | ANXA2P2 | **4133** | MIR30D |
| 993 | HBD | **2040** | PLS3 | **3087** | HPGDS | **4134** | POLR2C |
| 994 | RAD18 | **2041** | HSPA1B | **3088** | MYO5C | **4135** | CSNK1G1 |
| 995 | TYRP1 | **2042** | TNFAIP8L1 | **3089** | H3C14 | **4136** | MIR16-2 |
| 996 | PIBF1 | **2043** | ZG16B | **3090** | FUBP3 | **4137** | GANAB |
| 997 | ENPP2 | **2044** | ZNF281 | **3091** | ZNF687 | **4138** | CYTH3 |
| 998 | A2ML1 | **2045** | UVRAG | **3092** | GJB1 | **4139** | MLXIPL |
| 999 | HCAR3 | **2046** | PHLDB1 | **3093** | COL10A1 | **4140** | PTPRG |
| 1000 | TRL-TAG1-1 | **2047** | HLTF | **3094** | SMARCA2 | **4141** | LFNG |
| 1001 | TIMM8A | **2048** | MIR340 | **3095** | CAST | **4142** | TASOR |
| 1002 | ERO1A | **2049** | DHX38 | **3096** | SAMSN1 | **4143** | APRT |
| 1003 | INTS1 | **2050** | DHCR24 | **3097** | ANAPC1 | **4144** | UTS2 |
| 1004 | ARHGEF19 | **2051** | DNAJB6 | **3098** | BRD3 | **4145** | TUBB4B |
| 1005 | STIM2 | **2052** | KRT10 | **3099** | MIR671 | **4146** | C1QB |
| 1006 | NLRC5 | **2053** | TH | **3100** | WDR77 | **4147** | MED4 |
| 1007 | NPY | **2054** | PTGER1 | **3101** | CS | **4148** | DKC1 |
| 1008 | TAX1BP3 | **2055** | KLHL12 | **3102** | AFG3L2 | **4149** | YKT6 |
| 1009 | UBE2L3 | **2056** | TIAM2 | **3103** | RSPO2 | **4150** | IRS1 |
| 1010 | RPPH1 | **2057** | MIR10B | **3104** | SIAH2 | **4151** | RPL8 |
| 1011 | SPRED1 | **2058** | CAHM | **3105** | MAZ | **4152** | UBE2S |
| 1012 | GNB3 | **2059** | CCK | **3106** | ETF1 | **4153** | CEACAM7 |
| 1013 | LGALS4 | **2060** | PICALM | **3107** | SHB | **4154** | CHD3 |
| 1014 | KIF3B | **2061** | FABP12 | **3108** | CLASRP | **4155** | SNW1 |
| 1015 | SUPT6H | **2062** | ATP6V1E1 | **3109** | TFG | **4156** | FLG2 |
| 1016 | HAUS3 | **2063** | NPHP4 | **3110** | APH1A | **4157** | RTN3 |
| 1017 | AMBP | **2064** | CCNE1 | **3111** | MIR181A2 | **4158** | TXNDC5 |
| 1018 | KDM2A | **2065** | COLGALT1 | **3112** | HLA-F-AS1 | **4159** | DEK |
| 1019 | RARS1 | **2066** | DGKA | **3113** | SYT1 | **4160** | TOP3B |
| 1020 | PRPS2 | **2067** | RPL30 | **3114** | SCAMP3 | **4161** | SEC16A |
| 1021 | TACR2 | **2068** | MPG | **3115** | ARF1 | **4162** | ZNF451 |
| 1022 | MED20 | **2069** | IARS1 | **3116** | DDX50 | **4163** | RRP12 |
| 1023 | F7 | **2070** | GFM1 | **3117** | GNB5 | **4164** | SLC6A6 |
| 1024 | CRH | **2071** | CCNL2 | **3118** | MIR130B | **4165** | HOXA10 |
| 1025 | HELZ2 | **2072** | PIP5K1B | **3119** | LAMTOR1 | **4166** | LINC01727 |
| 1026 | RLN2 | **2073** | RALGDS | **3120** | PCNX3 | **4167** | HAUS1 |
| 1027 | H3-3A | **2074** | ITGA1 | **3121** | TRIM59 | **4168** | NDUFAB1 |
| 1028 | SIRT4 | **2075** | EPS15L1 | **3122** | ZDHHC5 | **4169** | SNRPB |
| 1029 | AFAP1-AS1 | **2076** | UBE2O | **3123** | SHBG | **4170** | ATG16L2 |
| 1030 | ADAMTS5 | **2077** | MMP11 | **3124** | RYK | **4171** | ATP6V1H |
| 1031 | MIR100 | **2078** | SLC47A1 | **3125** | SLC12A3 | **4172** | APAF1 |
| 1032 | H1-3 | **2079** | ANKRD28 | **3126** | B3GALNT2 | **4173** | PCBP1 |
| 1033 | NID1 | **2080** | ALPP | **3127** | ANO1 | **4174** | STT3A |
| 1034 | SMYD3 | **2081** | TBC1D14 | **3128** | MAG | **4175** | MPZL1 |
| 1035 | CNTROB | **2082** | KIR2DL4 | **3129** | NDUFS3 | **4176** | GTF3C4 |
| 1036 | IFT74 | **2083** | PCBP2 | **3130** | TOP2B | **4177** | H2BC12L |
| 1037 | RAB23 | **2084** | MIR27B | **3131** | YLPM1 | **4178** | CDH3 |
| 1038 | NPR3 | **2085** | PRSS3 | **3132** | MED16 | **4179** | TRABD |
| 1039 | ZCCHC8 | **2086** | PREB | **3133** | EFNB1 | **4180** | INTS2 |
| 1040 | UBE3A | **2087** | WDR47 | **3134** | ISOC2 | **4181** | LAMB2 |
| 1041 | SARAF | **2088** | ABCG4 | **3135** | GSTK1 | **4182** | DMAP1 |
| 1042 | DNAJA3 | **2089** | SMPD2 | **3136** | DDRGK1 | **4183** | MICA |
| 1043 | RTRAF | **2090** | JPT2 | **3137** | PSMD10 | **4184** | FGF20 |
| 1044 | PPM1D | **2091** | SERPINB3 | **3138** | GYPE | **4185** | RAP2A |
| 1045 | TIAL1 | **2092** | RHBG | **3139** | GH1 | **4186** | TUT4 |
| 1046 | ANXA3 | **2093** | SLA2 | **3140** | LGR4 | **4187** | DNAJC7 |
| 1047 | YTHDC2 | **2094** | STARD8 | **3141** | H2AZ1 |  | 0 |

**Table S10 Union targets of MP in Online OMIM and GeneCards**

| NO | Target | NO | Target | NO | Target | NO | Target |
| --- | --- | --- | --- | --- | --- | --- | --- |
| 1 | CSF1 | **1055** | CSF2 | **2109** | MIF | **3163** | TNF |
| 2 | IL10 | **1056** | MSR1 | **2110** | TLR4 | **3164** | IL1B |
| 3 | IL6 | **1057** | IFNG | **2111** | CCL2 | **3165** | CSF1R |
| 4 | STAT3 | **1058** | CCR5 | **2112** | CXCL8 | **3166** | TLR2 |
| 5 | IL4 | **1059** | NLRP3 | **2113** | CCL3 | **3167** | MARCO |
| 6 | ABCA1 | **1060** | CSF2RA | **2114** | MST1 | **3168** | STAT1 |
| 7 | MST1R | **1061** | CD163 | **2115** | CCL5 | **3169** | CCR2 |
| 8 | CD4 | **1062** | MMP9 | **2116** | CCL4 | **3170** | ITGAM |
| 9 | TGFB1 | **1063** | MAEA | **2117** | MRC1 | **3171** | CRYAB |
| 10 | CD274 | **1064** | CDC42 | **2118** | CD68 | **3172** | PARD3 |
| 11 | MMD | **1065** | CCR1 | **2119** | CXCR4 | **3173** | NLRC4 |
| 12 | CCL18 | **1066** | PTGS2 | **2120** | VEGFA | **3174** | HAVCR2 |
| 13 | CSF3 | **1067** | EPHA2 | **2121** | MMP12 | **3175** | SCRIB |
| 14 | PITX3 | **1068** | CHMP4B | **2122** | IL3 | **3176** | NOS2 |
| 15 | IL18 | **1069** | PPARG | **2123** | ABCG1 | **3177** | CD14 |
| 16 | JAK2 | **1070** | NFKB1 | **2124** | CXCL12 | **3178** | IL1A |
| 17 | CERNA3 | **1071** | IL2 | **2125** | WDPCP | **3179** | APOE |
| 18 | HMGB1 | **1072** | MAPK1 | **2126** | SLC11A1 | **3180** | CCL20 |
| 19 | FASLG | **1073** | CCL22 | **2127** | CRYBB2 | **3181** | STK11 |
| 20 | MACIR | **1074** | CD86 | **2128** | MTOR | **3182** | CRYAA |
| 21 | CD47 | **1075** | NR1H3 | **2129** | PARD6A | **3183** | CD36 |
| 22 | RIPOR1 | **1076** | UNC13D | **2130** | PRF1 | **3184** | RAC1 |
| 23 | ICAM1 | **1077** | PIK3CG | **2131** | HIF1A | **3185** | TNFSF11 |
| 24 | AKT1 | **1078** | WAS | **2132** | TLR3 | **3186** | LINC02605 |
| 25 | SIRPA | **1079** | IL13 | **2133** | CYBB | **3187** | RHOA |
| 26 | CFTR | **1080** | ITGB2 | **2134** | IL34 | **3188** | NFKBIA |
| 27 | IL33 | **1081** | CXCL10 | **2135** | SYK | **3189** | CX3CR1 |
| 28 | IL12B | **1082** | PDCD1 | **2136** | HLA-DRB1 | **3190** | STAT6 |
| 29 | TREM2 | **1083** | GDF15 | **2137** | CES1 | **3191** | LIPA |
| 30 | CD44 | **1084** | HMOX1 | **2138** | APOA1 | **3192** | PTEN |
| 31 | MYD88 | **1085** | LLGL2 | **2139** | IL5 | **3193** | CCR7 |
| 32 | SCARB1 | **1086** | EGFR | **2140** | VIM | **3194** | TP53 |
| 33 | CHI3L1 | **1087** | GBF1 | **2141** | MAP2K1 | **3195** | CASP3 |
| 34 | PRKCZ | **1088** | DVL2 | **2142** | LYST | **3196** | MEFV |
| 35 | XIAP | **1089** | IL2RA | **2143** | TNFRSF1A | **3197** | JUN |
| 36 | TLR9 | **1090** | BDNF-AS | **2144** | FAS | **3198** | CCR6 |
| 37 | TLR7 | **1091** | SOCS3 | **2145** | CTNNB1 | **3199** | NFE2L2 |
| 38 | LAMP1 | **1092** | CCL11 | **2146** | CCR3 | **3200** | SPI1 |
| 39 | IRF1 | **1093** | RAB27A | **2147** | CD40 | **3201** | P2RX7 |
| 40 | GSK3B | **1094** | IRF3 | **2148** | VIPAS39 | **3202** | SIGLEC1 |
| 41 | VANGL2 | **1095** | STXBP2 | **2149** | IDO1 | **3203** | PRKCA |
| 42 | LEMD2 | **1096** | WNT5A | **2150** | KLF4 | **3204** | IL1RN |
| 43 | CXCL2 | **1097** | IRF8 | **2151** | SGPP1 | **3205** | CD74 |
| 44 | MARK2 | **1098** | NOD2 | **2152** | FOXP3 | **3206** | FUZ |
| 45 | RELA | **1099** | PIK3R1 | **2153** | IL17A | **3207** | SOD2-OT1 |
| 46 | MAF | **1100** | MIP | **2154** | LPL | **3208** | PWAR1 |
| 47 | SLC7A7 | **1101** | ICAM3 | **2155** | CAPG | **3209** | PTK2 |
| 48 | HAMP | **1102** | CD81 | **2156** | MERTK | **3210** | YAP1 |
| 49 | CXCL1 | **1103** | TLR6 | **2157** | CHIT1 | **3211** | AIF1 |
| 50 | STING1 | **1104** | CRB1 | **2158** | CASP4 | **3212** | CXCR3 |
| 51 | OLR1 | **1105** | MUC1 | **2159** | CXCL9 | **3213** | IFNA1 |
| 52 | MAPK14 | **1106** | TJP1 | **2160** | COL12A1 | **3214** | STX11 |
| 53 | CCL7 | **1107** | CRP | **2161** | PTK2B | **3215** | CBS |
| 54 | CPLANE1 | **1108** | CCL19 | **2162** | IKBKB | **3216** | IL12A |
| 55 | SOCS1 | **1109** | CD209 | **2163** | LIF | **3217** | PARD6B |
| 56 | SLC40A1 | **1110** | TLR8 | **2164** | CCR8 | **3218** | CD5L |
| 57 | CCL17 | **1111** | PLAUR | **2165** | MAPK8 | **3219** | IRF5 |
| 58 | PRKCD | **1112** | IKBKG | **2166** | CYBA | **3220** | BMP6 |
| 59 | CCL21 | **1113** | MAPK3 | **2167** | IFNB1 | **3221** | CNR2 |
| 60 | SPP1 | **1114** | IL4R | **2168** | RAC2 | **3222** | MIRLET7C |
| 61 | SMAD3 | **1115** | IL23A | **2169** | BTK | **3223** | KITLG |
| 62 | SLC11A2 | **1116** | PYCARD | **2170** | SHH | **3224** | NR1H2 |
| 63 | S100A9 | **1117** | GATA3 | **2171** | TREM1 | **3225** | MYC |
| 64 | SIRT1 | **1118** | IL12RB1 | **2172** | PRKCI | **3226** | HCK |
| 65 | ADIPOQ | **1119** | INPPL1 | **2173** | PATJ | **3227** | SRC |
| 66 | MIR125A | **1120** | MMP2 | **2174** | ITGAL | **3228** | C5AR1 |
| 67 | SMAD2 | **1121** | BRAF | **2175** | TYROBP | **3229** | NCF2 |
| 68 | SH2D1A | **1122** | TNFRSF11A | **2176** | GJA3 | **3230** | STAT5A |
| 69 | HSPA5 | **1123** | PTPN11 | **2177** | MIR7-3HG | **3231** | CRYBA1 |
| 70 | SCARNA5 | **1124** | GAS5 | **2178** | LGALS3 | **3232** | CTLA4 |
| 71 | IL18R1 | **1125** | CD80 | **2179** | SFTPD | **3233** | CTSD |
| 72 | IFNGR1 | **1126** | MSN | **2180** | SMPD1 | **3234** | NPC1 |
| 73 | FOS | **1127** | PLA2G7 | **2181** | IL15 | **3235** | DLG1 |
| 74 | CASP8 | **1128** | LYN | **2182** | LLGL1 | **3236** | CX3CL1 |
| 75 | CYP27A1 | **1129** | MIR146B | **2183** | LDLR | **3237** | S100A8 |
| 76 | PTPRC | **1130** | KDM6B | **2184** | CETP | **3238** | GBA1 |
| 77 | FPR2 | **1131** | SOD1 | **2185** | MIR155 | **3239** | PGR-AS1 |
| 78 | NOTCH1 | **1132** | SERPINE1 | **2186** | CD9 | **3240** | ADA |
| 79 | ARF6 | **1133** | ERCC3 | **2187** | GATA2 | **3241** | MMP14 |
| 80 | H19 | **1134** | TIMP1 | **2188** | FCGR3A | **3242** | FCGR1A |
| 81 | PIK3CA | **1135** | CRB2 | **2189** | CLEC10A | **3243** | MIR21 |
| 82 | SAMHD1 | **1136** | SFTPA1 | **2190** | ROCK1 | **3244** | ITK |
| 83 | PANX1 | **1137** | CLEC7A | **2191** | NCKAP1L | **3245** | RHOG |
| 84 | CEBPB | **1138** | ARG1 | **2192** | FN1 | **3246** | FABP4 |
| 85 | LACC1 | **1139** | PTPN6 | **2193** | CTSL | **3247** | CCR4 |
| 86 | CREB1 | **1140** | TF | **2194** | GRB2 | **3248** | CORO1A |
| 87 | ACTA2 | **1141** | MMP1 | **2195** | CDH1 | **3249** | RAF1 |
| 88 | AP3B1 | **1142** | FLT1 | **2196** | SFTPB | **3250** | NPC2 |
| 89 | BCL2L1 | **1143** | PPARA | **2197** | FCGR2A | **3251** | MCL1 |
| 90 | ELAVL1 | **1144** | MPO | **2198** | VANGL1 | **3252** | IL2RG |
| 91 | NR4A1 | **1145** | PIK3CD | **2199** | STAT2 | **3253** | PLA2G4A |
| 92 | ERBB2 | **1146** | CASP1 | **2200** | AHR | **3254** | LILRB1 |
| 93 | ESR1 | **1147** | EZH2 | **2201** | LGALS9 | **3255** | BAX |
| 94 | IL4I1 | **1148** | TBK1 | **2202** | EGR1 | **3256** | ZFP36 |
| 95 | ITGB1 | **1149** | IL32 | **2203** | TFRC | **3257** | PLA2G5 |
| 96 | INPP5D | **1150** | ERCC2 | **2204** | ACP5 | **3258** | TNFAIP3 |
| 97 | RAB8A | **1151** | CMKLR1 | **2205** | TFEB | **3259** | BSG |
| 98 | MAFB | **1152** | MFHAS1 | **2206** | ZC3H12A | **3260** | MPL |
| 99 | IGF1 | **1153** | IRF7 | **2207** | FLT3 | **3261** | SOAT1 |
| 100 | CTSK | **1154** | CXCR2 | **2208** | CXCL16 | **3262** | TRAF6 |
| 101 | IL1RL1 | **1155** | EPO | **2209** | TGFB2 | **3263** | NEU1 |
| 102 | TNFSF10 | **1156** | SLAMF1 | **2210** | ANPEP | **3264** | CD40LG |
| 103 | MAPK10 | **1157** | ALOX15 | **2211** | TPP1 | **3265** | GUSB |
| 104 | SFTPC | **1158** | CAV1 | **2212** | ELN | **3266** | CR1 |
| 105 | CLEC4E | **1159** | LILRB2 | **2213** | SQSTM1 | **3267** | FOLR2 |
| 106 | GSDMD | **1160** | CD70 | **2214** | ACE2 | **3268** | ADAM17 |
| 107 | PARP14 | **1161** | APP | **2215** | FMNL1 | **3269** | CARD9 |
| 108 | JAK1 | **1162** | ELANE | **2216** | APOB | **3270** | TIAM1 |
| 109 | GC | **1163** | HGF | **2217** | CCL1 | **3271** | EP300 |
| 110 | TARS1 | **1164** | ABCB1 | **2218** | IL11 | **3272** | GPNMB |
| 111 | ITGAX | **1165** | IRAK1 | **2219** | BST2 | **3273** | CTSB |
| 112 | VCAM1 | **1166** | CAT | **2220** | CLEC4D | **3274** | CASP9 |
| 113 | ABL1 | **1167** | DAB2 | **2221** | JAK3 | **3275** | ALOX5 |
| 114 | ETS1 | **1168** | BCL2 | **2222** | IL37 | **3276** | CHUK |
| 115 | PARP1 | **1169** | IRGM | **2223** | QKI | **3277** | F3 |
| 116 | CCL14 | **1170** | HSF4 | **2224** | SERPINA1 | **3278** | HDAC1 |
| 117 | PF4 | **1171** | FOXO1 | **2225** | SUCNR1 | **3279** | STK4 |
| 118 | STAB1 | **1172** | RHOD | **2226** | IRAK3 | **3280** | PCSK9 |
| 119 | FADD | **1173** | JMJD6 | **2227** | CXCL11 | **3281** | ANXA1 |
| 120 | DAAM1 | **1174** | IL6R | **2228** | HBEGF | **3282** | CASP5 |
| 121 | MAPT | **1175** | LEP | **2229** | FCGR2B | **3283** | PATL2 |
| 122 | HRAS | **1176** | MMP3 | **2230** | VAV1 | **3284** | CD63 |
| 123 | HSPD1 | **1177** | TRA-TGC7-1 | **2231** | OSM | **3285** | ALB |
| 124 | TLR5 | **1178** | TICAM1 | **2232** | DPP4 | **3286** | ANXA2 |
| 125 | GZMB | **1179** | IL1R1 | **2233** | PJA2 | **3287** | CXCL5 |
| 126 | CLEC5A | **1180** | GSN | **2234** | TNFSF13B | **3288** | RIGI |
| 127 | HDAC6 | **1181** | SHC1 | **2235** | C3 | **3289** | EZR |
| 128 | PLAU | **1182** | KRAS | **2236** | PTGS1 | **3290** | HLA-G |
| 129 | NCF4 | **1183** | PWAR4 | **2237** | IL7 | **3291** | GPBAR1 |
| 130 | CD200R1 | **1184** | CXCL13 | **2238** | SP1 | **3292** | CSF3R |
| 131 | ITPR1 | **1185** | AXL | **2239** | IGHE | **3293** | CARMIL2 |
| 132 | CCL8 | **1186** | TGFBR1 | **2240** | PON1 | **3294** | FPR1 |
| 133 | DEFB4A | **1187** | IFIH1 | **2241** | NRAS | **3295** | CD27 |
| 134 | AIM2 | **1188** | MRC2 | **2242** | VAV3 | **3296** | HFE |
| 135 | CD8A | **1189** | STAT5B | **2243** | PLIN2 | **3297** | METTL3 |
| 136 | CBL | **1190** | LCK | **2244** | SCARB2 | **3298** | MPP1 |
| 137 | ZAP70 | **1191** | NUMA1 | **2245** | NPHP1 | **3299** | HSPA4 |
| 138 | ABCC2 | **1192** | CPLANE2 | **2246** | LILRB4 | **3300** | RIPK1 |
| 139 | PTK7 | **1193** | THBS1 | **2247** | CTSA | **3301** | DOCK8 |
| 140 | PPBP | **1194** | IL3RA | **2248** | PRKAA1 | **3302** | FAM76B |
| 141 | ZEB1 | **1195** | CCL23 | **2249** | COL4A3 | **3303** | ABCG2 |
| 142 | ARHGEF2 | **1196** | MIR146A | **2250** | ETS2 | **3304** | PLA2G2A |
| 143 | ROCK2 | **1197** | TLR1 | **2251** | TRPV4 | **3305** | MYO18A |
| 144 | CXCR1 | **1198** | MAPK9 | **2252** | RARRES2 | **3306** | IL27 |
| 145 | CCL15 | **1199** | PRKD1 | **2253** | FOXO3 | **3307** | MYDGF |
| 146 | FGL2 | **1200** | SELL | **2254** | ACTG1 | **3308** | CXCL14 |
| 147 | CD1C | **1201** | ITIH4 | **2255** | GRN | **3309** | PRKCE |
| 148 | APC | **1202** | PPP1CA | **2256** | CCN2 | **3310** | BRD4 |
| 149 | PTPN2 | **1203** | LCN2 | **2257** | POSTN | **3311** | GLB1 |
| 150 | ACOD1 | **1204** | FRYL | **2258** | AGER | **3312** | IRF4 |
| 151 | ATP7B | **1205** | SI | **2259** | CIITA | **3313** | CD46 |
| 152 | CDK6 | **1206** | MIR98 | **2260** | CD69 | **3314** | PDGFRB |
| 153 | SLC2A1 | **1207** | CD1A | **2261** | DYSF | **3315** | KIT |
| 154 | CGAS | **1208** | LIN7A | **2262** | TBXAS1 | **3316** | CCL26 |
| 155 | RACK1 | **1209** | FGR | **2263** | PRTN3 | **3317** | CD247 |
| 156 | TSPO | **1210** | SREBF1 | **2264** | RAB11A | **3318** | MYO5B |
| 157 | VEGFC | **1211** | PSAP | **2265** | FCER2 | **3319** | ABCC1 |
| 158 | TNFAIP8L2 | **1212** | APOBEC3G | **2266** | LY96 | **3320** | CLEC12A |
| 159 | AMOT | **1213** | KLRK1 | **2267** | PTCH1 | **3321** | CD83 |
| 160 | MAP1LC3A | **1214** | NAGA | **2268** | RAP1B | **3322** | IKZF1 |
| 161 | RARA | **1215** | MPP7 | **2269** | ACE | **3323** | SLC22A5 |
| 162 | KRIT1 | **1216** | AKT2 | **2270** | IFNGR2 | **3324** | TGM2 |
| 163 | BMP4 | **1217** | MDM2 | **2271** | STUB1 | **3325** | PLCG2 |
| 164 | RAP1A | **1218** | RUNX2 | **2272** | TGFA | **3326** | CXCR6 |
| 165 | MMP7 | **1219** | IL9 | **2273** | CSK | **3327** | PXN |
| 166 | LTA | **1220** | PRKCB | **2274** | NAIP | **3328** | CXCL3 |
| 167 | CTSG | **1221** | ITGAV | **2275** | LTF | **3329** | TNFAIP6 |
| 168 | CD1D | **1222** | TIMP3 | **2276** | CYP27B1 | **3330** | OCLN |
| 169 | ERN1 | **1223** | FGFR1 | **2277** | TIRAP | **3331** | ARSA |
| 170 | NPM1 | **1224** | S100B | **2278** | MBL2 | **3332** | CD276 |
| 171 | PECAM1 | **1225** | COPA | **2279** | DMBT1 | **3333** | PDPK1 |
| 172 | CCNT1 | **1226** | HBB | **2280** | BMP2 | **3334** | SLC3A2 |
| 173 | SMURF1 | **1227** | LIN7B | **2281** | ACTR2 | **3335** | SMAD4 |
| 174 | AP3D1 | **1228** | TGFBR2 | **2282** | SNCA | **3336** | CD3E |
| 175 | DDIT3 | **1229** | HDAC2 | **2283** | GALC | **3337** | VSIG4 |
| 176 | HLA-DRA | **1230** | HLA-DPB1 | **2284** | NOD1 | **3338** | PLA2G6 |
| 177 | FES | **1231** | DCAF1 | **2285** | RB1 | **3339** | CD38 |
| 178 | NR3C1 | **1232** | MIR155HG | **2286** | CTSS | **3340** | NHERF1 |
| 179 | LGALS1 | **1233** | PTGER4 | **2287** | IGF2BP2 | **3341** | VPS33B |
| 180 | MAP3K8 | **1234** | IL6ST | **2288** | MPLKIP | **3342** | GJB2 |
| 181 | RASGRP1 | **1235** | SLC48A1 | **2289** | PTPN1 | **3343** | PKHD1 |
| 182 | CCL25 | **1236** | LAMP2 | **2290** | SNAI1 | **3344** | FLT3LG |
| 183 | BECN1 | **1237** | CYCS | **2291** | CD33 | **3345** | SLC29A3 |
| 184 | VTCN1 | **1238** | ARHGAP35 | **2292** | CDKN3 | **3346** | S100A4 |
| 185 | B2M | **1239** | MFGE8 | **2293** | ARG2 | **3347** | JAG1 |
| 186 | VDR | **1240** | TRIM32 | **2294** | NOX4 | **3348** | TSC22D3 |
| 187 | TGFB3 | **1241** | ACTR3 | **2295** | SLC9A1 | **3349** | CD84 |
| 188 | ANXA5 | **1242** | CREBBP | **2296** | ITGB3 | **3350** | ACTA1 |
| 189 | IKBKE | **1243** | TNFRSF1B | **2297** | NFATC1 | **3351** | LBP |
| 190 | SLPI | **1244** | HLA-DPA1 | **2298** | TNFRSF11B | **3352** | PARK7 |
| 191 | OAS1 | **1245** | MARCKS | **2299** | FMN2 | **3353** | HCFC1 |
| 192 | PNP | **1246** | PROM1 | **2300** | UCP2 | **3354** | COPS5 |
| 193 | FGF2 | **1247** | CD28 | **2301** | TRIB1 | **3355** | TNFSF9 |
| 194 | HSP90AA1 | **1248** | INS | **2302** | MBP | **3356** | HLA-DMA |
| 195 | PLCG1 | **1249** | CAMP | **2303** | WWTR1 | **3357** | ATG5 |
| 196 | TUBB3 | **1250** | HLA-DQA1 | **2304** | TUBB | **3358** | HLA-C |
| 197 | LYZ | **1251** | PIK3CB | **2305** | DNASE2 | **3359** | IL10RA |
| 198 | RIPK3 | **1252** | TRC-GCA24-1 | **2306** | PAK1 | **3360** | TXNIP |
| 199 | ALOX15B | **1253** | RPGRIP1L | **2307** | ADGRB1 | **3361** | KIF5B |
| 200 | CEBPD | **1254** | DOCK7 | **2308** | DMD | **3362** | BBS2 |
| 201 | IL23R | **1255** | MIRLET7B | **2309** | PLA2G10 | **3363** | C1QBP |
| 202 | IL16 | **1256** | ZEB2 | **2310** | TTC7A | **3364** | JAM3 |
| 203 | CALCA | **1257** | LRP1 | **2311** | ATF3 | **3365** | RORA |
| 204 | PRKDC | **1258** | CDH5 | **2312** | CEACAM5 | **3366** | RETN |
| 205 | CTSC | **1259** | MAPK7 | **2313** | KCTD7 | **3367** | LOX |
| 206 | CCND1 | **1260** | DPYSL2 | **2314** | SIGLEC9 | **3368** | F2RL1 |
| 207 | NR1H4 | **1261** | RAB13 | **2315** | SNORD15A | **3369** | ITGA4 |
| 208 | S100A12 | **1262** | PKM | **2316** | TYR | **3370** | ACTB |
| 209 | ENG | **1263** | SLC30A1 | **2317** | EGF | **3371** | CCL24 |
| 210 | PFN1 | **1264** | IL12RB2 | **2318** | MOS | **3372** | COL4A5 |
| 211 | LIPE | **1265** | NCF1 | **2319** | CCL13 | **3373** | LYVE1 |
| 212 | FYN | **1266** | MYH9 | **2320** | HRG | **3374** | LMNA |
| 213 | RAB7A | **1267** | COL1A1 | **2321** | ETV3 | **3375** | CD248 |
| 214 | CFH | **1268** | APOC1 | **2322** | PDGFB | **3376** | PDPN |
| 215 | PGF | **1269** | BGN | **2323** | ARSB | **3377** | HDAC9 |
| 216 | RNF114 | **1270** | ADORA2A | **2324** | TNC | **3378** | NPHP3 |
| 217 | IRS2 | **1271** | FLNA | **2325** | ATM | **3379** | MMP13 |
| 218 | STXBP3 | **1272** | MET | **2326** | CDK9 | **3380** | NCOR1 |
| 219 | GFI1 | **1273** | LGALS3BP | **2327** | IDS | **3381** | CD82 |
| 220 | TNFSF15 | **1274** | LCAT | **2328** | SFRP5 | **3382** | STAT4 |
| 221 | GREM1 | **1275** | MIR511 | **2329** | FCGR3B | **3383** | HLA-DMB |
| 222 | PRKCQ | **1276** | HBA1 | **2330** | RICTOR | **3384** | ATG16L1 |
| 223 | MALT1 | **1277** | RAB5A | **2331** | LSP1 | **3385** | GAB3 |
| 224 | SOCS2 | **1278** | RELB | **2332** | CTPS1 | **3386** | IGF2BP1 |
| 225 | ITGB4 | **1279** | IL7R | **2333** | TUG1 | **3387** | RHOC |
| 226 | NF2 | **1280** | NR1D1 | **2334** | GPR55 | **3388** | ALKBH5 |
| 227 | PEX5 | **1281** | HP | **2335** | IL1RAPL2 | **3389** | IL36G |
| 228 | CPM | **1282** | MYO5A | **2336** | TSLP | **3390** | MANF |
| 229 | SIRT6 | **1283** | EDN1 | **2337** | ADAM8 | **3391** | GNAS-AS1 |
| 230 | LRRK2 | **1284** | BIRC2 | **2338** | CEACAM3 | **3392** | BAD |
| 231 | CHRNA7 | **1285** | CALR | **2339** | PON2 | **3393** | TAOK1 |
| 232 | MIR34A | **1286** | TRE-TTC3-1 | **2340** | RXRA | **3394** | RUNX1 |
| 233 | LRP5 | **1287** | PMEL | **2341** | SELE | **3395** | CYLD |
| 234 | DLL4 | **1288** | BCAR1 | **2342** | ATP8B1 | **3396** | TNFSF13 |
| 235 | BIRC3 | **1289** | PLTP | **2343** | GTF2H5 | **3397** | CLEC4A |
| 236 | SND1 | **1290** | CST3 | **2344** | BDNF | **3398** | PTGER2 |
| 237 | MAP3K7 | **1291** | MIR494 | **2345** | RIPK2 | **3399** | TRAF3 |
| 238 | PPP1R9B | **1292** | SGCB | **2346** | NR4A1AS | **3400** | MIR24-1 |
| 239 | F11R | **1293** | AIRE | **2347** | CD3G | **3401** | YBX1 |
| 240 | ZNF335 | **1294** | PSEN1 | **2348** | TIMD4 | **3402** | KEAP1 |
| 241 | BRSK1 | **1295** | PPT1 | **2349** | HLA-DQB1 | **3403** | GLA |
| 242 | RTN4 | **1296** | CDKN2A | **2350** | STAR | **3404** | CEBPA |
| 243 | HBA2 | **1297** | WIPF1 | **2351** | PIEZO1 | **3405** | VWF |
| 244 | BCL2A1 | **1298** | VIP | **2352** | NTN1 | **3406** | MADD |
| 245 | TYMP | **1299** | MAP2K4 | **2353** | AKT3 | **3407** | NPHS1 |
| 246 | GAPDH | **1300** | AREG | **2354** | DNM1L | **3408** | DLG4 |
| 247 | KDR | **1301** | TET2 | **2355** | KCNN4 | **3409** | SIPA1L3 |
| 248 | SPARC | **1302** | HSPG2 | **2356** | ECT2 | **3410** | TAFA4 |
| 249 | CTSF | **1303** | NGF | **2357** | MARCKSL1 | **3411** | TXN |
| 250 | MID1 | **1304** | MX1 | **2358** | SEMA4A | **3412** | MUC5B |
| 251 | SENP3 | **1305** | SIGLEC7 | **2359** | FHL2 | **3413** | ACAT1 |
| 252 | RNASE3 | **1306** | IRAK4 | **2360** | MIR145 | **3414** | DLG5 |
| 253 | SLTM | **1307** | PLEKHO2 | **2361** | GPR32 | **3415** | TEAD1 |
| 254 | TIFAB | **1308** | MAP2K2 | **2362** | ADAM10 | **3416** | ATF4 |
| 255 | SLC16A4 | **1309** | STX3 | **2363** | MAP7 | **3417** | PTX3 |
| 256 | FASN | **1310** | AGFG1 | **2364** | PHGDH | **3418** | WASF2 |
| 257 | MCM4 | **1311** | LRBA | **2365** | CXCR5 | **3419** | MIR221 |
| 258 | LIPG | **1312** | BCL6 | **2366** | FSCN1 | **3420** | CBLB |
| 259 | MIR130A | **1313** | ARFGEF1 | **2367** | PTPN22 | **3421** | HPS6 |
| 260 | PAFAH1B1 | **1314** | H2AX | **2368** | GPR183 | **3422** | PTGES |
| 261 | BCR | **1315** | HSPA8 | **2369** | FHL1 | **3423** | HMGCR |
| 262 | PLCB1 | **1316** | MPDZ | **2370** | AARS1 | **3424** | IL31 |
| 263 | BIRC5 | **1317** | NINJ1 | **2371** | NAGLU | **3425** | PDCD1LG2 |
| 264 | KARS1 | **1318** | TNFSF12 | **2372** | CD244 | **3426** | GRK2 |
| 265 | DUSP1 | **1319** | USP7 | **2373** | CD79A | **3427** | TWIST1 |
| 266 | TUBA1B | **1320** | FTH1 | **2374** | ARL8B | **3428** | SPN |
| 267 | POMT1 | **1321** | IL19 | **2375** | TUBA1A | **3429** | TSC2 |
| 268 | MAP3K5 | **1322** | RDX | **2376** | OTOF | **3430** | HNF1A-AS1 |
| 269 | KLF6 | **1323** | PPARGC1A | **2377** | MAPRE1 | **3431** | CNR1 |
| 270 | NOS1 | **1324** | IDUA | **2378** | P2RY2 | **3432** | ARRB2 |
| 271 | PDE4A | **1325** | MMAB | **2379** | MALAT1 | **3433** | AZU1 |
| 272 | NBAS | **1326** | NEAT1 | **2380** | CD207 | **3434** | CRTAM |
| 273 | FTO | **1327** | SLC15A1 | **2381** | CEACAM8 | **3435** | MIR451A |
| 274 | PLP1 | **1328** | SIN3A | **2382** | MIR222 | **3436** | LATS1 |
| 275 | THBD | **1329** | LDHA | **2383** | FZD5 | **3437** | IL22 |
| 276 | SHPK | **1330** | CCL14 | **2384** | ALDH2 | **3438** | HSF1 |
| 277 | YY1 | **1331** | MTCL1 | **2385** | VSIR | **3439** | PTAFR |
| 278 | PADI4 | **1332** | YWHAZ | **2386** | IGF2 | **3440** | ADAM9 |
| 279 | WNT1 | **1333** | CLASP2 | **2387** | SDCCAG8 | **3441** | MIR223 |
| 280 | VCL | **1334** | HDAC8 | **2388** | TMEM67 | **3442** | AEBP1 |
| 281 | FZD4 | **1335** | SFRP1 | **2389** | NOX1 | **3443** | PRMT1 |
| 282 | SLC37A4 | **1336** | TAP1 | **2390** | MYH11 | **3444** | CD3D |
| 283 | HLA-A | **1337** | TSC1 | **2391** | RASSF5 | **3445** | ICAM2 |
| 284 | CYP1A1 | **1338** | NAMPT | **2392** | PLSCR1 | **3446** | ARL13B |
| 285 | NLRP1 | **1339** | WDR1 | **2393** | SLC2A3 | **3447** | NT5E |
| 286 | SMAD7 | **1340** | PRKCH | **2394** | CTHRC1 | **3448** | MMP8 |
| 287 | CLN3 | **1341** | CD200 | **2395** | TEK | **3449** | TERT |
| 288 | DKK1 | **1342** | GJA1 | **2396** | AURKA | **3450** | SEMA7A |
| 289 | STK26 | **1343** | SLC7A11 | **2397** | EEA1 | **3451** | LAMC2 |
| 290 | LRP2 | **1344** | ABCD1 | **2398** | ANGPTL4 | **3452** | PRKAA2 |
| 291 | TMEM114 | **1345** | SKI | **2399** | RBPJ | **3453** | HSD17B4 |
| 292 | DEFB1 | **1346** | IDH2 | **2400** | MAP2K6 | **3454** | NDUFS4 |
| 293 | RAPGEF3 | **1347** | AP1B1 | **2401** | PTPRO | **3455** | DIABLO |
| 294 | ITGA5 | **1348** | NCOR2 | **2402** | TYK2 | **3456** | DDB1 |
| 295 | MIR99B | **1349** | HNRNPA2B1 | **2403** | CDK4 | **3457** | ADAR |
| 296 | CD1B | **1350** | SOD2 | **2404** | HLA-DQB2 | **3458** | TCF4 |
| 297 | GALNS | **1351** | TNFRSF6B | **2405** | TOLLIP | **3459** | ANGPT1 |
| 298 | KCNQ1 | **1352** | ARHGAP24 | **2406** | SCO2 | **3460** | IL2RB |
| 299 | RPN2 | **1353** | ILF3 | **2407** | LITAF | **3461** | G6PD |
| 300 | COL4A4 | **1354** | MCCC2 | **2408** | TAP2 | **3462** | PNPLA2 |
| 301 | CLDN7 | **1355** | TRIP10 | **2409** | CTNNA1 | **3463** | UGCG |
| 302 | VCAN | **1356** | NCL | **2410** | IL31RA | **3464** | HSPB8 |
| 303 | IDH1 | **1357** | XDH | **2411** | CYP3A4 | **3465** | MIR320A |
| 304 | PTPRJ | **1358** | FGF4 | **2412** | FBF1 | **3466** | STX4 |
| 305 | DCN | **1359** | EIF2AK2 | **2413** | SIGLEC15 | **3467** | ZBTB20 |
| 306 | BRCA1 | **1360** | CRKL | **2414** | CYP1B1 | **3468** | SNAP23 |
| 307 | MLKL | **1361** | GLI1 | **2415** | TTR | **3469** | MCAM |
| 308 | DDB2 | **1362** | CPVL | **2416** | MPI | **3470** | EIF4A1 |
| 309 | GNAI3 | **1363** | RAB11FIP2 | **2417** | MLLT3 | **3471** | IQGAP1 |
| 310 | TNFSF4 | **1364** | AHI1 | **2418** | NCAM1 | **3472** | DHCR7 |
| 311 | KDM1A | **1365** | PLCB3 | **2419** | MAT2A | **3473** | ARNT |
| 312 | OXA1L | **1366** | DICER1 | **2420** | BCL2L11 | **3474** | HSPA1A |
| 313 | WEE1 | **1367** | WNT3A | **2421** | MAP2K3 | **3475** | FCGRT |
| 314 | MTDH | **1368** | TGOLN2 | **2422** | SBNO2 | **3476** | FOSL2 |
| 315 | ADORA2B | **1369** | SELP | **2423** | SDC1 | **3477** | IFNA2 |
| 316 | TIGIT | **1370** | DEFB103A | **2424** | SLC29A1 | **3478** | MLC1 |
| 317 | TUBA1C | **1371** | AP1G1 | **2425** | GCA | **3479** | PVT1 |
| 318 | IL10RB | **1372** | MAPKAPK2 | **2426** | ALOX5AP | **3480** | PHB1 |
| 319 | MYH14 | **1373** | WNT7A | **2427** | IGF2BP3 | **3481** | WNT7B |
| 320 | SREBF2 | **1374** | SPTBN1 | **2428** | HNRNPD | **3482** | ISG15 |
| 321 | PRDX1 | **1375** | MKI67 | **2429** | SLC4A7 | **3483** | EGR2 |
| 322 | COL3A1 | **1376** | ACP1 | **2430** | IL21 | **3484** | CLN8 |
| 323 | SMARCA4 | **1377** | GSK3A | **2431** | ADGRE5 | **3485** | ADIPOR2 |
| 324 | FGF7 | **1378** | UMOD | **2432** | EIF2AK4 | **3486** | PRKAB1 |
| 325 | MIR27A | **1379** | YWHAE | **2433** | DBT | **3487** | RPS3 |
| 326 | TNFRSF9 | **1380** | METTL14 | **2434** | DNMT3A | **3488** | TRIM62 |
| 327 | FOXM1 | **1381** | TRIM21 | **2435** | YY1AP1 | **3489** | FANCI |
| 328 | HDAC5 | **1382** | CAPNS1 | **2436** | FAT4 | **3490** | PAK4 |
| 329 | GLP1R | **1383** | FYCO1 | **2437** | ITGAD | **3491** | SLC25A13 |
| 330 | DNASE1L3 | **1384** | DCSTAMP | **2438** | L1CAM | **3492** | MDK |
| 331 | TIMP2 | **1385** | FURIN | **2439** | CDKN1A | **3493** | HDAC3 |
| 332 | DHX9 | **1386** | MEF2D | **2440** | RAB8B | **3494** | SERPINB2 |
| 333 | ADRB2 | **1387** | NFAT5 | **2441** | NOTCH3 | **3495** | KMT2A |
| 334 | PCCA | **1388** | PDIA3 | **2442** | VTN | **3496** | RPS6KA1 |
| 335 | TAFA3 | **1389** | IKZF2 | **2443** | INPP5E | **3497** | HSPB1 |
| 336 | ELP1 | **1390** | FAT1 | **2444** | ADAMDEC1 | **3498** | PPIF |
| 337 | FLNB | **1391** | AR | **2445** | HADHA | **3499** | ADIPOR1 |
| 338 | KAT2B | **1392** | TRIM65 | **2446** | BCL3 | **3500** | AGTR1 |
| 339 | RAB10 | **1393** | CACNA1C | **2447** | RAB5C | **3501** | SLC26A4 |
| 340 | GLI3 | **1394** | ABCG5 | **2448** | PLOD1 | **3502** | CFLAR |
| 341 | M6PR | **1395** | SELPLG | **2449** | COL7A1 | **3503** | PRKN |
| 342 | SH3BP2 | **1396** | CYTH1 | **2450** | DOCK2 | **3504** | EPHB2 |
| 343 | GLUL | **1397** | COL5A1 | **2451** | LCP1 | **3505** | PADI2 |
| 344 | CASP7 | **1398** | HSP90B1 | **2452** | LGMN | **3506** | STAP1 |
| 345 | SERPINF1 | **1399** | PML | **2453** | ADCY10 | **3507** | HLA-B |
| 346 | SLC4A1 | **1400** | ILK | **2454** | ST14 | **3508** | CDH2 |
| 347 | CD22 | **1401** | ADGRE2 | **2455** | ZBP1 | **3509** | TAT |
| 348 | HTRA2 | **1402** | TNIP1 | **2456** | HDAC11 | **3510** | HHLA2 |
| 349 | WNT4 | **1403** | MMACHC | **2457** | BLM | **3511** | IRF9 |
| 350 | VASP | **1404** | NLRP7 | **2458** | CPT1A | **3512** | LZTR1 |
| 351 | GPC3 | **1405** | PIGR | **2459** | KPNA2 | **3513** | IL17F |
| 352 | MFN2 | **1406** | CD163L1 | **2460** | DDR1 | **3514** | SPIRE1 |
| 353 | NEDD4L | **1407** | NUP85 | **2461** | LOXL2 | **3515** | MT-TP |
| 354 | TNPO1 | **1408** | ODC1 | **2462** | ADA2 | **3516** | NR4A3 |
| 355 | F9 | **1409** | PTBP1 | **2463** | MLANA | **3517** | EXOC4 |
| 356 | KPNA3 | **1410** | XCL1 | **2464** | DNMT1 | **3518** | HDLBP |
| 357 | COX5A | **1411** | ARPC1B | **2465** | PCCB | **3519** | PRKACA |
| 358 | MYO6 | **1412** | MIR379 | **2466** | THOC5 | **3520** | MIR214 |
| 359 | HSPA12A | **1413** | SPINT1 | **2467** | RGS12 | **3521** | TP63 |
| 360 | FBN1 | **1414** | TMEM106A | **2468** | LTB4R | **3522** | BRIP1 |
| 361 | GBP2 | **1415** | PLCB4 | **2469** | SLC26A2 | **3523** | LDAH |
| 362 | MRTFA | **1416** | NEB | **2470** | MIR619 | **3524** | MIR3135B |
| 363 | TKT | **1417** | ACADVL | **2471** | NLRP12 | **3525** | ADCYAP1 |
| 364 | CIRBP | **1418** | EFEMP2 | **2472** | NOTCH2 | **3526** | SNX27 |
| 365 | CPEB4 | **1419** | PANK4 | **2473** | TRAF2 | **3527** | MYLK |
| 366 | PON3 | **1420** | PRNP | **2474** | MME | **3528** | TNFRSF8 |
| 367 | SLC7A1 | **1421** | SLC5A1 | **2475** | TUBG1 | **3529** | YWHAQ |
| 368 | SLC39A14 | **1422** | IL18RAP | **2476** | F13A1 | **3530** | AQP4 |
| 369 | MKS1 | **1423** | F2 | **2477** | CD48 | **3531** | IFNAR1 |
| 370 | SLC38A3 | **1424** | FZD2 | **2478** | EBAG9 | **3532** | RPS6KB1 |
| 371 | SAA1 | **1425** | CRK | **2479** | NEDD9 | **3533** | TNFRSF18 |
| 372 | DAG1 | **1426** | ITLN1 | **2480** | PLK3 | **3534** | MTTP |
| 373 | ITGA6 | **1427** | FGFR2 | **2481** | ISG20 | **3535** | ASL |
| 374 | TMSB4X | **1428** | ENO1 | **2482** | CCDC88C | **3536** | HNRNPA1 |
| 375 | TOR1A | **1429** | HPSE | **2483** | LGALS8 | **3537** | ADAMTSL1 |
| 376 | ICOS | **1430** | C5AR2 | **2484** | TXNRD1 | **3538** | MUC4 |
| 377 | MAN2B1 | **1431** | CAMK2A | **2485** | EXOC3 | **3539** | CEP290 |
| 378 | PPP2CA | **1432** | WNT11 | **2486** | CTCF | **3540** | HCG18 |
| 379 | BMPR2 | **1433** | PIK3R2 | **2487** | PEBP1 | **3541** | ELMO1 |
| 380 | GNAI1 | **1434** | CALCR | **2488** | PDHX | **3542** | PODXL |
| 381 | CUL4A | **1435** | MIR210 | **2489** | EIF4E | **3543** | RUFY3 |
| 382 | SCN5A | **1436** | LIG4 | **2490** | TRPA1 | **3544** | DEFB103B |
| 383 | CSTB | **1437** | NPHS2 | **2491** | ABCC8 | **3545** | ABR |
| 384 | AMOTL1 | **1438** | NR4A2 | **2492** | CLEC6A | **3546** | DLK1 |
| 385 | CD34 | **1439** | TPI1 | **2493** | TRIM22 | **3547** | OPTN |
| 386 | NUDC | **1440** | PFKFB3 | **2494** | SIGIRR | **3548** | LRPPRC |
| 387 | TRPV1 | **1441** | HLA-DQA2 | **2495** | TRP-AGG2-5 | **3549** | ADAMTS4 |
| 388 | PTGER3 | **1442** | SLC46A2 | **2496** | KIF26B | **3550** | GIT1 |
| 389 | PPIA | **1443** | STK24 | **2497** | DYNC2H1 | **3551** | TACR1 |
| 390 | CD2 | **1444** | CYP2J2 | **2498** | RGCC | **3552** | GZMA |
| 391 | SNHG1 | **1445** | SLC17A5 | **2499** | ASPH | **3553** | ERVW-1 |
| 392 | TP53BP2 | **1446** | FERMT3 | **2500** | CYP21A2 | **3554** | SLC22A4 |
| 393 | PHB2 | **1447** | CKAP5 | **2501** | SPON2 | **3555** | CLDN1 |
| 394 | RAB20 | **1448** | FLII | **2502** | ASS1 | **3556** | HLA-DRB3 |
| 395 | FCER1G | **1449** | P2RX4 | **2503** | SETD2 | **3557** | NEDD8 |
| 396 | CDH23 | **1450** | RELN | **2504** | WNT2 | **3558** | HMGA2 |
| 397 | NCOA4 | **1451** | YWHAG | **2505** | ITM2B | **3559** | MUC2 |
| 398 | SOAT2 | **1452** | PROS1 | **2506** | GBP1 | **3560** | ARHGAP31 |
| 399 | CYP19A1 | **1453** | GATA1 | **2507** | IL17RA | **3561** | MIR199B |
| 400 | PSTPIP1 | **1454** | TYRO3 | **2508** | LIPC | **3562** | USF1 |
| 401 | TGIF1 | **1455** | CYP24A1 | **2509** | IL26 | **3563** | MCPH1 |
| 402 | DIAPH1 | **1456** | TFR2 | **2510** | REL | **3564** | SOX2 |
| 403 | ZFP36L1 | **1457** | AMOTL2 | **2511** | FMR1 | **3565** | SEPTIN2 |
| 404 | VIM-AS1 | **1458** | RORC | **2512** | HOXB8 | **3566** | CD24 |
| 405 | CTNND1 | **1459** | MARK3 | **2513** | MAP1LC3B | **3567** | EPHA3 |
| 406 | PLEC | **1460** | CDX2 | **2514** | TUBB2A | **3568** | DCTN2 |
| 407 | SIRT3 | **1461** | GSR | **2515** | S1PR2 | **3569** | IGF1R |
| 408 | ANK3 | **1462** | ACKR3 | **2516** | SDCBP | **3570** | ALDH3A2 |
| 409 | ARHGEF11 | **1463** | BAK1 | **2517** | MIR1207 | **3571** | CHIA |
| 410 | CEL | **1464** | NUMB | **2518** | CA9 | **3572** | NTRK1 |
| 411 | POMT2 | **1465** | XPC | **2519** | UBC | **3573** | RRAS |
| 412 | EBP | **1466** | XCR1 | **2520** | CD58 | **3574** | GAA |
| 413 | EEF1A1 | **1467** | YTHDF2 | **2521** | HMGCL | **3575** | HMGA1 |
| 414 | KIF3A | **1468** | TUBA3C | **2522** | EPOR | **3576** | NDUFA13 |
| 415 | F10 | **1469** | CALM1 | **2523** | RHO | **3577** | SLC16A1 |
| 416 | PDHB | **1470** | AQP1 | **2524** | RS1 | **3578** | POLR2A |
| 417 | CXCL6 | **1471** | NIFK-AS1 | **2525** | SENP1 | **3579** | PEX6 |
| 418 | APOC2 | **1472** | AP1M1 | **2526** | P2RY12 | **3580** | PPP1CB |
| 419 | MEF2A | **1473** | FZD7 | **2527** | VAV2 | **3581** | ERBB3 |
| 420 | IRF2BP2 | **1474** | CD19 | **2528** | NOTCH4 | **3582** | MYH10 |
| 421 | TET1 | **1475** | TUBA4A | **2529** | OSBPL8 | **3583** | BMAL1 |
| 422 | DDX20 | **1476** | TTC8 | **2530** | ABCA2 | **3584** | HSD11B1 |
| 423 | DNAH8 | **1477** | KCNH2 | **2531** | BBS1 | **3585** | CCR5AS |
| 424 | IFNA21 | **1478** | SNHG16 | **2532** | MAVS | **3586** | ABL2 |
| 425 | ULK1 | **1479** | PKD1 | **2533** | ADGRE3 | **3587** | FNDC5 |
| 426 | GNAS | **1480** | SDC2 | **2534** | SH2B3 | **3588** | HS2ST1 |
| 427 | PRDM1 | **1481** | ARL4C | **2535** | CUX1 | **3589** | CSPG4 |
| 428 | SMN1 | **1482** | PPARGC1B | **2536** | CSNK2A1 | **3590** | PLEKHO1 |
| 429 | PLK1 | **1483** | F2R | **2537** | TRIM25 | **3591** | IL36RN |
| 430 | DST | **1484** | PRDX2 | **2538** | MAPKAP1 | **3592** | SLAMF7 |
| 431 | KLK2 | **1485** | PRICKLE4 | **2539** | UHRF1 | **3593** | LCP2 |
| 432 | EIF2AK3 | **1486** | XRCC6 | **2540** | MKKS | **3594** | B9D2 |
| 433 | HSP90AB1 | **1487** | MYO1D | **2541** | TP73 | **3595** | MIR373 |
| 434 | SLC5A6 | **1488** | SRSF2 | **2542** | TJP2 | **3596** | LOC112533672 |
| 435 | FTL | **1489** | CDK2 | **2543** | SH2D1B | **3597** | BGLAP |
| 436 | NOS3 | **1490** | ARRB1 | **2544** | ADM | **3598** | MCCC1 |
| 437 | MIR33B | **1491** | AMH | **2545** | RALA | **3599** | TPX2 |
| 438 | ST6GAL1 | **1492** | MIR29A | **2546** | LAMA3 | **3600** | BID |
| 439 | SEC24B | **1493** | SRGN | **2547** | MGLL | **3601** | ALMS1 |
| 440 | TLN1 | **1494** | EREG | **2548** | DAPK1 | **3602** | HVCN1 |
| 441 | RASA1 | **1495** | SCN8A | **2549** | GAS6 | **3603** | MFSD6 |
| 442 | CTTN | **1496** | ESR2 | **2550** | BMP7 | **3604** | SLC23A2 |
| 443 | SDC4 | **1497** | TCF7L2 | **2551** | TAB2 | **3605** | SLC1A3 |
| 444 | PKN2 | **1498** | KIF14 | **2552** | MAP3K1 | **3606** | UNC13B |
| 445 | LMNB1 | **1499** | JUNB | **2553** | NCR1 | **3607** | ABI1 |
| 446 | HLA-DOB | **1500** | RPS7 | **2554** | HDAC4 | **3608** | AARS2 |
| 447 | STIM1 | **1501** | SLC9A3 | **2555** | WT1 | **3609** | BPI |
| 448 | WASF1 | **1502** | KIF2C | **2556** | ARAF | **3610** | ATP7A |
| 449 | AGA | **1503** | RO60 | **2557** | IFI16 | **3611** | FBXW7 |
| 450 | MIR382 | **1504** | PSIP1 | **2558** | LBR | **3612** | ATP6V1B2 |
| 451 | POR | **1505** | NR2C2 | **2559** | MYB | **3613** | FOXE3 |
| 452 | KCNMA1 | **1506** | CLEC4M | **2560** | AGT | **3614** | CD151 |
| 453 | MIR142 | **1507** | SART1 | **2561** | DNM2 | **3615** | MAP2K7 |
| 454 | KRT18 | **1508** | PIK3C3 | **2562** | PGK1 | **3616** | GLS |
| 455 | SCD | **1509** | IGFBP2 | **2563** | OSBPL3 | **3617** | GPR132 |
| 456 | NGFR | **1510** | ALPL | **2564** | HEXIM1 | **3618** | CWC27 |
| 457 | ADORA3 | **1511** | AIFM1 | **2565** | TAB1 | **3619** | DACT1 |
| 458 | PRL | **1512** | MIR33A | **2566** | LAMP3 | **3620** | PLCB2 |
| 459 | TAX1BP1 | **1513** | IGF2R | **2567** | EPSTI1 | **3621** | COL5A2 |
| 460 | MED12 | **1514** | INHBA | **2568** | G3BP1 | **3622** | PEX19 |
| 461 | GTF2H2 | **1515** | SYT7 | **2569** | BBS9 | **3623** | HEXA |
| 462 | HAVCR1 | **1516** | ABCC4 | **2570** | PLPP3 | **3624** | ANK2 |
| 463 | ATP8A1 | **1517** | TAPBP | **2571** | CD55 | **3625** | GRIP1 |
| 464 | NFKB2 | **1518** | KLRD1 | **2572** | HDAC7 | **3626** | SACS |
| 465 | HLA-E | **1519** | TERF2IP | **2573** | HLA-DRB5 | **3627** | SEMA3A |
| 466 | FSTL1 | **1520** | MMP28 | **2574** | USP14 | **3628** | NBN |
| 467 | GPR84 | **1521** | FIRRE | **2575** | HYOU1 | **3629** | GFAP |
| 468 | FKRP | **1522** | PTPN3 | **2576** | PSMC6 | **3630** | MTHFD1 |
| 469 | SESN2 | **1523** | SKAP2 | **2577** | ACTN1 | **3631** | PLIN1 |
| 470 | NDUFS7 | **1524** | TFAP2A | **2578** | SLC2A5 | **3632** | SLC15A2 |
| 471 | TLR10 | **1525** | GPT | **2579** | TMEM119 | **3633** | TRPM2 |
| 472 | NPPA | **1526** | USP9X | **2580** | MIR122 | **3634** | PLG |
| 473 | HEXB | **1527** | SIX1 | **2581** | ZDHHC7 | **3635** | MIR30A |
| 474 | RNF213 | **1528** | CBX3 | **2582** | FMN1 | **3636** | PABPC1 |
| 475 | S1PR1 | **1529** | EGLN1 | **2583** | LRP6 | **3637** | SYMPK |
| 476 | ZFP36L2 | **1530** | MECP2 | **2584** | SOCS6 | **3638** | EIF4EBP1 |
| 477 | UCHL1 | **1531** | EEF1D | **2585** | KIR3DL1 | **3639** | EWSR1 |
| 478 | HNRNPDL | **1532** | PMM2 | **2586** | NRIP1 | **3640** | TUBA3D |
| 479 | KLF2 | **1533** | FUS | **2587** | FPR3 | **3641** | POU1F1 |
| 480 | ERBIN | **1534** | NQO1 | **2588** | ITGA3 | **3642** | PDHA1 |
| 481 | CAPN2 | **1535** | RMC1 | **2589** | PIP5K1C | **3643** | STRIP1 |
| 482 | PRPF8 | **1536** | NDUFS6 | **2590** | ACADM | **3644** | AP2B1 |
| 483 | FMNL2 | **1537** | CSNK2B | **2591** | GNLY | **3645** | MIR216A |
| 484 | AGPS | **1538** | DNASE1L1 | **2592** | LMNB2 | **3646** | NTRK2 |
| 485 | MIR34C | **1539** | MEF2C | **2593** | NEDD4 | **3647** | RP2 |
| 486 | AHSG | **1540** | POLG | **2594** | IFI30 | **3648** | YWHAB |
| 487 | NES | **1541** | AGO2 | **2595** | ICOSLG | **3649** | BBS12 |
| 488 | ORAI1 | **1542** | SERPINE2 | **2596** | APBA3 | **3650** | KPNB1 |
| 489 | PTPA | **1543** | ENTPD1 | **2597** | LAIR1 | **3651** | PDLIM2 |
| 490 | OTC | **1544** | FFAR4 | **2598** | NRG1 | **3652** | DCLRE1C |
| 491 | SF1 | **1545** | EN1 | **2599** | PIM1 | **3653** | STAB2 |
| 492 | FANCE | **1546** | MGAT5 | **2600** | SLC4A2 | **3654** | ORM1 |
| 493 | SEMA4D | **1547** | VAMP7 | **2601** | PDE2A | **3655** | P2RY1 |
| 494 | CLMP | **1548** | CP | **2602** | CALM2 | **3656** | EPB41L5 |
| 495 | IL17D | **1549** | USP18 | **2603** | PLXNB2 | **3657** | HIF1AN |
| 496 | PDP1 | **1550** | ALOX12 | **2604** | ARHGEF1 | **3658** | DOT1L |
| 497 | TNFAIP2 | **1551** | CTDSP2 | **2605** | CASK | **3659** | F8 |
| 498 | BEST1 | **1552** | PRRC2B | **2606** | RAB14 | **3660** | RHOB |
| 499 | SNX10 | **1553** | RAC3 | **2607** | KRT5 | **3661** | MITF |
| 500 | KAT5 | **1554** | STIL | **2608** | SLC9D1 | **3662** | MIR125B1 |
| 501 | MYO1C | **1555** | OTUD5 | **2609** | PGAM5 | **3663** | EPCAM |
| 502 | MIR126 | **1556** | CDK1 | **2610** | GLRX | **3664** | CELSR1 |
| 503 | TMOD3 | **1557** | ATG7 | **2611** | ACSL1 | **3665** | APPL1 |
| 504 | C5 | **1558** | MACF1 | **2612** | FLOT1 | **3666** | ALK |
| 505 | SPHK1 | **1559** | DSPP | **2613** | CTSH | **3667** | AZGP1 |
| 506 | PDGFA | **1560** | LAG3 | **2614** | NRP1 | **3668** | E2F1 |
| 507 | MYL6 | **1561** | ATF2 | **2615** | KPNA1 | **3669** | ANGPT2 |
| 508 | BACE1 | **1562** | AP2A1 | **2616** | WNT2B | **3670** | CD2AP |
| 509 | ACLY | **1563** | ATP1A1 | **2617** | SFPQ | **3671** | CEACAM6 |
| 510 | MRAS | **1564** | WASL | **2618** | SPRY2 | **3672** | OCSTAMP |
| 511 | PPM1A | **1565** | SOS1 | **2619** | SERPINB1 | **3673** | GLDC |
| 512 | JUND | **1566** | PDCD6IP | **2620** | BCKDHA | **3674** | TG |
| 513 | MGMT | **1567** | ACKR2 | **2621** | TOMM20 | **3675** | SLC1A2 |
| 514 | MYH2 | **1568** | HTT | **2622** | LPA | **3676** | SLC2A4 |
| 515 | TAC1 | **1569** | FLOT2 | **2623** | HSP90AA2P | **3677** | MAP1B |
| 516 | CAPN1 | **1570** | LINC02620 | **2624** | PLAT | **3678** | PLEK |
| 517 | RECK | **1571** | IFN1@ | **2625** | MIAT | **3679** | PEX1 |
| 518 | AKT1S1 | **1572** | DAXX | **2626** | PTGDS | **3680** | S100A11 |
| 519 | ANK1 | **1573** | SPTLC1 | **2627** | EXOC2 | **3681** | LAPTM5 |
| 520 | JMJD1C | **1574** | KIF4A | **2628** | HNRNPM | **3682** | ACTN4 |
| 521 | TRPM7 | **1575** | CLU | **2629** | HNRNPU | **3683** | SAV1 |
| 522 | ARID1A | **1576** | CLTC | **2630** | HSPA6 | **3684** | CALM3 |
| 523 | ADPRH | **1577** | LPCAT3 | **2631** | DUSP3 | **3685** | PGM3 |
| 524 | SNORD44 | **1578** | LAMB3 | **2632** | DSG2 | **3686** | LAMA1 |
| 525 | SLC19A3 | **1579** | NRP2 | **2633** | RUVBL1 | **3687** | CHGA |
| 526 | RPS6 | **1580** | RNASET2 | **2634** | RPLP2 | **3688** | NFATC2 |
| 527 | SLC22A2 | **1581** | SYNE4 | **2635** | RPS6KA5 | **3689** | SOD3 |
| 528 | EIF5A | **1582** | GFER | **2636** | NCK1 | **3690** | MIR9-2HG |
| 529 | HPRT1 | **1583** | ILF2 | **2637** | P4HB | **3691** | UBE2K |
| 530 | G6PC1 | **1584** | TEAD4 | **2638** | USH1G | **3692** | AURKB |
| 531 | TNFRSF12A | **1585** | IFT140 | **2639** | EDNRB | **3693** | SF3B2 |
| 532 | TNFSF18 | **1586** | DRD2 | **2640** | ELF4 | **3694** | TNFRSF14 |
| 533 | GPX1 | **1587** | IHH | **2641** | HDC | **3695** | IVD |
| 534 | ALCAM | **1588** | DYNLL1 | **2642** | XPA | **3696** | NEK7 |
| 535 | IL18BP | **1589** | FANCD2 | **2643** | SIGLEC10 | **3697** | ID3 |
| 536 | TRP-AGG2-6 | **1590** | WHRN | **2644** | FUCA1 | **3698** | DGAT1 |
| 537 | ADAM12 | **1591** | FOSL1 | **2645** | MS4A4A | **3699** | SPINT2 |
| 538 | MFN1 | **1592** | MIR124-1 | **2646** | GATA6 | **3700** | MYO10 |
| 539 | MT-CYB | **1593** | F2RL2 | **2647** | VAPA | **3701** | C4A |
| 540 | PIK3C2A | **1594** | SLC35A1 | **2648** | WWC1 | **3702** | SLC46A1 |
| 541 | PAEP | **1595** | EVC2 | **2649** | FNBP1 | **3703** | CD1E |
| 542 | FGFR3 | **1596** | UNC5B | **2650** | STMN1 | **3704** | ADM2 |
| 543 | SRSF3 | **1597** | IRF2 | **2651** | NONO | **3705** | COL8A1 |
| 544 | HCST | **1598** | P2RY11 | **2652** | APOBEC3F | **3706** | TMEM237 |
| 545 | DLD | **1599** | PAPPA-AS1 | **2653** | RPL11 | **3707** | MDM4 |
| 546 | EDN2 | **1600** | GNB1 | **2654** | P2RX5-TAX1BP3 | **3708** | PLIN3 |
| 547 | NR0B1 | **1601** | LIPF | **2655** | CGA | **3709** | C3AR1 |
| 548 | RLBP1 | **1602** | ARID3A | **2656** | PVR | **3710** | FLT4 |
| 549 | VPS26A | **1603** | ZC3HAV1 | **2657** | MBNL1 | **3711** | OFD1 |
| 550 | USP12 | **1604** | KCNA3 | **2658** | GNAI2 | **3712** | SMPD3 |
| 551 | CAMK2G | **1605** | ATP2A2 | **2659** | MDH2 | **3713** | N4BP1 |
| 552 | RBP4 | **1606** | IL13RA1 | **2660** | COL4A1 | **3714** | FLI1 |
| 553 | RPL29 | **1607** | SGK1 | **2661** | RANBP2 | **3715** | CYSLTR2 |
| 554 | NNMT | **1608** | TNFRSF13B | **2662** | DAAM2 | **3716** | ARHGEF7 |
| 555 | PEX11B | **1609** | SRF | **2663** | PARVA | **3717** | MSH6 |
| 556 | WDR5 | **1610** | DAGLB | **2664** | REG3A | **3718** | SST |
| 557 | FMNL3 | **1611** | ELK4 | **2665** | RAG1 | **3719** | C1S |
| 558 | CCL16 | **1612** | CLIC1 | **2666** | KPNA4 | **3720** | KRT17 |
| 559 | FABP7 | **1613** | RTP3 | **2667** | SLC7A5 | **3721** | GRK3 |
| 560 | CPS1 | **1614** | RPS27A | **2668** | RFX5 | **3722** | PRC1 |
| 561 | CRTC3 | **1615** | KLRB1 | **2669** | FREM2 | **3723** | CCDC85C |
| 562 | FYB1 | **1616** | SEPTIN9 | **2670** | GCLM | **3724** | CDK5 |
| 563 | PLEKHG5 | **1617** | PTPN13 | **2671** | AP1M2 | **3725** | KIAA0586 |
| 564 | GNB2 | **1618** | DCLK1 | **2672** | TRIM24 | **3726** | ADCY1 |
| 565 | TSPAN32 | **1619** | ATXN2 | **2673** | C1QA | **3727** | CEBPE |
| 566 | RPL18A | **1620** | S100A7 | **2674** | LUM | **3728** | POLD1 |
| 567 | FERMT1 | **1621** | MUC5AC | **2675** | AQP3 | **3729** | VDAC1 |
| 568 | SERPINC1 | **1622** | CEP131 | **2676** | CD300LF | **3730** | SLC6A8 |
| 569 | ANGPTL2 | **1623** | MIR193A | **2677** | RET | **3731** | DUSP6 |
| 570 | DLG2 | **1624** | DNM3 | **2678** | NR1I2 | **3732** | E2F4 |
| 571 | ARHGDIA | **1625** | FIP1L1 | **2679** | GALK1 | **3733** | TUBB6 |
| 572 | ERC1 | **1626** | JUP | **2680** | RPS3A | **3734** | USO1 |
| 573 | PPM1F | **1627** | RAB35 | **2681** | CMTM6 | **3735** | SULT2B1 |
| 574 | FOLR1 | **1628** | VIPR1 | **2682** | TGFBI | **3736** | GMNN |
| 575 | PITRM1 | **1629** | FLG | **2683** | UBA1 | **3737** | ST3GAL4 |
| 576 | FANCL | **1630** | SLC12A6 | **2684** | MYO1F | **3738** | CHD4 |
| 577 | TBC1D1 | **1631** | POLH | **2685** | UBA52 | **3739** | TNFRSF10A |
| 578 | RPL18 | **1632** | CEP55 | **2686** | DUOX2 | **3740** | MOB1A |
| 579 | ASGR1 | **1633** | CFL1 | **2687** | FOLH1 | **3741** | CASP2 |
| 580 | MIR486-1 | **1634** | TRPV2 | **2688** | PKP4 | **3742** | FGF1 |
| 581 | ARHGAP17 | **1635** | PRKAG1 | **2689** | GLI2 | **3743** | MIR375 |
| 582 | CYSLTR1 | **1636** | YWHAH | **2690** | CDK5RAP3 | **3744** | RAB39A |
| 583 | ALG1 | **1637** | NDUFV1 | **2691** | PEX16 | **3745** | PEX13 |
| 584 | SYVN1 | **1638** | KHDRBS1 | **2692** | RPS19 | **3746** | CNTF |
| 585 | GDNF | **1639** | SCNN1B | **2693** | CAP1 | **3747** | ABCC11 |
| 586 | S100A1 | **1640** | PRKD3 | **2694** | RPL7A | **3748** | MIR133B |
| 587 | AQP5 | **1641** | USP4 | **2695** | ABCC5 | **3749** | CYP17A1 |
| 588 | ENO2 | **1642** | ERBB4 | **2696** | YBX3 | **3750** | GPR137B |
| 589 | CAPZA1 | **1643** | HYLS1 | **2697** | DEF6 | **3751** | VHL |
| 590 | SDHA | **1644** | SAP30BP | **2698** | IMPDH2 | **3752** | ANXA6 |
| 591 | GCDH | **1645** | LEPR | **2699** | VIPR2 | **3753** | STK38 |
| 592 | OXER1 | **1646** | DNAJB1 | **2700** | RBM15 | **3754** | ADAMTS1 |
| 593 | LHFPL2 | **1647** | BYSL | **2701** | IL13RA2 | **3755** | IL1R2 |
| 594 | RPGR | **1648** | TRIB3 | **2702** | RPS18 | **3756** | GAB2 |
| 595 | HGS | **1649** | HTRA1 | **2703** | RNASEL | **3757** | GPER1 |
| 596 | CYP3A5 | **1650** | DDX39A | **2704** | PHLPP1 | **3758** | TMPO |
| 597 | RAB22A | **1651** | MPZ | **2705** | APOH | **3759** | AKAP8 |
| 598 | GPR65 | **1652** | CISH | **2706** | MIR181A1 | **3760** | MIR17 |
| 599 | DPYD | **1653** | APOBEC3A | **2707** | NTRK3 | **3761** | MCTS1 |
| 600 | FGF23 | **1654** | RPS15 | **2708** | SP3 | **3762** | SLC2A6 |
| 601 | PIK3R4 | **1655** | SATB1 | **2709** | ENAH | **3763** | CRMA |
| 602 | G3BP2 | **1656** | APOA2 | **2710** | SNHG7 | **3764** | LPCAT2 |
| 603 | MOG | **1657** | POLR3A | **2711** | CCN1 | **3765** | SYNC |
| 604 | LY9 | **1658** | CSN1S1 | **2712** | YES1 | **3766** | VAMP3 |
| 605 | RPL6 | **1659** | CFB | **2713** | PSMA6 | **3767** | CCN4 |
| 606 | MYCN | **1660** | WDR83 | **2714** | MATR3 | **3768** | IFIT3 |
| 607 | TRIM28 | **1661** | SIRT2 | **2715** | TFE3 | **3769** | APCS |
| 608 | EIF2S1 | **1662** | GIT2 | **2716** | CYRIB | **3770** | MAP4K4 |
| 609 | FGD5-AS1 | **1663** | ATP11C | **2717** | GGT1 | **3771** | SERPINA3 |
| 610 | PKP2 | **1664** | MLEC | **2718** | IL1F10 | **3772** | HK1 |
| 611 | HNRNPH1 | **1665** | PDLIM1 | **2719** | VPS51 | **3773** | APEX1 |
| 612 | DUOX1 | **1666** | DLG3 | **2720** | MIR150 | **3774** | HGFAC |
| 613 | PLXNA3 | **1667** | ERAP1 | **2721** | CYP11A1 | **3775** | KPNA5 |
| 614 | MIR128-1 | **1668** | OTULIN | **2722** | ENHO | **3776** | DLEU1 |
| 615 | MIR125B2 | **1669** | FHOD1 | **2723** | CCDC88A | **3777** | CDKN1B |
| 616 | RBL2 | **1670** | KIF20A | **2724** | APLNR | **3778** | AKR1B1 |
| 617 | CCNT2 | **1671** | HNRNPK | **2725** | NECTIN1 | **3779** | GPR15 |
| 618 | PLD1 | **1672** | CHEK2 | **2726** | HEPH | **3780** | MYO9A |
| 619 | TNS3 | **1673** | TNFRSF4 | **2727** | TMEM201 | **3781** | FMO3 |
| 620 | PCDH15 | **1674** | TUFM | **2728** | NEU3 | **3782** | TNS1 |
| 621 | MANBA | **1675** | NR5A2 | **2729** | ADNP | **3783** | VAC14 |
| 622 | ANLN | **1676** | KL | **2730** | RUVBL2 | **3784** | RPSA |
| 623 | DDX5 | **1677** | ADAD2-AS1 | **2731** | SRRT | **3785** | PF4V1 |
| 624 | RAB21 | **1678** | CPQ | **2732** | DPEP1 | **3786** | DAPK2 |
| 625 | SRSF1 | **1679** | GNA14 | **2733** | IL24 | **3787** | APOC3 |
| 626 | ANO6 | **1680** | EPRS1 | **2734** | PTS | **3788** | TXLNA |
| 627 | ATP5F1A | **1681** | RNF31 | **2735** | MEG3 | **3789** | RFTN1 |
| 628 | GNA13 | **1682** | WNK1 | **2736** | KRT8 | **3790** | RTEL1 |
| 629 | RPL13 | **1683** | CPOX | **2737** | PTPRZ1 | **3791** | TBCD |
| 630 | PRPF3 | **1684** | COPG1 | **2738** | GRAP2 | **3792** | LTB |
| 631 | MIR140 | **1685** | ITGB7 | **2739** | FERMT2 | **3793** | ENPP1 |
| 632 | IL27RA | **1686** | PLA2G4C | **2740** | CEACAM1 | **3794** | PLEKHM2 |
| 633 | CFAP298 | **1687** | CKB | **2741** | FOSB | **3795** | MMUT |
| 634 | SARM1 | **1688** | ANTXR1 | **2742** | FUT4 | **3796** | PRX |
| 635 | XRCC1 | **1689** | CCR9 | **2743** | ATP5F1B | **3797** | SLC23A1 |
| 636 | ECSIT | **1690** | LEF1 | **2744** | SCGB1A1 | **3798** | VAMP8 |
| 637 | CA2 | **1691** | LINC01194 | **2745** | PSMA5 | **3799** | ITGA2 |
| 638 | PFN2 | **1692** | ERCC4 | **2746** | AIMP1 | **3800** | NUP153 |
| 639 | MXD1 | **1693** | IER3 | **2747** | GSTP1 | **3801** | MCUB |
| 640 | CCNB1 | **1694** | APLN | **2748** | KPNA6 | **3802** | ACSL4 |
| 641 | EFHD2 | **1695** | SRP14 | **2749** | MIR143 | **3803** | NFKBIZ |
| 642 | MAP2 | **1696** | POU5F1 | **2750** | PTHLH | **3804** | GRK5 |
| 643 | AP1S1 | **1697** | PTMA | **2751** | PRG2 | **3805** | MIR301B |
| 644 | SLC6A3 | **1698** | SNHG12 | **2752** | BBIP1 | **3806** | PI3 |
| 645 | PSMD2 | **1699** | WWP2 | **2753** | RBM17 | **3807** | CTNS |
| 646 | TCF3 | **1700** | PIK3C2B | **2754** | RRAS2 | **3808** | PDCD10 |
| 647 | LDLRAP1 | **1701** | AFP | **2755** | IAPP | **3809** | UBE2L6 |
| 648 | NLRC3 | **1702** | SLC9A8 | **2756** | CCDC47 | **3810** | CARS1 |
| 649 | CHRFAM7A | **1703** | XBP1 | **2757** | ACTC1 | **3811** | CXADR |
| 650 | TRAF5 | **1704** | TTPA | **2758** | RHOH | **3812** | SRM |
| 651 | RNF128 | **1705** | PSMD4 | **2759** | PNKD | **3813** | RBSN |
| 652 | MICB | **1706** | UCP1 | **2760** | TTYH3 | **3814** | XRCC5 |
| 653 | ETV5 | **1707** | SIAH1 | **2761** | CLDN11 | **3815** | LATS2 |
| 654 | HOTAIRM1 | **1708** | NME1 | **2762** | FGF5 | **3816** | ATP1B1 |
| 655 | ALDOA | **1709** | ANXA4 | **2763** | MIR30B | **3817** | TNPO3 |
| 656 | ABCG8 | **1710** | CCND3 | **2764** | PROK2 | **3818** | LRG1 |
| 657 | GARS1 | **1711** | CLCA1 | **2765** | SPHK2 | **3819** | BAMBI |
| 658 | GSTO1 | **1712** | HNRNPL | **2766** | EPAS1 | **3820** | PDCD4 |
| 659 | VASH1 | **1713** | FOXC1 | **2767** | SMG6 | **3821** | MIR24-2 |
| 660 | COL11A2 | **1714** | PAH | **2768** | CLRN1 | **3822** | EHBP1L1 |
| 661 | PCNA | **1715** | FRAS1 | **2769** | SWAP70 | **3823** | VLDLR |
| 662 | SPTLC2 | **1716** | MIR200B | **2770** | TOP2A | **3824** | FAU |
| 663 | SYT11 | **1717** | MIR23A | **2771** | SMURF2 | **3825** | BBS10 |
| 664 | SP110 | **1718** | EDIL3 | **2772** | CYP2E1 | **3826** | CSTA |
| 665 | GDI2 | **1719** | MEN1 | **2773** | YTHDF1 | **3827** | FFAR2 |
| 666 | CUL3 | **1720** | AQP2 | **2774** | LINC01140 | **3828** | PRLR |
| 667 | USP15 | **1721** | USH1C | **2775** | PRRC2A | **3829** | MAD1L1 |
| 668 | CYP2A6 | **1722** | RALBP1 | **2776** | CDC42SE1 | **3830** | KLF3 |
| 669 | SLC9A9 | **1723** | ETV6 | **2777** | APOL1 | **3831** | MAP6 |
| 670 | ALYREF | **1724** | ITGB5 | **2778** | IGHG1 | **3832** | SLC39A8 |
| 671 | WARS1 | **1725** | A2M | **2779** | NLRX1 | **3833** | WDR26 |
| 672 | TCOF1 | **1726** | RAN | **2780** | ACACA | **3834** | ALDH3A1 |
| 673 | HNRNPC | **1727** | DCTN1 | **2781** | AGK | **3835** | CPSF2 |
| 674 | DNAJA1 | **1728** | EGLN3 | **2782** | PPP2R1A | **3836** | PRMT5 |
| 675 | MCU | **1729** | ECE1 | **2783** | BBS7 | **3837** | PYGB |
| 676 | SLC1A4 | **1730** | ANKLE2 | **2784** | NRIR | **3838** | OTUB1 |
| 677 | HRH2 | **1731** | CHI3L2 | **2785** | NLRP2 | **3839** | STAU1 |
| 678 | PLXNA1 | **1732** | NODAL | **2786** | ABCB11 | **3840** | MLST8 |
| 679 | ARPC5 | **1733** | DDR2 | **2787** | TRAF3IP2 | **3841** | ERCC5 |
| 680 | RNF113A | **1734** | PTP4A3 | **2788** | KIF13A | **3842** | TNIP2 |
| 681 | CDC42BPA | **1735** | PDE3B | **2789** | GSDME | **3843** | ASH1L |
| 682 | TUBB4A | **1736** | FXR1 | **2790** | HRH4 | **3844** | MIR204 |
| 683 | AFDN | **1737** | TFPI | **2791** | MS4A7 | **3845** | RPL35 |
| 684 | MYH4 | **1738** | IFNL1 | **2792** | MMP10 | **3846** | NOP56 |
| 685 | DYRK1A | **1739** | SUMO2 | **2793** | MIRLET7D | **3847** | OPRK1 |
| 686 | FBXO11 | **1740** | VCP | **2794** | TCIRG1 | **3848** | STK3 |
| 687 | APOA4 | **1741** | SLC1A5 | **2795** | NORAD | **3849** | SMARCB1 |
| 688 | CLCN7 | **1742** | UNC119 | **2796** | TEC | **3850** | WDR82 |
| 689 | ABCA4 | **1743** | RAB1B | **2797** | RPL13A | **3851** | TERF2 |
| 690 | MIR181D | **1744** | MIR9-1 | **2798** | KNG1 | **3852** | PDCD6 |
| 691 | DHX33 | **1745** | MYO9B | **2799** | CYP11B1 | **3853** | FAM161A |
| 692 | H6PD | **1746** | KCNJ2 | **2800** | CD300A | **3854** | RPL28 |
| 693 | RPS11 | **1747** | MIR106A | **2801** | ECM1 | **3855** | TPO |
| 694 | CHEK1 | **1748** | MAPRE3 | **2802** | MARS1 | **3856** | BACH1 |
| 695 | NPPB | **1749** | GINS1 | **2803** | HAND2 | **3857** | MVP |
| 696 | NOP53 | **1750** | ACHE | **2804** | EDC4 | **3858** | MIRLET7A1 |
| 697 | UBE2D2 | **1751** | NF1 | **2805** | HIP1 | **3859** | CELSR2 |
| 698 | NCOA5 | **1752** | TRIM29 | **2806** | BIN1 | **3860** | LY86 |
| 699 | SLC39A10 | **1753** | BPIFA1 | **2807** | RPL26 | **3861** | ARIH2 |
| 700 | CRLF2 | **1754** | DDX3X | **2808** | SLC25A5 | **3862** | TRPC6 |
| 701 | H4C4 | **1755** | MYH7 | **2809** | MAP4 | **3863** | GAB1 |
| 702 | KDM5B | **1756** | RAI14 | **2810** | PDGFRA | **3864** | TRIM33 |
| 703 | PACSIN2 | **1757** | MIR192 | **2811** | IL1RAP | **3865** | TFAM |
| 704 | ANXA11 | **1758** | HPGD | **2812** | SLC19A1 | **3866** | ADGRV1 |
| 705 | VPS13B | **1759** | TMEM216 | **2813** | PPP1CC | **3867** | THRIL |
| 706 | IL21R | **1760** | TBX21 | **2814** | ARHGEF6 | **3868** | CTSE |
| 707 | MTA1 | **1761** | OCA2 | **2815** | GBE1 | **3869** | PLEKHA4 |
| 708 | ADGRF5 | **1762** | CALCOCO2 | **2816** | PRCC | **3870** | AGO1 |
| 709 | HNRNPF | **1763** | CSN2 | **2817** | HMGN2 | **3871** | SLC9A6 |
| 710 | STX1A | **1764** | ARHGAP25 | **2818** | PRKCG | **3872** | PLCD1 |
| 711 | KCNN1 | **1765** | EPHB1 | **2819** | USP10 | **3873** | EIF3B |
| 712 | STRAP | **1766** | SLFN11 | **2820** | LPXN | **3874** | MIR302A |
| 713 | SSB | **1767** | RPA1 | **2821** | PAX5 | **3875** | TK1 |
| 714 | ZC3H7A | **1768** | SLAMF6 | **2822** | ELK1 | **3876** | LIMK1 |
| 715 | VPS33A | **1769** | NCR2 | **2823** | SLC25A19 | **3877** | SRRM2 |
| 716 | FAS-AS1 | **1770** | SETDB1 | **2824** | CKAP4 | **3878** | RRM2 |
| 717 | NUDT21 | **1771** | GRB10 | **2825** | BCAN | **3879** | UBR5 |
| 718 | MIR203A | **1772** | ATRX | **2826** | RAB1A | **3880** | HCAR2 |
| 719 | NBR1 | **1773** | LARRPM | **2827** | RPTOR | **3881** | PHKA2 |
| 720 | WTAP | **1774** | HECTD4 | **2828** | NTF3 | **3882** | ABHD12 |
| 721 | SLC29A2 | **1775** | NCOA3 | **2829** | CYBRD1 | **3883** | FEN1 |
| 722 | NUP214 | **1776** | ARHGAP45 | **2830** | PARVB | **3884** | RAPGEF6 |
| 723 | IL36B | **1777** | PPP1R12A | **2831** | IMMT | **3885** | GABARAP |
| 724 | WDR62 | **1778** | MYO1E | **2832** | RAD51 | **3886** | SLC16A3 |
| 725 | IGFBP3 | **1779** | TLE1 | **2833** | DHX30 | **3887** | SHOC2 |
| 726 | ZHX2 | **1780** | PIM2 | **2834** | COMT | **3888** | DDX17 |
| 727 | SULT1A1 | **1781** | H2AC21 | **2835** | HK2 | **3889** | PPFIA1 |
| 728 | GNAQ | **1782** | RBX1 | **2836** | PRKAR1A | **3890** | MGAM |
| 729 | PINK1 | **1783** | P2RX1 | **2837** | RACGAP1 | **3891** | SLC8A1 |
| 730 | FER | **1784** | XPO5 | **2838** | CFI | **3892** | MIR148B |
| 731 | CACNA2D1 | **1785** | EMD | **2839** | PIKFYVE | **3893** | DIDO1 |
| 732 | HNRNPR | **1786** | PEX14 | **2840** | SMAD5 | **3894** | PAPPA |
| 733 | GOLGA2 | **1787** | ADRM1 | **2841** | TMPRSS2 | **3895** | HSPE1 |
| 734 | MORC2 | **1788** | HYAL1 | **2842** | HES1 | **3896** | IDE |
| 735 | SPAST | **1789** | DHX15 | **2843** | KDM5A | **3897** | ITCH |
| 736 | FABP5 | **1790** | S100P | **2844** | PIN1 | **3898** | HNRNPAB |
| 737 | GOLM1 | **1791** | FAM120A | **2845** | EIF2B4 | **3899** | CANX |
| 738 | RGS1 | **1792** | PSME2 | **2846** | GNPAT | **3900** | H3C1 |
| 739 | SUN2 | **1793** | RAB31 | **2847** | GPI | **3901** | SUMO1 |
| 740 | C1QC | **1794** | KIF2A | **2848** | DLGAP5 | **3902** | SORL1 |
| 741 | ARL11 | **1795** | METAP1 | **2849** | ARCN1 | **3903** | SUV39H1 |
| 742 | BCLAF1 | **1796** | SBNO1 | **2850** | PRKRA | **3904** | PLGRKT |
| 743 | LRPAP1 | **1797** | RBMX | **2851** | IK | **3905** | MIR195 |
| 744 | NOP2 | **1798** | STX7 | **2852** | HRH1 | **3906** | IFI27 |
| 745 | ABCF1 | **1799** | RHOV | **2853** | STAM | **3907** | TRIM72 |
| 746 | SAMD9L | **1800** | LIN28B | **2854** | SPTAN1 | **3908** | GNG5 |
| 747 | FGF9 | **1801** | MAX | **2855** | LMLN | **3909** | PIAS1 |
| 748 | MUC16 | **1802** | ETV4 | **2856** | IFITM3 | **3910** | HOMER3 |
| 749 | PRKAR2A | **1803** | H2AC6 | **2857** | FLNC | **3911** | BNIP3 |
| 750 | ERCC1 | **1804** | CLCN1 | **2858** | TCTN2 | **3912** | HSPA9 |
| 751 | ERG | **1805** | TAFAZZIN | **2859** | SAFB | **3913** | SLC25A1 |
| 752 | EIF4B | **1806** | APLP2 | **2860** | OIT3 | **3914** | ADAM28 |
| 753 | ABCA12 | **1807** | TARDBP | **2861** | PLA2G1B | **3915** | NEURL4 |
| 754 | RAB6A | **1808** | AP2M1 | **2862** | RAD50 | **3916** | SLC27A1 |
| 755 | NCOA2 | **1809** | RTKN | **2863** | NIN | **3917** | GRK6 |
| 756 | KIFC1 | **1810** | IBTK | **2864** | IL25 | **3918** | LINC01094 |
| 757 | POLA1 | **1811** | MX2 | **2865** | PLXNB1 | **3919** | UBXN7 |
| 758 | RPL14 | **1812** | COLEC12 | **2866** | DNER | **3920** | MIR138-2 |
| 759 | WASHC5 | **1813** | DSC1 | **2867** | UBE2I | **3921** | CDCA4 |
| 760 | PDE4DIP | **1814** | NIPBL | **2868** | FLCN | **3922** | BRD9 |
| 761 | CCAR2 | **1815** | INPP4A | **2869** | CEP104 | **3923** | KLF7 |
| 762 | CAVIN1 | **1816** | MSRA | **2870** | NELFE | **3924** | EEF2 |
| 763 | SLC2A9 | **1817** | KIF22 | **2871** | BUD23 | **3925** | DNM1 |
| 764 | SRSF5 | **1818** | INVS | **2872** | LINC00467 | **3926** | TGFBR3 |
| 765 | ITPR3 | **1819** | GPX4 | **2873** | ATP5F1D | **3927** | TPM3 |
| 766 | SLC26A3 | **1820** | PFKL | **2874** | PLSCR3 | **3928** | HUWE1 |
| 767 | CYP2C8 | **1821** | BATF2 | **2875** | AMIGO2 | **3929** | UTRN |
| 768 | RIPPLY2 | **1822** | HLA-DOA | **2876** | MIR215 | **3930** | TIA1 |
| 769 | APOBEC1 | **1823** | SEC23B | **2877** | PRKAG2 | **3931** | IL20 |
| 770 | STC1 | **1824** | TNFRSF25 | **2878** | SCYL1 | **3932** | EBI3 |
| 771 | LACTB | **1825** | PLPP1 | **2879** | BAG3 | **3933** | CPEB1 |
| 772 | RABGAP1L | **1826** | AKR1B10 | **2880** | NUP37 | **3934** | ACIN1 |
| 773 | SMAD1 | **1827** | IL15RA | **2881** | AGXT | **3935** | KAT7 |
| 774 | RPL37A | **1828** | H3-3B | **2882** | PSPC1 | **3936** | BANF1 |
| 775 | SPARCL1 | **1829** | RPLP0 | **2883** | SIK3 | **3937** | RECQL |
| 776 | SF3A1 | **1830** | USP8 | **2884** | SUCLG1 | **3938** | CCT3 |
| 777 | MIR4306 | **1831** | EIF2B1 | **2885** | TNFRSF10B | **3939** | SLCO2B1 |
| 778 | RUBCN | **1832** | RAPGEF2 | **2886** | ICMT | **3940** | LIFR |
| 779 | FKBP1A | **1833** | DNASE1 | **2887** | KIAA0753 | **3941** | OSMR |
| 780 | MAD2L1 | **1834** | H2BC11 | **2888** | UNG | **3942** | GFOD3P |
| 781 | PTGDR2 | **1835** | MAPK12 | **2889** | TRAP1 | **3943** | HKDC1 |
| 782 | PDK1 | **1836** | PLD2 | **2890** | KIF1C | **3944** | MIR1246 |
| 783 | ELL | **1837** | HAUS5 | **2891** | CPSF6 | **3945** | NUP62 |
| 784 | NHERF2 | **1838** | HNRNPA0 | **2892** | MED1 | **3946** | NECTIN2 |
| 785 | ZFYVE27 | **1839** | CNMD | **2893** | NUPR1 | **3947** | MCM3 |
| 786 | DCT | **1840** | ADGRG1 | **2894** | CCT8 | **3948** | GAMT |
| 787 | ALDOB | **1841** | SLC25A20 | **2895** | HSD3B2 | **3949** | PEX3 |
| 788 | FANCB | **1842** | TMT1B | **2896** | PAG1 | **3950** | C9orf72 |
| 789 | KCNB1 | **1843** | SH3GL1 | **2897** | WLS | **3951** | DAZAP1 |
| 790 | DCD | **1844** | NUFIP2 | **2898** | GRP | **3952** | GNA11 |
| 791 | PIP5K1A | **1845** | HCLS1 | **2899** | DOCK1 | **3953** | ARHGEF18 |
| 792 | GTF2H1 | **1846** | RRBP1 | **2900** | DUSP19 | **3954** | WNK4 |
| 793 | XPO1 | **1847** | PLLP | **2901** | HMCN1 | **3955** | LPCAT1 |
| 794 | SVIL | **1848** | SYNCRIP | **2902** | PSMA7 | **3956** | FOXQ1 |
| 795 | CDK12 | **1849** | EPB41L3 | **2903** | TWIST2 | **3957** | UPF1 |
| 796 | HDGF | **1850** | OCRL | **2904** | H2AC12 | **3958** | CSE1L |
| 797 | SRI | **1851** | MIR30C1 | **2905** | UBB | **3959** | SBF2 |
| 798 | BZW1 | **1852** | CARD8 | **2906** | MRPS7 | **3960** | PABPC4 |
| 799 | KHSRP | **1853** | IL17RB | **2907** | RIOK1 | **3961** | ZYX |
| 800 | MNX1 | **1854** | PPP1R10 | **2908** | SIK2 | **3962** | MIR4435-2HG |
| 801 | CD93 | **1855** | CD59 | **2909** | JAM2 | **3963** | SFRP2 |
| 802 | RAB29 | **1856** | RPL10A | **2910** | MIR20A | **3964** | SF3A2 |
| 803 | PDE5A | **1857** | MIR200C | **2911** | LONP1 | **3965** | BAIAP2 |
| 804 | GNA12 | **1858** | MCM6 | **2912** | RNF2 | **3966** | RSL1D1 |
| 805 | BLK | **1859** | CALB2 | **2913** | SF3B3 | **3967** | RAB9A |
| 806 | AKR1C2 | **1860** | MIR423 | **2914** | SNHG14 | **3968** | RPS8 |
| 807 | VPS28 | **1861** | FDFT1 | **2915** | PRKG1 | **3969** | FFAR3 |
| 808 | EIF4A3 | **1862** | FBN2 | **2916** | SUMO3 | **3970** | DIP2B |
| 809 | LPAR3 | **1863** | HOXB2 | **2917** | DLST | **3971** | EIF2B2 |
| 810 | CCNB2 | **1864** | LOC117152610 | **2918** | LOC117152611 | **3972** | RPL31 |
| 811 | MIA | **1865** | RPA2 | **2919** | LNCARSR | **3973** | NFE2L3 |
| 812 | CLIC3 | **1866** | CACNA2D4 | **2920** | ABCE1 | **3974** | PPP1R15A |
| 813 | EDA | **1867** | SNHG29 | **2921** | KRT1 | **3975** | NPC1L1 |
| 814 | TGM3 | **1868** | METAP2 | **2922** | BTLA | **3976** | MNDA |
| 815 | ARFGAP3 | **1869** | H2BC21 | **2923** | BANCR | **3977** | DARS1 |
| 816 | EPHB4 | **1870** | CTAG1B | **2924** | IQGAP2 | **3978** | GM2A |
| 817 | CNOT8 | **1871** | TRA | **2925** | EIF4G1 | **3979** | SUPT16H |
| 818 | DIAPH3 | **1872** | GTF2E2 | **2926** | TUT7 | **3980** | SLC25A12 |
| 819 | OSCP1 | **1873** | HBG2 | **2927** | TCERG1 | **3981** | TAL1 |
| 820 | LRRC59 | **1874** | DDX21 | **2928** | APOBEC3H | **3982** | CLDN18 |
| 821 | MIR138-1 | **1875** | STOML2 | **2929** | MAPK11 | **3983** | RPL34 |
| 822 | RASGRF1 | **1876** | PCYT1A | **2930** | HSPA2 | **3984** | MTHFR |
| 823 | CUBN | **1877** | TUBA8 | **2931** | SLC39A7 | **3985** | MYO1A |
| 824 | PIK3R3 | **1878** | NAXE | **2932** | LARP1 | **3986** | IQSEC1 |
| 825 | MAP1S | **1879** | PSME1 | **2933** | BLVRB | **3987** | NCR3 |
| 826 | LPP | **1880** | SUN1 | **2934** | RASA2 | **3988** | RPS9 |
| 827 | DDX39B | **1881** | ID1 | **2935** | CSNK2A2 | **3989** | PURA |
| 828 | CHTOP | **1882** | SELENBP1 | **2936** | RPL32 | **3990** | PPP6R1 |
| 829 | KAT6A | **1883** | SLC6A12 | **2937** | UBE2D1 | **3991** | IBSP |
| 830 | NR3C2 | **1884** | ASGR2 | **2938** | COPZ1 | **3992** | SLC4A4 |
| 831 | CEP72 | **1885** | TRNT1 | **2939** | SNRPD2 | **3993** | PLPPR5-AS1 |
| 832 | VDAC2 | **1886** | FGF19 | **2940** | TBXT | **3994** | NBR2 |
| 833 | THY1 | **1887** | VTI1B | **2941** | CNOT9 | **3995** | BMPR1A |
| 834 | AHNAK | **1888** | STEAP3 | **2942** | VPS35 | **3996** | TAOK3 |
| 835 | TNFAIP8 | **1889** | CTNND2 | **2943** | RAB32 | **3997** | CACNA1A |
| 836 | NKRF | **1890** | TSR1 | **2944** | OIP5-AS1 | **3998** | ZNF148 |
| 837 | PTGDR | **1891** | MC1R | **2945** | GLMN | **3999** | RBM10 |
| 838 | MIR326 | **1892** | STEAP4 | **2946** | MKNK2 | **4000** | SRA1 |
| 839 | LCOR | **1893** | KRT19 | **2947** | MS4A1 | **4001** | DCP1A |
| 840 | TUBGCP6 | **1894** | PCAT6 | **2948** | MATK | **4002** | ARHGAP30 |
| 841 | SPTBN4 | **1895** | KDM6A | **2949** | CCL27 | **4003** | UTP18 |
| 842 | ALPK1 | **1896** | STK16 | **2950** | EPS15 | **4004** | PPIB |
| 843 | PAX6 | **1897** | GBA2 | **2951** | SPATA2 | **4005** | MCRS1 |
| 844 | OGT | **1898** | F2RL3 | **2952** | CASP6 | **4006** | FH |
| 845 | DDIT4 | **1899** | AHCY | **2953** | POMC | **4007** | RPL36 |
| 846 | RAD21 | **1900** | PGD | **2954** | PDE4D | **4008** | MIR381 |
| 847 | ACTBL2 | **1901** | AKAP12 | **2955** | UBE2D3 | **4009** | CACYBP |
| 848 | POF1B | **1902** | MAGI2 | **2956** | LPAR1 | **4010** | TWF1 |
| 849 | CA4 | **1903** | EIF4G2 | **2957** | BCOR | **4011** | TMEM30A |
| 850 | ATG4B | **1904** | SLC7A8 | **2958** | RAB11B | **4012** | ACACB |
| 851 | CTBP1 | **1905** | RALY | **2959** | CSNK1G2 | **4013** | TNIK |
| 852 | IPO7 | **1906** | NANOG | **2960** | TRIO | **4014** | RPL3 |
| 853 | BATF3 | **1907** | MIR9-2 | **2961** | HAS2 | **4015** | DPM1 |
| 854 | SGMS2 | **1908** | CACNA2D2 | **2962** | CACNA2D3 | **4016** | WDR83OS |
| 855 | SLC25A6 | **1909** | PGM1 | **2963** | OVOL2 | **4017** | MIRLET7E |
| 856 | MIR148A | **1910** | CACNA1H | **2964** | CACNB4 | **4018** | CNN2 |
| 857 | LINC00665 | **1911** | DRD5 | **2965** | LGALS13 | **4019** | TRIM5 |
| 858 | TNFSF8 | **1912** | RNF41 | **2966** | ITGA2B | **4020** | DCHS1 |
| 859 | NDC80 | **1913** | RPL23 | **2967** | CLIP1 | **4021** | HTR2B |
| 860 | SLIT2 | **1914** | BRD2 | **2968** | MIR497 | **4022** | RAB37 |
| 861 | SLC25A11 | **1915** | SERPINH1 | **2969** | CCAR1 | **4023** | PKP3 |
| 862 | SHARPIN | **1916** | PHLDB2 | **2970** | PORCN | **4024** | DCST1-AS1 |
| 863 | PSMA1 | **1917** | PSMA3 | **2971** | FOXK2 | **4025** | DNAJA2 |
| 864 | CDX1 | **1918** | AKAP13 | **2972** | ABCB4 | **4026** | SEC13 |
| 865 | SEPTIN7 | **1919** | RPL21 | **2973** | MLH1 | **4027** | DISP1 |
| 866 | KLHDC7B-DT | **1920** | ATP6V0D2 | **2974** | PPID | **4028** | ADD1 |
| 867 | KIF13B | **1921** | BLZF1 | **2975** | BHLHE40 | **4029** | DROSHA |
| 868 | SMG7 | **1922** | ELOC | **2976** | SFN | **4030** | SOX4 |
| 869 | SGMS1 | **1923** | SNHG20 | **2977** | LDHB | **4031** | PELP1 |
| 870 | AVPR2 | **1924** | LINC00240 | **2978** | ADORA1 | **4032** | DOK1 |
| 871 | LIG3 | **1925** | ATF7 | **2979** | PRKACB | **4033** | KRT7 |
| 872 | SQOR | **1926** | FLVCR1 | **2980** | PTPRE | **4034** | HNF1B |
| 873 | MDC1 | **1927** | RHBDF1 | **2981** | TOE1 | **4035** | RPS6KA6 |
| 874 | SLC2A10 | **1928** | PITX1 | **2982** | VARS1 | **4036** | EXOSC2 |
| 875 | MARK4 | **1929** | MIR4505 | **2983** | LINC01010 | **4037** | RCAN1 |
| 876 | BBC3 | **1930** | ERAP2 | **2984** | ALDH1A2 | **4038** | ARL1 |
| 877 | PKN1 | **1931** | ODF1 | **2985** | ETFA | **4039** | ATG12 |
| 878 | LPIN1 | **1932** | HSD17B10 | **2986** | ATP9A | **4040** | TUBGCP3 |
| 879 | IRF6 | **1933** | DEFA1 | **2987** | SIPA1 | **4041** | MYL9 |
| 880 | SLC39A5 | **1934** | ZMIZ1 | **2988** | GPR174 | **4042** | SLC12A2 |
| 881 | SERPING1 | **1935** | RPL22 | **2989** | CLDN4 | **4043** | RUNX3 |
| 882 | ZIC2 | **1936** | NAPSA | **2990** | PPP6C | **4044** | KCNA2 |
| 883 | CDC42SE2 | **1937** | SLC27A4 | **2991** | REN | **4045** | CYC1 |
| 884 | TRMT112 | **1938** | BRAP | **2992** | GJB6 | **4046** | YIPF5 |
| 885 | STX5 | **1939** | RPL23A | **2993** | HNF4A | **4047** | NFYA |
| 886 | ACBD3 | **1940** | PPL | **2994** | ARPC4 | **4048** | KIF16B |
| 887 | HAUS4 | **1941** | LEMD3 | **2995** | ITGB6 | **4049** | GRHL3 |
| 888 | CDK11B | **1942** | RPS17 | **2996** | ACVR1 | **4050** | PLXDC1 |
| 889 | DANCR | **1943** | SKP1 | **2997** | SYTL2 | **4051** | INSR |
| 890 | TM6SF1 | **1944** | BAG6 | **2998** | MLPH | **4052** | UBAP2L |
| 891 | IPO5 | **1945** | EDN3 | **2999** | PRDM2 | **4053** | OSBP |
| 892 | NUP133 | **1946** | MED17 | **3000** | AIMP2 | **4054** | PRDX6 |
| 893 | RING1 | **1947** | CD6 | **3001** | S100A6 | **4055** | KLK11 |
| 894 | SCAP | **1948** | OAT | **3002** | MIR4319 | **4056** | RCN1 |
| 895 | GEM | **1949** | TWF2 | **3003** | NEIL3 | **4057** | BRD1 |
| 896 | BCAT1 | **1950** | GP2 | **3004** | MYL12B | **4058** | TPM4 |
| 897 | EFEMP1 | **1951** | CERK | **3005** | SSR1 | **4059** | SPOP |
| 898 | CDK11A | **1952** | DHFR | **3006** | POU2F1 | **4060** | TCF12 |
| 899 | SRSF7 | **1953** | NSF | **3007** | ADAM1A | **4061** | PPIG |
| 900 | LRRFIP2 | **1954** | ATP5PB | **3008** | PLCD4 | **4062** | ADGRD1 |
| 901 | ATG13 | **1955** | LTB4R2 | **3009** | SNRNP70 | **4063** | MIR32 |
| 902 | ADCYAP1R1 | **1956** | HMMR-AS1 | **3010** | TAGLN2 | **4064** | CNP |
| 903 | RETNLB | **1957** | S100A10 | **3011** | TRAPPC14 | **4065** | SFRP4 |
| 904 | PUM1 | **1958** | CORO7 | **3012** | RPL19 | **4066** | CPNE8 |
| 905 | REG4 | **1959** | RPL5 | **3013** | PDIA6 | **4067** | KLHL21 |
| 906 | CEP170 | **1960** | SBDS | **3014** | MIR19B1 | **4068** | MAGEA3 |
| 907 | MIB1 | **1961** | CDC25C | **3015** | MCM5 | **4069** | MIR186 |
| 908 | BLVRA | **1962** | RPL17 | **3016** | TWNK | **4070** | DOCK11 |
| 909 | ST2 | **1963** | RBL1 | **3017** | GCH1 | **4071** | MIR22 |
| 910 | RRP1B | **1964** | LETM1 | **3018** | SYNE2 | **4072** | ASAP1 |
| 911 | CSPP1 | **1965** | SEC61B | **3019** | GHR | **4073** | MARVELD2 |
| 912 | HSPB6 | **1966** | GAP43 | **3020** | NXF1 | **4074** | PDIA5 |
| 913 | DLX6-AS1 | **1967** | KLK3 | **3021** | PRPS1 | **4075** | CORO1C |
| 914 | ECHS1 | **1968** | MIR30C2 | **3022** | AKAP8L | **4076** | TBC1D10A |
| 915 | LENG8 | **1969** | CACNB2 | **3023** | RTCA | **4077** | ZC3H11A |
| 916 | KIF11 | **1970** | SNAP29 | **3024** | LTBP1 | **4078** | CGB5 |
| 917 | SUSD2 | **1971** | MIR199A2 | **3025** | CDA | **4079** | KCMF1 |
| 918 | SAMD9 | **1972** | FAP | **3026** | RPL9 | **4080** | RCC1 |
| 919 | GLS2 | **1973** | POTEE | **3027** | PLXNC1 | **4081** | DGCR8 |
| 920 | IL36A | **1974** | C4B | **3028** | TRN-GTT2-1 | **4082** | CAMK4 |
| 921 | PRDX3 | **1975** | RAB11FIP1 | **3029** | PTGES2 | **4083** | NPEPPS |
| 922 | SOST | **1976** | MSRB1 | **3030** | RHEB | **4084** | AXIN1 |
| 923 | LTC4S | **1977** | TRIM27 | **3031** | STK38L | **4085** | PAICS |
| 924 | SLC6A2 | **1978** | PTPN9 | **3032** | RXFP1 | **4086** | FGF8 |
| 925 | NPHP3-ACAD11 | **1979** | TRIM63 | **3033** | PELI1 | **4087** | ADGRA3 |
| 926 | CYP4V2 | **1980** | UBASH3B | **3034** | MIR324 | **4088** | TUBB1 |
| 927 | MB | **1981** | LINC01150 | **3035** | FKBP15 | **4089** | CRTC2 |
| 928 | SLC39A1 | **1982** | EIF2B5 | **3036** | PDE8A | **4090** | DGKQ |
| 929 | PAK2 | **1983** | WRAP53 | **3037** | NCSTN | **4091** | CSTF2 |
| 930 | HNRNPA3 | **1984** | MAP3K11 | **3038** | CLDN23 | **4092** | RPS16 |
| 931 | ARHGEF12 | **1985** | HOXA9 | **3039** | COMP | **4093** | PRRC2C |
| 932 | RAPGEF1 | **1986** | PSMB10 | **3040** | TP53BP1 | **4094** | PRDX4 |
| 933 | ANAPC2 | **1987** | AGPAT1 | **3041** | MIR124-3 | **4095** | ALKBH3 |
| 934 | ARHGEF28 | **1988** | ZNF202 | **3042** | VAMP2 | **4096** | ARHGDIB |
| 935 | RARG | **1989** | LCN10 | **3043** | NFE2L1 | **4097** | ARHGAP5 |
| 936 | RETSAT | **1990** | WASHC4 | **3044** | ARL5B | **4098** | ALDH18A1 |
| 937 | RAB7B | **1991** | HLA-DRB4 | **3045** | SMC2 | **4099** | TBC1D15 |
| 938 | SAFB2 | **1992** | TULP1 | **3046** | ATG14 | **4100** | KRT79 |
| 939 | SPRY1 | **1993** | PER1 | **3047** | NSDHL | **4101** | MIR181B1 |
| 940 | ALDH1A1 | **1994** | USF2 | **3048** | DGAT2 | **4102** | PKP1 |
| 941 | APOBEC3C | **1995** | POLA2 | **3049** | TRN-GTT2-7 | **4103** | POLDIP3 |
| 942 | STX12 | **1996** | HAUS6 | **3050** | TPM1 | **4104** | DYNC1H1 |
| 943 | HSPA14 | **1997** | CRIPTO | **3051** | SKP2 | **4105** | SPINK1 |
| 944 | PAXIP1 | **1998** | CLUH | **3052** | NALT1 | **4106** | VGF |
| 945 | NCOA1 | **1999** | H4C16 | **3053** | GDI1 | **4107** | DSP |
| 946 | IGFBP4 | **2000** | RPS20 | **3054** | ARFGEF2 | **4108** | RHOU |
| 947 | POLR2B | **2001** | RPL24 | **3055** | LINC00662 | **4109** | LY75 |
| 948 | CASP14 | **2002** | FFAR1 | **3056** | SYTL1 | **4110** | EPS8 |
| 949 | SLIT1 | **2003** | CYP7A1 | **3057** | ITPR2 | **4111** | PSMF1 |
| 950 | SORBS1 | **2004** | STX2 | **3058** | DUT | **4112** | RNY3 |
| 951 | EFNA2 | **2005** | ANGPTL3 | **3059** | PLK2 | **4113** | GSTM2 |
| 952 | KRT77 | **2006** | DDA1 | **3060** | RAB3GAP1 | **4114** | PSMC2 |
| 953 | NECAB3 | **2007** | MIR92A2 | **3061** | NUP107 | **4115** | CYRIA |
| 954 | RAP2B | **2008** | MIR199A1 | **3062** | KRT85 | **4116** | NACA |
| 955 | FCHO2 | **2009** | TSG101 | **3063** | RPS4X | **4117** | CALML3 |
| 956 | ACTG2 | **2010** | MYCBP2 | **3064** | PRMT3 | **4118** | TNFRSF13C |
| 957 | ZFP91 | **2011** | ATP1B2 | **3065** | SON | **4119** | PLOD2 |
| 958 | CKLF | **2012** | MIR92A1 | **3066** | TNNT2 | **4120** | OXT |
| 959 | DCTN4 | **2013** | CTAGE1 | **3067** | SOX9 | **4121** | SMPX |
| 960 | GUK1 | **2014** | FIG4 | **3068** | INF2 | **4122** | VEZF1 |
| 961 | ATP11A | **2015** | NAAA | **3069** | RBM14 | **4123** | IDI1 |
| 962 | MPIG6B | **2016** | RAB34 | **3070** | TONSL | **4124** | RBCK1 |
| 963 | PPA1 | **2017** | DHX40 | **3071** | ACVR1B | **4125** | GSTT2B |
| 964 | MAST2 | **2018** | RPS10 | **3072** | RPL4 | **4126** | RPS13 |
| 965 | NPR1 | **2019** | HMOX2 | **3073** | STRN4 | **4127** | CDKN2B-AS1 |
| 966 | PSME3 | **2020** | RDH11 | **3074** | SNHG17 | **4128** | LMAN1 |
| 967 | HINFP | **2021** | MIR769 | **3075** | EVL | **4129** | RREB1 |
| 968 | DOCK4 | **2022** | TOP1 | **3076** | NDRG1 | **4130** | CLPB |
| 969 | AXIN2 | **2023** | CDC42EP1 | **3077** | STXBP1 | **4131** | MIR1291 |
| 970 | JAG2 | **2024** | PTH1R | **3078** | STK39 | **4132** | TRIM56 |
| 971 | HAR1B | **2025** | CCT6A | **3079** | LIMK2 | **4133** | WDR6 |
| 972 | MIR296 | **2026** | VEGFB | **3080** | EIF3A | **4134** | PTGES3 |
| 973 | GATAD2A | **2027** | XIST | **3081** | MARCHF2 | **4135** | GRIN1 |
| 974 | SLC22A12 | **2028** | GTF2I | **3082** | PEPD | **4136** | DES |
| 975 | ELMO2 | **2029** | HMMR | **3083** | SPC25 | **4137** | SH3KBP1 |
| 976 | GPR37 | **2030** | RPL35A | **3084** | RPS6KA3 | **4138** | CALCRL |
| 977 | RAB3B | **2031** | TFF2 | **3085** | THRAP3 | **4139** | RPS23 |
| 978 | COL11A1 | **2032** | EIF2S3 | **3086** | CBFB | **4140** | FALEC |
| 979 | GYPA | **2033** | CACNA1G | **3087** | TRPM4 | **4141** | SERF2 |
| 980 | CRNDE | **2034** | RPRD2 | **3088** | CDC42BPB | **4142** | OPA1 |
| 981 | MACC1 | **2035** | PTP4A1 | **3089** | MIR543 | **4143** | LAMA2 |
| 982 | SLC20A1 | **2036** | SERPINB9 | **3090** | TKTL1 | **4144** | MIR16-1 |
| 983 | BRCA2 | **2037** | GULP1 | **3091** | POLR1A | **4145** | MIR9-3 |
| 984 | STAMBP | **2038** | NDRG2 | **3092** | TNFRSF21 | **4146** | STAM2 |
| 985 | PRPF6 | **2039** | MYL2 | **3093** | NT5DC2 | **4147** | LAT |
| 986 | GLO1 | **2040** | SYNPO | **3094** | PEA15 | **4148** | PMP22 |
| 987 | WNT6 | **2041** | SPDEF | **3095** | TBL1XR1 | **4149** | PBRM1 |
| 988 | KLRC1 | **2042** | ZNF143 | **3096** | PPP1R7 | **4150** | DEFA5 |
| 989 | GAR1 | **2043** | TFPT | **3097** | HOTAIR | **4151** | NDUFV3 |
| 990 | UACA | **2044** | CNTNAP1 | **3098** | PROCR | **4152** | SRD5A1 |
| 991 | SLC6A4 | **2045** | CPSF7 | **3099** | RPS29 | **4153** | MIR206 |
| 992 | RPS2 | **2046** | PRKG2 | **3100** | ANXA2P2 | **4154** | MIR30D |
| 993 | HBD | **2047** | PLS3 | **3101** | HPGDS | **4155** | POLR2C |
| 994 | RAD18 | **2048** | HSPA1B | **3102** | MYO5C | **4156** | CSNK1G1 |
| 995 | TYRP1 | **2049** | TNFAIP8L1 | **3103** | H3C14 | **4157** | MIR16-2 |
| 996 | PIBF1 | **2050** | ZG16B | **3104** | FUBP3 | **4158** | GANAB |
| 997 | ENPP2 | **2051** | ZNF281 | **3105** | ZNF687 | **4159** | CYTH3 |
| 998 | A2ML1 | **2052** | UVRAG | **3106** | GJB1 | **4160** | MLXIPL |
| 999 | HCAR3 | **2053** | PHLDB1 | **3107** | COL10A1 | **4161** | PTPRG |
| 1000 | TRL-TAG1-1 | **2054** | HLTF | **3108** | SMARCA2 | **4162** | LFNG |
| 1001 | TIMM8A | **2055** | MIR340 | **3109** | CAST | **4163** | TASOR |
| 1002 | ERO1A | **2056** | DHX38 | **3110** | SAMSN1 | **4164** | APRT |
| 1003 | INTS1 | **2057** | DHCR24 | **3111** | ANAPC1 | **4165** | UTS2 |
| 1004 | ARHGEF19 | **2058** | DNAJB6 | **3112** | BRD3 | **4166** | TUBB4B |
| 1005 | STIM2 | **2059** | KRT10 | **3113** | MIR671 | **4167** | C1QB |
| 1006 | NLRC5 | **2060** | TH | **3114** | WDR77 | **4168** | MED4 |
| 1007 | NPY | **2061** | PTGER1 | **3115** | CS | **4169** | DKC1 |
| 1008 | TAX1BP3 | **2062** | KLHL12 | **3116** | AFG3L2 | **4170** | YKT6 |
| 1009 | UBE2L3 | **2063** | TIAM2 | **3117** | RSPO2 | **4171** | IRS1 |
| 1010 | RPPH1 | **2064** | MIR10B | **3118** | SIAH2 | **4172** | RPL8 |
| 1011 | SPRED1 | **2065** | CAHM | **3119** | MAZ | **4173** | UBE2S |
| 1012 | GNB3 | **2066** | CCK | **3120** | ETF1 | **4174** | CEACAM7 |
| 1013 | LGALS4 | **2067** | PICALM | **3121** | SHB | **4175** | CHD3 |
| 1014 | KIF3B | **2068** | FABP12 | **3122** | CLASRP | **4176** | SNW1 |
| 1015 | SUPT6H | **2069** | ATP6V1E1 | **3123** | TFG | **4177** | FLG2 |
| 1016 | HAUS3 | **2070** | NPHP4 | **3124** | APH1A | **4178** | RTN3 |
| 1017 | AMBP | **2071** | CCNE1 | **3125** | MIR181A2 | **4179** | TXNDC5 |
| 1018 | KDM2A | **2072** | COLGALT1 | **3126** | HLA-F-AS1 | **4180** | DEK |
| 1019 | RARS1 | **2073** | DGKA | **3127** | SYT1 | **4181** | TOP3B |
| 1020 | PRPS2 | **2074** | RPL30 | **3128** | SCAMP3 | **4182** | SEC16A |
| 1021 | TACR2 | **2075** | MPG | **3129** | ARF1 | **4183** | ZNF451 |
| 1022 | MED20 | **2076** | IARS1 | **3130** | DDX50 | **4184** | RRP12 |
| 1023 | F7 | **2077** | GFM1 | **3131** | GNB5 | **4185** | SLC6A6 |
| 1024 | CRH | **2078** | CCNL2 | **3132** | MIR130B | **4186** | HOXA10 |
| 1025 | HELZ2 | **2079** | PIP5K1B | **3133** | LAMTOR1 | **4187** | LINC01727 |
| 1026 | RLN2 | **2080** | RALGDS | **3134** | PCNX3 | **4188** | HAUS1 |
| 1027 | H3-3A | **2081** | ITGA1 | **3135** | TRIM59 | **4189** | NDUFAB1 |
| 1028 | SIRT4 | **2082** | EPS15L1 | **3136** | ZDHHC5 | **4190** | SNRPB |
| 1029 | AFAP1-AS1 | **2083** | UBE2O | **3137** | SHBG | **4191** | ATG16L2 |
| 1030 | ADAMTS5 | **2084** | MMP11 | **3138** | RYK | **4192** | ATP6V1H |
| 1031 | MIR100 | **2085** | SLC47A1 | **3139** | SLC12A3 | **4193** | APAF1 |
| 1032 | H1-3 | **2086** | ANKRD28 | **3140** | B3GALNT2 | **4194** | PCBP1 |
| 1033 | NID1 | **2087** | ALPP | **3141** | ANO1 | **4195** | STT3A |
| 1034 | SMYD3 | **2088** | TBC1D14 | **3142** | MAG | **4196** | MPZL1 |
| 1035 | CNTROB | **2089** | KIR2DL4 | **3143** | NDUFS3 | **4197** | GTF3C4 |
| 1036 | IFT74 | **2090** | PCBP2 | **3144** | TOP2B | **4198** | H2BC12L |
| 1037 | RAB23 | **2091** | MIR27B | **3145** | YLPM1 | **4199** | CDH3 |
| 1038 | NPR3 | **2092** | PRSS3 | **3146** | MED16 | **4200** | TRABD |
| 1039 | ZCCHC8 | **2093** | PREB | **3147** | EFNB1 | **4201** | INTS2 |
| 1040 | UBE3A | **2094** | WDR47 | **3148** | ISOC2 | **4202** | LAMB2 |
| 1041 | SARAF | **2095** | ABCG4 | **3149** | GSTK1 | **4203** | DMAP1 |
| 1042 | DNAJA3 | **2096** | SMPD2 | **3150** | DDRGK1 | **4204** | MICA |
| 1043 | RTRAF | **2097** | JPT2 | **3151** | PSMD10 | **4205** | FGF20 |
| 1044 | PPM1D | **2098** | SERPINB3 | **3152** | GYPE | **4206** | RAP2A |
| 1045 | TIAL1 | **2099** | RHBG | **3153** | GH1 | **4207** | TUT4 |
| 1046 | ANXA3 | **2100** | SLA2 | **3154** | LGR4 | **4208** | DNAJC7 |
| 1047 | YTHDC2 | **2101** | STARD8 | **3155** | H2AZ1 | **4209** | AFAP1AS1 |
| 1048 | VNN1 | **2102** | AIP | **3156** | RIPOR2 | **4210** | PELATON |
| 1049 | MIR505 | **2103** | EMILIN1 | **3157** | CSF2RB | **4211** | CSF2RY |
| 1050 | MPEG1 | **2104** | PAM16 | **3158** | MMD2 | **4212** | TRIM35 |
| 1051 | CLEC12B | **2105** | PSTPIP2 | **3159** | CCL3L1 | **4213** | SCARA3 |
| 1052 | SLAMF8 | **2106** | TMEM229B | **3160** | MS | **4214** | AIFBL2 |
| 1053 | RA | **2107** | AD1 | **3161** | PLCA3 | **4215** | HECTD3 |
| 1054 | SHTN1 | **2108** | TTD7 | **3162** | FZD6 |  |  |

**Table S11 MP targets from GSE121410**

| NO | Target | NO | Target | NO | Target | NO | Target |
| --- | --- | --- | --- | --- | --- | --- | --- |
| 1 | KLK9 | **2030** | IL12A | **4059** | IGLC3 | **6088** | IGKC |
| 2 | TFDP1 | **2031** | NEURL4 | **4060** | SERINC3 | **6089** | SERP1 |
| 3 | RPL23 | **2032** | DUS1L | **4061** | SPATC1L | **6090** | IGHJ4 |
| 4 | PKD1 | **2033** | PRKD1 | **4062** | GIGYF1 | **6091** | CDS2 |
| 5 | ANGPTL2 | **2034** | ARHGEF1 | **4063** | IRAK2 | **6092** | SPTA1 |
| 6 | POU2AF1 | **2035** | ANKRD39 | **4064** | SFR1 | **6093** | NACA |
| 7 | MFRP | **2036** | RNF31 | **4065** | IFI30 | **6094** | BICRA |
| 8 | EIF6 | **2037** | EIF3A | **4066** | MACF1 | **6095** | HAS3 |
| 9 | CNTNAP1 | **2038** | RPL31 | **4067** | CAPN15 | **6096** | PITPNM1 |
| 10 | SELENOK | **2039** | ERFE | **4068** | ADAMTS10 | **6097** | LMNB1 |
| 11 | UBXN11 | **2040** | NPM1 | **4069** | CHMP4B | **6098** | PABPC1 |
| 12 | IGHJ3 | **2041** | RNPEPL1 | **4070** | TCEA2 | **6099** | PAXX |
| 13 | AP5B1 | **2042** | CRYM | **4071** | SNAPC4 | **6100** | DGKQ |
| 14 | DQX1 | **2043** | ABCA7 | **4072** | INPP4A | **6101** | IGHJ1 |
| 15 | POGZ | **2044** | ITPR3 | **4073** | STRN4 | **6102** | RCOR3 |
| 16 | IKBKB | **2045** | TAFAZZIN | **4074** | WWTR1 | **6103** | TAZ |
| 17 | MYL6 | **2046** | PKNOX1 | **4075** | SUB1 | **6104** | PC4 |
| 18 | TAOK2 | **2047** | ADRB1 | **4076** | STARD3NL | **6105** | TCEAL8 |
| 19 | SRPK3 | **2048** | RPL11 | **4077** | HCFC1R1 | **6106** | PCBP2 |
| 20 | GUCY1A1 | **2049** | RPL36A | **4078** | GUCA1B | **6107** | ZFHX3 |
| 21 | MTG2 | **2050** | MCTP1 | **4079** | RPS20 | **6108** | MMP14 |
| 22 | HTRA2 | **2051** | PHF1 | **4080** | RPS26 | **6109** | MMP13 |
| 23 | IGSF9 | **2052** | MED21 | **4081** | FAM120B | **6110** | DGKH |
| 24 | MAU2 | **2053** | SPG21 | **4082** | IGHJ2 | **6111** | ABTB3 |
| 25 | INSYN2A | **2054** | NAA50 | **4083** | STK38 | **6112** | OCEL1 |
| 26 | CASK | **2055** | CSN3 | **4084** | CIC | **6113** | FRS3 |
| 27 | RTCB | **2056** | YBX1 | **4085** | PDE4A | **6114** | CBX7 |
| 28 | IQGAP3 | **2057** | SH3BGRL | **4086** | HEL-S-115 | **6115** | MTHFSD |
| 29 | ZGPAT | **2058** | INSYN2B | **4087** | GLIPR1 | **6116** | LRRC18 |
| 30 | FRZB | **2059** | EEF1B2 | **4088** | JCHAIN | **6117** | MICAL1 |
| 31 | S100A8 | **2060** | SMG1 | **4089** | SEZ6L2 | **6118** | TTLL4 |
| 32 | ZBTB37 | **2061** | TUBGCP3 | **4090** | SELENOO | **6119** | CREBZF |
| 33 | CNDP2 | **2062** | YEATS4 | **4091** | ARL1 | **6120** | DUS3L |
| 34 | CMPK1 | **2063** | PEX10 | **4092** | PANK4 | **6121** | REEP2 |
| 35 | ITGA9 | **2064** | PHKA1 | **4093** | INTS1 | **6122** | GMIP |
| 36 | TLE3 | **2065** | CMTM4 | **4094** | DVL1 | **6123** | DVL1P1 |
| 37 | FAM161A | **2066** | H3-3A | **4095** | H3F3B | **6124** | CRNKL1 |
| 38 | VARS1 | **2067** | VARS2 | **4096** | ZFP82 | **6125** | GTPBP6 |
| 39 | ARPC5 | **2068** | KCNJ9 | **4097** | BRSK1 | **6126** | ZBTB4 |
| 40 | TXNRD2 | **2069** | MEX3C | **4098** | SMG5 | **6127** | HINT1 |
| 41 | ABL1 | **2070** | RBM12 | **4099** | DLGAP4 | **6128** | RTRAF |
| 42 | DCUN1D2 | **2071** | INPP5D | **4100** | RHOJ | **6129** | PARP4 |
| 43 | HAAO | **2072** | ARID3B | **4101** | RPTOR | **6130** | ADHFE1 |
| 44 | H1-2 | **2073** | ACP6 | **4102** | TMEFF1 | **6131** | PTPRM |
| 45 | SBF1 | **2074** | MYO15A | **4103** | APEH | **6132** | MARVELD1 |
| 46 | ATP5MK | **2075** | GNG11 | **4104** | CDK1 | **6133** | MCM9 |
| 47 | LSG1 | **2076** | RPS28 | **4105** | CELF1 | **6134** | UTP3 |
| 48 | EPRS1 | **2077** | QARS1 | **4106** | QARS | **6135** | ZZEF1 |
| 49 | RABGGTA | **2078** | AKAP8 | **4107** | SLC29A1 | **6136** | TBP |
| 50 | CLCN2 | **2079** | CHADL | **4108** | FBXL12 | **6137** | XRCC3 |
| 51 | TNRC18 | **2080** | BTN3A3 | **4109** | BTF3 | **6138** | BTN3A2 |
| 52 | CSNK1E | **2081** | SFI1 | **4110** | CSDE1 | **6139** | FNBP1 |
| 53 | NFX1 | **2082** | RPS25 | **4111** | CDK10 | **6140** | MAN2C1 |
| 54 | TRIM65 | **2083** | ARL10 | **4112** | TXNDC17 | **6141** | RPS8 |
| 55 | MRPL37 | **2084** | SLC2A3 | **4113** | KLHL20 | **6142** | QPRT |
| 56 | HEL-S-90n | **2085** | KANSL1 | **4114** | RAB6B | **6143** | NBEAL2 |
| 57 | NDOR1 | **2086** | TUT1 | **4115** | PRKCD | **6144** | CLCN4 |
| 58 | SPPL3 | **2087** | MAP1S | **4116** | PLD2 | **6145** | SLC17A9 |
| 59 | RPL7A | **2088** | RPL37 | **4117** | LTF | **6146** | ADAT1 |
| 60 | MAPK8IP3 | **2089** | ARAP3 | **4118** | DGLUCY | **6147** | BUD31 |
| 61 | NDUFA1 | **2090** | ZNF183 | **4119** | NFE2L1 | **6148** | NRF1 |
| 62 | ARHGAP22 | **2091** | INSIG2 | **4120** | YEATS2 | **6149** | CHD8 |
| 63 | SGCE | **2092** | PPID | **4121** | FHOD1 | **6150** | FAM98C |
| 64 | MEIS1 | **2093** | IQGAP1 | **4122** | SETD1B | **6151** | ARPC1B |
| 65 | AP1G2 | **2094** | CDC23 | **4123** | NOC2L | **6152** | ELK4 |
| 66 | HRH1 | **2095** | PCIF1 | **4124** | CYGB | **6153** | PICK1 |
| 67 | TOP3B | **2096** | JADE2 | **4125** | TGFBR1 | **6154** | TRMU |
| 68 | TTLL3 | **2097** | RAI1 | **4126** | DHX37 | **6155** | MAML3 |
| 69 | DOCK1 | **2098** | MRPL45 | **4127** | RPS17 | **6156** | MAP3K3 |
| 70 | ATP5MG | **2099** | CELF4 | **4128** | RPL28 | **6157** | TOMM34 |
| 71 | CSF2RA | **2100** | ABCC5 | **4129** | DDX51 | **6158** | ICE1 |
| 72 | OLFML3 | **2101** | MZB1 | **4130** | ADGRL3 | **6159** | XPO6 |
| 73 | RPS11 | **2102** | YWHAE | **4131** | RBM7 | **6160** | SLC27A3 |
| 74 | CEP120 | **2103** | TNRC6A | **4132** | DNAJC16 | **6161** | RPS24 |
| 75 | ATG4B | **2104** | OLA1 | **4133** | NCOA2 | **6162** | ZKSCAN7 |
| 76 | KHNYN | **2105** | WDFY4 | **4134** | RAD51D | **6163** | XKR8 |
| 77 | ARL5A | **2106** | INO80E | **4135** | SMYD3 | **6164** | RPL6 |
| 78 | POMT1 | **2107** | VASN | **4136** | RPL36AL | **6165** | SIPA1 |
| 79 | MRPS10 | **2108** | PKN1 | **4137** | DNMT3B | **6166** | D2HGDH |
| 80 | PLCB2 | **2109** | MRI1 | **4138** | RELB | **6167** | SMPDL3B |
| 81 | PLN | **2110** | SLC2A6 | **4139** | AGAP2 | **6168** | ATP5IF1 |
| 82 | SAMSN1 | **2111** | KMT2B | **4140** | MEGF8 | **6169** | TASOR2 |
| 83 | RNF216 | **2112** | ZFYVE27 | **4141** | MDM4 | **6170** | SUGP2 |
| 84 | FRMD6 | **2113** | KMT2C | **4142** | HMGN1 | **6171** | TMEM120B |
| 85 | GPR107 | **2114** | DENND1C | **4143** | KLF5 | **6172** | CKLF |
| 86 | TBC1D8B | **2115** | NEDD8 | **4144** | ZCCHC7 | **6173** | UBL5 |
| 87 | UBALD2 | **2116** | NF1 | **4145** | ATP10D | **6174** | DISC1 |
| 88 | TSNAX-DISC1 | **2117** | TMBIM1 | **4146** | RPL29 | **6175** | EIF1AX |
| 89 | STARD13 | **2118** | PTP4A1 | **4147** | AGPAT2 | **6176** | CD207 |
| 90 | PLTP | **2119** | ZFYVE28 | **4148** | RNF17 | **6177** | NSUN5 |
| 91 | CCNT2 | **2120** | NHLRC2 | **4149** | PFDN5 | **6178** | PDCD11 |
| 92 | YJEFN3 | **2121** | SLC6A12 | **4150** | TRMT2A | **6179** | SIKE1 |
| 93 | MAP3K5 | **2122** | MAPK6 | **4151** | NPRL3 | **6180** | TRIM3 |
| 94 | UNC13D | **2123** | ARRB2 | **4152** | SPCS1 | **6181** | PSD4 |
| 95 | ANKS6 | **2124** | IRF4 | **4153** | PWWP3A | **6182** | MUM1 |
| 96 | MACROH2A1 | **2125** | JOSD2 | **4154** | CAMP | **6183** | CHAMP1 |
| 97 | CAMTA1 | **2126** | COL27A1 | **4155** | PTCHD1 | **6184** | BICD2 |
| 98 | SERTAD2 | **2127** | RAB40C | **4156** | ITPR2 | **6185** | CHST15 |
| 99 | KANSL3 | **2128** | B3GNT7 | **4157** | PIGV | **6186** | UCKL1 |
| 100 | RECQL5 | **2129** | AGMO | **4158** | MAP3K4 | **6187** | DPH2 |
| 101 | ZYX | **2130** | HS3ST3A1 | **4159** | PHC3 | **6188** | MGP |
| 102 | RPS29 | **2131** | HNRNPU | **4160** | COX5A | **6189** | ORM2 |
| 103 | CNR2 | **2132** | MAMDC4 | **4161** | KMT5B | **6190** | RPL39 |
| 104 | MARF1 | **2133** | P3H3 | **4162** | ADCY9 | **6191** | COTL1 |
| 105 | PDCL3 | **2134** | MICALL1 | **4163** | ANPEP | **6192** | AKNA |
| 106 | DENND4B | **2135** | SLC23A2 | **4164** | SZT2 | **6193** | UROD |
| 107 | TANC2 | **2136** | TBPL1 | **4165** | PLPPR4 | **6194** | WDR47 |
| 108 | PRKCH | **2137** | EARS2 | **4166** | DEAF1 | **6195** | SPN |
| 109 | ENO3 | **2138** | SYT11 | **4167** | RALGPS1 | **6196** | COG8 |
| 110 | OAZ1 | **2139** | NT5C2 | **4168** | NDNF | **6197** | KLHDC1 |
| 111 | PLCXD1 | **2140** | THADA | **4169** | PTPRS | **6198** | PER2 |
| 112 | RPL4 | **2141** | CDK5RAP2 | **4170** | UBR1 | **6199** | KMT2E |
| 113 | GBA2 | **2142** | CD83 | **4171** | CETN3 | **6200** | SCAND1 |
| 114 | MTX3 | **2143** | ABCA5 | **4172** | RPL19 | **6201** | CDV3 |
| 115 | KRTCAP3 | **2144** | MYO1C | **4173** | MYO1E | **6202** | ARFRP1 |
| 116 | SLC38A1 | **2145** | INIP | **4174** | ELOB | **6203** | NUDCD3 |
| 117 | ARRB1 | **2146** | NELFA | **4175** | CERS6 | **6204** | REXO1 |
| 118 | DNAJC7 | **2147** | MALSU1 | **4176** | PHF21B | **6205** | UBE2R2 |
| 119 | FAM193B | **2148** | BTBD9 | **4177** | CCDC137 | **6206** | PAX5 |
| 120 | CEACAM18 | **2149** | PTPRO | **4178** | PTPRU | **6207** | CD302 |
| 121 | ADAM10 | **2150** | TSPAN13 | **4179** | TM4SF13 | **6208** | PSTK |
| 122 | CTBP2 | **2151** | CAMK2B | **4180** | ANKRD23 | **6209** | PSMF1 |
| 123 | ASCC2 | **2152** | SLC9A8 | **4181** | GSTO1 | **6210** | HEL-S-21 |
| 124 | SFSWAP | **2153** | IL7R | **4182** | CLIP2 | **6211** | TMEM81 |
| 125 | MTMR11 | **2154** | SPG7 | **4183** | MTERF3 | **6212** | INCA1 |
| 126 | MMAA | **2155** | SLC7A5P2 | **4184** | N4BP2L2 | **6213** | METTL3 |
| 127 | DMAC1 | **2156** | METAP1 | **4185** | SLC25A4 | **6214** | ZCCHC4 |
| 128 | ALKBH1 | **2157** | CIZ1 | **4186** | COG2 | **6215** | PSMC3 |
| 129 | PRRT3 | **2158** | EDRF1 | **4187** | SMARCA2 | **6216** | NCKAP5 |
| 130 | RNF167 | **2159** | TBKBP1 | **4188** | TG | **6217** | METTL9 |
| 131 | TMA7 | **2160** | ITGA6 | **4189** | WDR37 | **6218** | EIF4B |
| 132 | CPSF1 | **2161** | PPIL2 | **4190** | DDX23 | **6219** | KRR1 |
| 133 | MINK1 | **2162** | CNPY2 | **4191** | TNPO3 | **6220** | DYNC1LI2 |
| 134 | ILKAP | **2163** | FKTN | **4192** | FUNDC2 | **6221** | PRDX1 |
| 135 | TTC17 | **2164** | MYADM | **4193** | FTH1 | **6222** | GPSM1 |
| 136 | RPS27L | **2165** | TYK2 | **4194** | APIP | **6223** | ASB16 |
| 137 | TMED10 | **2166** | TRPM4 | **4195** | UQCRB | **6224** | CLTA |
| 138 | ATG16L1 | **2167** | LIMK2 | **4196** | BAHD1 | **6225** | SCAF8 |
| 139 | TAFA3 | **2168** | MFNG | **4197** | SLC30A1 | **6226** | ZSCAN20 |
| 140 | ZFR2 | **2169** | PDSS2 | **4198** | RINT1 | **6227** | RUNX1 |
| 141 | HNRNPUL2 | **2170** | BCL2 | **4199** | CYCS | **6228** | ULK3 |
| 142 | SETD5 | **2171** | FHIP2B | **4200** | RPL15 | **6229** | CXCL17 |
| 143 | FCER1G | **2172** | TRIP12 | **4201** | PAN2 | **6230** | NLRP4 |
| 144 | RDH14 | **2173** | RAP1B | **4202** | MRGBP | **6231** | PRKD2 |
| 145 | ADCK2 | **2174** | RHBDF1 | **4203** | CAMTA2 | **6232** | DDX39B |
| 146 | TRIR | **2175** | EIF3H | **4204** | EIF3S3 | **6233** | LRCH4 |
| 147 | RPL23A | **2176** | TRMT1 | **4205** | CD82 | **6234** | FAM133B |
| 148 | DHX30 | **2177** | IFT172 | **4206** | STAT6 | **6235** | DGKD |
| 149 | SH2B3 | **2178** | NR3C1 | **4207** | UQCC1 | **6236** | PAG1 |
| 150 | USP7 | **2179** | NFRKB | **4208** | STXBP5 | **6237** | TMPPE |
| 151 | DEDD | **2180** | PRPS1L1 | **4209** | UBR3 | **6238** | ST3GAL6 |
| 152 | PHC2 | **2181** | RBM4 | **4210** | KCTD13 | **6239** | CACNA1A |
| 153 | TRIM11 | **2182** | ELMO3 | **4211** | CCNL2 | **6240** | PRDM15 |
| 154 | TAF1A | **2183** | GDI1 | **4212** | SMARCB1 | **6241** | NF2 |
| 155 | MEFV | **2184** | DYNLL1 | **4213** | DLC1 | **6242** | DLEC1 |
| 156 | INVS | **2185** | A4GALT | **4214** | ETFA | **6243** | MED26 |
| 157 | MIB2 | **2186** | RPL14 | **4215** | TM6SF1 | **6244** | GNPTG |
| 158 | RJD9 | **2187** | FAM131A | **4216** | SMIM14 | **6245** | TMSB10 |
| 159 | PIK3C2A | **2188** | GTF3C2 | **4217** | MTCL2 | **6246** | CDC37 |
| 160 | MBD5 | **2189** | CCDC159 | **4218** | RUBCNL | **6247** | MRPL2 |
| 161 | TOMM7 | **2190** | TOM7 | **4219** | MAP3K9 | **6248** | UTP25 |
| 162 | GABARAPL2 | **2191** | L3MBTL2 | **4220** | NCOA3 | **6249** | EPS15L1 |
| 163 | B3GNTL1 | **2192** | DPY19L3 | **4221** | KLHL13 | **6250** | GDF15 |
| 164 | RAP2C | **2193** | EME2 | **4222** | OMA1 | **6251** | ARID1A |
| 165 | IL1R2 | **2194** | CEBPD | **4223** | FBXO40 | **6252** | ARV1 |
| 166 | NDUFA2 | **2195** | CCL2 | **4224** | GTPBP2 | **6253** | ITGAM |
| 167 | CADM1 | **2196** | SARM1 | **4225** | TCEAL9 | **6254** | EMB |
| 168 | TUBB4A | **2197** | NPHP3 | **4226** | PHLPP2 | **6255** | KIF3C |
| 169 | ICOSLG | **2198** | UQCRH | **4227** | YWHAQ | **6256** | PRDM11 |
| 170 | LENG8 | **2199** | B3GALT2 | **4228** | ARRDC1 | **6257** | ZBTB16 |
| 171 | CHID1 | **2200** | POLRMT | **4229** | LMAN2L | **6258** | EDC4 |
| 172 | DYNC1H1 | **2201** | COPZ1 | **4230** | PATZ1 | **6259** | BZW1 |
| 173 | PPM1A | **2202** | SNRPE | **4231** | CTDSP2 | **6260** | SCP2 |
| 174 | SYCP2 | **2203** | CTSW | **4232** | EIF1 | **6261** | SUI1 |
| 175 | PSMB1 | **2204** | ETS1 | **4233** | UBA7 | **6262** | DDX55 |
| 176 | ATP8A1 | **2205** | SMURF1 | **4234** | SYMPK | **6263** | HAUS5 |
| 177 | TFEB | **2206** | AlphaTFEB | **4235** | LRCH1 | **6264** | MTOR |
| 178 | FHL3 | **2207** | SPEG | **4236** | CCL7 | **6265** | MCP-3 |
| 179 | RICTOR | **2208** | ELAC1 | **4237** | DAZAP2 | **6266** | MBTPS1 |
| 180 | TRAPPC8 | **2209** | DHRS7B | **4238** | RAB8B | **6267** | EXOSC8 |
| 181 | GALNT4 | **2210** | RPL21 | **4239** | MKNK1 | **6268** | FGD3 |
| 182 | ZNF823 | **2211** | ZFP36 | **4240** | FUT11 | **6269** | CCDC171 |
| 183 | MYO7A | **2212** | SEC61B | **4241** | CTDNEP1 | **6270** | TRIP6 |
| 184 | CD19 | **2213** | UNK | **4242** | EML6 | **6271** | RPS12 |
| 185 | CFL1 | **2214** | ZC3H18 | **4243** | RNF13 | **6272** | MEIS3 |
| 186 | CLASRP | **2215** | DBNDD1 | **4244** | NR2C2 | **6273** | TRIM24 |
| 187 | ACTG1 | **2216** | PTPN1 | **4245** | FUNDC1 | **6274** | CEP295NL |
| 188 | SEPTIN2 | **2217** | SPNS3 | **4246** | CALD1 | **6275** | SP4 |
| 189 | XIAP | **2218** | VAPA | **4247** | NUP133 | **6276** | MICU3 |
| 190 | RPLP0 | **2219** | HPS1 | **4248** | NCKIPSD | **6277** | CHPF2 |
| 191 | KHSRP | **2220** | PDGFA | **4249** | LIPE | **6278** | TMF1 |
| 192 | RPL41 | **2221** | ARFGAP1 | **4250** | ARFGAP3 | **6279** | MEF2A |
| 193 | NR1H3 | **2222** | PFDN6 | **4251** | HKE2 | **6280** | PACS2 |
| 194 | BZW2 | **2223** | GFM2 | **4252** | KMT2A | **6281** | GRK3 |
| 195 | ABHD6 | **2224** | FBXL17 | **4253** | ORAI3 | **6282** | SUN1 |
| 196 | MOSMO | **2225** | MUTYH | **4254** | ASPHD1 | **6283** | SPATA2L |
| 197 | S100A13 | **2226** | BEND6 | **4255** | GLI1 | **6284** | SNRNP70 |
| 198 | DKFZp434N041 | **2227** | DGUOK | **4256** | ZMAT2 | **6285** | CYB561D1 |
| 199 | WDR81 | **2228** | ITGA8 | **4257** | POU6F1 | **6286** | KCTD18 |
| 200 | LARGE1 | **2229** | LPCAT1 | **4258** | HMGB1 | **6287** | PRELID2 |
| 201 | MIDEAS | **2230** | RNF8 | **4259** | KMO | **6288** | COPS2 |
| 202 | RNPC3 | **2231** | ATXN7 | **4260** | CSNK1A1 | **6289** | ZMAT1 |
| 203 | VPS36 | **2232** | CREB5 | **4261** | HMGCL | **6290** | IFITM3 |
| 204 | GAPDHS | **2233** | VTI1B | **4262** | DUSP16 | **6291** | MOB3A |
| 205 | NAB1 | **2234** | PCNX3 | **4263** | SEMA4A | **6292** | VAMP7 |
| 206 | UBQLN2 | **2235** | TBRG4 | **4264** | GALNT3 | **6293** | PTCD3 |
| 207 | SLC25A38 | **2236** | POLR1B | **4265** | NIBAN3 | **6294** | U2AF2 |
| 208 | ANKRD10 | **2237** | GALNT12 | **4266** | LRRTM2 | **6295** | PTK2B |
| 209 | MROH5 | **2238** | HMCES | **4267** | NPEPL1 | **6296** | RELA |
| 210 | PTMA | **2239** | PTMAP7 | **4268** | CYP27A1 | **6297** | DEK |
| 211 | MRPS9 | **2240** | RIC8B | **4269** | FBXO17 | **6298** | FUBP1 |
| 212 | NFATC1 | **2241** | RPS21 | **4270** | INO80D | **6299** | TNRC6C |
| 213 | MAB21L2 | **2242** | LMF1 | **4271** | UQCR11 | **6300** | LILRA5 |
| 214 | SON | **2243** | CDR2 | **4272** | CABLES1 | **6301** | DHX34 |
| 215 | DNAAF3 | **2244** | BTBD6 | **4273** | YPEL1 | **6302** | MORF4L2 |
| 216 | EIF2S2 | **2245** | LETM2 | **4274** | BCL9 | **6303** | AMIGO1 |
| 217 | RBM8A | **2246** | RBM8 | **4275** | GMEB2 | **6304** | TTC5 |
| 218 | REV1 | **2247** | CPSF6 | **4276** | NLE1 | **6305** | TMEM258 |
| 219 | GIT2 | **2248** | TLE1 | **4277** | STK25 | **6306** | RPS15 |
| 220 | PLSCR3 | **2249** | MGME1 | **4278** | DIDO1 | **6307** | UBE2A |
| 221 | ORC5 | **2250** | TPT1 | **4279** | DST | **6308** | TUBGCP6 |
| 222 | ARHGAP4 | **2251** | NIN | **4280** | VPS35 | **6309** | PRPSAP1 |
| 223 | PTPRC | **2252** | PEG10 | **4281** | ID3 | **6310** | GON4L |
| 224 | EEF1G | **2253** | FEM1A | **4282** | HCRT | **6311** | PPOX |
| 225 | TFG | **2254** | MMGT1 | **4283** | NEURL1 | **6312** | RSBN1 |
| 226 | VPS16 | **2255** | RPAP1 | **4284** | TMEM150B | **6313** | RAB39A |
| 227 | RAB34 | **2256** | PIK3CB | **4285** | NDUFB11 | **6314** | KLHL9 |
| 228 | COA5 | **2257** | SIRT7 | **4286** | BRPF3 | **6315** | PSD2 |
| 229 | ARHGEF2 | **2258** | PHF8 | **4287** | TRIM68 | **6316** | RNF145 |
| 230 | AGAP3 | **2259** | SUMO1 | **4288** | TANGO6 | **6317** | ARMC7 |
| 231 | MAP3K14 | **2260** | USP48 | **4289** | KLHL18 | **6318** | RINL |
| 232 | PHRF1 | **2261** | COL9A3 | **4290** | H2BC4 | **6319** | HIST1H2BE |
| 233 | HIST1H2BC | **2262** | GBGT1 | **4291** | EIF2AK4 | **6320** | CAPRIN1 |
| 234 | COL17A1 | **2263** | UBE2G1 | **4292** | NUP35 | **6321** | PLIN3 |
| 235 | SYAP1 | **2264** | VPS51 | **4293** | TPR | **6322** | SRP14 |
| 236 | PDE6B | **2265** | CERS5 | **4294** | CD81 | **6323** | RBM4B |
| 237 | IFI27 | **2266** | TMEM245 | **4295** | SNRPD2 | **6324** | LOC645339 |
| 238 | POLR3E | **2267** | ZMYND15 | **4296** | WNK1 | **6325** | DTX2 |
| 239 | ALS2CL | **2268** | TPST2 | **4297** | KLHL30 | **6326** | DICER1 |
| 240 | SLC26A1 | **2269** | ESRRA | **4298** | KCNJ2 | **6327** | DOCK8 |
| 241 | TLR2 | **2270** | PGAM5 | **4299** | MTPAP | **6328** | SEC24B |
| 242 | NAP1L2 | **2271** | SFT2D1 | **4300** | GABRR2 | **6329** | CWC15 |
| 243 | CLCN6 | **2272** | RUNX3 | **4301** | CLIP1 | **6330** | DCP1B |
| 244 | MKS1 | **2273** | NAA25 | **4302** | CLIC3 | **6331** | ST7 |
| 245 | LRP12 | **2274** | FAM4A1 | **4303** | CAPZA1 | **6332** | DIABLO |
| 246 | GOPC | **2275** | MRPL42 | **4304** | EIF2D | **6333** | ATP5F1E |
| 247 | COG1 | **2276** | POLR1D | **4305** | SCRIB | **6334** | CRYBG2 |
| 248 | PDZD4 | **2277** | CTIF | **4306** | CCDC167 | **6335** | FUT7 |
| 249 | APC2 | **2278** | APOC2 | **4307** | SLC25A23 | **6336** | ANAPC2 |
| 250 | MIER2 | **2279** | CRY2 | **4308** | STX7 | **6337** | PPP3CA |
| 251 | NCL | **2280** | ANKRD52 | **4309** | ITGA10 | **6338** | ACAD9 |
| 252 | FAM98B | **2281** | INPP5B | **4310** | BIRC6 | **6339** | CCDC186 |
| 253 | ABHD16A | **2282** | COPB2 | **4311** | AHCTF1 | **6340** | MLLT10 |
| 254 | SRSF4 | **2283** | BBS9 | **4312** | VWA5B2 | **6341** | CCNB2 |
| 255 | LSM4 | **2284** | ADCK5 | **4313** | BCO2 | **6342** | DYRK4 |
| 256 | LRRK1 | **2285** | CHMP1B | **4314** | CCNI | **6343** | LSS |
| 257 | FAAP100 | **2286** | METTL17 | **4315** | COG5 | **6344** | SSR4 |
| 258 | KAT5 | **2287** | YTHDC1 | **4316** | RRM2B | **6345** | ATG101 |
| 259 | MYO18A | **2288** | LRWD1 | **4317** | DNHD1 | **6346** | NDUFV3 |
| 260 | ZMIZ1 | **2289** | CPEB3 | **4318** | P4HB | **6347** | OK/SW-cl.24 |
| 261 | SHPRH | **2290** | KIF21B | **4319** | SMC5 | **6348** | ZC3H7A |
| 262 | ACP5 | **2291** | OTUD4 | **4320** | KCTD16 | **6349** | NELFB |
| 263 | NADK | **2292** | OGT | **4321** | CIAO3 | **6350** | CEACAM16 |
| 264 | SMIM5 | **2293** | USPL1 | **4322** | CRIM1 | **6351** | LSM12 |
| 265 | PTPRCAP | **2294** | SAFB2 | **4323** | RAPGEF1 | **6352** | CARS2 |
| 266 | IPO9 | **2295** | SCIMP | **4324** | TMEM198 | **6353** | HOOK3 |
| 267 | PEX14 | **2296** | LIMK1 | **4325** | ASB2 | **6354** | CCRL2 |
| 268 | DCTN2 | **2297** | KRT10 | **4326** | MRPL20 | **6355** | ALYREF |
| 269 | DDX43 | **2298** | FAM120A | **4327** | SPTSSA | **6356** | HDLBP |
| 270 | PPM1H | **2299** | TRMT44 | **4328** | TASOR | **6357** | ACKR2 |
| 271 | SLC9A4 | **2300** | CHMP2A | **4329** | LYZL4 | **6358** | LYZA |
| 272 | TSSK2 | **2301** | CXCL1 | **4330** | CMTR1 | **6359** | EIF4G2 |
| 273 | AAG1 | **2302** | RPS6KA3 | **4331** | KRIT1 | **6360** | CCM1 |
| 274 | HSD17B4 | **2303** | SLCO2B1 | **4332** | GSDME | **6361** | CENPF |
| 275 | TANGO2 | **2304** | SRP72 | **4333** | HEL103 | **6362** | NKX2-1 |
| 276 | TTF1 | **2305** | NCS1 | **4334** | XRCC5 | **6363** | KATNB1 |
| 277 | GCN1 | **2306** | GCN1L1 | **4335** | TARS2 | **6364** | UGCG |
| 278 | SMYD5 | **2307** | MIEF2 | **4336** | CORT | **6365** | FHIP2A |
| 279 | COPS9 | **2308** | ATP2B1 | **4337** | FBXO7 | **6366** | SECISBP2 |
| 280 | RGL3 | **2309** | NIPA2 | **4338** | HES7 | **6367** | MYL12A |
| 281 | RASSF5 | **2310** | LAGE3 | **4339** | SMDT1 | **6368** | TACC1 |
| 282 | GGA3 | **2311** | IRF9 | **4340** | SKIC2 | **6369** | SKIV2L |
| 283 | SEM1 | **2312** | GALNT7 | **4341** | XKR6 | **6370** | EIF3F |
| 284 | IFP38 | **2313** | TBCB | **4342** | SDCBP | **6371** | PTBP3 |
| 285 | SSX2IP | **2314** | TMEM221 | **4343** | ZNF692 | **6372** | TRIM15 |
| 286 | TMEM253 | **2315** | NIBAN1 | **4344** | GPSM2 | **6373** | PTGER1 |
| 287 | QTRT2 | **2316** | PCDHGA1 | **4345** | TNNI3 | **6374** | NAT10 |
| 288 | STX16 | **2317** | ERBIN | **4346** | RPL38 | **6375** | SNX20 |
| 289 | GRSF1 | **2318** | GABBR1 | **4347** | BEX3 | **6376** | TMED5 |
| 290 | CYLD | **2319** | CHMP5 | **4348** | CALHM2 | **6377** | METTL22 |
| 291 | TRAK1 | **2320** | TET3 | **4349** | IL17C | **6378** | ANKRD53 |
| 292 | ACCS | **2321** | CDK14 | **4350** | LRRC14 | **6379** | ARCN1 |
| 293 | ALKBH3 | **2322** | EIF4E | **4351** | METAP2 | **6380** | PLEKHN1 |
| 294 | RPL7 | **2323** | SLC38A5 | **4352** | CALM2 | **6381** | CALM1 |
| 295 | CHTOP | **2324** | PARP8 | **4353** | CFDP1 | **6382** | FOXJ3 |
| 296 | LYSET | **2325** | HNRNPH2 | **4354** | ATP5MC2 | **6383** | JUNB |
| 297 | RPL30 | **2326** | UBR2 | **4355** | ANKRD13A | **6384** | ATF7IP |
| 298 | XPO5 | **2327** | FAM110B | **4356** | RCBTB1 | **6385** | CELF2 |
| 299 | SLC16A13 | **2328** | PLAG1 | **4357** | KDM2B | **6386** | RAB5C |
| 300 | IGF2BP3 | **2329** | RPL22 | **4358** | RSBN1L | **6387** | INTS12 |
| 301 | FAM118B | **2330** | IRF3 | **4359** | GPR152 | **6388** | TRMT11 |
| 302 | GLCCI1 | **2331** | ACP1 | **4360** | MOB2 | **6389** | PRKCB |
| 303 | PABPN1 | **2332** | TNFSF13B | **4361** | GSAP | **6390** | RAB31 |
| 304 | FOXN3 | **2333** | PMPCA | **4362** | PARVB | **6391** | SFXN2 |
| 305 | TBC1D25 | **2334** | XAB2 | **4363** | IL10RA | **6392** | OSTF1 |
| 306 | DHRS1 | **2335** | SMUG1 | **4364** | ERLN | **6393** | ATP5PB |
| 307 | RBBP8 | **2336** | TSACC | **4365** | DMTF1 | **6394** | FBXL4 |
| 308 | CABP4 | **2337** | NRAS | **4366** | GGH | **6395** | RHBDD2 |
| 309 | C2CD5 | **2338** | TRMT1L | **4367** | PLEKHM3 | **6396** | NDST1 |
| 310 | TMOD3 | **2339** | KCTD10 | **4368** | TLCD2 | **6397** | PNRC1 |
| 311 | RGL1 | **2340** | SYT3 | **4369** | EIF3L | **6398** | AGK |
| 312 | KCTD4 | **2341** | TNFAIP8L2 | **4370** | TAOK3 | **6399** | ATP2C1 |
| 313 | AASDH | **2342** | ERO1B | **4371** | TLR8 | **6400** | HLCS |
| 314 | MRPS21 | **2343** | ATP6V1D | **4372** | TOR1AIP2 | **6401** | PCNX2 |
| 315 | ZBTB49 | **2344** | TM4SF5 | **4373** | PPP1CB | **6402** | TMEM131L |
| 316 | ABR | **2345** | RBM14 | **4374** | ANKS3 | **6403** | DNAJC1 |
| 317 | IQSEC2 | **2346** | TEPSIN | **4375** | FER1L5 | **6404** | ZCCHC2 |
| 318 | TVP23B | **2347** | TVP23C-CDRT4 | **4376** | STRAP | **6405** | TMEM134 |
| 319 | NDUFA6 | **2348** | NUP50 | **4377** | USE1 | **6406** | RPL17 |
| 320 | TRAF1 | **2349** | NAA16 | **4378** | TMEM243 | **6407** | NEMP2 |
| 321 | FAM234B | **2350** | B4GALT3 | **4379** | HGS | **6408** | BST1 |
| 322 | HAGHL | **2351** | UBXN1 | **4380** | CORO7 | **6409** | CDK6 |
| 323 | ZC3H3 | **2352** | SIPA1L3 | **4381** | PAICS | **6410** | HEATR6 |
| 324 | NUTF2 | **2353** | GGNBP1 | **4382** | KMT2D | **6411** | MBD3 |
| 325 | IFFO1 | **2354** | GPKOW | **4383** | TLR4 | **6412** | SLC39A1 |
| 326 | LYPLA1 | **2355** | DUSP10 | **4384** | RABL6 | **6413** | SULF2 |
| 327 | COL15A1 | **2356** | ERCC4 | **4385** | IFT74 | **6414** | FBXO24 |
| 328 | TELO2 | **2357** | CTSZ | **4386** | AP1AR | **6415** | POP4 |
| 329 | NLRP2B | **2358** | ERCC2 | **4387** | ZSCAN26 | **6416** | FRA10AC1 |
| 330 | SH3GLB1 | **2359** | TAP2 | **4388** | SEC14L3 | **6417** | CPNE3 |
| 331 | IRF5 | **2360** | MYCBP2 | **4389** | SKAP2 | **6418** | HPS5 |
| 332 | LCOR | **2361** | BICRAL | **4390** | GNA11 | **6419** | SNAPC3 |
| 333 | COL20A1 | **2362** | CERS4 | **4391** | PDE7B | **6420** | PABPC4 |
| 334 | EXOSC5 | **2363** | DNAJA2 | **4392** | PTPN18 | **6421** | PHAX |
| 335 | PPIB | **2364** | AMPD2 | **4393** | DDX56 | **6422** | PNPLA6 |
| 336 | SRRM1 | **2365** | B4GALNT2 | **4394** | SCN2B | **6423** | RAD1 |
| 337 | RNASE6 | **2366** | POGLUT1 | **4395** | TSGA10IP | **6424** | HUS1B |
| 338 | TJP3 | **2367** | TALDO1 | **4396** | OMG | **6425** | CARD16 |
| 339 | COP1 | **2368** | GABARAP | **4397** | FBXO21 | **6426** | NDUFC1 |
| 340 | FAM118A | **2369** | MRPL54 | **4398** | AMPD3 | **6427** | DOP1A |
| 341 | DOPEY1 | **2370** | CD244 | **4399** | PSRC1 | **6428** | PLCG2 |
| 342 | HACE1 | **2371** | ATG9B | **4400** | DHX35 | **6429** | PRELID3A |
| 343 | AMN1 | **2372** | IZUMO4 | **4401** | CD276 | **6430** | ARAP1 |
| 344 | LCMT1 | **2373** | MRPL14 | **4402** | MRPL32 | **6431** | FAM193A |
| 345 | AMBRA1 | **2374** | SH2D3C | **4403** | ST7L | **6432** | PRMT3 |
| 346 | HNRNPA2B1 | **2375** | CHN2 | **4404** | BAG1 | **6433** | HACD2 |
| 347 | SH3PXD2A | **2376** | PRKCA | **4405** | PRKACA | **6434** | DENND2C |
| 348 | ACTR5 | **2377** | BTG1 | **4406** | GOLPH3 | **6435** | PSMC5 |
| 349 | LGMN | **2378** | SPIN4 | **4407** | ASF1A | **6436** | PSMC1 |
| 350 | LPCAT4 | **2379** | MBOAT2 | **4408** | ATRNL1 | **6437** | DENND6B |
| 351 | ANK1 | **2380** | TOR1B | **4409** | TRIM39 | **6438** | EXOSC10 |
| 352 | TMED2 | **2381** | RNP24 | **4410** | PARP10 | **6439** | MVB12B |
| 353 | PSMC6 | **2382** | NDUFB3 | **4411** | ALDH1A2 | **6440** | GPATCH2L |
| 354 | DOK2 | **2383** | SART3 | **4412** | EFCAB14 | **6441** | MTO1 |
| 355 | CLCN3 | **2384** | HJURP | **4413** | CENPB | **6442** | TRERF1 |
| 356 | LDAF1 | **2385** | RAB18 | **4414** | DPH5 | **6443** | FMNL1 |
| 357 | SEC61G | **2386** | RPS9 | **4415** | ATP5PD | **6444** | ATP5H |
| 358 | CDK18 | **2387** | ANKRD50 | **4416** | MYO1B | **6445** | EXOC6B |
| 359 | ENTPD6 | **2388** | MMP24 | **4417** | RPL5 | **6446** | SLC16A10 |
| 360 | SFXN4 | **2389** | EMP3 | **4418** | RPL10 | **6447** | MTDH |
| 361 | ALDH16A1 | **2390** | ZP2 | **4419** | BRAT1 | **6448** | ARHGAP39 |
| 362 | GPR35 | **2391** | FES | **4420** | PACRG | **6449** | NAB2 |
| 363 | DUSP12 | **2392** | GRAP2 | **4421** | CDKN2AIPNL | **6450** | EIF5A |
| 364 | EIF5A1 | **2393** | SH3BP2 | **4422** | CSNK2A1 | **6451** | ZFHX2 |
| 365 | CUL7 | **2394** | BCL3 | **4423** | PXMP4 | **6452** | HSBP1 |
| 366 | BRD1 | **2395** | AHR | **4424** | PNPLA8 | **6453** | PPFIBP2 |
| 367 | RPS13 | **2396** | UBE2V1 | **4425** | EEF2KMT | **6454** | NDUFB10 |
| 368 | SMIM18 | **2397** | INTS8 | **4426** | FAM181A | **6455** | PKHD1L1 |
| 369 | ATG4D | **2398** | SPG11 | **4427** | YIPF1 | **6456** | XPO4 |
| 370 | HNRNPR | **2399** | GPR68 | **4428** | RFX5 | **6457** | HMG20B |
| 371 | FBXO46 | **2400** | NCKAP1L | **4429** | PEX11G | **6458** | EMILIN1 |
| 372 | ANO8 | **2401** | PNISR | **4430** | STEAP3 | **6459** | TNFRSF12A |
| 373 | CCR1 | **2402** | GLOD4 | **4431** | FAM219B | **6460** | ABRACL |
| 374 | KDM3B | **2403** | MATN2 | **4432** | GPN1 | **6461** | GASK1B |
| 375 | FAM198B | **2404** | DNMBP | **4433** | SAAL1 | **6462** | H1-4 |
| 376 | HIST1H1E | **2405** | ERH | **4434** | SKP2 | **6463** | FOS |
| 377 | NAMPT | **2406** | ZBTB26 | **4435** | SMAD6 | **6464** | RAB1A |
| 378 | ATP6V1F | **2407** | TNFRSF21 | **4436** | EP300 | **6465** | MECP2 |
| 379 | SIGLEC1 | **2408** | OGG1 | **4437** | CNOT1 | **6466** | AP3S1 |
| 380 | MRPL30 | **2409** | PDGFB | **4438** | UBL3 | **6467** | DNASE1L1 |
| 381 | PPP4R1 | **2410** | WDR20 | **4439** | DKFZp686P0251 | **6468** | BEND4 |
| 382 | SOCS3 | **2411** | LRRC59 | **4440** | TAPBPL | **6469** | MST1 |
| 383 | STK4 | **2412** | TXNIP | **4441** | SLC35E2A | **6470** | RND3 |
| 384 | VIM | **2413** | HEL113 | **4442** | KRT80 | **6471** | LRCH3 |
| 385 | CPQ | **2414** | ST3GAL1 | **4443** | PRKAG3 | **6472** | RAC3 |
| 386 | CPNE8 | **2415** | COL10A1 | **4444** | GADD45A | **6473** | UHMK1 |
| 387 | TMEM129 | **2416** | ACOT11 | **4445** | ISCU | **6474** | PRR33 |
| 388 | CDKAL1 | **2417** | SETD3 | **4446** | CLCF1 | **6475** | TAF8 |
| 389 | DEPDC5 | **2418** | CAPZB | **4447** | SUGT1 | **6476** | EIF2B2 |
| 390 | ZDHHC8 | **2419** | ATXN7L1 | **4448** | BCORL1 | **6477** | CXCL2 |
| 391 | VPS13D | **2420** | LRIG2 | **4449** | REST | **6478** | AKAP13 |
| 392 | CDKN2C | **2421** | CBFA2T3 | **4450** | MRPS14 | **6479** | LAMTOR1 |
| 393 | NADSYN1 | **2422** | LUC7L3 | **4451** | FNDC9 | **6480** | GBP7 |
| 394 | RARA | **2423** | NR1B1 | **4452** | RPS27A | **6481** | RRAGB |
| 395 | ATP5F1C | **2424** | SOX30 | **4453** | TOMM22 | **6482** | PSMD10 |
| 396 | LAPTM5 | **2425** | EXD2 | **4454** | RHOF | **6483** | CREG2 |
| 397 | IFT140 | **2426** | MDN1 | **4455** | FUZ | **6484** | SZRD1 |
| 398 | ZFP37 | **2427** | TM9SF3 | **4456** | ZBTB48 | **6485** | TLN1 |
| 399 | WDR5B | **2428** | NFE2L2 | **4457** | KEL | **6486** | UQCRQ |
| 400 | RPL10A | **2429** | P2RY2 | **4458** | RPLP1 | **6487** | RRP1 |
| 401 | IDH3B | **2430** | HS3ST1 | **4459** | ZHX1 | **6488** | ZUP1 |
| 402 | ZUFSP | **2431** | IL33 | **4460** | TAGLN2 | **6489** | LUC7L |
| 403 | PTBP1 | **2432** | GPR153 | **4461** | SLAMF9 | **6490** | SNX3 |
| 404 | SLC15A4 | **2433** | PDCD6 | **4462** | ALG2 | **6491** | ARL4C |
| 405 | NPC2 | **2434** | CASC3 | **4463** | BORCS5 | **6492** | PLCL2 |
| 406 | NAXD | **2435** | GOLIM4 | **4464** | DCAF17 | **6493** | IL1RL1 |
| 407 | ANKRD11 | **2436** | STARD9 | **4465** | ANXA1 | **6494** | MMP12 |
| 408 | CYBB | **2437** | ACSL1 | **4466** | SEC16A | **6495** | PIDD1 |
| 409 | DCDC2B | **2438** | FBRSL1 | **4467** | SENP1 | **6496** | AFAP1L2 |
| 410 | URB1 | **2439** | FGD2 | **4468** | ELOC | **6497** | DNASE1 |
| 411 | SPPL2A | **2440** | IMP3 | **4469** | TTLL5 | **6498** | PSMD8 |
| 412 | CASP3 | **2441** | ASCL2 | **4470** | PLXNA4 | **6499** | NDUFAF6 |
| 413 | RPL3 | **2442** | PSMA7 | **4471** | CLN6 | **6500** | SNW1 |
| 414 | RAD21 | **2443** | DNAJB9 | **4472** | SEMA6B | **6501** | SLC35B3 |
| 415 | RPL36 | **2444** | ADAM33 | **4473** | TMEM91 | **6502** | SH3KBP1 |
| 416 | PHTF2 | **2445** | WWOX | **4474** | PVR | **6503** | ABHD5 |
| 417 | TAF9B | **2446** | RGS19 | **4475** | DYNLRB1 | **6504** | MFSD1 |
| 418 | SLC28A2 | **2447** | DDX17 | **4476** | HNRNPC | **6505** | DHX33 |
| 419 | SELENOF | **2448** | IDNK | **4477** | DENND10 | **6506** | C8G |
| 420 | USP4 | **2449** | SLFN14 | **4478** | CUL4B | **6507** | LPP |
| 421 | CABIN1 | **2450** | IST1 | **4479** | ZMYND11 | **6508** | PRR5 |
| 422 | USP22 | **2451** | AFDN | **4480** | AMZ1 | **6509** | KIAA1950 |
| 423 | SLC3A2 | **2452** | RTTN | **4481** | ATP13A1 | **6510** | ITPK1 |
| 424 | KCNK3 | **2453** | PIAS3 | **4482** | DKFZp434O0617 | **6511** | ING4 |
| 425 | DNAH2 | **2454** | MSR1 | **4483** | NDUFA7 | **6512** | SNAPC5 |
| 426 | ARHGAP27 | **2455** | KANK2 | **4484** | PKN3 | **6513** | GNA15 |
| 427 | RABGGTB | **2456** | TRAF7 | **4485** | TRIM7 | **6514** | RAMAC |
| 428 | DOK3 | **2457** | GARNL3 | **4486** | EXOC8 | **6515** | FPGT |
| 429 | ROMO1 | **2458** | MED13L | **4487** | PFDN1 | **6516** | IGHD |
| 430 | RPS15A | **2459** | NDUFA12 | **4488** | CSTB | **6517** | TMEM161A |
| 431 | PLA2G4B | **2460** | ANAPC16 | **4489** | S100A9 | **6518** | POLG |
| 432 | RALGAPA1 | **2461** | PHIP | **4490** | WDR11 | **6519** | UBE2M |
| 433 | LRRC3 | **2462** | ZKSCAN3 | **4491** | C1QTNF6 | **6520** | COX7A2L |
| 434 | SCAF1 | **2463** | PRDM10 | **4492** | PTPRE | **6521** | ERGIC1 |
| 435 | PKIA | **2464** | TBC1D10B | **4493** | LRRC4 | **6522** | SESTD1 |
| 436 | ZCCHC8 | **2465** | ADSS2 | **4494** | ADSS | **6523** | DUSP11 |
| 437 | CLCC1 | **2466** | MRPL18 | **4495** | SESN2 | **6524** | WRAP73 |
| 438 | SMIM15 | **2467** | POGK | **4496** | ANKRD44 | **6525** | ATP5F1D |
| 439 | INPP5E | **2468** | CCPG1 | **4497** | LGI4 | **6526** | PNO1 |
| 440 | CLEC7A | **2469** | SGK1 | **4498** | SMARCA5 | **6527** | IK |
| 441 | EPS8 | **2470** | DNMT3A | **4499** | LTO1 | **6528** | VAV3 |
| 442 | ACBD6 | **2471** | SNRNP25 | **4500** | NDUFB8 | **6529** | ACSS1 |
| 443 | KPNA2 | **2472** | PPP1R12B | **4501** | RAB19 | **6530** | CASP9 |
| 444 | ZFP90 | **2473** | CTDSPL | **4502** | FOXRED2 | **6531** | SEMA4C |
| 445 | ADORA3 | **2474** | MRPS23 | **4503** | PLEKHA2 | **6532** | CAVIN4 |
| 446 | ADAM23 | **2475** | ADAM28 | **4504** | METTL2A | **6533** | RHOG |
| 447 | ARHG | **2476** | NDUFA10 | **4505** | OLFM1 | **6534** | AFG2B |
| 448 | ABITRAM | **2477** | ITPA | **4506** | SNX17 | **6535** | DNAJC11 |
| 449 | MBP | **2478** | PRG2 | **4507** | RBM6 | **6536** | DUSP23 |
| 450 | COX4I1 | **2479** | GOLGA5 | **4508** | SLC25A5 | **6537** | ACSL3 |
| 451 | MLLT3 | **2480** | C1RL | **4509** | TIMP1 | **6538** | SYTL1 |
| 452 | NSUN4 | **2481** | TXNL1 | **4510** | HEL-S-114 | **6539** | BANP |
| 453 | TRIM47 | **2482** | HADHA | **4511** | RGS1 | **6540** | RSPH10B |
| 454 | TICAM1 | **2483** | ANKRD61 | **4512** | LPAR5 | **6541** | KPG_010 |
| 455 | TREML2 | **2484** | MYEF2 | **4513** | SERGEF | **6542** | PCSK1 |
| 456 | TMEM256 | **2485** | TRIM56 | **4514** | METTL25B | **6543** | ECI2 |
| 457 | CHP1 | **2486** | CHORDC1 | **4515** | PSMA1 | **6544** | COQ6 |
| 458 | MGAM | **2487** | MGA | **4516** | LYRM9 | **6545** | AP1S1 |
| 459 | CNTROB | **2488** | GALNS | **4517** | TRPV2 | **6546** | CWF19L1 |
| 460 | MLLT11 | **2489** | AF1Q | **4518** | H2AX | **6547** | REC114 |
| 461 | TTI2 | **2490** | SUMO3 | **4519** | RMND5A | **6548** | VCP |
| 462 | CDC25B | **2491** | ERCC8 | **4520** | PLA2G7 | **6549** | RAB27A |
| 463 | ARAF | **2492** | RGS14 | **4521** | ZNF445 | **6550** | PPAN |
| 464 | UNKL | **2493** | OTUD1 | **4522** | CLTC | **6551** | PTPRA |
| 465 | ARVCF | **2494** | LRRC28 | **4523** | TSPOAP1 | **6552** | RBP1 |
| 466 | ARID4A | **2495** | CARM1 | **4524** | PRDX2 | **6553** | HEL-S-2a |
| 467 | KIF26B | **2496** | IFRD1 | **4525** | SLC25A17 | **6554** | NUP93 |
| 468 | NETO2 | **2497** | MYBPH | **4526** | GALNT10 | **6555** | OSBPL10 |
| 469 | GRN | **2498** | COX5B | **4527** | ID2 | **6556** | TUBB |
| 470 | GCC1 | **2499** | SERPINA2 | **4528** | ATR | **6557** | ANTXR1 |
| 471 | AP2S1 | **2500** | BCOR | **4529** | FURIN | **6558** | FAM222A |
| 472 | H2AZ2 | **2501** | CCR10 | **4530** | ZKSCAN4 | **6559** | UFC1 |
| 473 | SLC22A17 | **2502** | FUT4 | **4531** | ABRAXAS1 | **6560** | BET1 |
| 474 | SLC35D2 | **2503** | PPP1R3C | **4532** | PPARG | **6561** | PPARG1D5 |
| 475 | NR1C3 | **2504** | S100PBP | **4533** | WBP4 | **6562** | ZBED3 |
| 476 | INO80 | **2505** | EIF4A1 | **4534** | PTCD2 | **6563** | MBD4 |
| 477 | MED1 | **2506** | LEMD3 | **4535** | MED12 | **6564** | TNRC11 |
| 478 | CYRIA | **2507** | CR1L | **4536** | CAVIN3 | **6565** | EEF1AKMT2 |
| 479 | MFSD10 | **2508** | EIF3K | **4537** | TMIGD3 | **6566** | PDE1B |
| 480 | TRAF3 | **2509** | WDR4 | **4538** | RPS19 | **6567** | MPPE1 |
| 481 | AP3D1 | **2510** | ANAPC13 | **4539** | GLRX5 | **6568** | TSPAN5 |
| 482 | AVPR2 | **2511** | CRKL | **4540** | IMPDH2 | **6569** | SLC1A5 |
| 483 | RPS7 | **2512** | PHF12 | **4541** | MARK2 | **6570** | CEP85L |
| 484 | BRI3BP | **2513** | ABCF3 | **4542** | ITFG2 | **6571** | CCDC92B |
| 485 | HIP1 | **2514** | TSEN54 | **4543** | JAK2 | **6572** | PIGM |
| 486 | NBEAL1 | **2515** | LBR | **4544** | DOCK11 | **6573** | PSMD6 |
| 487 | ACP2 | **2516** | GINS3 | **4545** | HPS3 | **6574** | DEXI |
| 488 | POLR3GL | **2517** | PCMT1 | **4546** | CEP152 | **6575** | DUSP4 |
| 489 | LAMTOR2 | **2518** | RRAS2 | **4547** | TUBGCP4 | **6576** | FAM162A |
| 490 | PPIA | **2519** | SYNRG | **4548** | DNAJC14 | **6577** | FASTKD2 |
| 491 | OSBPL11 | **2520** | CNIH2 | **4549** | ALG8 | **6578** | LYSMD3 |
| 492 | ZBTB18 | **2521** | CGGBP1 | **4550** | MERTK | **6579** | HNRNPL |
| 493 | EMC8 | **2522** | AKT3 | **4551** | ARID5A | **6580** | LDLRAD4 |
| 494 | ARF4 | **2523** | SMAD1 | **4552** | GPR18 | **6581** | ARF1 |
| 495 | MDFIC | **2524** | CD84 | **4553** | H6PD | **6582** | IRF2BP1 |
| 496 | TRAF2 | **2525** | TANK | **4554** | CKAP4 | **6583** | TMEM38B |
| 497 | PDLIM2 | **2526** | CCDC188 | **4555** | FLNA | **6584** | FARSA |
| 498 | INTS13 | **2527** | IER5L | **4556** | ARSI | **6585** | DYNC2I2 |
| 499 | CIAO2A | **2528** | SELENON | **4557** | SMIM24 | **6586** | NINJ2 |
| 500 | RIN3 | **2529** | IL1RN | **4558** | SMAD7 | **6587** | CKS1B |
| 501 | PIM1 | **2530** | GTF2B | **4559** | BTN2A2 | **6588** | PSMC4 |
| 502 | ATP6V0D1 | **2531** | CDC34 | **4560** | P2RY12 | **6589** | NUP98 |
| 503 | ABCC10 | **2532** | TBCK | **4561** | MIIP | **6590** | SQSTM1 |
| 504 | AFG1L | **2533** | FARS2 | **4562** | PRKCE | **6591** | TAF4 |
| 505 | IAH1 | **2534** | RPL22L1 | **4563** | GART | **6592** | UBAP2 |
| 506 | PLSCR1 | **2535** | SF3B2 | **4564** | SCPEP1 | **6593** | RPS6KC1 |
| 507 | CEP55 | **2536** | PPP1R21 | **4565** | TCTA | **6594** | MIEN1 |
| 508 | RLIM | **2537** | TBC1D5 | **4566** | TRAPPC5 | **6595** | TNPO2 |
| 509 | ATP5PO | **2538** | NAV2 | **4567** | MGAT4A | **6596** | NFKB2 |
| 510 | SLAMF7 | **2539** | PEX6 | **4568** | EIF4E3 | **6597** | SLC31A1 |
| 511 | SH3BP1 | **2540** | CD27 | **4569** | CSTF2 | **6598** | SPIRE1 |
| 512 | SCAF4 | **2541** | HPCAL1 | **4570** | CD53 | **6599** | SSR3 |
| 513 | TRIT1 | **2542** | ASGR2 | **4571** | EBPL | **6600** | SSBP4 |
| 514 | SETD4 | **2543** | ARHGAP30 | **4572** | TUBB6 | **6601** | NARS1 |
| 515 | XRCC1 | **2544** | LGALS9 | **4573** | DYNLT3 | **6602** | ANGEL1 |
| 516 | PDCL | **2545** | ANKRD65 | **4574** | GNRH1 | **6603** | SINHCAF |
| 517 | ADNP | **2546** | DNASE1L2 | **4575** | GFUS | **6604** | DCP1A |
| 518 | PWWP2A | **2547** | ARID5B | **4576** | NUCKS1 | **6605** | RNF130 |
| 519 | NLRX1 | **2548** | BAD | **4577** | AATF | **6606** | RALBP1 |
| 520 | EIF3E | **2549** | KRT222 | **4578** | PFKFB4 | **6607** | NUP214 |
| 521 | PCED1B | **2550** | LIFR | **4579** | MRPL34 | **6608** | TMEM241 |
| 522 | CANX | **2551** | ITPRIPL2 | **4580** | TRAK2 | **6609** | PAXBP1 |
| 523 | RPS23 | **2552** | CNIH1 | **4581** | DRAP1 | **6610** | PPP1R3E |
| 524 | ZDHHC17 | **2553** | YPEL5 | **4582** | IVD | **6611** | SACM1L |
| 525 | TAS1R3 | **2554** | TTI1 | **4583** | QRFP | **6612** | RPLP2 |
| 526 | INKA1 | **2555** | WAPL | **4584** | ST3GAL3 | **6613** | ZBTB3 |
| 527 | ZDHHC23 | **2556** | APRT | **4585** | TNIP1 | **6614** | RPS18 |
| 528 | ZNF276 | **2557** | KRBA1 | **4586** | PHKA2 | **6615** | DPF2 |
| 529 | LCP1 | **2558** | BLTP3A | **4587** | UHRF1BP1 | **6616** | PES1 |
| 530 | SMIM13 | **2559** | TMCO6 | **4588** | SEC22C | **6617** | COPB1 |
| 531 | PRDX5 | **2560** | COX6C | **4589** | TPPP | **6618** | FREP1 |
| 532 | CETN2 | **2561** | SS18 | **4590** | SKP1 | **6619** | POC1B |
| 533 | VIPAS39 | **2562** | UBP1 | **4591** | SYNE1 | **6620** | FAM114A2 |
| 534 | GDI2 | **2563** | ARMC8 | **4592** | ELP1 | **6621** | IKBKAP |
| 535 | NDUFB9 | **2564** | SIK2 | **4593** | PEX16 | **6622** | UST |
| 536 | MAPRE1 | **2565** | USP10 | **4594** | USP32 | **6623** | NRIP3 |
| 537 | SPAM1 | **2566** | SPCS2 | **4595** | TMEM209 | **6624** | PRDX6 |
| 538 | FLVCR1 | **2567** | FOXJ2 | **4596** | RAB43 | **6625** | RHBDD1 |
| 539 | DPEP2 | **2568** | FPGS | **4597** | COG3 | **6626** | RHOBTB3 |
| 540 | SLC25A3 | **2569** | DHRS3 | **4598** | SRC | **6627** | APOE |
| 541 | SLC12A7 | **2570** | PDE6G | **4599** | ADM | **6628** | LOXL3 |
| 542 | RNF180 | **2571** | ART4 | **4600** | DOK1 | **6629** | TNS4 |
| 543 | RASL11B | **2572** | SEPTIN8 | **4601** | 8-Sep | **6630** | SOD2 |
| 544 | EIF3I | **2573** | EIF3S2 | **4602** | ETF1 | **6631** | ARHGAP6 |
| 545 | TBC1D15 | **2574** | CHD6 | **4603** | PRAG1 | **6632** | CCDC124 |
| 546 | RAB7A | **2575** | RPL35 | **4604** | KIF5B | **6633** | TMCO1 |
| 547 | MTG1 | **2576** | LIMD1 | **4605** | GRK2 | **6634** | TMCC2 |
| 548 | OSGEPL1 | **2577** | TRADD | **4606** | FLRT2 | **6635** | R3HDM1 |
| 549 | BBIP1 | **2578** | PIGB | **4607** | PTP4A3 | **6636** | C1QC |
| 550 | ADIB | **2579** | POLR2I | **4608** | SCMH1 | **6637** | FMNL3 |
| 551 | PIGO | **2580** | CDC14A | **4609** | DYNLL2 | **6638** | TRMT61A |
| 552 | MAG | **2581** | CPD | **4610** | TTC14 | **6639** | PHF23 |
| 553 | ZFYVE9 | **2582** | GTF2H2 | **4611** | GTF2H2C_2 | **6640** | GTF2H2C |
| 554 | PPP2R2D | **2583** | MAST3 | **4612** | ELOF1 | **6641** | NEPRO |
| 555 | UFD1 | **2584** | UFD1L | **4613** | PRAP1 | **6642** | SCYL3 |
| 556 | LRRC55 | **2585** | LRRC49 | **4614** | RPS6KA5 | **6643** | ZBTB39 |
| 557 | ADGRE1 | **2586** | C2CD3 | **4615** | RBM19 | **6644** | CKAP5 |
| 558 | POR | **2587** | DKFZp686G04235 | **4616** | TYW3 | **6645** | TMEM229B |
| 559 | ABI3 | **2588** | PHLDB1 | **4617** | MS4A3 | **6646** | MAP2K1 |
| 560 | RAB2A | **2589** | IFRD2 | **4618** | PRELID1 | **6647** | IPO8 |
| 561 | KDELR1 | **2590** | H2AZ1 | **4619** | JARID2 | **6648** | ROCK2 |
| 562 | NDUFA13 | **2591** | SGK3 | **4620** | TICAM2 | **6649** | GMFG |
| 563 | NIF3L1 | **2592** | MS4A1 | **4621** | PTBP2 | **6650** | METTL16 |
| 564 | PHF14 | **2593** | PDXK | **4622** | PRPF38B | **6651** | DKFZp434O1172 |
| 565 | TOMM5 | **2594** | ING5 | **4623** | CAPN7 | **6652** | TRPT1 |
| 566 | RNF20 | **2595** | RERE | **4624** | ELP5 | **6653** | BLMH |
| 567 | FOCAD | **2596** | RBPJ | **4625** | MEMO1 | **6654** | WHAMM |
| 568 | PRADC1 | **2597** | SLC41A3 | **4626** | IRAG2 | **6655** | TBCA |
| 569 | TATDN3 | **2598** | DYRK2 | **4627** | PTPRJ | **6656** | MARCKSL1 |
| 570 | DLG1 | **2599** | EHBP1 | **4628** | PDLIM5 | **6657** | LONP2 |
| 571 | RETREG1 | **2600** | ALG6 | **4629** | ADAM19 | **6658** | CDK2AP2 |
| 572 | DOC-1R | **2601** | ID1 | **4630** | UBB | **6659** | DSEL |
| 573 | RPL32 | **2602** | MCMBP | **4631** | EEIG2 | **6660** | TUBGCP5 |
| 574 | EDF1 | **2603** | WBP11 | **4632** | RAB14 | **6661** | DHFR |
| 575 | DYR | **2604** | SMNDC1 | **4633** | EXOC1 | **6662** | MFAP3 |
| 576 | IL17RA | **2605** | NEK7 | **4634** | IARS1 | **6663** | IARS |
| 577 | ACTR8 | **2606** | MKI67 | **4635** | TCF12 | **6664** | PGK1 |
| 578 | PPP5C | **2607** | KALRN | **4636** | ANKFY1 | **6665** | ACAP2 |
| 579 | FIGN | **2608** | PLXNA1 | **4637** | PMS1 | **6666** | INCENP |
| 580 | TRIM26 | **2609** | PEBP1 | **4638** | GLYR1 | **6667** | MAX |
| 581 | LHFPL6 | **2610** | HTR2B | **4639** | PIP4K2B | **6668** | NDUFV2 |
| 582 | PDCD4 | **2611** | ABCB10 | **4640** | CALCRL | **6669** | PTGS2 |
| 583 | TRPC4AP | **2612** | MVB12A | **4641** | CCDC25 | **6670** | NDUFS4 |
| 584 | DDR2 | **2613** | HTR7 | **4642** | RNASE4 | **6671** | VMP1 |
| 585 | PSMB2 | **2614** | DNAJC28 | **4643** | ATP5PF | **6672** | ATP5J |
| 586 | TMEM242 | **2615** | TK2 | **4644** | KIF2C | **6673** | NOP53 |
| 587 | NUPR2 | **2616** | POU2F2 | **4645** | TTC39C | **6674** | TMEM222 |
| 588 | NXPE3 | **2617** | PGAP3 | **4646** | OXR1 | **6675** | CNPY4 |
| 589 | VPS33B | **2618** | SPTLC2 | **4647** | AHCYL2 | **6676** | ZMIZ2 |
| 590 | RANBP2 | **2619** | IL27RA | **4648** | SNX29 | **6677** | UNC119 |
| 591 | CCDC166 | **2620** | CRTC1 | **4649** | SPATS2L | **6678** | ATP6V1B2 |
| 592 | RBM33 | **2621** | RXRB | **4650** | GPR84 | **6679** | DHODH |
| 593 | SLC25A45 | **2622** | STAT3 | **4651** | SRSF11 | **6680** | RNF215 |
| 594 | DDX20 | **2623** | TMEM175 | **4652** | ATP6AP2 | **6681** | MMS19 |
| 595 | MEA1 | **2624** | SALL2 | **4653** | ARL6IP4 | **6682** | DDX27 |
| 596 | GMEB1 | **2625** | LGALS1 | **4654** | HTATSF1 | **6683** | SLC13A3 |
| 597 | PAIP2 | **2626** | RMI1 | **4655** | EML1 | **6684** | SIRT5 |
| 598 | DIMT1 | **2627** | PRXL2B | **4656** | RIPK1 | **6685** | CHAF1B |
| 599 | MPP7 | **2628** | USP11 | **4657** | LTN1 | **6686** | ADCY7 |
| 600 | CRAT | **2629** | CUEDC2 | **4658** | BSCL2 | **6687** | ELAVL4 |
| 601 | ZBTB32 | **2630** | CISH | **4659** | EPOR | **6688** | ITGA2 |
| 602 | VPS33A | **2631** | MCM3AP | **4660** | RAB3GAP1 | **6689** | DOCK6 |
| 603 | ERCC1 | **2632** | SRPK1 | **4661** | ANGPTL1 | **6690** | COQ10B |
| 604 | IQGAP2 | **2633** | CD37 | **4662** | RAB5A | **6691** | DNAJC10 |
| 605 | ANAPC1 | **2634** | EIF3C | **4663** | PCGF5 | **6692** | BMF |
| 606 | NELFE | **2635** | SAE1 | **4664** | HTRA3 | **6693** | ITM2B |
| 607 | PCED1A | **2636** | CDH11 | **4665** | NDRG4 | **6694** | ANGPT4 |
| 608 | BIN1 | **2637** | SSB | **4666** | HNRNPUL1 | **6695** | DCTPP1 |
| 609 | PANK2 | **2638** | AMIGO3 | **4667** | RWDD4 | **6696** | PREX1 |
| 610 | APOLD1 | **2639** | TNFAIP8L1 | **4668** | CEP19 | **6697** | IER3 |
| 611 | PRG1 | **2640** | PADI2 | **4669** | MPLKIP | **6698** | PIP4K2A |
| 612 | EMD | **2641** | OMD | **4670** | SH2D7 | **6699** | DISP1 |
| 613 | PGD | **2642** | ACVRL1 | **4671** | GPR22 | **6700** | DONSON |
| 614 | MAP1LC3A | **2643** | OPRM1 | **4672** | BHLHA9 | **6701** | GRIPAP1 |
| 615 | FNDC8 | **2644** | SMYD1 | **4673** | ATPSCKMT | **6702** | RGS2 |
| 616 | ABCD1 | **2645** | NAE1 | **4674** | PAPOLG | **6703** | SPINT1 |
| 617 | DPY30 | **2646** | SDHC | **4675** | ATAD3A | **6704** | CCDC78 |
| 618 | DCAF5 | **2647** | CYP2R1 | **4676** | MAPK3 | **6705** | UBE2E3 |
| 619 | APCDD1 | **2648** | GGT7 | **4677** | SHARPIN | **6706** | YBX3 |
| 620 | CDC14B | **2649** | HMBS | **4678** | BGN | **6707** | RBCK1 |
| 621 | SAR1B | **2650** | KIZ | **4679** | SERPIND1 | **6708** | PURB |
| 622 | FRAT2 | **2651** | FANCF | **4680** | UBL7 | **6709** | GALM |
| 623 | RNF121 | **2652** | DPM3 | **4681** | JRK | **6710** | PPP1R15B |
| 624 | NOVA1 | **2653** | GDPGP1 | **4682** | TMEM260 | **6711** | SLC26A10P |
| 625 | AGBL5 | **2654** | ARL3 | **4683** | STXBP1 | **6712** | ALKBH2 |
| 626 | TECPR2 | **2655** | ATP6V1G1 | **4684** | SELENOP | **6713** | TPM4 |
| 627 | RPL27 | **2656** | TSEN15 | **4685** | PDCD2L | **6714** | DNCL1 |
| 628 | VPS41 | **2657** | RAD9A | **4686** | UQCC2 | **6715** | ADAP1 |
| 629 | TGFBR2 | **2658** | SCYL2 | **4687** | HMGN2 | **6716** | TMEM262 |
| 630 | ACVR2B | **2659** | DNAJC15 | **4688** | MEF2C | **6717** | PMVK |
| 631 | ATP6V1A | **2660** | CCDC158 | **4689** | WASHC4 | **6718** | CKAP2 |
| 632 | ACVR1B | **2661** | NOS1AP | **4690** | JMY | **6719** | CAPNS1 |
| 633 | CRIPT | **2662** | GCNT7 | **4691** | TMEM71 | **6720** | WDR77 |
| 634 | ZMYM6 | **2663** | PSD3 | **4692** | AK3 | **6721** | AK6 |
| 635 | DPAGT1 | **2664** | BNIP3L | **4693** | PIM3 | **6722** | NRP1 |
| 636 | NELL1 | **2665** | MYH10 | **4694** | PSMB6 | **6723** | BAMBI |
| 637 | SNX2 | **2666** | RILPL2 | **4695** | CAPG | **6724** | NCAPG |
| 638 | LGI2 | **2667** | PLIN2 | **4696** | COPS6 | **6725** | TMED7 |
| 639 | COX7B | **2668** | EPB41 | **4697** | MRFAP1 | **6726** | MAP4K3 |
| 640 | TIMM13 | **2669** | ADSL | **4698** | HINT3 | **6727** | SUCLA2 |
| 641 | NR2F6 | **2670** | CENPV | **4699** | RPL26 | **6728** | STBD1 |
| 642 | IL4R | **2671** | UBE2C | **4700** | RECQL | **6729** | MTA3 |
| 643 | CRLF3 | **2672** | HDGFL3 | **4701** | RPS6KA2 | **6730** | RIC3 |
| 644 | KTN1 | **2673** | CHCHD3 | **4702** | SLC7A11 | **6731** | S100A10 |
| 645 | UQCR10 | **2674** | TMEM63A | **4703** | HFE | **6732** | TIMM8B |
| 646 | RAE1 | **2675** | HS3ST3B1 | **4704** | RNF122 | **6733** | PPTC7 |
| 647 | TTC21B | **2676** | GAS2L3 | **4705** | ATP8B2 | **6734** | RAP2A |
| 648 | MTFMT | **2677** | NFATC4 | **4706** | RFC2 | **6735** | HNRNPA0 |
| 649 | PLCD4 | **2678** | RPS27 | **4707** | TMEM95 | **6736** | B4GALT6 |
| 650 | BRI3 | **2679** | ITM2C | **4708** | NUP85 | **6737** | BLVRA |
| 651 | ARGLU1 | **2680** | CUTA | **4709** | UCK2 | **6738** | TSPAN33 |
| 652 | TMEM87A | **2681** | BMP8B | **4710** | SLC16A7 | **6739** | SDF2 |
| 653 | HEMK2 | **2682** | GPR34 | **4711** | MRPL9 | **6740** | MIOS |
| 654 | PLBD2 | **2683** | TXNDC5 | **4712** | BRD8 | **6741** | GPATCH4 |
| 655 | GYPA | **2684** | GYPB | **4713** | CLK4 | **6742** | TSPYL1 |
| 656 | KYAT3 | **2685** | ZNHIT3 | **4714** | C1QTNF7 | **6743** | RPL27A |
| 657 | RAB33A | **2686** | RPL9 | **4715** | TTLL2 | **6744** | ZC3H8 |
| 658 | TMEM135 | **2687** | UBE2K | **4716** | STXBP3 | **6745** | DGCR2 |
| 659 | DDX52 | **2688** | ATF7 | **4717** | CD79A | **6746** | SNRPG |
| 660 | CPSF4L | **2689** | SETD7 | **4718** | NR4A1 | **6747** | SMARCAL1 |
| 661 | GGA2 | **2690** | RPL8 | **4719** | FTO | **6748** | RTEL1 |
| 662 | RTEL1-TNFRSF6B | **2691** | AKR1A1 | **4720** | CYB5R3 | **6749** | PLEK |
| 663 | IGHV1-3 | **2692** | KMT5A | **4721** | RAB11B | **6750** | TM2D2 |
| 664 | PPP1CA | **2693** | CNOT10 | **4722** | NFAM1 | **6751** | CS |
| 665 | SEPTIN11 | **2694** | HERPUD1 | **4723** | CENPO | **6752** | ZBTB21 |
| 666 | MIGA2 | **2695** | CD46 | **4724** | TM2D1 | **6753** | PRPF40A |
| 667 | NYNRIN | **2696** | FBXO31 | **4725** | XRN1 | **6754** | POLR3F |
| 668 | NCK1 | **2697** | CDR2L | **4726** | LRATD2 | **6755** | CEP83 |
| 669 | IGHMBP2 | **2698** | RABGAP1L | **4727** | OARD1 | **6756** | DAP |
| 670 | DNPEP | **2699** | MATK | **4728** | DIAPH2 | **6757** | GTF2H5 |
| 671 | IMMP2L | **2700** | Mgu | **4729** | UROS | **6758** | PCYOX1L |
| 672 | RPS10 | **2701** | RELT | **4730** | RPL13 | **6759** | FAM53B |
| 673 | EGFL8 | **2702** | WDR25 | **4731** | CSRP1 | **6760** | IL15 |
| 674 | KCTD6 | **2703** | SNX1 | **4732** | IFNK | **6761** | IF1FA |
| 675 | KCNE3 | **2704** | DET1 | **4733** | APH1B | **6762** | PLXNB2 |
| 676 | PALLD | **2705** | CHMP2B | **4734** | TMSB4X | **6763** | TRAPPC12 |
| 677 | ERLEC1 | **2706** | LRP1 | **4735** | CCHCR1 | **6764** | NACC2 |
| 678 | MRPL41 | **2707** | MRPL27 | **4736** | DDN | **6765** | VPS37C |
| 679 | SOCS2 | **2708** | TPST1 | **4737** | CCNG1 | **6766** | OGFOD1 |
| 680 | SPRED1 | **2709** | ARHGEF10L | **4738** | BPTF | **6767** | ADAM12 |
| 681 | NDUFS6 | **2710** | TTPAL | **4739** | SLC66A3 | **6768** | PPRC1 |
| 682 | ARID1B | **2711** | ADAM9 | **4740** | ENTPD7 | **6769** | FBXW5 |
| 683 | C5AR2 | **2712** | RIOX2 | **4741** | ERN2 | **6770** | MYSM1 |
| 684 | DBF4 | **2713** | ZKSCAN8 | **4742** | EOGT | **6771** | SEC24D |
| 685 | TUBGCP2 | **2714** | NCAPH | **4743** | FGF10 | **6772** | GUCY1B1 |
| 686 | MCM2 | **2715** | CCNL1 | **4744** | OTUD6B | **6773** | ATP6V1H |
| 687 | MGAT5 | **2716** | MASP1 | **4745** | SELENOI | **6774** | FLOT1 |
| 688 | SLC19A1 | **2717** | CDKN1A | **4746** | TLE6 | **6775** | MAPK14 |
| 689 | AHNAK | **2718** | PSMD4 | **4747** | LSM11 | **6776** | MYO1G |
| 690 | KIF20A | **2719** | ADAMTS6 | **4748** | ESD | **6777** | SMIM26 |
| 691 | ATL3 | **2720** | RPL35A | **4749** | MITF | **6778** | TLK1 |
| 692 | WASHC5 | **2721** | PPIH | **4750** | RASGRF1 | **6779** | TOMM20 |
| 693 | VDAC2 | **2722** | TARBP1 | **4751** | MRPL39 | **6780** | UBE2L3 |
| 694 | FZD10 | **2723** | SWAP70 | **4752** | SYP | **6781** | MAPDA |
| 695 | YWHAZ | **2724** | SLC25A43 | **4753** | RHOA | **6782** | SNTB2 |
| 696 | AHCYL1 | **2725** | TUBB4B | **4754** | CD9 | **6783** | UQCRFS1 |
| 697 | CDCA3 | **2726** | MYO9B | **4755** | IL2RA | **6784** | DSTN |
| 698 | SRGN | **2727** | ARHGEF11 | **4756** | ATP11C | **6785** | ALG1 |
| 699 | PLXNB1 | **2728** | RCCD1 | **4757** | PSMB3 | **6786** | PPP1R14B |
| 700 | ANXA4 | **2729** | TMEM86B | **4758** | PRICKLE3 | **6787** | TTC33 |
| 701 | PDE12 | **2730** | HIGD1A | **4759** | DLD | **6788** | TMEM219 |
| 702 | RNF7 | **2731** | FLAD1 | **4760** | ARL8B | **6789** | PAPOLA |
| 703 | WAC | **2732** | KCNS3 | **4761** | RAC1 | **6790** | RAB35 |
| 704 | FAM53A | **2733** | WDR44 | **4762** | YBEY | **6791** | CSNK1G2 |
| 705 | HSD17B10 | **2734** | PRAMEF8 | **4763** | SLC22A14 | **6792** | NOM1 |
| 706 | UBE2W | **2735** | LAMC3 | **4764** | AMER1 | **6793** | CDC20 |
| 707 | SHQ1 | **2736** | CMC4 | **4765** | MTCP1 | **6794** | SUN2 |
| 708 | NPTN | **2737** | HMMR | **4766** | C3 | **6795** | GOLPH3L |
| 709 | SERPINB7 | **2738** | THUMPD3 | **4767** | PRSS36 | **6796** | RAI14 |
| 710 | RBM15B | **2739** | SNX9 | **4768** | CHD4 | **6797** | ABHD15 |
| 711 | TRAF3IP3 | **2740** | TAX1BP1 | **4769** | GATAD2B | **6798** | ALDH2 |
| 712 | ZBTB8A | **2741** | DGKG | **4770** | ASPM | **6799** | SLC12A6 |
| 713 | TAC4 | **2742** | SNX22 | **4771** | DCBLD2 | **6800** | PDP2 |
| 714 | PARVG | **2743** | NDUFA3 | **4772** | PMM2 | **6801** | SDHB |
| 715 | SRP9 | **2744** | PDHA1 | **4773** | CXCL16 | **6802** | TEX12 |
| 716 | PREB | **2745** | TAX1BP3 | **4774** | PIN1 | **6803** | SDHAF4 |
| 717 | ATP5MF | **2746** | PHF19 | **4775** | AARS1 | **6804** | CCAR2 |
| 718 | UBE3C | **2747** | DNAJB11 | **4776** | NAPRT | **6805** | ZDHHC24 |
| 719 | CPSF3 | **2748** | TVP23A | **4777** | SNRPF | **6806** | ITPRIPL1 |
| 720 | RNF25 | **2749** | IL27 | **4778** | SLC25A39 | **6807** | ADRB2 |
| 721 | ITGB2 | **2750** | KCNB1 | **4779** | FIS1 | **6808** | MT-RNR1 |
| 722 | SLC4A7 | **2751** | ADD1 | **4780** | NTMT1 | **6809** | SEPHS1 |
| 723 | MPND | **2752** | NDUFB4 | **4781** | FCRLA | **6810** | FCRL1 |
| 724 | STK10 | **2753** | AMELX | **4782** | LACC1 | **6811** | LIMA1 |
| 725 | PRXL2C | **2754** | NATD1 | **4783** | SNRPB2 | **6812** | AHSA2P |
| 726 | SCRN3 | **2755** | PPP1R15A | **4784** | SIN3B | **6813** | ATP13A2 |
| 727 | FAAP20 | **2756** | PGLS | **4785** | HEL-S-304 | **6814** | PRKCI |
| 728 | DIP2B | **2757** | POLE3 | **4786** | MSH5 | **6815** | MSH5-SAPCD1 |
| 729 | ZFYVE16 | **2758** | SMIM3 | **4787** | WDR43 | **6816** | SDHD |
| 730 | GNG5 | **2759** | TMEM109 | **4788** | XPNPEP3 | **6817** | ADGRA1 |
| 731 | FER | **2760** | GTPBP1 | **4789** | RHEBL1 | **6818** | PBXIP1 |
| 732 | HDGF | **2761** | UBTD1 | **4790** | GPM6B | **6819** | SWT1 |
| 733 | GRB10 | **2762** | APLP2 | **4791** | HEATR1 | **6820** | SLX9 |
| 734 | APOBEC1 | **2763** | TOR4A | **4792** | ADAM22 | **6821** | DPH3 |
| 735 | RAD23B | **2764** | CTU1 | **4793** | POLR1C | **6822** | ZFP3 |
| 736 | AP4E1 | **2765** | NSD1 | **4794** | TSSK6 | **6823** | RPS2 |
| 737 | OK/KNS-cl.7 | **2766** | TFCP2 | **4795** | PAFAH1B2 | **6824** | DNTTIP2 |
| 738 | POLD1 | **2767** | TPBGL | **4796** | LACTB | **6825** | HSPA5 |
| 739 | AP1B1 | **2768** | EZH1 | **4797** | ATP2A3 | **6826** | BFAR |
| 740 | HDAC4 | **2769** | GAPDH | **4798** | IFITM2 | **6827** | BEST1 |
| 741 | RTN1 | **2770** | ARHGAP9 | **4799** | PHKG2 | **6828** | FNDC3A |
| 742 | EMG1 | **2771** | UGP2 | **4800** | PROZ | **6829** | DDX47 |
| 743 | NME7 | **2772** | MROH2A | **4801** | KLF10 | **6830** | BACH1 |
| 744 | BRIP1 | **2773** | SLC37A1 | **4802** | SMC4 | **6831** | PTCD1 |
| 745 | ILVBL | **2774** | TRIM16 | **4803** | DZIP1 | **6832** | RASGRP4 |
| 746 | ACSBG1 | **2775** | ELOA | **4804** | SSBP2 | **6833** | POLR3A |
| 747 | DHX15 | **2776** | AAAS | **4805** | HIGD2A | **6834** | LDHA |
| 748 | GPR155 | **2777** | ANP32B | **4806** | POU5F2 | **6835** | PARS2 |
| 749 | TMEM9B | **2778** | CCN4 | **4807** | CIB1 | **6836** | UFM1 |
| 750 | CARMIL1 | **2779** | GADD45B | **4808** | HERC1 | **6837** | CTBS |
| 751 | PAFAH1B3 | **2780** | CDK4 | **4809** | MRC1 | **6838** | ZBTB45 |
| 752 | UBR4 | **2781** | MMAB | **4810** | CD44 | **6839** | ANKRD34A |
| 753 | CPNE1 | **2782** | TSTD1 | **4811** | MKRN2 | **6840** | ITGAX |
| 754 | ARL5B | **2783** | SLC6A4 | **4812** | HTT | **6841** | GOLGB1 |
| 755 | EIF5B | **2784** | CEND1 | **4813** | PPM1F | **6842** | CPT1A |
| 756 | LNPK | **2785** | HMGN5 | **4814** | CCAR1 | **6843** | ARL4A |
| 757 | MRPL36 | **2786** | TRAPPC13 | **4815** | ELF4 | **6844** | PNKD |
| 758 | MR1 | **2787** | CFAP44 | **4816** | ANKHD1 | **6845** | EEF2 |
| 759 | TFRC | **2788** | MSANTD2 | **4817** | APLF | **6846** | TRAPPC3 |
| 760 | ZNHIT6 | **2789** | DES | **4818** | tmp_locus_29 | **6847** | NDUFA4 |
| 761 | HERC3 | **2790** | LOX | **4819** | NUPR1 | **6848** | ABCA2 |
| 762 | GLUL | **2791** | PIG59 | **4820** | NPAS1 | **6849** | PALS1 |
| 763 | MPP5 | **2792** | HARS1 | **4821** | HARS | **6850** | ACTA2 |
| 764 | YAE1 | **2793** | EHD1 | **4822** | MPP1 | **6851** | SLC38A2 |
| 765 | MYG1 | **2794** | VPS25 | **4823** | SLC39A11 | **6852** | WDCP |
| 766 | RCE1 | **2795** | BCL2L12 | **4824** | RBBP9 | **6853** | LMO2 |
| 767 | BAHCC1 | **2796** | NBL1 | **4825** | KLHL5 | **6854** | IDH1 |
| 768 | ZFP28 | **2797** | POLB | **4826** | FANCG | **6855** | CCDC88B |
| 769 | TIMM10B | **2798** | STON2 | **4827** | LRRC8B | **6856** | PAPSS1 |
| 770 | UTP11 | **2799** | ACVR1 | **4828** | KY | **6857** | MRPS35 |
| 771 | MRPS28 | **2800** | TXLNA | **4829** | KDM4C | **6858** | CACNA2D1 |
| 772 | CDK12 | **2801** | AGPAT4 | **4830** | PDE3A | **6859** | HKDC1 |
| 773 | TOP1 | **2802** | FOXN2 | **4831** | ESPL1 | **6860** | SELENOS |
| 774 | NUP160 | **2803** | EHMT1 | **4832** | GPR176 | **6861** | PRPF8 |
| 775 | UBE2H | **2804** | DHPS | **4833** | RHBDF2 | **6862** | SMARCD3 |
| 776 | NES | **2805** | STAP1 | **4834** | ACAA2 | **6863** | JUN |
| 777 | MTX2 | **2806** | LITAF | **4835** | AGGF1 | **6864** | DPYSL3 |
| 778 | RNF10 | **2807** | SMC6 | **4836** | SNRK | **6865** | CCM2 |
| 779 | FBXW4 | **2808** | CCT2 | **4837** | HEL-S-100n | **6866** | TRIM14 |
| 780 | MIGA1 | **2809** | GABPB1 | **4838** | CAVIN1 | **6867** | TMEM94 |
| 781 | PBRM1 | **2810** | TERF1 | **4839** | CTU2 | **6868** | MTCH1 |
| 782 | SLC30A4 | **2811** | DOCK10 | **4840** | ZC3H12D | **6869** | SERINC5 |
| 783 | PTGR1 | **2812** | FAS | **4841** | FASN | **6870** | OPTN |
| 784 | SLC9A7 | **2813** | RAP2B | **4842** | TDRKH | **6871** | INO80C |
| 785 | PANX1 | **2814** | E4F1 | **4843** | TMEM208 | **6872** | SPO11 |
| 786 | LRRFIP1 | **2815** | KASH5 | **4844** | NUDT6 | **6873** | NT5C3B |
| 787 | MARS2 | **2816** | VPS13B | **4845** | RERG | **6874** | TRIO |
| 788 | RAG1 | **2817** | PAN3 | **4846** | RTN4 | **6875** | TTC28 |
| 789 | CLK2 | **2818** | PER3 | **4847** | SPATA46 | **6876** | ENKUR |
| 790 | KLF13 | **2819** | CIITA | **4848** | SLIT3 | **6877** | H1-0 |
| 791 | APEX2 | **2820** | LAMTOR5 | **4849** | GAS7 | **6878** | TOP2B |
| 792 | NIPSNAP2 | **2821** | NCOA4 | **4850** | GLMP | **6879** | CH25H |
| 793 | TM4SF19 | **2822** | EEPD1 | **4851** | BATF2 | **6880** | CMKLR1 |
| 794 | PIAS1 | **2823** | PGP | **4852** | INTS11 | **6881** | BRWD1 |
| 795 | KARS1 | **2824** | DDX3X | **4853** | CD48 | **6882** | GLE1 |
| 796 | LRPAP1 | **2825** | HSD11B1 | **4854** | TMEM128 | **6883** | CACNB3 |
| 797 | VRK1 | **2826** | DCTN6 | **4855** | CSRP2 | **6884** | VAMP3 |
| 798 | REEP3 | **2827** | SIRT2 | **4856** | DGCR8 | **6885** | GRPEL1 |
| 799 | LRRC40 | **2828** | MLH3 | **4857** | CEBPB | **6886** | ABRAXAS2 |
| 800 | RAN | **2829** | CST3 | **4858** | GATAD1 | **6887** | UMPS |
| 801 | PPP1R3F | **2830** | PELO | **4859** | PPL | **6888** | OAZ3 |
| 802 | AGRP | **2831** | LAMP2 | **4860** | KSR1 | **6889** | MAP3K1 |
| 803 | UBE4A | **2832** | FOXP4 | **4861** | CEP290 | **6890** | CUL1 |
| 804 | MPP2 | **2833** | DLG2 | **4862** | TRAFD1 | **6891** | TXNRD1 |
| 805 | TASP1 | **2834** | CFAP184 | **4863** | NIBAN2 | **6892** | FAM129B |
| 806 | TCERG1L | **2835** | TRAF5 | **4864** | RAB1B | **6893** | EXOG |
| 807 | ASPH | **2836** | ATRN | **4865** | INTS5 | **6894** | PIGL |
| 808 | MBIP | **2837** | TIFAB | **4866** | ZSCAN22 | **6895** | TMC6 |
| 809 | VGLL3 | **2838** | VASP | **4867** | NECAP2 | **6896** | ACOD1 |
| 810 | TNK2 | **2839** | TAF1D | **4868** | LAPTM4B | **6897** | VAV2 |
| 811 | PGBD1 | **2840** | HIC1 | **4869** | TET1 | **6898** | PKM |
| 812 | TMEM62 | **2841** | REXO2 | **4870** | PGM1 | **6899** | PELP1 |
| 813 | NOB1 | **2842** | CNOT8 | **4871** | PUM1 | **6900** | CCDC71L |
| 814 | ANGPTL8 | **2843** | NFKBID | **4872** | TNFAIP3 | **6901** | CNN3 |
| 815 | MGRN1 | **2844** | POMGNT1 | **4873** | DLGAP5 | **6902** | UBE2D1 |
| 816 | FBXW2 | **2845** | RILPL1 | **4874** | ZSCAN30 | **6903** | ARRDC2 |
| 817 | NEDD9 | **2846** | PLK2 | **4875** | LMBR1L | **6904** | TPCN2 |
| 818 | RAB38 | **2847** | PLSCR4 | **4876** | CCND1 | **6905** | HNRNPA1 |
| 819 | SYPL1 | **2848** | SYPL | **4877** | COMTD1 | **6906** | LUZP1 |
| 820 | ARHGDIB | **2849** | GPR160 | **4878** | RFTN1 | **6907** | CASP4 |
| 821 | SMC1B | **2850** | HIKESHI | **4879** | PSMA2 | **6908** | DKFZp686D09174 |
| 822 | DBI | **2851** | WTIP | **4880** | SLC29A3 | **6909** | GNA12 |
| 823 | SPATA1 | **2852** | SLC9A5 | **4881** | ASB6 | **6910** | PEX2 |
| 824 | DOP1B | **2853** | HMOX2 | **4882** | CNN2 | **6911** | WLS |
| 825 | FADS6 | **2854** | POFUT1 | **4883** | CALU | **6912** | GK |
| 826 | PRDM9 | **2855** | RGS18 | **4884** | ARL5C | **6913** | MRPL24 |
| 827 | SOX4 | **2856** | FOXP3 | **4885** | ARSB | **6914** | RAP1GAP2 |
| 828 | ACBD3 | **2857** | FAM178B | **4886** | TAMM41 | **6915** | PARP3 |
| 829 | PPP2CB | **2858** | CDH23 | **4887** | MAGED2 | **6916** | SAMD4B |
| 830 | ATP5MC3 | **2859** | ATP5G3 | **4888** | CLEC4E | **6917** | SVBP |
| 831 | MEDAG | **2860** | BABAM2 | **4889** | RPS6 | **6918** | RUFY2 |
| 832 | TRIM36 | **2861** | TPRKB | **4890** | AS3MT | **6919** | COPS7A |
| 833 | TM9SF2 | **2862** | AKAP10 | **4891** | GNG12 | **6920** | USP9X |
| 834 | TLCD5 | **2863** | TIPRL | **4892** | CENPE | **6921** | GET3 |
| 835 | BOLA3 | **2864** | CCDC12 | **4893** | PSMD2 | **6922** | ACOT9 |
| 836 | CAMLG | **2865** | CD80 | **4894** | CCND3 | **6923** | TMEM116 |
| 837 | MPP3 | **2866** | DLG3 | **4895** | QPCT | **6924** | PRKCG |
| 838 | NCDN | **2867** | PRKAR2B | **4896** | TREM2 | **6925** | TMEM50A |
| 839 | SMP1 | **2868** | CSNK2B | **4897** | EPHA2 | **6926** | TSHZ2 |
| 840 | KLC4 | **2869** | VRK2 | **4898** | CMAHP | **6927** | YAF2 |
| 841 | PLP2 | **2870** | ST8SIA6 | **4899** | NVL | **6928** | ETAA1 |
| 842 | RASGRF2 | **2871** | RBM5 | **4900** | CD14 | **6929** | WWP2 |
| 843 | ATF1 | **2872** | NPB | **4901** | PTEN | **6930** | TEP1 |
| 844 | HIPK3 | **2873** | YIPF4 | **4902** | PERM1 | **6931** | MED9 |
| 845 | RALGDS | **2874** | FLJ00185 | **4903** | PSIP1 | **6932** | MCL1 |
| 846 | QSOX2 | **2875** | ACER3 | **4904** | BHLHE40 | **6933** | MECR |
| 847 | ANKRD27 | **2876** | REPS2 | **4905** | ANKDD1B | **6934** | P2RY1 |
| 848 | RARG | **2877** | UBXN8 | **4906** | MKNK2 | **6935** | HSPA13 |
| 849 | CXCL3 | **2878** | YWHAB | **4907** | PPP6R3 | **6936** | DHRS9 |
| 850 | STN1 | **2879** | STON1 | **4908** | ATG9A | **6937** | PDE7A |
| 851 | RBM15 | **2880** | YDJC | **4909** | VWA8 | **6938** | KCTD15 |
| 852 | SYNGAP1 | **2881** | PELI1 | **4910** | TRUB1 | **6939** | MAIP1 |
| 853 | NEK2 | **2882** | AAMP | **4911** | GLRX3 | **6940** | PPIL3 |
| 854 | TPD52L2 | **2883** | DKFZp686A1765 | **4912** | PIP4P1 | **6941** | CDK3 |
| 855 | CDKN3 | **2884** | NHERF1 | **4913** | PCID2 | **6942** | ELF2 |
| 856 | BCL2L1 | **2885** | PIGN | **4914** | HMOX1 | **6943** | CNOT3 |
| 857 | ZRSR2P1 | **2886** | TRMT13 | **4915** | CHST9 | **6944** | PDE5A |
| 858 | SPACA9 | **2887** | ANXA5 | **4916** | PLA2G4A | **6945** | PRRC2A |
| 859 | PLD3 | **2888** | STAMBPL1 | **4917** | ELANE | **6946** | YARS2 |
| 860 | DNAJC19 | **2889** | GPR171 | **4918** | MAP4K5 | **6947** | STAMBP |
| 861 | ATL1 | **2890** | FOXM1 | **4919** | MTMR14 | **6948** | ANAPC5 |
| 862 | H4C1 | **2891** | HIST1H4H | **4920** | CLDN4 | **6949** | PRELID3B |
| 863 | BOD1L1 | **2892** | UBE2E1 | **4921** | ACADSB | **6950** | TMEM168 |
| 864 | COQ8B | **2893** | ATP8B4 | **4922** | CDC42SE2 | **6951** | DENND2D |
| 865 | PLXNB3 | **2894** | CCT7 | **4923** | SSR2 | **6952** | KLHL36 |
| 866 | CSF2RB | **2895** | MARCKS | **4924** | HNRNPA3 | **6953** | STX12 |
| 867 | MAP10 | **2896** | PPP2CA | **4925** | PSMD7 | **6954** | TMEM37 |
| 868 | NFKBIA | **2897** | SGTA | **4926** | DBN1 | **6955** | DERL2 |
| 869 | ADPRH | **2898** | UIMC1 | **4927** | PLIN4 | **6956** | JAG1 |
| 870 | SLC12A9 | **2899** | SOD1 | **4928** | CCDC117 | **6957** | ASH2L |
| 871 | RELL1 | **2900** | ADAR | **4929** | GSPT2 | **6958** | ZWINT |
| 872 | PRKAR1A | **2901** | RAPSN | **4930** | PLEKHF2 | **6959** | ASH1L |
| 873 | TNFSF12 | **2902** | TWEAK | **4931** | MCCC1 | **6960** | TMEM263 |
| 874 | SLC35C2 | **2903** | NT5DC2 | **4932** | HNRNPF | **6961** | INAFM1 |
| 875 | NFIL3 | **2904** | METTL25 | **4933** | WDR41 | **6962** | RPS14 |
| 876 | RASA2 | **2905** | FNBP4 | **4934** | COLGALT2 | **6963** | NOL6 |
| 877 | CCL25 | **2906** | LRRC8C | **4935** | VAV1 | **6964** | GLIPR2 |
| 878 | LPIN1 | **2907** | SIK3 | **4936** | RRP9 | **6965** | RPP40 |
| 879 | FMNL2 | **2908** | ING3 | **4937** | CANT1 | **6966** | DUSP8 |
| 880 | EIF2B3 | **2909** | FGFR1OP2 | **4938** | MDC1 | **6967** | CCDC142 |
| 881 | SPATA6 | **2910** | RABEP2 | **4939** | KLHL26 | **6968** | EXOC7 |
| 882 | NFYA | **2911** | ATP6V0D2 | **4940** | GAK | **6969** | PIP5K1C |
| 883 | GTPBP8 | **2912** | UBIAD1 | **4941** | ADIPOR1 | **6970** | RBM39 |
| 884 | LTBP2 | **2913** | LTBP3 | **4942** | DAD1 | **6971** | AARSD1 |
| 885 | PI4KB | **2914** | RABAC1 | **4943** | ZMYM3 | **6972** | ABHD4 |
| 886 | PPP6R1 | **2915** | METTL21A | **4944** | CLEC9A | **6973** | SLC25A20 |
| 887 | NFS1 | **2916** | PDS5A | **4945** | TRPM2 | **6974** | TMTC1 |
| 888 | DCAF8 | **2917** | RSPRY1 | **4946** | IL1R1 | **6975** | DEGS1 |
| 889 | GSKIP | **2918** | FBXO10 | **4947** | HVCN1 | **6976** | GUCA1A |
| 890 | BRMS1L | **2919** | MLH1 | **4948** | GLB1 | **6977** | HPS4 |
| 891 | ERICH1 | **2920** | ASAP3 | **4949** | RAB9A | **6978** | PGM3 |
| 892 | ACRBP | **2921** | IL18RAP | **4950** | SDR39U1 | **6979** | ANP32A |
| 893 | DIP2A | **2922** | TSEN2 | **4951** | NNT | **6980** | KAT7 |
| 894 | MOGS | **2923** | OXCT1 | **4952** | KIFAP3 | **6981** | REV3L |
| 895 | CBY1 | **2924** | ATF4 | **4953** | OS9 | **6982** | ERO1A |
| 896 | ACOT2 | **2925** | RANBP3 | **4954** | GALNT1 | **6983** | GTF3A |
| 897 | NUDT9 | **2926** | CUL3 | **4955** | PPP3R1 | **6984** | FBXO33 |
| 898 | GSTCD | **2927** | ARMC1 | **4956** | FGR | **6985** | KLHDC4 |
| 899 | YTHDF2 | **2928** | RCC1L | **4957** | CDKN2D | **6986** | MPG |
| 900 | ULK2 | **2929** | MRPL19 | **4958** | HOMER1 | **6987** | MTFR2 |
| 901 | TTLL9 | **2930** | DCLK1 | **4959** | CFAP100 | **6988** | BOK |
| 902 | LSMEM2 | **2931** | PRKAA1 | **4960** | SFXN5 | **6989** | USP5 |
| 903 | EED | **2932** | ZFP36L1 | **4961** | BRF1 | **6990** | EIF5 |
| 904 | GMPR | **2933** | GMPR2 | **4962** | GSPT1 | **6991** | SNRPD3 |
| 905 | TDO2 | **2934** | PGAM1 | **4963** | CALHM5 | **6992** | EPN1 |
| 906 | WDR19 | **2935** | NEBL | **4964** | OSM | **6993** | GNB4 |
| 907 | NIPAL1 | **2936** | SYNE3 | **4965** | LSP1 | **6994** | RGS9 |
| 908 | RAPGEF6 | **2937** | CHCHD1 | **4966** | DDX19A | **6995** | HSPA4L |
| 909 | AQP7 | **2938** | AQP9 | **4967** | PSMB9 | **6996** | ARPC3 |
| 910 | MLXIP | **2939** | DPP4 | **4968** | CFP | **6997** | GMFB |
| 911 | ADAMTS12 | **2940** | PPM1K | **4969** | MIR22HG | **6998** | NUDCD2 |
| 912 | ANKRD34B | **2941** | POLD4 | **4970** | POLDIP2 | **6999** | LETMD1 |
| 913 | NCF1 | **2942** | TSKS | **4971** | LMNA | **7000** | UBASH3B |
| 914 | CARD11 | **2943** | SARNP | **4972** | ALAS1 | **7001** | ING2 |
| 915 | GPR21 | **2944** | GPR162 | **4973** | NOL9 | **7002** | PI4K2A |
| 916 | PTPN22 | **2945** | ARHGAP23 | **4974** | MAPKAPK5 | **7003** | PLA2G15 |
| 917 | CASP1 | **2946** | HCAR2 | **4975** | RAB32 | **7004** | CC2D2A |
| 918 | BCDIN3D | **2947** | OAZ2 | **4976** | CUX1 | **7005** | PURA |
| 919 | URB2 | **2948** | RPS3 | **4977** | SNX4 | **7006** | DYNLT2B |
| 920 | HEXB | **2949** | IRF8 | **4978** | AOAH | **7007** | ACAP3 |
| 921 | CTRL | **2950** | DUS4L | **4979** | PP35 | **7008** | DUS4L-BCAP29 |
| 922 | RHAG | **2951** | ITGB5 | **4980** | CAMKK2 | **7009** | HAVCR2 |
| 923 | DCAKD | **2952** | SMC3 | **4981** | SNRNP48 | **7010** | PISD |
| 924 | DNAJB6 | **2953** | DDX49 | **4982** | MSRB3 | **7011** | HERC6 |
| 925 | GPC1 | **2954** | TRAPPC6A | **4983** | TMEM65 | **7012** | ATG12 |
| 926 | RPGRIP1 | **2955** | PLP1 | **4984** | GAPVD1 | **7013** | CLDN20 |
| 927 | SLA | **2956** | SEPSECS | **4985** | ST6GALNAC6 | **7014** | EIF2AK1 |
| 928 | NLRC5 | **2957** | IFT88 | **4986** | CORO1C | **7015** | PIBF1 |
| 929 | GTDC1 | **2958** | MRPL23 | **4987** | AQR | **7016** | EML2 |
| 930 | DHX38 | **2959** | RAB7B | **4988** | ANGPTL4 | **7017** | ATP6V1C1 |
| 931 | TAF11 | **2960** | POLR3D | **4989** | USP50 | **7018** | RCN1 |
| 932 | HAUS6 | **2961** | CCDC15 | **4990** | HOXA10 | **7019** | CCDC82 |
| 933 | NCAPG2 | **2962** | MRPL3 | **4991** | ROR2 | **7020** | SLC44A1 |
| 934 | ALPK1 | **2963** | TBC1D31 | **4992** | ADAP2 | **7021** | CARS1 |
| 935 | CARS | **2964** | AMH | **4993** | MIF | **7022** | KCNK6 |
| 936 | TESK1 | **2965** | PIGG | **4994** | ATG3 | **7023** | CSK |
| 937 | ELK3 | **2966** | COX6B1 | **4995** | RNF149 | **7024** | TSPAN17 |
| 938 | DAPP1 | **2967** | NUCB1 | **4996** | ZC3H15 | **7025** | CPEB4 |
| 939 | PDIA4 | **2968** | ARHGEF37 | **4997** | CDK9 | **7026** | JAML |
| 940 | AXL | **2969** | SH3RF1 | **4998** | LPCAT2 | **7027** | CD274 |
| 941 | KIF1C | **2970** | PRKAR2A | **4999** | FRAT1 | **7028** | SETD2 |
| 942 | CLASP1 | **2971** | CREB3 | **5000** | MRM1 | **7029** | TUBB3 |
| 943 | PTAR1 | **2972** | SLC16A1 | **5001** | PHF5A | **7030** | DCLRE1B |
| 944 | BAZ1A | **2973** | MSRB1 | **5002** | REEP1 | **7031** | PWP2 |
| 945 | HGF | **2974** | RAB6A | **5003** | RNF213 | **7032** | STAC |
| 946 | TAGLN3 | **2975** | MTPN | **5004** | DKFZp761E1322 | **7033** | SNF8 |
| 947 | NUP43 | **2976** | IL18R1 | **5005** | HECTD2 | **7034** | UBQLN1 |
| 948 | TNFRSF9 | **2977** | GRWD1 | **5006** | WIPI1 | **7035** | SLC50A1 |
| 949 | MYBPHL | **2978** | NDUFC2 | **5007** | NRIP2 | **7036** | BRMS1 |
| 950 | CBX6 | **2979** | RBFOX2 | **5008** | FRYL | **7037** | SUGP1 |
| 951 | GSR | **2980** | SORL1 | **5009** | PDGFRB | **7038** | SLC49A4 |
| 952 | PLA2G12A | **2981** | C6 | **5010** | RNASEL | **7039** | RBM3 |
| 953 | TOR2A | **2982** | SMIM10L1 | **5011** | DTD1 | **7040** | C1D |
| 954 | FOSL2 | **2983** | DHRS4 | **5012** | ELMO1 | **7041** | CYP51A1 |
| 955 | PDS5B | **2984** | KLHL22 | **5013** | ENO4 | **7042** | CORO2A |
| 956 | AK8 | **2985** | UBE2N | **5014** | HEL-S-71 | **7043** | NOS3 |
| 957 | NANOS3 | **2986** | PIH1D2 | **5015** | LASP1 | **7044** | TSPAN11 |
| 958 | PARD6A | **2987** | AKIRIN2 | **5016** | FAM187B | **7045** | STK32A |
| 959 | SMARCC1 | **2988** | GABPB2 | **5017** | IGLC1 | **7046** | MAP1LC3B |
| 960 | DKFZp762B153 | **2989** | IGLC2 | **5018** | COMMD10 | **7047** | DAZAP1 |
| 961 | TAGAP | **2990** | FEM1C | **5019** | PSMA6 | **7048** | PCBP4 |
| 962 | TMEM255A | **2991** | FLNB | **5020** | ERCC6L2 | **7049** | VIPR1 |
| 963 | MSLNL | **2992** | RFT1 | **5021** | SLC52A1 | **7050** | PRR14L |
| 964 | R3HDM4 | **2993** | MSANTD4 | **5022** | TRAPPC9 | **7051** | LAT2 |
| 965 | SLC7A8 | **2994** | STK38L | **5023** | BBS2 | **7052** | PARP11 |
| 966 | ABCB8 | **2995** | APBB1IP | **5024** | PKN2 | **7053** | MAP4K2 |
| 967 | NDUFS7 | **2996** | DNAJB8 | **5025** | SMAD3 | **7054** | NUDT8 |
| 968 | ABCB7 | **2997** | CALCOCO1 | **5026** | SAMD12 | **7055** | PRR13 |
| 969 | SMAD9 | **2998** | JAK3 | **5027** | KPNA1 | **7056** | ATP6V1E1 |
| 970 | MIDN | **2999** | IRF2BPL | **5028** | ALAS2 | **7057** | CR2 |
| 971 | NXPH4 | **3000** | POLR2F | **5029** | TBCE | **7058** | TBX2 |
| 972 | RPS16 | **3001** | RFXAP | **5030** | CCNO | **7059** | ANKRD66 |
| 973 | MTHFR | **3002** | MAT2A | **5031** | SESN3 | **7060** | KLHL7 |
| 974 | PRIMPOL | **3003** | VANGL2 | **5032** | MCAM | **7061** | ISCA1 |
| 975 | EHD3 | **3004** | EHD2 | **5033** | AP2A2 | **7062** | USP31 |
| 976 | CCDC69 | **3005** | TMEM64 | **5034** | EPOP | **7063** | ADK |
| 977 | RGS16 | **3006** | UTP15 | **5035** | CAPN2 | **7064** | MRE11 |
| 978 | MRE11A | **3007** | TMEM97 | **5036** | BTBD3 | **7065** | CACFD1 |
| 979 | APP | **3008** | HELQ | **5037** | NUDT16 | **7066** | FYB1 |
| 980 | FYB | **3009** | OSBPL8 | **5038** | ATP6V1G3 | **7067** | DGAT2 |
| 981 | ANXA3 | **3010** | PODXL2 | **5039** | SHLD2 | **7068** | SLC11A1 |
| 982 | NRAMP1 | **3011** | TPGS1 | **5040** | DPEP1 | **7069** | PODNL1 |
| 983 | ZFP64 | **3012** | SLC37A3 | **5041** | USP1 | **7070** | SMAD5 |
| 984 | DKFZp781O1323 | **3013** | YTHDF3 | **5042** | ACTR1A | **7071** | ZBTB40 |
| 985 | NDUFS5 | **3014** | WASF2 | **5043** | FAM168B | **7072** | AKIRIN1 |
| 986 | LRRIQ3 | **3015** | RAB20 | **5044** | GANC | **7073** | ASB1 |
| 987 | NUDT21 | **3016** | GPX4 | **5045** | ALCAM | **7074** | MIPEP |
| 988 | CTNNB1 | **3017** | GCFC2 | **5046** | CCNA2 | **7075** | AMD1 |
| 989 | EID3 | **3018** | EID2B | **5047** | USP12 | **7076** | ANKS1A |
| 990 | EXOC5 | **3019** | CACUL1 | **5048** | PCDHGA9 | **7077** | TULP3 |
| 991 | ANGPTL7 | **3020** | OAT | **5049** | CADM3 | **7078** | FBLN5 |
| 992 | AK1 | **3021** | IFNAR2 | **5050** | CCNH | **7079** | RAD50 |
| 993 | BROX | **3022** | POLR1G | **5051** | CAST | **7080** | UNC93B1 |
| 994 | SAT1 | **3023** | CPSF7 | **5052** | FITM2 | **7081** | ATP1B3 |
| 995 | NAGLU | **3024** | ufHSD2 | **5053** | CD164 | **7082** | RAB13 |
| 996 | ASB5 | **3025** | DKFZp313J0816 | **5054** | TIAL1 | **7083** | BCAN |
| 997 | AGTRAP | **3026** | PLXDC2 | **5055** | CDK13 | **7084** | RAMP3 |
| 998 | AZIN1 | **3027** | SYNJ1 | **5056** | SNX14 | **7085** | PHLDA1 |
| 999 | RNF217 | **3028** | TMED3 | **5057** | AMZ2 | **7086** | ATP2A2 |
| 1000 | SSC4D | **3029** | CASTOR2 | **5058** | PGLYRP1 | **7087** | OTUB1 |
| 1001 | KBTBD7 | **3030** | USP53 | **5059** | CRYBG1 | **7088** | NPTXR |
| 1002 | DDX11 | **3031** | STMN1 | **5060** | CALR | **7089** | HUNK |
| 1003 | GNG10 | **3032** | RAF1 | **5061** | SGF29 | **7090** | SDK1 |
| 1004 | SNX6 | **3033** | CEP164 | **5062** | AUP1 | **7091** | GLI3 |
| 1005 | MAP7D3 | **3034** | EXTL3 | **5063** | SLU7 | **7092** | IGF1 |
| 1006 | EMILIN2 | **3035** | HBEGF | **5064** | MBD2 | **7093** | AURKAIP1 |
| 1007 | PCDHGA12 | **3036** | HAGH | **5065** | PARD3B | **7094** | GFOD1 |
| 1008 | MICOS10 | **3037** | TMEM186 | **5066** | GUCY1A2 | **7095** | PRMT7 |
| 1009 | COA8 | **3038** | TMEM171 | **5067** | ZC3H14 | **7096** | RBM25 |
| 1010 | TMOD1 | **3039** | ERMAP | **5068** | ORC3 | **7097** | HEXD |
| 1011 | CNOT9 | **3040** | DHX36 | **5069** | ALG10B | **7098** | LIF |
| 1012 | SPHK1 | **3041** | TMEM269 | **5070** | SELPLG | **7099** | CC2D1A |
| 1013 | SSPN | **3042** | SCAI | **5071** | TMEM165 | **7100** | PF4 |
| 1014 | PFDN2 | **3043** | CXCR6 | **5072** | OPN3 | **7101** | CAMK2N2 |
[truncated: 536,581 more chars]
